# Euphosantianane A–D: Antiproliferative Premyrsinane Diterpenoids from the Endemic Egyptian Plant *Euphorbia Sanctae-Catharinae*

**DOI:** 10.3390/molecules23092221

**Published:** 2018-09-01

**Authors:** Mohamed-Elamir F. Hegazy, Ahmed R. Hamed, Mahmoud A. A. Ibrahim, Zienab Talat, Eman H. Reda, Nahla S. Abdel-Azim, Faiza M. Hammouda, Seikou Nakamura, Hisashi Matsuda, Eman G. Haggag, Paul W. Paré, Thomas Efferth

**Affiliations:** 1 Chemistry of Medicinal Plants Department, National Research Centre, 33 El-Bohouth St., Dokki, Giza 12622, Egypt; Elamir77@live.com (M.-E.F.H.); n1ragab2004@yahoo.com (A.R.H.); nahlaabdelazim@yahoo.com (N.S.A.-A.); fmhammouda@yahoo.com (F.M.H.); 2 Department of Pharmaceutical Biology, Institute of Pharmacy and Biochemistry, University of Mainz, Staudinger Weg 5, 55128 Mainz, Germany; efferth@uni-mainz.de; 3 Biology Unit, Central Laboratory for Pharmaceutical and Drug Industries Research Division, National Research Centre, 33 El-Bohouth St., Dokki, Giza 12622, Egypt; 4 Computational Chemistry Laboratory, Chemistry Department, Faculty of Science, Minia University, Minia 61519, Egypt; m.ibrahim@compchem.net; 5 Phytochemistry Lab., National Organization for Drug Control and Research, Giza 12622, Egypt; zizishakour@yahoo.com (Z.T.); dremanhusseinreda@gmail.com (E.H.R.); 6 Department of Pharmacognosy, Kyoto Pharmaceutical University, Misasagi, Yamashina-ku, Kyoto 607-8412, Japan; naka@mb.kyoto-phu.ac.jp (S.N.); matsuda@mb.kyoto-phu.ac.jp (H.M.); 7 Department of Pharmacognosy, Helwan University, Cairo 12622, Egypt; wemisr@hotmail.com; 8 Department of Chemistry and Biochemistry, Texas Tech University, Lubbock, TX 79409, USA

**Keywords:** *Euphorbia sanctae-catharinae*, Euphorbiaceae, diterpenes, flavonoids, TDDTF-ECD, tumor anti-proliferative activity

## Abstract

*Euphorbia* species are rich in diterpenes. A solvent extraction of *Euphorbia sanctae-catharinae*, a species indigenous to the Southern Sinai of Egypt, afforded several premyrsinane diterpenoids (**1**–**4**) as well as previously reported metabolites (**5**–**13**) that included three flavonoids. Isolated compounds were chemically characterized by spectroscopic analysis. Identified compounds were bioassayed for anti-proliferative activity in vitro against colon (Caco-2) and lung (A549) tumor cell lines. Compound **9** exhibited robust anti-proliferative activity against A549 cells (IC_50_ = 3.3 µM). Absolute configurations for **8** versus **9** were determined by experimental and TDDFT-calculated electronic circular dichorism (ECD) spectra.

## 1. Introduction

The genus Euphorbia is the largest genus in the family Euphorbiaceae. The genus comprises over 2000 species worldwide [1] and its global distribution includes more than 750 species in Africa and 42 indigenous to Egypt [2]. All plants in the genus share a poisonous, milky-white, latex-like sap as well as a unique floral structure, in which each flower in the cluster is reduced to its barest of essential parts for sexual reproduction. Euphorbia species have been widely used in folk medicine for the treatment of diarrhea, inflammation, and swellings and the milky sap has been tested as a wart remover [3,4,5]. Some species have been used in the treatment of dermatosis, paralysis and body pain as well as a poultice for skin ulcerations [6]. A number of biological activities ranging from cytotoxic [7], hepatoprotective [8,9], antispasmodic [10], anti-inflammatory [11], antibacterial [12,13], antifungal [10] and anti-mutagenic [14], antiviral [15] have been reported.

Some *Euphorbia* species are indigenous to the Sinai Peninsula [16,17] with *E. sanctae-catharinae* (also known as St. Katherine spurge) endemic to the Gebel Wadi, a system of deep/dry river valleys separated by the high elevation Katherine Mountains. As part of our research to investigate and biologically evaluate the wild Egyptian plants [18,19,20,21,22,23,24,25], herein, it is the first phytochemical investigation of *E. sanctae-catharinae* that specifically targets secondary metabolites that may exhibit anti-tumor activity. 

## 2. Results and Discussion

**Figure 1:**
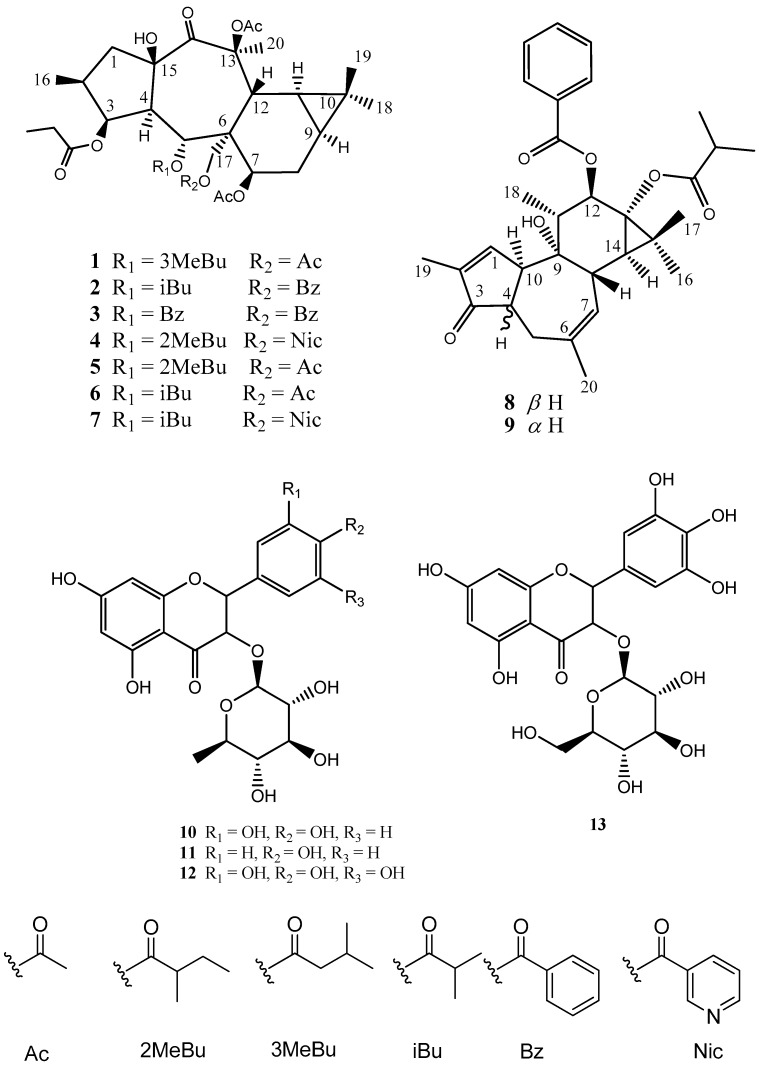
Identified compounds from *Euphorbia sanctae-catharinae.* Ac = acetyl, Bz = benzoyl, Nic = nicotinoyl, Bu = butanoyl, iBu = isobutanoyl, 2MeBu = 2-methylbutanoyl, 2MeBu 3-dimethylbutanoyl, Nic = nicotinoyl.

A methylene chloride/methanol (1:1) extract of air-dried, aerial parts of *E. sanctae-catharinae* was separated into pure chemical components using normal and reversed phase chromatographic separations to afford new (**1**–**4**) as well as previously isolated (**5**–**13**) compounds (Figure 1).

**Figure 2:**
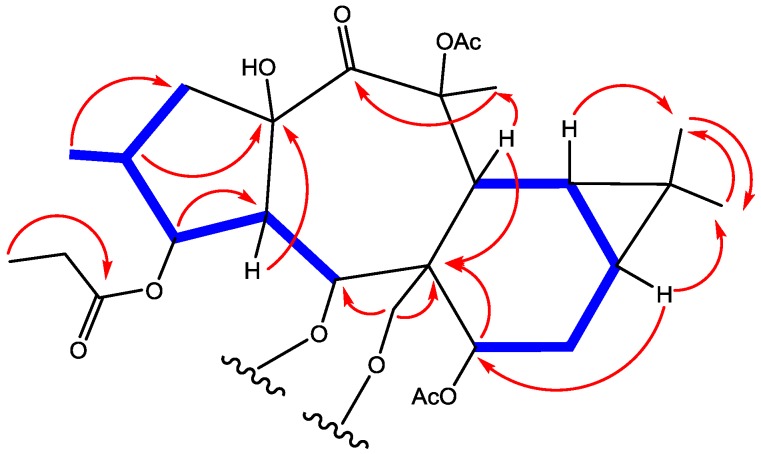
Observed DQF-COSY and HMBC correlations for **1**–**4**.

Compound **1** was obtained as colorless oil with positive optical rotation (${\left[\alpha \right]}_{D}^{25}$ + 20.0 in MeOH). HRFABMS analysis showed a molecular ion peak at *m*/*z* 673.3203 [M + Na]^+^ corresponding to a molecular formula of C_34_H_50_O_12_Na (calcd. 673.3200). The IR spectrum displayed absorption bands for OH (3532 cm^−1^) and ester carbonyl (1741 cm^−1^) groups. The ^1^H-NMR spectrum contained signals typical for three acetyl groups at δ_H_ 2.06, 2.08 and 2.09. The spectrum also displayed signals for seven methyl groups (one primary at δ_H_ 1.08 (6H), three secondary at δ_H_ 0.87, 0.90 and 0.92, four tertiary at δ_H_ 0.87, 1.04 and 1.68) and three oxygenated methine protons referred to ester functions at δ_H_ 4.48 (d, *J* = 6.6), 5.24 (dd, *J* = 3.6, 6.0), 6.18 (d, *J* = 11.4) and one oxygenated methyelene at δ_H_ 4.39 (d, *J* = 12.0) and 4.31 (d, *J* = 12.0). Additionally, two aliphatic methine δ_H_ 0.72 (m) indicated the presence of a cyclopropane moiety (Table 1). ^13^C-NMR and DEPT spectra displayed 32 carbons including five ester carbonyls (δ_C_ 170.0, 170.4, 170. 7, 174.2 and 174.3), one free keto carbon (δ_C_ 204.5), 7 methyls, 5 methylenes (one of them oxygenated), 8 methines (two of them oxygenated), and four quaternary carbons (two of them oxygenated). Ten degrees of unsaturation were deduced suggesting a tetracyclic diterpene premyrsinane skeleton. Two-dimensional NMR (COSY, HMQC and HMBC) comparisons with 7 that had been previously published suggested a 5/7/6 cyclic structure [16,26,27]. Differences in the spectroscopic data between **1** and **6** were limited to C-5. Indeed, functionality differences for Euphorbia premyrsinane diterpenoids are usually localized to C-3, C-5, C-7 and/or C-17. HRFABMS of 1 indicated the addition of a methlyene unit in comparison with 6. DEPT analysis confirmed an additional methylene group at δ_C_ 42.8 (δ_H_ 2.32, m) and correlations with signals at δ_H_ 1.97 (m) and δ_C_ 174.2 in DQF-COSY and HMBC analyses, respectively, situated the methyl as an addition to the butyrate unit [28]. Moreover, an HMBC correlation between H-5 (δ_H_ 6.18, d, *J* = 11.4) and δ_C_ 174.2 established the presence of 2-methylbutyrate at C-5 (δ_C_ 68.8). These data suggested that signals for a 2-methylbutyryl unit in **5** was replaced by 3-methylbutyryl moiety (δ_C_ 174.2, 21.4, 21.4, 26.5, 42.8) in **1** (Figure 2). This small modification was confirmed by COSY, HMBC analysis.

**Figure 3:**
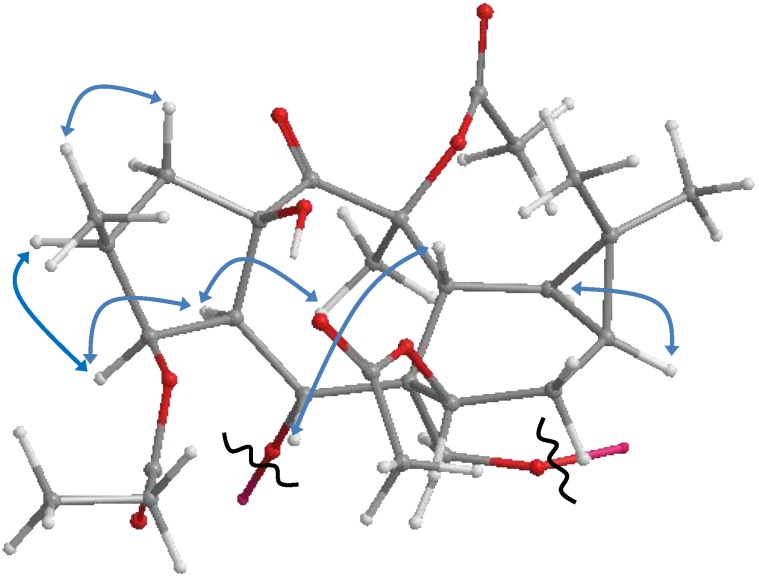
Observed NOESY correlations for **1**–**4**.

The relative configuration was elucidated as based on biosynthetic presidency. For all naturally derived myrsinol diterpenes isolated to date, the three (5/7/6) fused ring system that forms the myrsinol skeleton are joined in a trans configuration with H-4 and H_2_-17 α-oriented. Based on this initial configuration, NOE correlations between H-4 and H-1 provided evidence for an α-assignment H-1. NOE correlations between H-1α/H-2 and H-1β/CH_3_-16 established an α-orientation for H-2. NOE interactions between H-5/7-OAc, H-5/H-12 and H-7/H_2_-17 established an α-orientation for H-7 and a β-orientation for H-12. NOESY correlations observed between H-1β, H-14 and H-16 indicated the positioning of these functional groups on the same ringside consistent with a *cis* configuration (Figure 3). All stereochemical assignments are consistent with previously reported premyrsinane diterpenes [16,26]. Therefore, the structure was assigned as premyrsinol-3-propanoate-5(α-3 methyl) butyrate-7, 13, 17-triacetate (euphosantianane A).

Compound **2** was obtained as colorless oil with positive optical rotation (${\left[\alpha \right]}_{D}^{25}$ + 27.0 in MeOH). HRFABMS analysis showed a molecular ion peak at *m*/*z* 721.3206 [M + Na]^+^ corresponding to a molecular formula of C_38_H_50_O_12_Na (calcd. 721.3200). The IR spectrum displayed absorption bands for OH (3532 cm^−1^) and ester carbonyl (1741 cm^−1^) groups, as well as characteristic aromatic ring absorptions (1450 and 716 cm^−1^). 1D- and 2D-NMR spectra (Table 1, Figure 2 and Figure 3) were similar to those of previously published **7** [25,26] albeit signals for the acetate group at C-17 are replaced by a benyzoly moiety in 2. NOESY correlations were observed to be the same for both **2** and **6**. Therefore **2** was assigned as premyrsinol-3-propanoate-5-isobutyrate-7,13-diacetate-17-benzoate (euphosantianane B).

Compound **3** was obtained as colorless oil with positive optical rotation (${\left[\alpha \right]}_{D}^{25}$ + 64.0 in MeOH). HRFABMS analysis showed a molecular ion peak at *m*/*z* 755.3050 [M + Na]^+^ corresponding to a molecular formula of C_38_H_50_O_12_Na (calcd. 755.3043). The IR spectrum displayed absorption bands for OH (3532 cm^−1^) and ester carbonyl (1741 cm^−1^) groups, as well as characteristic aromatic ring absorptions (1450 and 716 cm^−1^). 1D- and 2D-NMR spectra (Table 1, Figure 2 and Figure 3) were similar to those of compound **2** except for an isoproponate group at C-5 in **2** being replaced by a second benyzoly moiety in **3**. The same NOESY correlations were detected in both 2 and 3. Therefore, the structure was assigned as premyrsinol-3-propanoate-5-benzoate-17-benzoyl (euphosantianane C).

Compound **4** was obtained as colorless oil with positive optical rotation (${\left[\alpha \right]}_{D}^{25}$ + 30.4 in MeOH). HRFABMS analysis showed a molecular ion peak at *m*/*z* 736.3312 [M + Na]^+^ corresponding to a molecular formula of C_38_H_50_O_12_Na (calcd. 736.3309). The IR spectrum displayed absorption bands for OH (3532 cm^−1^) and ester carbonyl (1741 cm^−1^) groups, as well as characteristic aromatic ring absorptions (1450 and 716 cm^−1^). 1D- and 2D-NMR spectra (Table 1, Figure 2 and Figure 3) were similar to those of 5 except that the 2-methyl butyl substitution at C-17 in 5 was replaced by a nicotedial moiety in **4**. NOESY correlations were observed to be the same in both 4 and 5 [16]. Therefore, the structure was assigned as premyrsinol-3-propanoate-5(α-2-methyl) butyrate-7,13-diacetate-17-nicotinate (euphosantianane D).

Nine known compounds have been isolated for the first time from *E. sanctae-catharinae* including five diterpenes: 7β,13β,17-*O*-triacetyl-5α-*O*-(2-methylbutyryl)-3β-*O*-propanoyl14-oxopremyrsinol (**5**) [16], premyrsinol-3-propanoate-5-isobutyrate-7,13,17-triacetate (**6**) [26], premyrsinol-3-propanoate-5-isobutyrate-7,13-triacetate-17-nicotinate (**7**) [26], 4,20-Dideoxy(4α)phorbol-12-benzoate-13-isobutyrate (**8**) [29], 4,12,20-trideoxyphorbol-13-(2,3-dimethyl) butyrate (**9**) [30]; and four flavonoid gylcosides: quercetin-3-*O*-α-rhamnopyranoside (**10**) [31], kaempferol-3-*O*-rhamnoside (**11**) [32], myricetin-3-*O*-rhamnoside (**12**) [33], quercetin-3-*O*-galactopyranoside (**13**) [34].

The potent activity of **9** rather than it epimer encourage motivated a greater examination of the absolute configurations of **8** and **9** utilizing TDDFT-ECD calculations. Conformational search was first carried out using MMFF94S force field (time-dependent density functional) within a 10 kcal/mol energy window with the use of Omega2 software, OpenEye Scientific Software, Santa Fe, NM, USA. Molecular dynamics simulation of 10 ns was then performed for each conformer in methanol.

**Figure 4:**
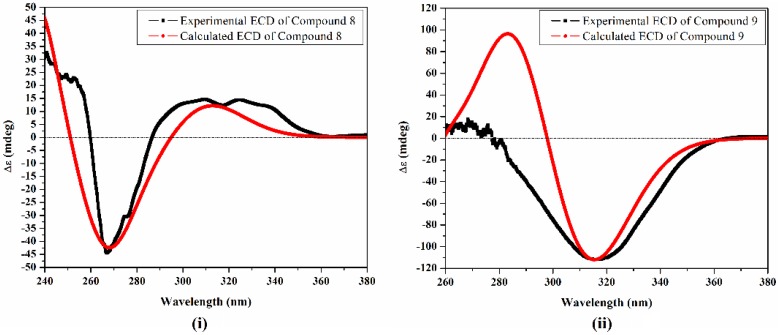
Experimental and theoretical ECD spectra for compounds **8** (**i**) and **9** (**ii**).

Uncorrelated snapshots were collected every 10 ps over 10 ns MD simulation and subjected to a geometrical optimization in methanol at the B3LYP/6-31G* level of theory, followed by frequency calculations. TDDFT calculations were then performed for each set of conformers at the same level of theory. The Boltzmann-weighted ECD (Equivalent Circulating Density) curves were generated and compared to the experimental spectra (Figure 4). The calculated ECD curves of compounds **8** and **9** gave a good agreement with the experimental data (Figure 4i,ii, respectively). A negative Cotton effect at 287 nm for the lactone ring π→π* transitions and a positive Cotton effect of 315 nm for the lactone n→π* transitions was observed for **8**, while **9** gave opposite Cotton effects (Figure 4). The TDDFT-ECD calculations and spectral data supported the conclusion that the absolute configuration at C-10 for **8** and **9** are S and R, respectively.

**Figure 5:**
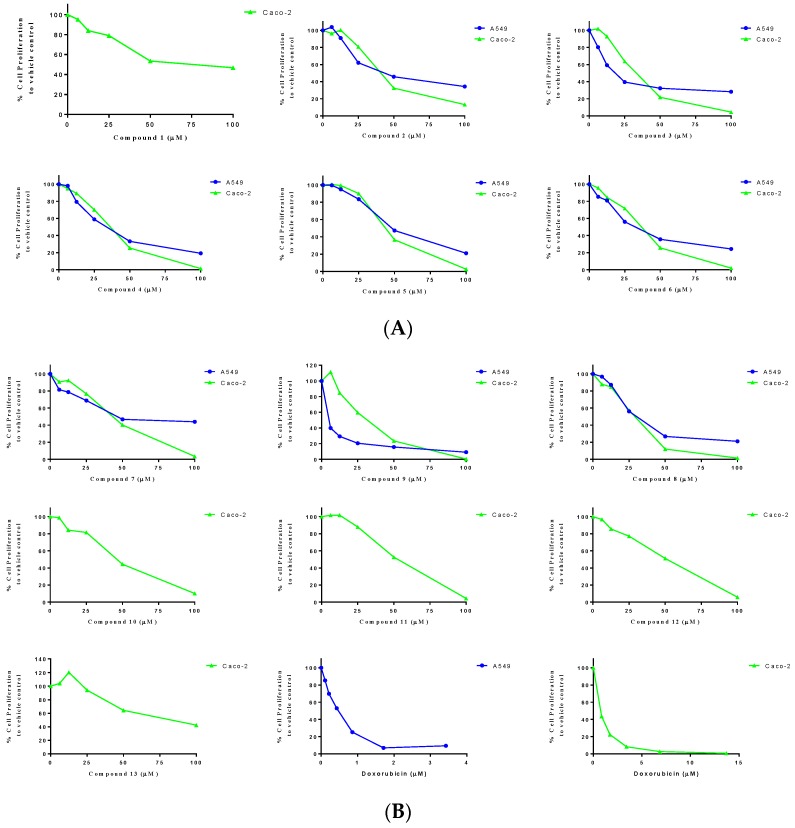
Concentration-response curve fits of the effect of isolated compounds **1**–**6** (**A**) and **7**–**13** (**B**) on the cell proliferation of Caco-2 (green triangles) or A549 (blue spheres). Cell proliferation was determined as % of vehicle control (MTT reduction assay) as detailed in the Experimental section.

**Figure 6:**
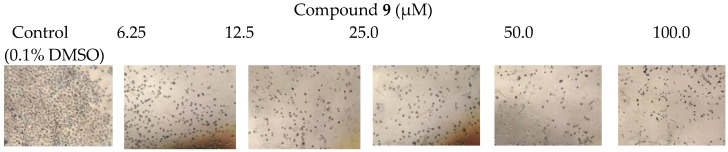
Photomicrographic images of A549 cells depict increasing morphological toxicity include cell monolayer disruption and cell shrinkage with 48 h exposure to **9** at increasing concentrations. Magnification = 150×.

Compounds **1**–**13** were tested for cytotoxic activity against human cancer cell lines of colon (Caco-2) and lung (A549) using doxorubicin HCl as positive control (Figure 5A,B and Table 2). Compound **9** showed the highest cytotoxic activity against lung cancer cells with an IC50 value of 3.3 µM (Figure 6), while an epimer of 8 exhibited cytotoxic activity against colon cancer cells with an IC_50_ of 26.1 µM.

## 3. Experimental Section

### 3.1. General Experimental Procedures

Specific rotation was measured with Perkin–Elmer-341 MC digital polarimeter (Wellesley, MA, USA) and IR spectra were collected on a JASCO FT/IR-6300 spectrometer (Easton, MD, USA). ^1^H- and ^13^C-NMR spectra were recorded in CDCl_3_ on a JEOL ECA- 600 spectrometer (600 MHz for ^1^H and 150 MHz for ^13^C) (JEOL Ltd., Tokyo, Japan). All chemical shifts (δ) are given in ppm units with reference to TMS as an internal standard and coupling constants (*J*) are reported in Hz. FAB-MS experiments were performed using a Thermo ISQ Single Quadrupole system and HR-FAB-MS experiments were performed on Fourier transform ion cyclotron mass spectrometer (Thermo Scientific, San Jose, CA, USA). High performance liquid chromatography (HPLC) was performed with an Agilent pump equipped with an Agilent-1200 with refractive index (RI) detector (Santa Clara, CA, USA) and a semi-preparative reversed-phase column (Econosphere™, RP-C18, 5 μm, 250 × 4.6 mm, Alltech, Deerfield, IL, USA). Silica gel 60 (230–400 mesh, Merck, Darmstadt, Germany) was used for column chromatography; reversed-phase silica gel for column chromatography, Chromatorex ODS DM1020T (Fuji Silysia Chemical, Ltd., 100–200 mesh. Pre-coated silica gel plates (Kieselgel 60 F254, 0.25 mm, Merck, Darmstadt, Germany) were used for TLC analyses. Spots were visualized by heating after spraying with 10% H_2_SO_4_.

### 3.2. Plant Material 

*Euphorbia sanctae-catharinae* plants were collected in May 2013 from Wadi Jibaal in St Katherine Protectorate, south Sinai, Egypt in the flowering stage. A voucher specimen (#212) has been deposited in the herbarium of the National Research Centre. The collection was taking place under the permission of St Katherine Protectorate for scientific purposes. The plant was kindly authenticated by Dr. Mona Marzouk, Associate Professor of Taxonomy, National Research Center, Cairo, Egypt.

### 3.3. Extraction and Isolation

Aerial parts (2.0 kg) were powdered and extracted with CH_2_Cl_2_:MeOH (1:1) at room temperature. The extract was concentrated in vacuo to obtain a gummy residue (110 g). The concentrated crude extract (110 g) was fractionated on silica gel flash CC (5 × 60 cm) and eluted with gradient solvents of increasing polarity starting with (100%) *n*-hexane followed by a gradient of *n*-hexane/ethyl acetate up to 100 % ethyl acetate. Seventeen fractions were collected and pooled together according to the TLC profile (using solvent systems: S1: n-hexane:EtOAc (4:1 *v*/*v*), S2: methylene chloride:methanol (7:0.5 *v*/*v*), S3: n-hexane:EtOAc (1:1 *v*/*v*)). Vanillin-sulphuric acid spray reagent was used as spray reagents for spots dete ction on the chromatograms. The chromatograms were visualized in visible and under UV light (at 254 nm and 365 nm). Similar fractions were combined according to their chromatographic patterns to yield nine collected fractions. These fractions were then subjected for chemical investigation. The nine different subfractions that obtained were A (10 gm), B (8 gm), C (8.5 gm), D (6 gm), E (10 gm), F (12 gm), G (11 gm), H (9 gm), I (20 gm). From TLC profiles, fractions that appeared to contain mainly fatty acids and low levels of terpenes and flavonoids were not advanced for additional HPLC purification. Fraction D (6 gm) as subjected to further fractionation on ODS column (3 × 60 cm) using 85% MeOH: 15% H_2_O and finally wash with 100% MeOH. The obtained fraction was subjected to isolation and purification by a reversed phase HPLC (20 × 250 cm) using MeOH:H_2_O (85%:15%, 2.5 L) to afford nine compounds (**1**, 20 mg), (**2**, 8.5 mg), (**3**, 7.0 mg), (**4**, 6.5 mg), (**5**, 14 mg), (**6**, 11 mg), (**7**, 8 mg), (**8**, 9 mg) and (**9**, 6 mg). Fraction F (12 gm) was also subjected to further fractionation on ODS column (3 × 60 cm) using (85% MeOH: 15% H_2_O) and finally washed with 100% MeOH. The obtained fraction was further purified by a reversed phase HPLC using MeOH: H_2_O (1:1, 2.5 L) to afford one compound (**10**, 6.5 mg). Fraction G (11 gm) was also subjected for isolation and purification by a reversed phase HPLC using MeOH: H_2_O (1:1, 2.5 L) to afford one compound (**11**, 7 mg). Fraction H (9 mg) was subjected for isolation and purification by a reversed phase HPLC using MeOH/H_2_O (50%/50%, 2.5 L) to afford two compounds (**12**, 8.5 mg) and (**13**, 6 mg).

Premyrsinol-3-propanoate-5(α-3methyl)butyrate-7,13,17-triacetate (euphosantianane A, **1**): colorless oil; ${\left[\alpha \right]}_{D}^{25}$ + 20.0 (c 0.01, MeOH); FT-IR (KBr) v_max_: 3532, 1741,1450 and 716 cm^−1^; ^1^H- and ^13^C-NMR data, see Table S1; HRFABMS *m*/*z* 673.3203(M + Na); (calcd. for C_20_H_30_O_2_Na, 673.3200).

Premyrsinol-3-propanoate-5-isobutyrate-7,13diacetate-17-benzoate (euphosantianane B, **2**): white powder; ${\left[\alpha \right]}_{D}^{25}$ + 27.0 (c 0.01, MeOH)); FT-IR (KBr) v_max_: 3532, 1741, 1450 and 716 cm^−1^; ^1^H- and ^13^C-NMR data, see Table S1; HRFABMS *m*/*z* 721.3206 (M + Na); (calcd. for C_38_H_50_O_12_Na, 721.3200).

Premyrsinol-3-propanoate-5-benzoate-17-benzoyl (euphosantianane C, **3**): white powder; ${\left[\alpha \right]}_{D}^{25}$ + 64.0 (c 0.01, MeOH); FT-IR (KBr) v_max_: 3532, 1741, 1450 and 716 cm^−1^; ^1^H- and ^13^C-NMR data, see Table S1; HRFABMS *m*/*z* 755.3050 (M + Na); (calcd. for C_41_H_48_O_12_Na, 755.3043).

Premyrsinol-3-propanoate-5(α-2methyl)butyrate-7,13-diacetate-17-nicotinate (euphosantianane D, **4**): white powder; ${\left[\alpha \right]}_{D}^{25}$ + 30.4 (c 0.01, MeOH); FT-IR (KBr) v_max_: 3532, 1741,1450 and 716 cm^−1^; ^1^H- and ^13^C-NMR data, see Table S1; HRFABMS *m*/*z* 736.3312 (M + Na); (calcd. for C_38_H_51_O_12_NNa, 736.3309).

### 3.4. Biological Activity

#### 3.4.1. Cell Culture

All materials and reagents for the cell cultures were purchased from Lonza (Verviers, Belgium). Human colon cancer cell line Caco-2 and human lung cancer cell line A549 were maintained as monolayer culture in Dulbecco’s modified Eagle’s medium (DMEM) supplemented with 10% FBS, 4 mM glutamine, 100 U/mL penicillin, and 100 µg/mL streptomycin sulfate. Monolayers were passaged at 70–90% confluence using trypsin-EDTA solution. All cell incubations were maintained at humidified CO_2_ incubator with 5% CO_2_ at 37 °C.

#### 3.4.2. Cell Proliferation Assay

Anti-proliferative studies were performed using a modified MTT (3-[4,5-dimethylthiazole-2-yl]-2,5-diphenyltetrazolium bromide) assay based on a previously published method [35,36]. Appropriate cell densities of exponentially growing A549 and Caco-2 cells (5000 cells/well) were seeded onto 96-well plates. After a 24 h incubation period with 5% CO_2_ at 37 °C, stock test compounds (**1**–**13**) dissolved in dimethyl sulfoxide (DMSO) were added at concentrations of 100, 50, 25, 12.5, and 6.25 µM in culture medium (final DMSO concentration in medium = 0.1%, by volume). After 48 h of incubation, MTT solution in PBS (5 mg/mL) was added to each well, after which the incubation was resumed for a further 90 min [37,38]. The formation of intracellular formazan crystals (mitochondrial reduction product of MTT) was confirmed by a phase contrast microscopic examination. Photomicrographs were taken using an inverted microscope (Ziess, Germany) with attached eye-piece digital camera (Total magnification = 150×). At the end of the incubation period, the medium was removed and 100 µL of DMSO was added to each well to dissolve formed formazan crystals with shaking for 10 min (200 rpm). Dissolved crystals were quantified by reading the absorbance at 492 nm (OD) on a microplate reader (Sunrise™ microplate reader, Tecan Austria GmbH, Grödig, Austria) and were used as a measure of cell proliferation. 

#### 3.4.3. Anti-Proliferation Quantitative Analysis

Cell proliferation was determined by comparing the average OD values of the control wells with those of the samples (quadrate to octuplet treatments) both represented as % proliferation [control proliferation (0.1% DMSO) = 100%]. The IC_50_ values (concentration of sample causing 50% loss of cell proliferation of the vehicle control) were calculated using the concentration-response curve fit to the non-linear regression model using GraphPad Prism^®^ v6.0 software (GraphPad Software Inc., San Diego, CA, USA).

### 3.5. Computational Calculations

Conformational ensembles for **8** and **9** were generated with MMFF94S force field and an energy window of 10 kcal/mol using Omega2 software [39,40]. To avoid missing any possible conformers, all generated conformers were subjected to molecular dynamics (MD) simulation for 10 ns in methanol with AMBER14 software (University of California, San Francisco, CA, USA) [41]. Uncorrelated conformations were then collected every 10 ps over the 10 ns MD simulation time and minimized using the truncated Newton linear conjugate gradient method with LBFGS preconditioning implemented in AMBER14 software [41]. All unique conformations, in terms of energy, were then geometrically optimized at the B3LYP/6-31G* level of theory using Gaussian09 (Gaussian, Inc., Wallingford CT, USA) [42]. A vibrational frequency calculation was performed to confirm the minimum energy state of the optimized conformers as well as to calculate the corresponding free energies. TDDFT calculations were carried out at the B3LYP/6-31G* level of theory and the first 50 excitation states were calculated. To consider the solvent effect in optimization and TDDFT calculations, a polarizable continuum model (PCM) using methanol as a solvent was incorporated. ECD spectra were then generated using the SpecDis 1.71 (Berlin, Germany) [43,44] by applying Gaussian band shapes with sigma = 0.25 ev. The theoretical ECD spectrum was generated by averaging the ECD spectra of each conformer using Boltzmann statistics. Wavelength shift and intensity scaling were applied in the computational/experimental comparison. 

## Figures and Tables

**Table 1:** ^1^H-NMR and ^13^C-NMR spectral data of compounds **1**–**5** (600 MHz, δ-ppm).

No.	1		2		3		4	
	*d*_H_ (*J* in Hz)	δ_C_	*d*_H_ (*J* in Hz)	δ_C_	*d*_H_ (*J* in Hz)	δ_C_	*d*_H_ (*J* in Hz)	δ_C_
1α	3.12 dd (8.4, 13.8)	42.9	3.14 dd (8.4, 13.8)	42.9	3.15 dd (7.2, 13.8)	42.9	3.14 dd (7.8, 13.2)	42.9
1β	1.59 dd (12.6, 13.2)		1.60 dd (13.2)		1.61 dd (13.8, 13.2)		1.61 dd	
2	1.80 m	37.5	1.86 m	37.5	1.87 m	37.3	1.79 m	37.5
3	5.24 dd (3.6, 6.0)	78.4	5.23 t	78.4	5.36 t (3.6)	78.3	5.21 t	77.3
4	2.32 m	50.4	2.32 m	50.5	2.34 m	50.4	2.36 dd (3.0)	50.6
5	6.18 d (11.4)	68.8	6.21 d (12.0)	69.1	6.46 d (11.4)	69.9	6.23 d (12.0)	69
6	----	47.4	----	47.8	----	48.2	----	47.7
7	4.48 d (6.6)	70.7	4.72 d (6.6)	71	4.97 d (6.6)	70.7	4.85 d (12.6)	70.8
8α	2.09 m	23.9	2.14 m	21.4	2.05 m	22.2	2.09 m	23.9
8β	1.80 brd (17.0)	23.9	1.77 brd (17.0)	21.4	1.87 brd (17.0)	22.2	1.90 d (13.2)	23.9
9	0.72 m	18.9	0.77 m	18.4	0.72 m	19.1	0.62 m	19
10	----	18.2	----	18.3	----	18.4	----	18.3
11	0.72 m	21.4	0.77 m	18.5	0.72 m	21.5	0.62 m	21.3
12	3.37 d (6)	34.8	3.46 d (5.4)	33.9	3.55 d (6.6)	35.3	3.46 d (3.6)	35
13	----	86.0	----	85.9	----	85.9	----	85.8
14	----	204.5	----	204.5	----	204.4	----	204.3
15-OH	4.44 s	84.1	4.44 s	84.1	4.45 s	84.2	4.48 s	84.1
16	0.87 d (6.0)	14.1	0.86 d (1.8)	14.2	0.86 d (6.0)	14	0.87 d (14.4)	14.7
17α	4.39 d (12.0)	63.6	4.81 d (12.0)	64	4.58 d (11.4)	63.4	4.67 (d, *J* = 11.4 Hz)	64.5
17β	4.31 d (12.0)		4.46 d (12.0)		4.91 d (10.8)		4.46 brd (11.4)	
18	1.04 s	29.5	1.05 s	29.5	1.06 s	29.5	1.05 s	29.5
19	0.90 s	14.9	0.94 s	14.9	0.95 s	15	0.93 s	14.9
20	1.68 s	24.6	1.73 s	24.6	1.66 s	25	1.71 s	25.8

^1^H-NMR of other signals (δ), for **1**: O-Prop: 2.31 (q, *J* = 7.0 Hz), 1.08 (t, *J* = 7.0 Hz); O-3MeBu, 1.97 m, 2.32 m, 0. 90 (d, *J* = 7.8 Hz), 0.92 (d, *J* = 7.8 Hz); OAc-7, 2.08 (s); OAc-13, 2.09 (s); OAc-17, 2.06 (s). For **2**: O-Prop: 2.30 (q, *J* = 8.4 Hz), 1.08 (t, *J* = 8.4 Hz); O-*i*Bu, 2.39 m, 1.07 (d, *J* = 7.0 Hz), 1.09 (d, *J* = 7.0 Hz); OBz, 7.91 (AA′), 7.58 (C), 7.47; OAc-7, 2.14 (s); OAc-13, 2.15 (s). For **3**: O-Prop: 1.08 (t, *J* = 7 Hz), 2.45 (q, *J* = 7 Hz); OBz, 7.70 (brd, *J* = 7.2 Hz), 7.52 (brdd, *J* = 7.2 Hz), 7.33 m, 7.11 (m), 7.00 (brt, *J* = 7.2); OAc-7, 2.12 (s); OAc-13, 2.17 (s). For **4**: O-Prop: 1.08 (t, *J* = 7.8 Hz),2.25 (q, *J* = 9.0 Hz); O-MeBu, 2.14 m, 1.29 m, 1.06 (d, *J* = 7.8 Hz), 1.07 (t, *J* = 7.8 Hz); O-Nic, 7.43 (dd, *J* = 4.8, 7.8 Hz), 8.18 (t, *J* = 7.8 Hz), 8.80 (br d, *J* = 7.8 Hz), 9.14 br s; OAc-7, 2.10 (s); OAc-13, 2.05 (s). ^13^C-NMR other signals (δ), for **1**: O-Prop: 8.9, 27.8; O-3MeBu, 21.4, 21.4, 26.5, 42.8; OAc-7, 170.0; OAc-13, 170.7; OAc-17, 170.4; C=O (prop, 174.2); C=O (3-MeBu, 174.3). For **2**: O-Prop: 8.9, 27.7; O-*i*Bu, 34.9, 18.6, 19.0; OBz, 128.9, 129.4, 133.6, 128.9, 129.4, 130.1; OAc-7, 170.0; OAc-13, 170.0; C=O (prop, 170.7); C=O (iBu, 174.1). For **3**: O-Prop: 8.8,27.6; OBz-17, 132.9, 129.2, 127.9, 132.7, 127.9, 129.2, 129.4; OBz-5, 129.4, 129.2, 127.9, 129.6, 127.9, 129.2; OAc-7, 170.2; OAc-13, 170.2; C=O (prop, 170.8); C=O (OBz-5, 165.3); C=O (OBz-17, 173.6). For **5**: O-Prop: 8.9, 27.8; O-MeBu, 11.6, 40.8, 14.9, 26.0; O-Nic, 153.9, 150.6, 136.9, 125.8, 123.7; OAc-7, 170.0; OAc-13, 170.7.

**Table 2:** IC_50_ values for **1**–**13** against proliferation of human Caco-2 and A549 tumor cell lines.

Compound	IC_50_ on Caco-2 (µM) ^a^	IC_50_ on A549 (µM) ^a^
**1**	75.8 (0.950)	>100
**2**	40.5 (0.989)	48.5 (0.927)
**3**	31.0 (0.999)	21.5 (0.924)
**4**	33.2 (0.993)	32.8 (0.988)
**5**	43.5 (0.999)	50.1 (0.9960)
**6**	33.3 (0.984)	33.1 (0.983)
**7**	40.3 (0.979)	60.3 (0.937)
**8**	26.1 (0.979)	31.3 (0.971)
**9**	29.4 (0.972)	3.3 (0.996)
**10**	43.9 (0.975)	>100
**11**	50.2 (0.993)	>100
**12**	44.7 (0.961)	>100
**13**	79.4 (0.843)	>100
Doxorubicin HCl	0.7 (0.999)	0.4 (0.987)

^a^ Goodness of fit values (R^2^) given in parentheses based on non-linear regression using GraphPad prism V 6.0 software (GraphPad Software Inc., San Diego, CA, USA).

## Supplementary Materials

The supplementary materials are available online at http://www.mdpi.com/1420-3049/23/9/2221/s1.
