# Supplementary material for: Euphosantianane A–D: Antiproliferative Premyrsinane Diterpenoids from the Endemic Egyptian Plant Euphorbia Sanctae-Catharinae
Source: Molecules. 2018 Sep 1;23(9):2221. doi: 10.3390/molecules23092221 (PMC6225227; doi:10.3390/molecules23092221)

Euphosantianane A-D: AntiproliferativePremyrsinaneDiterpenoids from the Endemic Egyptian Plant *Euphorbia sanctae-catharinae*

Mohamed Elamir F. Hegazy[a,b], Ahmed R. Hamed[a,c], Mahmoud A. A. Ibrahim[d], Zienab Talat[e], Eman H. Reda[e], Nahla S. Abdel-Azim[a], Fayza M. Hammouda[a], Seikou Nakamura[f], Hisashi Matsuda[f], Eman G. Haggag[g], Paul W. Paré[h,*], Thomas Efferth[b]

[1] Chemistry of Medicinal Plants Department, National Research Centre, 33 El-Bohouth St., Dokki, Giza 12622, Egypt.

[2] Department of Pharmaceutical Biology, Institute of Pharmacy and Biochemistry, University of Mainz, Staudinger Weg 5, 55128 Mainz, Germany

[3] Biology Unit, Central Laboratory for Pharmaceutical and Drug Industries Research Division, National Research Centre, 33 El-Bohouth St., Dokki, Giza 12622, Egypt.

[4] Computational Chemistry Laboratory, Chemistry Department, Faculty of Science, Minia University, Minia 61519, Egypt.

[5] Phytochemistry Lab., National Organization for Drug Control and Research, Giza, Egypt

[6] Kyoto Pharmaceutical University, Misasagi, Yamashina-ku, Kyoto 607-8412, Japan

[7] Helwan University, Department of Pharmacognosy, Cairo, Egypt.

[8] Department of Chemistry and Biochemistry, Texas Tech University, Lubbock, TX 79409, USA

* Correspondence: paul.pare@ttu.edu; +1-806-834-0461; Fax: +1-806-742-1289.

Content

**Supplementary**	**Pages**
S1. ^{1}H NMR (600 MHz, $CDCl_3$) spectrum of **1**	**11**
S2. ^{13}C NMR (150 MHz, $CDCl_3$) spectrum of **1**	**13**
S3. DEPT (150 MHz, $CDCl_3$) spectra of **1**	**17**
S4. ^{1}H ^{1}H COSY spectrum of **1** in $CDCl_3$	**18**
S5. HMBC spectrum of **1** in $CDCl_3$	**19**
S6. HSQC spectrum of **1** in $CDCl_3$	**22**
S7. NOESY experiment of **1** in $CDCl_3$	**23**
S8. LR-EI-MS of **1**	**24**
S9. HR-EI-MS of **1**	**25**
S10. ^{1}H NMR (600 MHz, $CDCl_3$) spectrum of **2**	**26**
S11. ^{13}C NMR (150 MHz, $CDCl_3$) spectrum of **2**	**28**

S12. DEPT (150 MHz, $CDCl_3$) spectra of **2** **30**

S13. ^1H ^1H COSY spectrum of **2** in $CDCl_3$ **31**

S14. HMBC spectrum of **2** in $CDCl_3$ **33**

S15. HSQC spectrum of **2** in $CDCl_3$ **36**

S16. NOESY experiment of **2** in $CDCl_3$ **39**

S17. LR-EI-MS of **2** **40**

S18. HR-EI-MS of **2** **41**

S19. ^1H NMR (600 MHz, $CDCl_3$) spectrum of **3** **42**

S20. ^{13}C NMR (150 MHz, $CDCl_3$) spectrum of **3** **44**

S21. DEPT (150 MHz, $CDCl_3$) spectra of **3** **45**

S22. ^1H ^1H COSY spectrum of **3** in $CDCl_3$ **46**

S23. HMBC spectrum of **3** in $CDCl_3$ **47**

S24. HSQC spectrum of **3** in $CDCl_3$ **49**

S25. NOESY experiment of **3** in $CDCl_3$ **50**

S26. LR-EI-MS of **3** **51**

S27. HR-EI-MS of **3** **52**

S28. ^{1}H NMR (600 MHz, $CDCl_3$) spectrum of **4** **53**

S29. ^{13}C NMR (150 MHz, $CDCl_3$) spectrum of **4** **55**

S30. DEPT (150 MHz, $CDCl_3$) spectra of **4** **57**

S31. ^{1}H ^{1}H COSY spectrum of **4** in $CDCl_3$ **58**

S32. HMBC spectrum of **4** in $CDCl_3$ **59**

S33. HSQC spectrum of **4** in $CDCl_3$ **61**

S34. NOESY experiment of **4** in $CDCl_3$ **63**

S35. LR-EI-MS of **4** **64**

S36. HR-EI-MS of **65**

S37. ^{1}H NMR (600 MHz, $CDCl_3$) spectrum of **5** **66**

S38. ^{13}C NMR (150 MHz, $CDCl_3$) spectrum of **5** **67**

S39. DEPT (150 MHz, $CDCl_3$) spectra of **5** **68**

S40. ^{1}H ^{1}H COSY spectrum of **5** in $CDCl_3$ **69**

S41. HMBC spectrum of **5** in $CDCl_3$ **71**

S42. HSQC spectrum of **5** in $CDCl_3$ **75**

S43. NOESY experiment of **5** in $CDCl_3$ **77**

S44. LR-EI-MS of **5** **78**

S45. HR-EI-MS of **5** **79**

S46. ^{1}H NMR (600 MHz, $CDCl_3$) spectrum of **6** **80**

S47. ^{13}C NMR (150 MHz, $CDCl_3$) spectrum of **6** **81**

S48. DEPT (150 MHz, $CDCl_3$) spectra of **6** **83**

S49. ^{1}H ^{1}H COSY spectrum of **6** in $CDCl_3$ **84**

S50. HMBC spectrum of **6** in $CDCl_3$ **85**

S51. HSQC spectrum of **6** in $CDCl_3$ **87**

S52. ^{1}H NMR (600 MHz, $CDCl_3$) spectrum of **7** **88**

S53. ^{13}C NMR (150 MHz, $CDCl_3$) spectrum of **7** **89**

S54. LR-EI-MS of **7** **90**

S55. ^{1}H NMR (600 MHz, $CDCl_3$) spectrum of **8** **91**

S56. ^{13}C NMR (150 MHz, $CDCl_3$) spectrum of **8** **92**

S57. LR-EI-MS of **8** **93**

S58. ^{1}H NMR (600 MHz, $CDCl_3$) spectrum of **9** **94**

S59. ^{13}C NMR (150 MHz, $CDCl_3$) spectrum of **9** **95**

S60. . ^{1}H ^{1}H COSY spectrum of **9** in $CDCl_3$ **97**

S61. HMBC spectrum of **9** in $CDCl_3$ **98**

S62. HSQC spectrum of **9** in $CDCl_3$ **99**

S63. NOESY experiment of **9** in $CDCl_3$ **100**

S64. LR-EI-MS of **9** **101**

S65. ^{1}H NMR (600 MHz, $CDCl_3$) spectrum of **10** **102**

S66. ^{13}C NMR (150 MHz, $CDCl_3$) spectrum of **10** **`103**

S67. ^{1}H NMR (600 MHz, $CDCl_3$) spectrum of **11** **104**

S68. ^{13}C NMR (150 MHz, $CDCl_3$) spectrum of **11** **105**

S69. ^{1}H NMR (600 MHz, $CDCl_3$) spectrum of **12** **106**

S70. ^{13}C NMR (150 MHz, $CDCl_3$) spectrum of **12** **107**

S71. ^{1}H NMR (600 MHz, $CDCl_3$) spectrum of **13** **108**

S72. ^{13}C NMR (150 MHz, $CDCl_3$) spectrum of **13** **109**

S73. Photomicrographs showing morphological changes of Caco-2 cells following 48 h exposure to compound 1 serial dilutions as mentioned at the *Experimental* section. Morphological signs of cytotoxicity include cell rounding, shrinking and loss of monolayer integrity compared to vehicle control. Total magnifictaion=150×. **110**

S74. Photomicrographs showing morphological changes of Caco-2 cells (A) or A549 cells (B) following 48 h exposure to compound 2 serial dilutions as mentioned at the *Experimental* section. Morphological signs of cytotoxicity include cell rounding, shrinking and loss of monolayer integrity compared to vehicle control. Total magnifictaion=150×. **111**

S75. Photomicrographs showing morphological changes of Caco-2 cells (A) or A549 cells (B) following 48 h exposure to compound 3 serial dilutions as mentioned at the *Experimental* section. Morphological signs of cytotoxicity include cell rounding, shrinking and loss of monolayer integrity compared to vehicle control. Total magnifictaion=150×. **112**

S76. Photomicrographs showing morphological changes of Caco-2 cells (A) or A549 cells (B) following 48 h exposure to compound 4 serial dilutions as mentioned at the *Experimental* section. Morphological signs of cytotoxicity include cell rounding, shrinking and loss of monolayer integrity compared to vehicle control. Total magnifictaion=150×. **113**

S77. Photomicrographs showing morphological changes of Caco-2 cells (A) or A549 cells (B) following 48 h exposure to compound 5 serial dilutions as mentioned at the *Experimental* section. Morphological signs of cytotoxicity include cell rounding, shrinking and loss of monolayer integrity compared to vehicle control. Total magnifictaion=150×. **114**

S78. Photomicrographs showing morphological changes of Caco-2 cells (A) or A549 cells (B) following 48 h exposure to compound 6 serial dilutions as mentioned at the *Experimental* section. Morphological signs of cytotoxicity include cell rounding, shrinking and loss of monolayer integrity compared to vehicle control. Total magnifictaion=150×. **115**

S79. Photomicrographs showing morphological changes of Caco-2 cells (A) or A549 cells (B) following 48 h exposure to compound 7 serial dilutions as mentioned at the *Experimental* section. Morphological signs of cytotoxicity include cell rounding, shrinking and loss of monolayer integrity compared to vehicle control. Total magnifictaion=150×. **116**

S80. Photomicrographs showing morphological changes of Caco-2 cells (A) or A549 cells (B) following 48 h exposure to compound 8 serial dilutions as mentioned at the *Experimental* section. Morphological signs of **117**

cytotoxicity include cell rounding, shrinking and loss of monolayer integrity compared to vehicle control. Total magnifictaion=150×.

S81. Photomicrographs showing morphological changes of Caco-2 cells (A) or A549 cells (B) following 48 h exposure to compound 9 serial dilutions as mentioned at the *Experimental* section. Morphological signs of cytotoxicity include cell rounding, shrinking and loss of monolayer integrity compared to vehicle control. Total magnifictaion=150×. **118**

S82. Photomicrographs showing morphological changes of Caco-2 cells (A) or A549 cells (B) following 48 h exposure to compound 10 serial dilutions as mentioned at the *Experimental* section. Morphological signs of cytotoxicity include cell rounding, shrinking and loss of monolayer integrity compared to vehicle control. Total magnifictaion=150×. **119**

S83. Photomicrographs showing morphological changes of Caco-2 cells following 48 h exposure to compound 11 serial dilutions as mentioned at the *Experimental* section. Morphological signs of cytotoxicity include cell rounding, shrinking and loss of monolayer integrity compared to vehicle control. Total magnifictaion=150×. **120**

S84. Photomicrographs showing morphological changes of Caco-2 cells following 48 h exposure to compound 12 serial dilutions as mentioned at the *Experimental* section. Morphological signs of cytotoxicity include cell rounding, shrinking and loss of monolayer integrity compared to vehicle control. Total magnifictaion=150×. **121**

S85. Photomicrographs showing morphological changes of Caco-2 cells following 48 h exposure to compound 13 serial dilutions as mentioned at the Materials and Methods section. Morphological signs of cytotoxicity include cell rounding, shrinking and loss of monolayer integrity compared to vehicle control. Total magnifictaion=150×. **122**

S86. Photomicrographs showing morphological changes of A549 cells following 48 h exposure to serial dilutions of doxorubicin HCl as mentioned at the Materials and Methods section. Morphological signs of cytotoxicity include cell rounding, shrinking and complete loss of monolayer integrity compared to vehicle control. Total magnifictaion=150×. **123**

S87. Photomicrographs showing morphological changes of Caco-2 cells following 48 h exposure to serial dilutions of doxorubicin HCl as mentioned at the Materials and Methods section. Morphological signs of cytotoxicity include cell rounding, shrinking and complete loss of monolayer integrity compared to vehicle control. Total magnifictaion=150×. **124**

S1. ^{1}H NMR (600 MHz, $CDCl_3$) spectrum of **1**

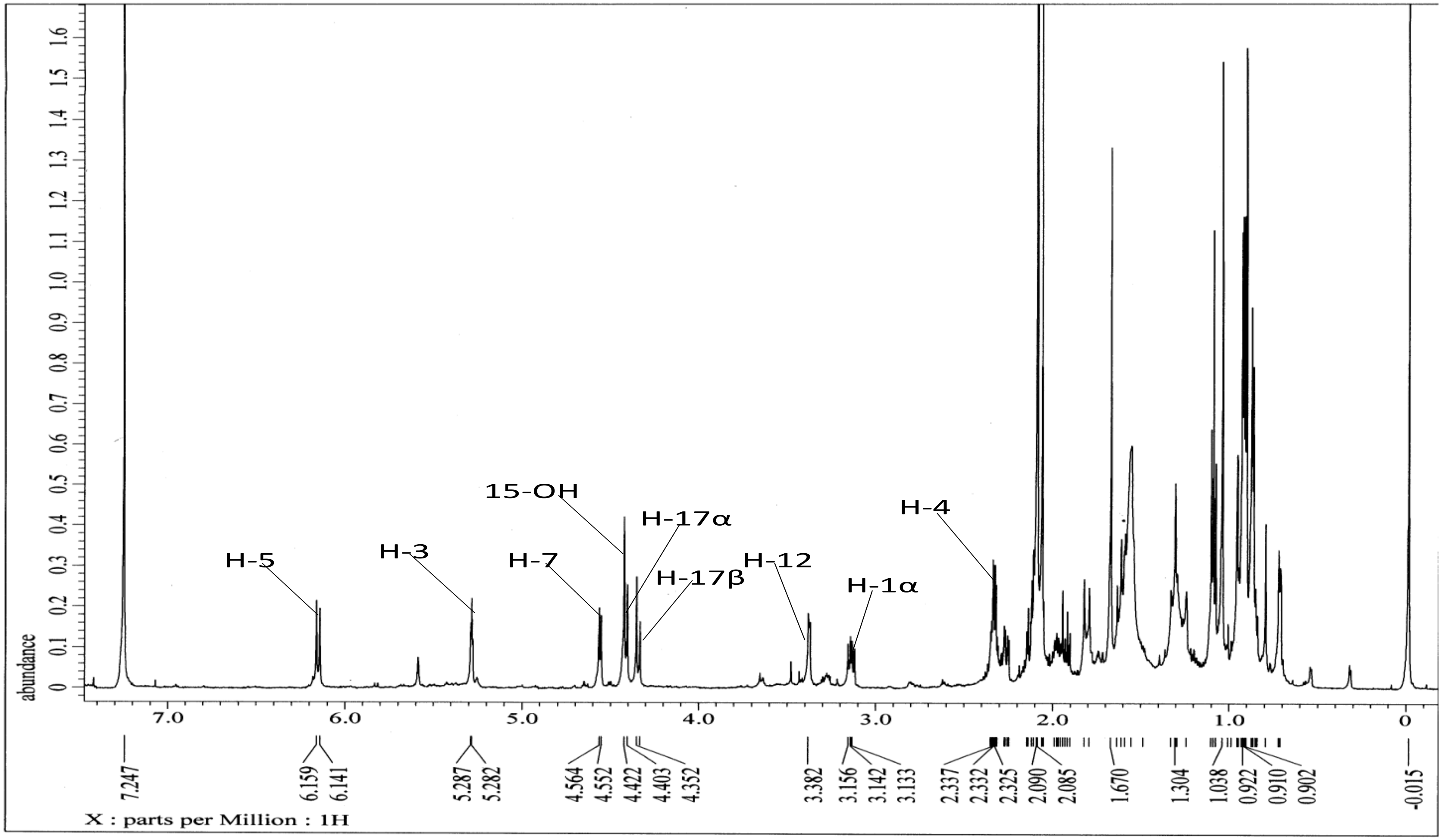

S1. ^{1}H NMR (600 MHz, $CDCl_3$) spectrum of **1**

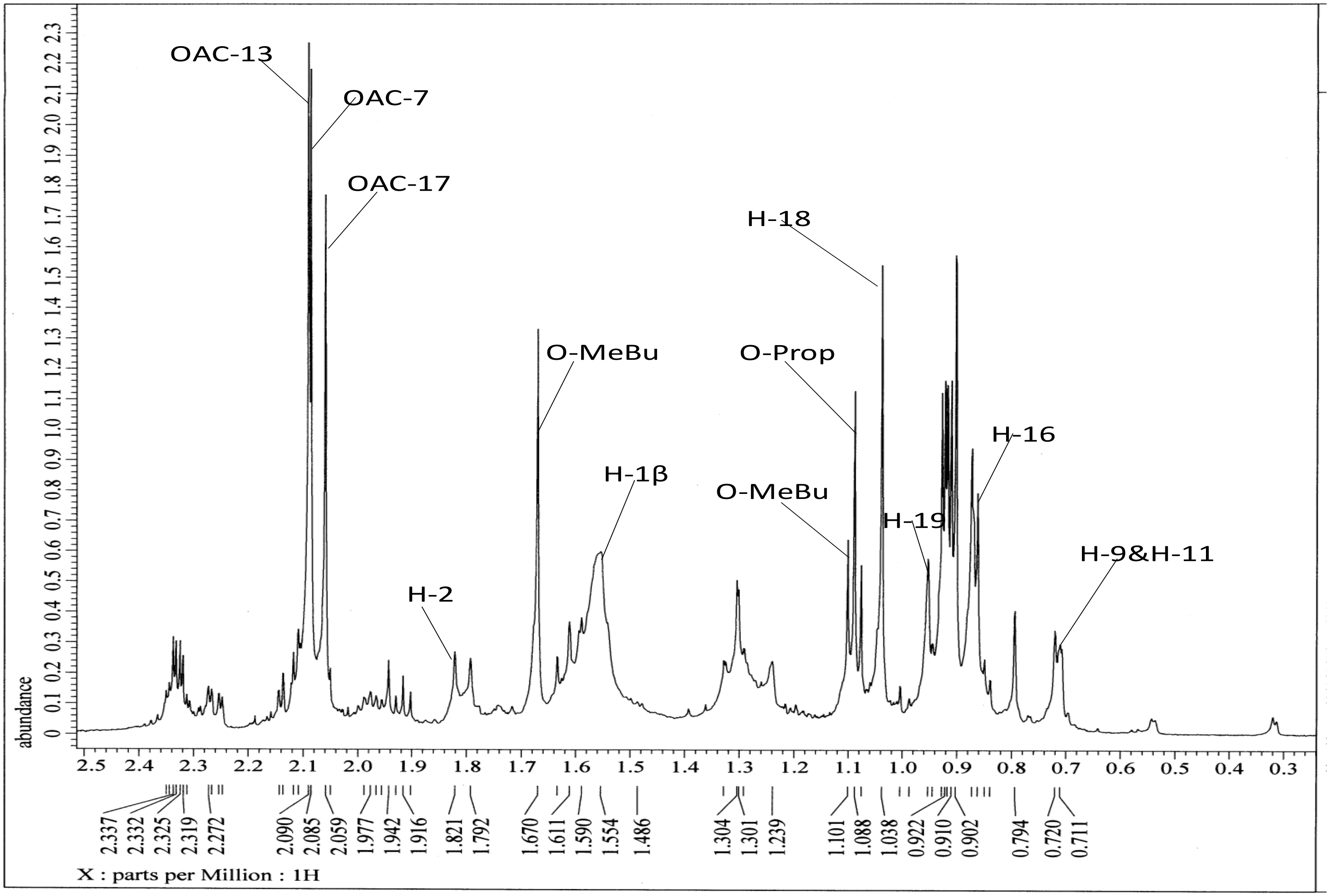

S2. ^{13}C NMR (150 MHz, $CDCl_3$) spectrum of **1**

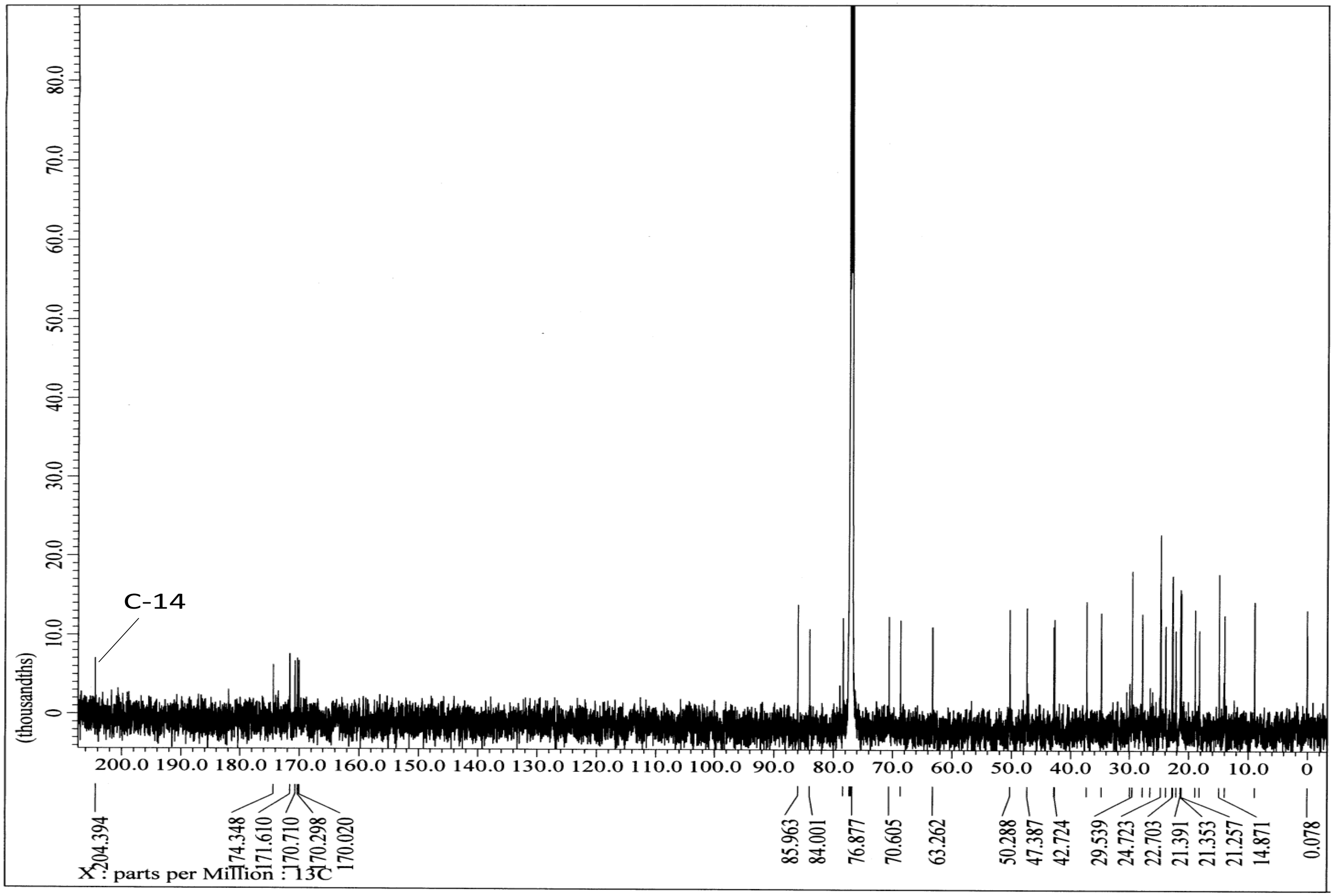

S2. ^{13}C NMR (150 MHz, $CDCl_3$) spectrum of **1**

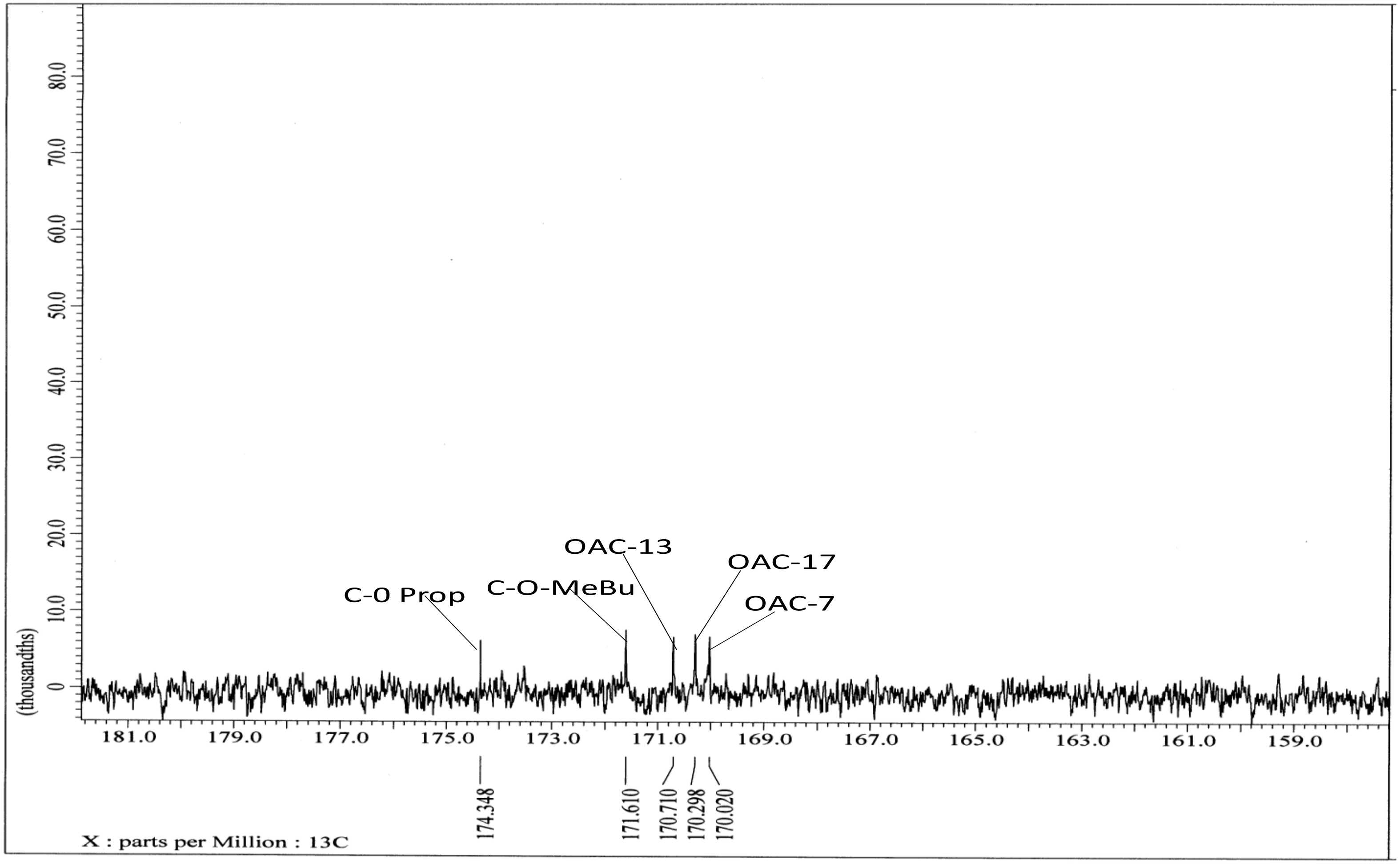

S2. ^{13}C NMR (150 MHz, $CDCl_3$) spectrum of **1**

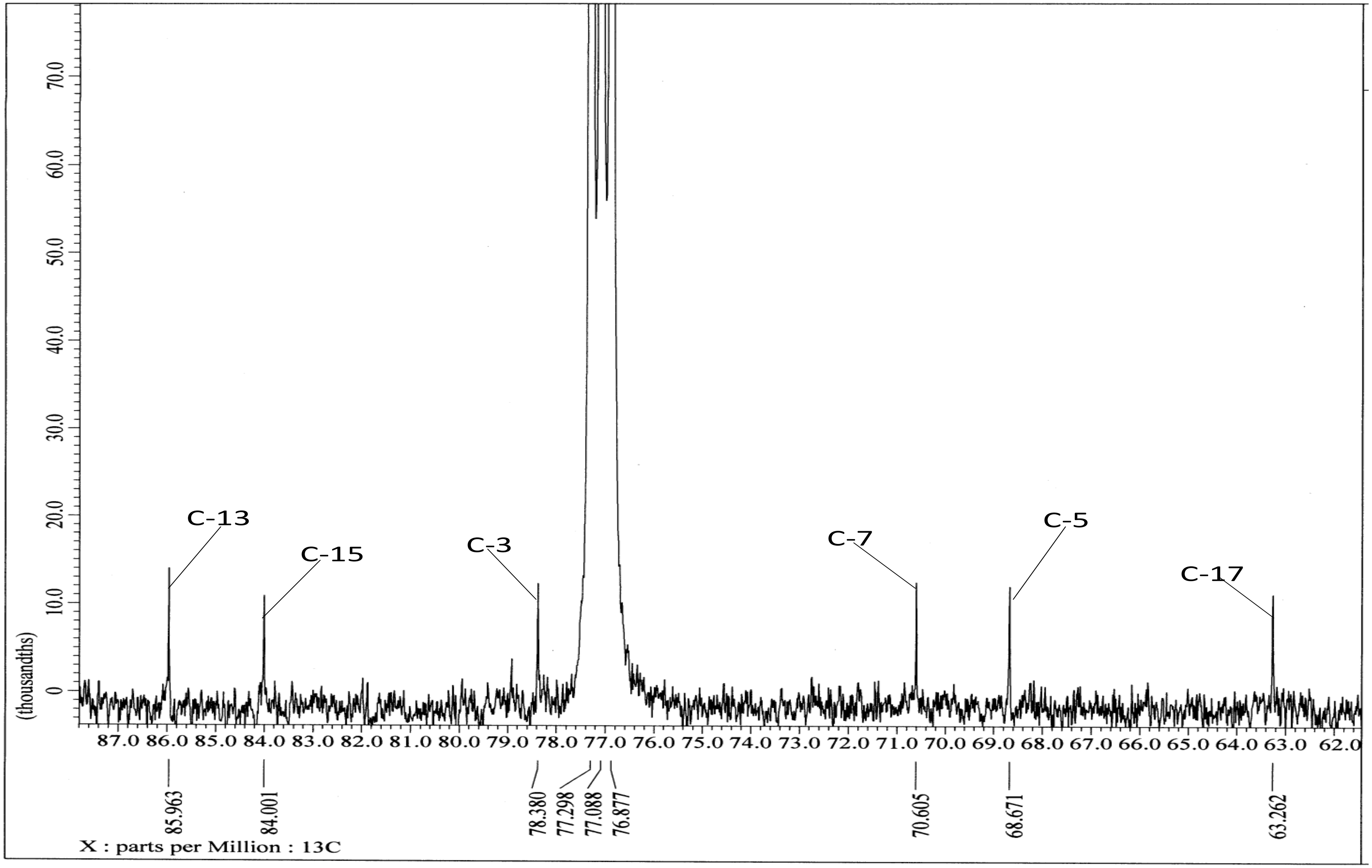

S2. ^{13}C NMR (150 MHz, $CDCl_3$) spectrum of **1**

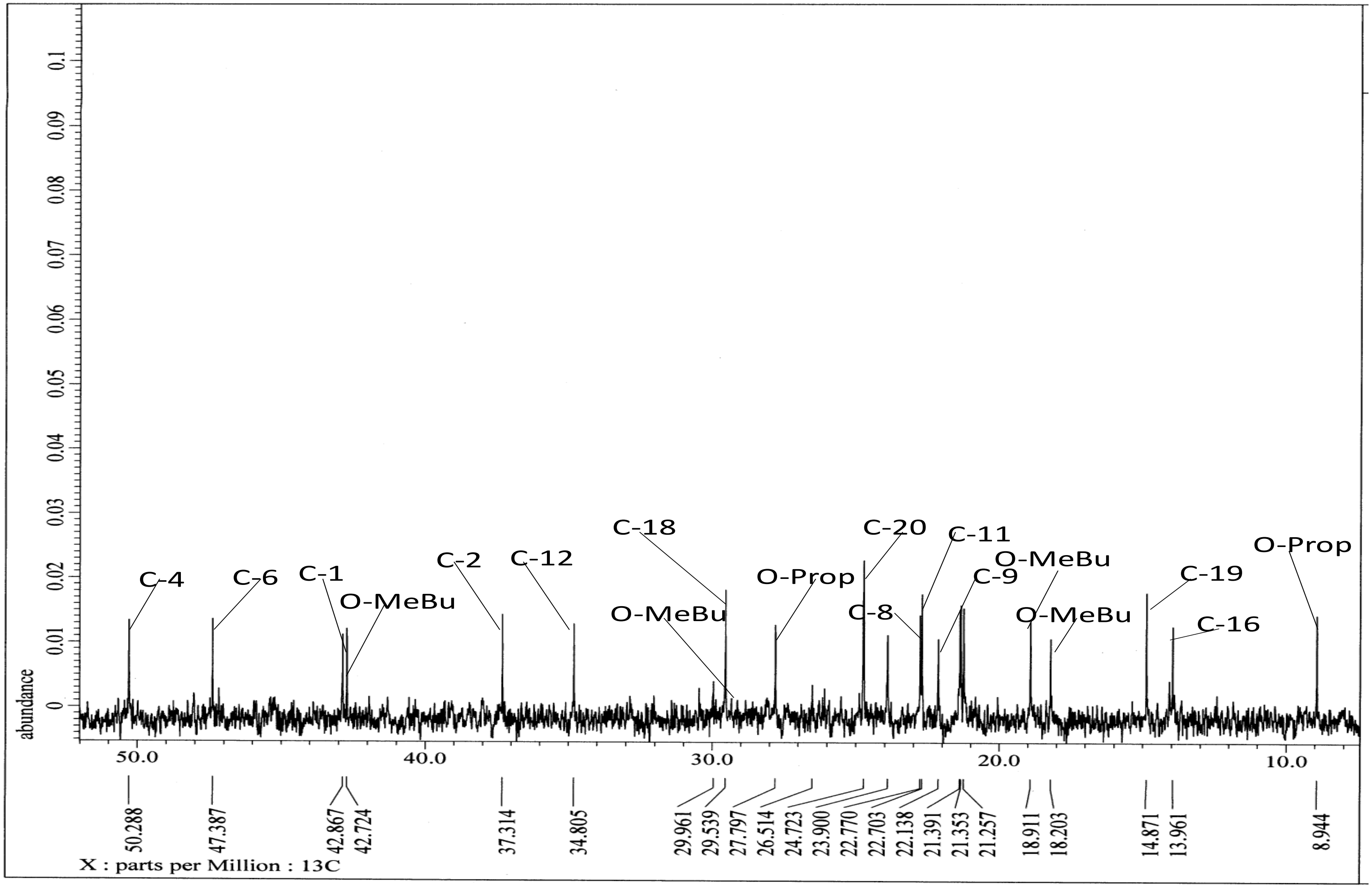

S3. DEPT (150 MHz, $CDCl_3$) spectra of **1**

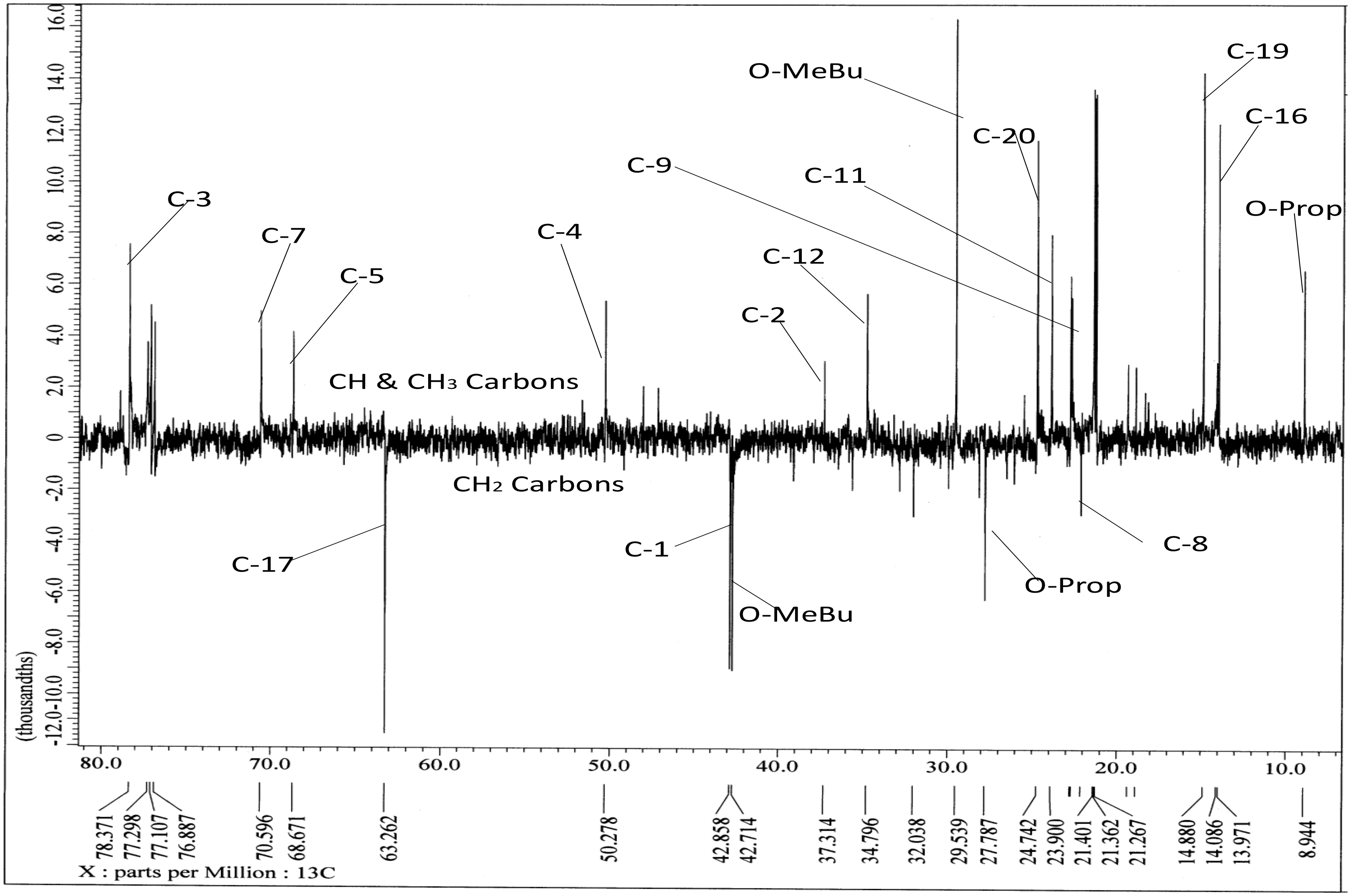

S4. ^{1}H ^{1}H COSY spectrum of **1** in $CDCl_3$

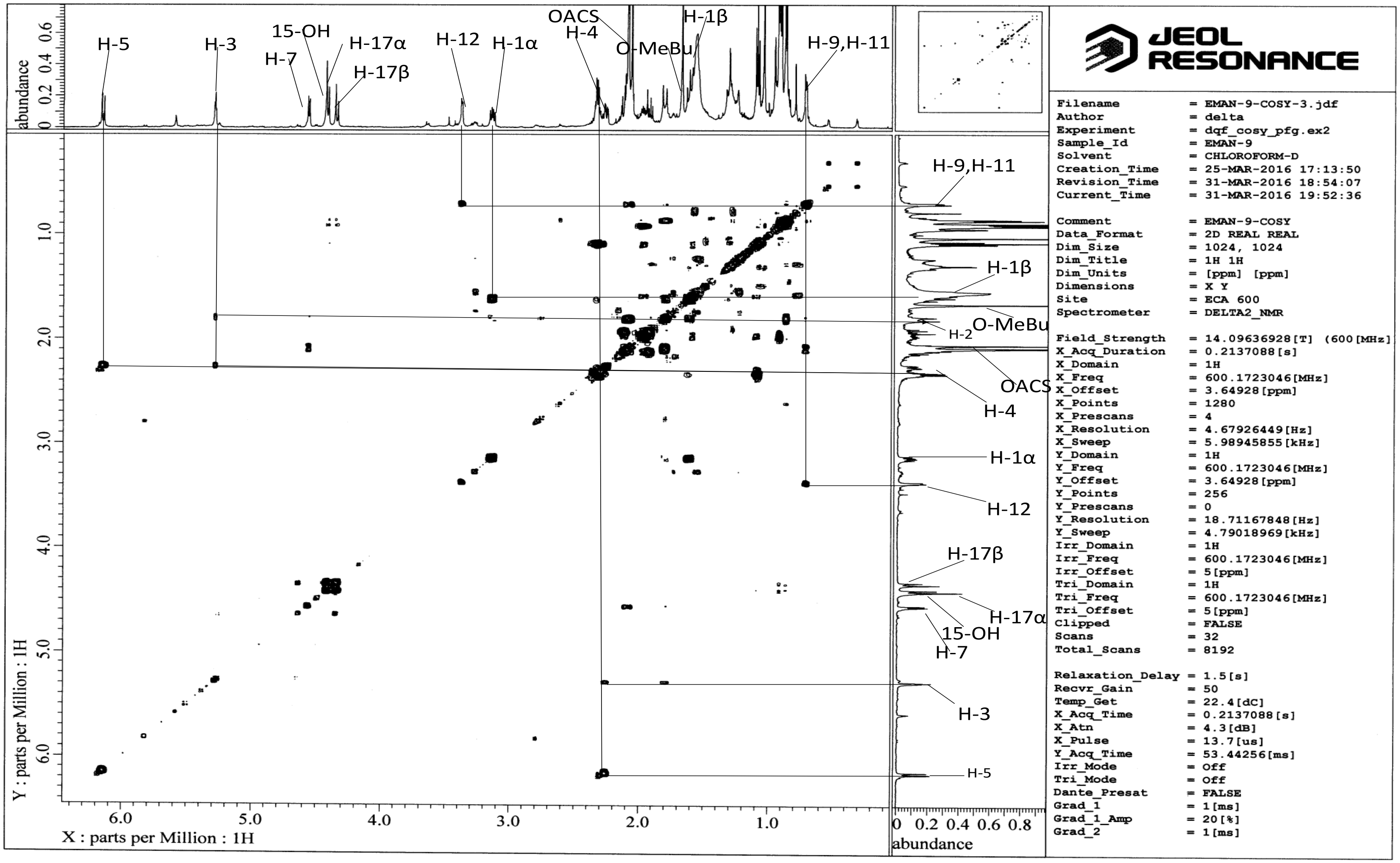

S5.. HMBC spectrum of **1** in $CDCl_3$

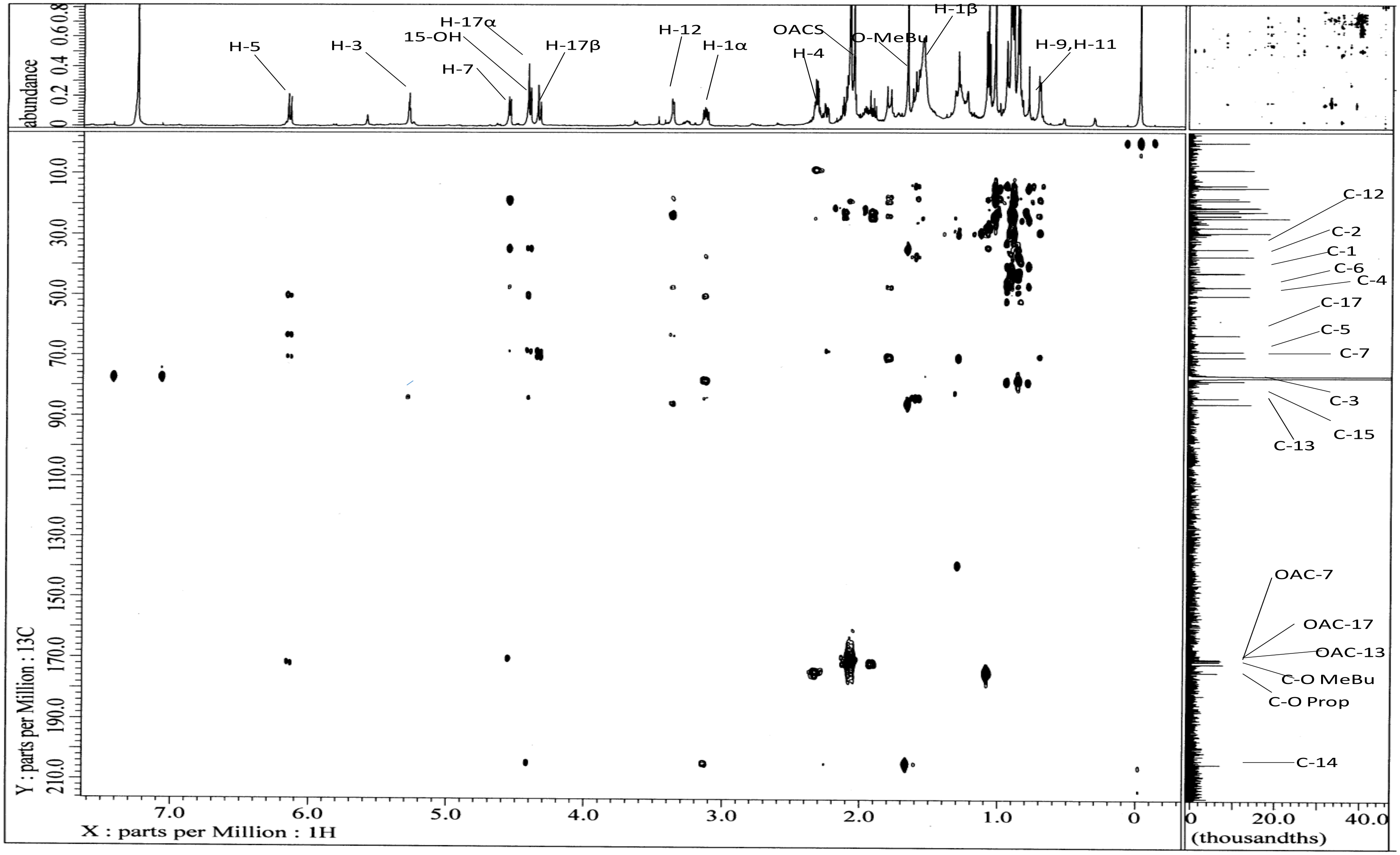

S5.. HMBC spectrum of **1** in $CDCl_3$

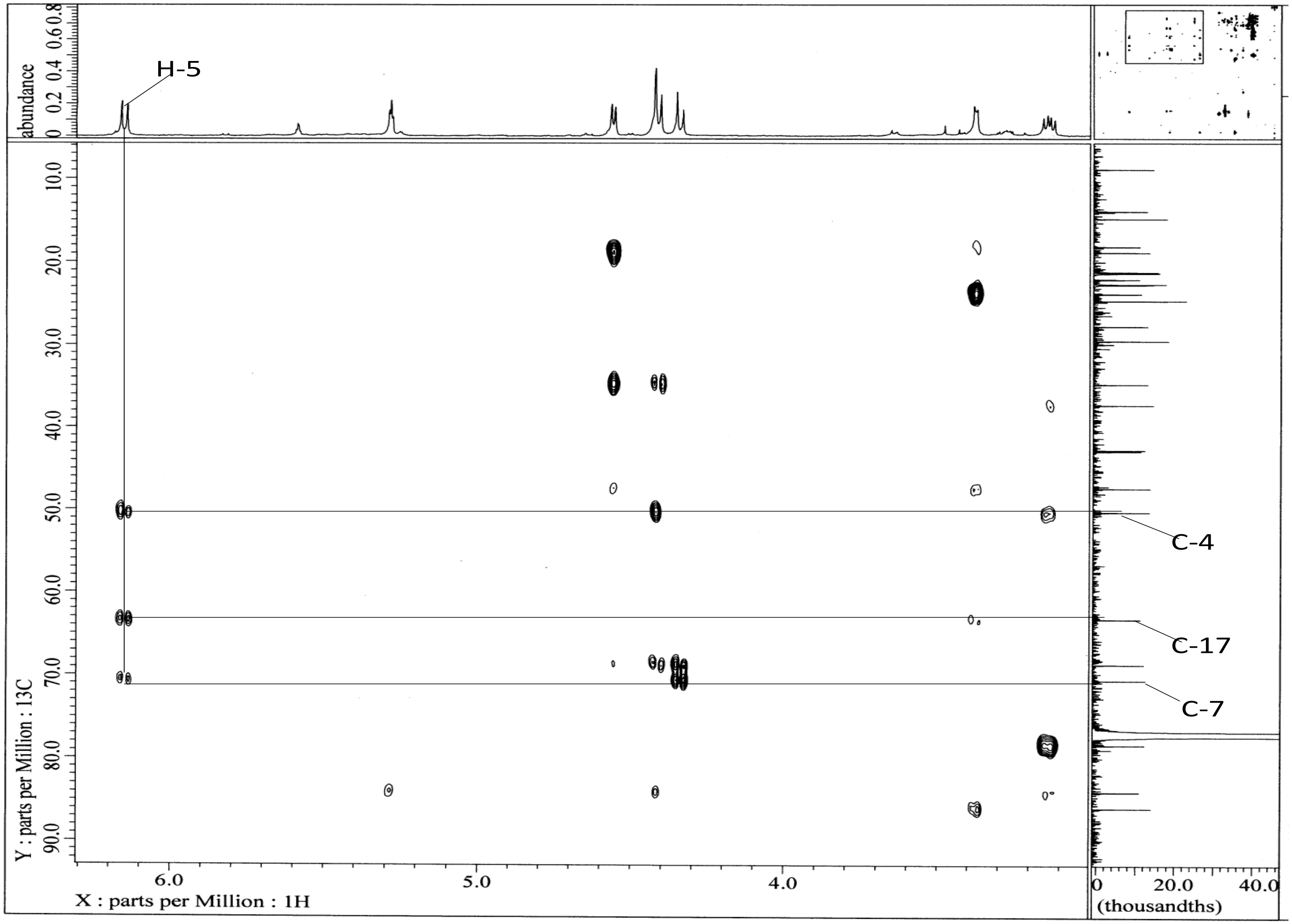

S5. HMBC spectrum of **1** in $CDCl_3$

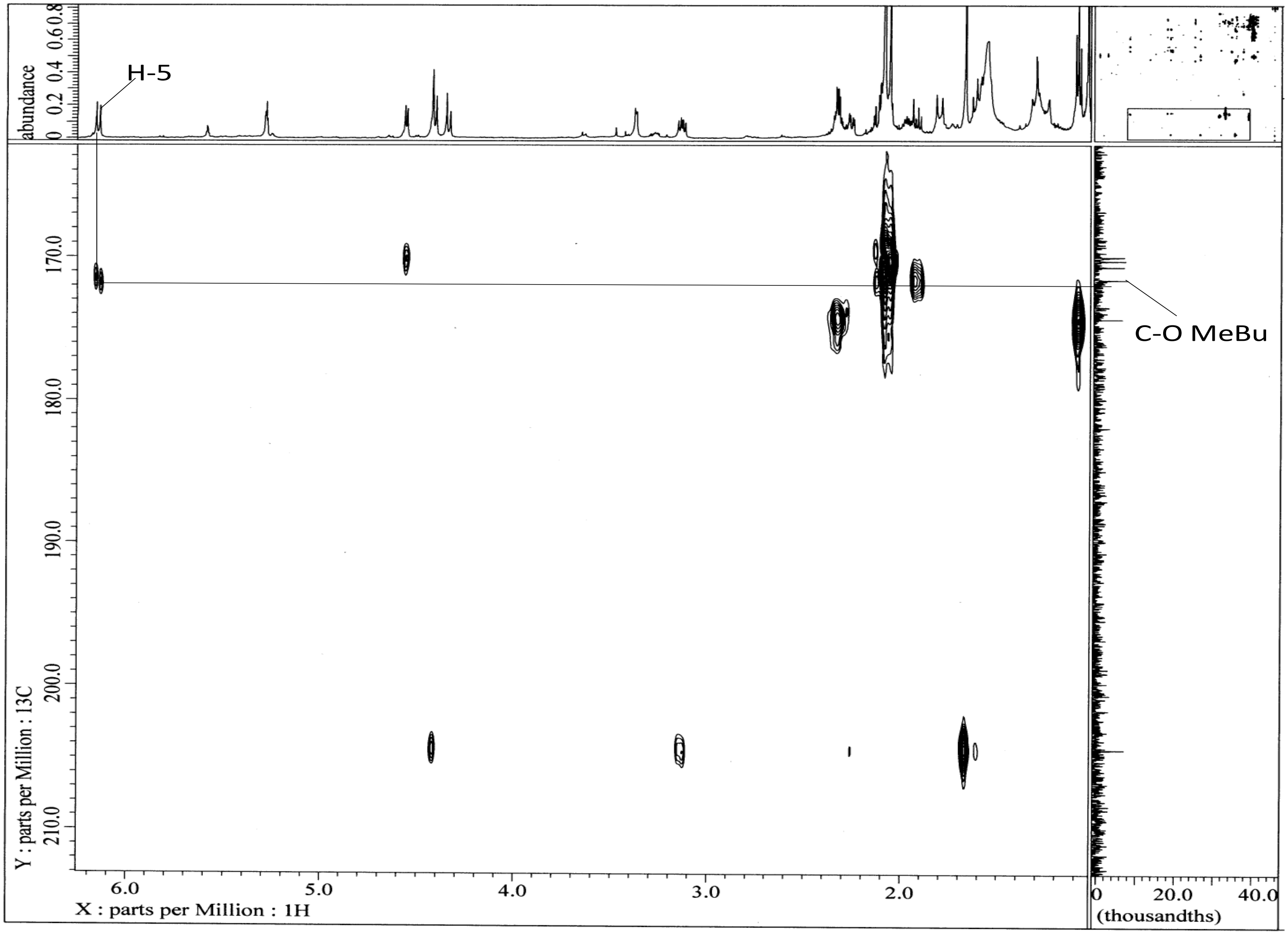

S6. HSQC spectrum of **1** in $CDCl_3$

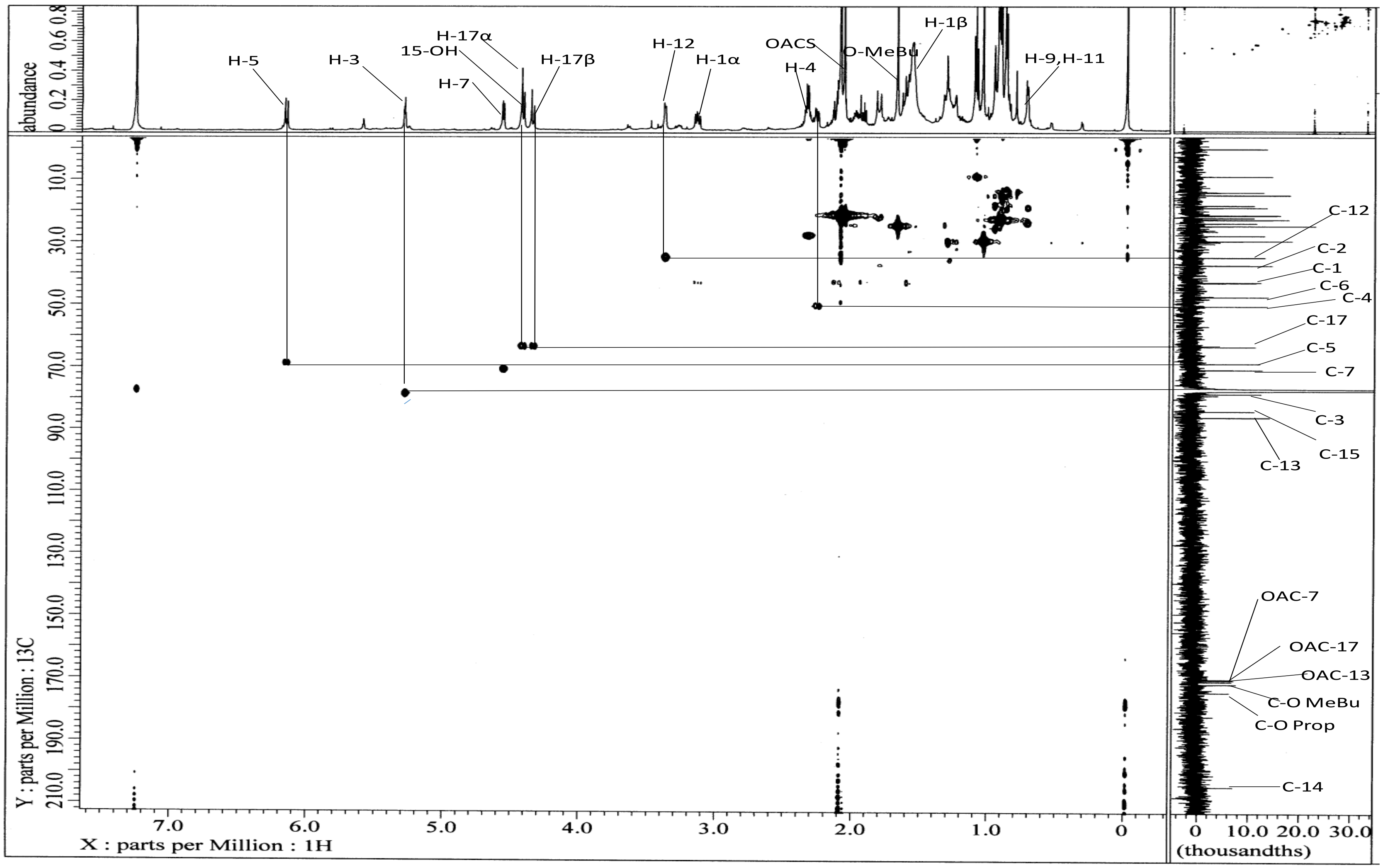

S7. NOESY spectrum of **1** in $CDCl_3$

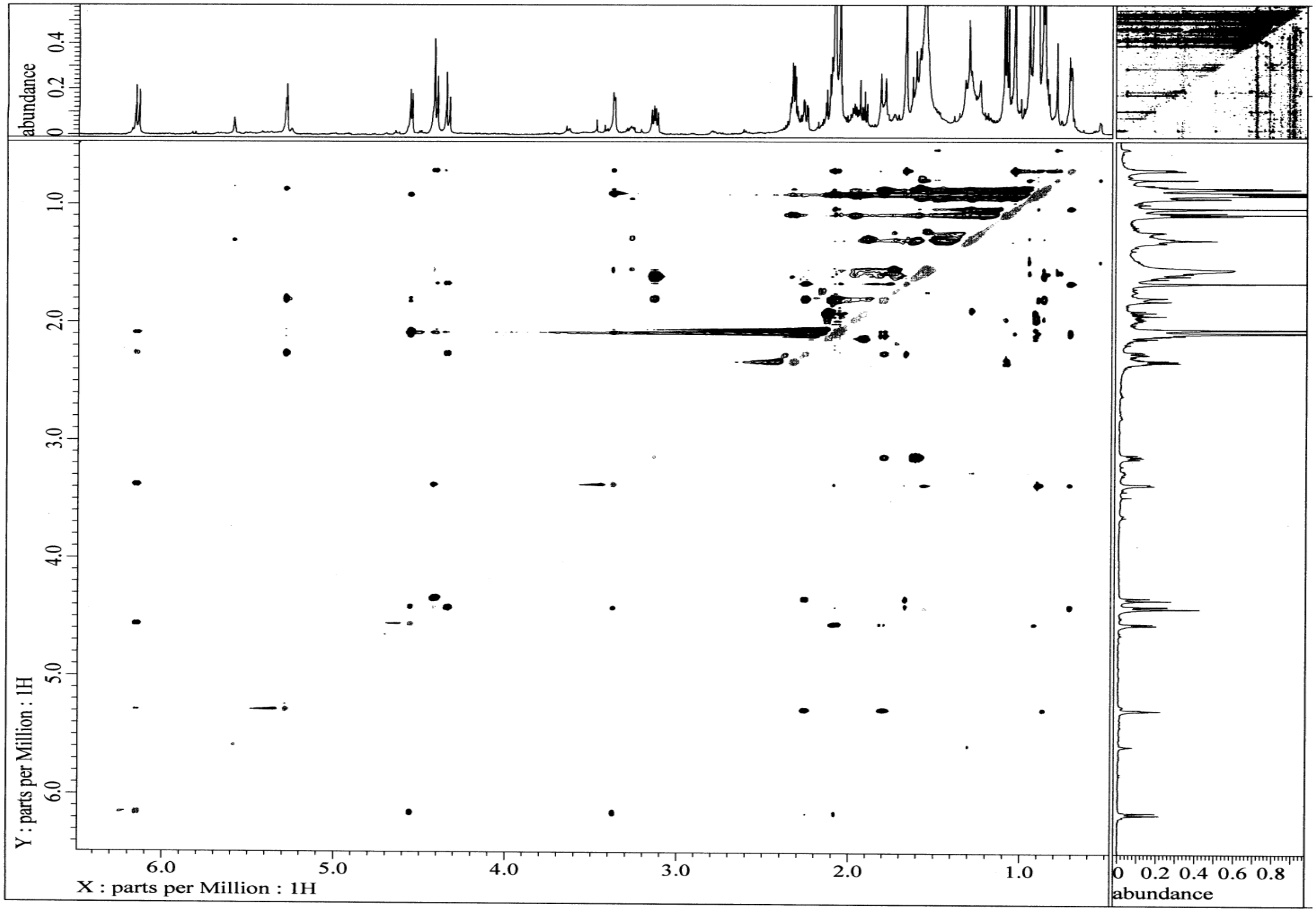

S8. LR-EI-MS of **1**

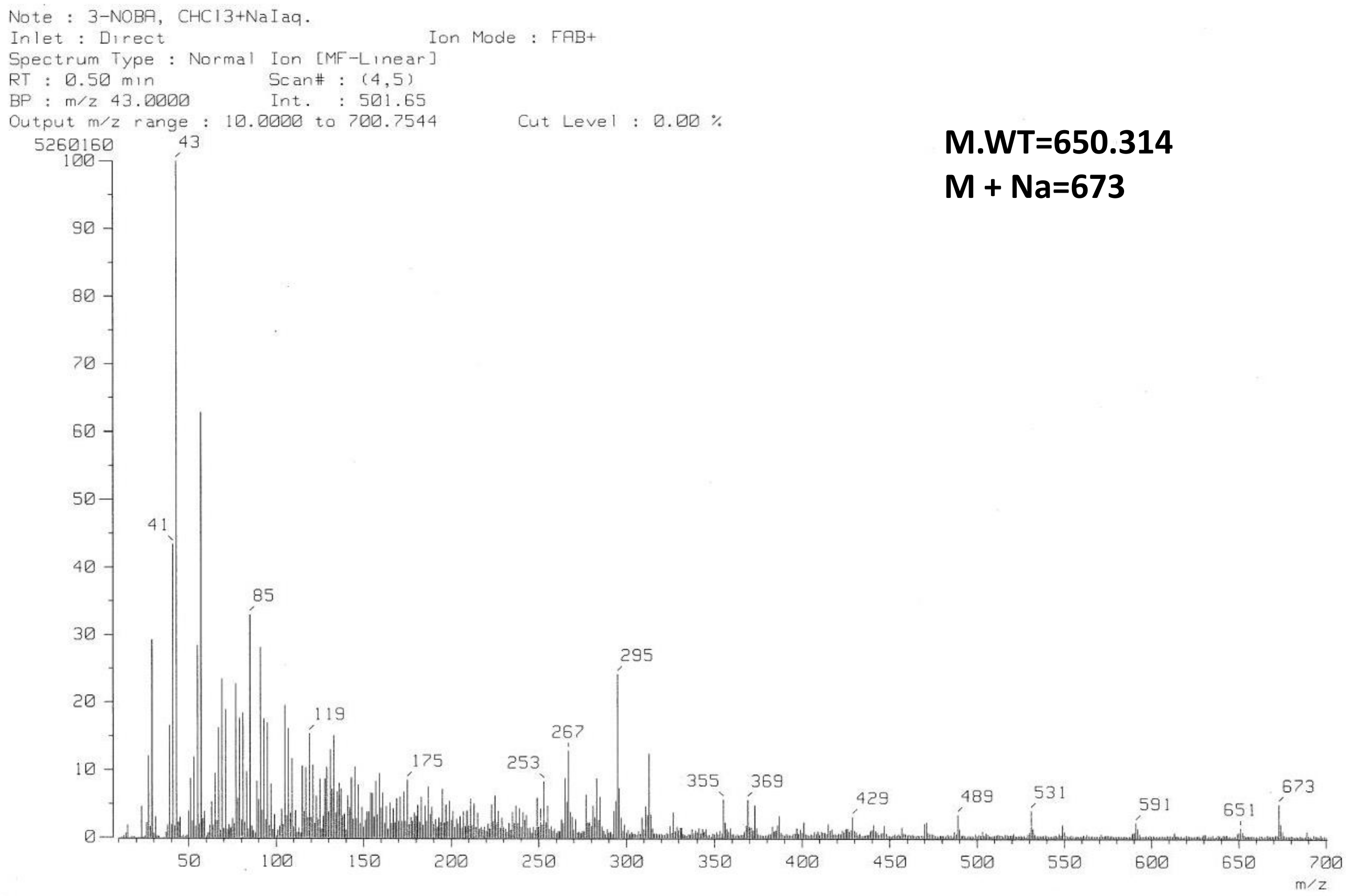

M.WT=650.314
M + Na=673

S9. . HR-EI-MS of **1**

```
Note  :  3-NOBA,  CHCl3+NaIaq.
Inlet  :  Direct                                Ion  Mode  :  FAB+
RT  :  0.60  min                                      Scan#:  3
Elements  :  C  34/0,  H  60/0,  O  12/0,  Na  1/0
Mass  Tolerance        :  1000ppm,  3mmu  if  m/z  <  3,  5mmu  if  m/z  >  5
Unsaturation  (U.S.)  :  -0.5  -  30.0

Observed  m/z  Int%     Err[ppm  /  mmu]      U.S.   Composition
  673.3203      10.7        +0.5  /   +0.3       9.5    C  34  H  50  O  12  Na


[ Theoretical  Ion  Distribution ]                                  Page:  1
Molecular  Formula  :  C34  H50  O12  Na
                     (m/z    673.3200,  MW     673.7536,  U.S.    9.5)
Base  Peak  :     673.3200,  Averaged  MW  :     673.7479(a),     673.7486(w)

    m/z        INT.
  673.3200  100.0000  ************************************************************
  674.3234   38.2731  ***********************
  675.3261    9.5195  ******
  676.3288    1.7753  *
  677.3314    0.2721
  678.3340    0.0357
  679.3365    0.0041
  680.3390    0.0004
```

S10. ^{1}H NMR (600 MHz, $CDCl_3$) spectrum of **2**

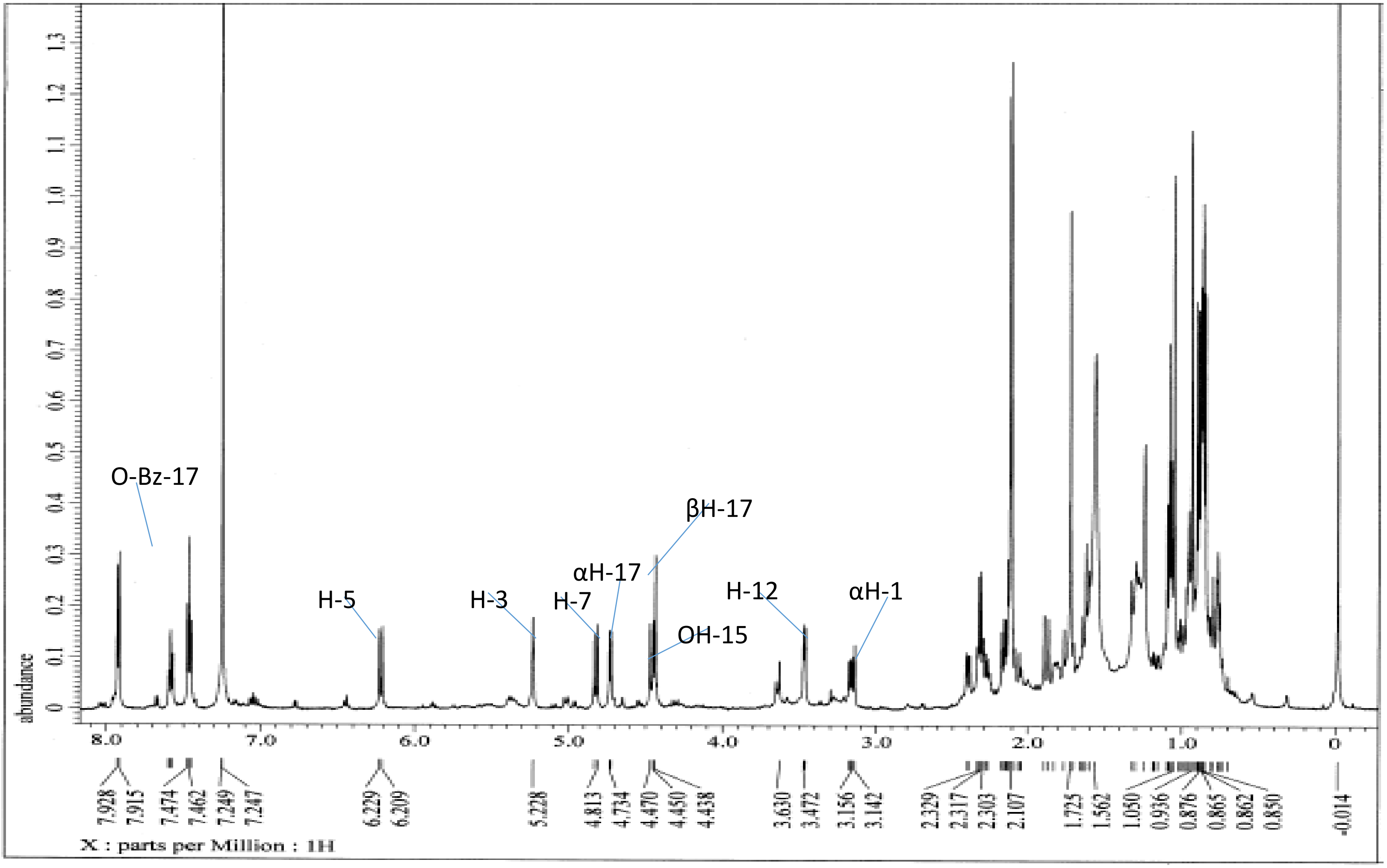

S10. ^{1}H NMR (600 MHz, $CDCl_3$) spectrum of **2**

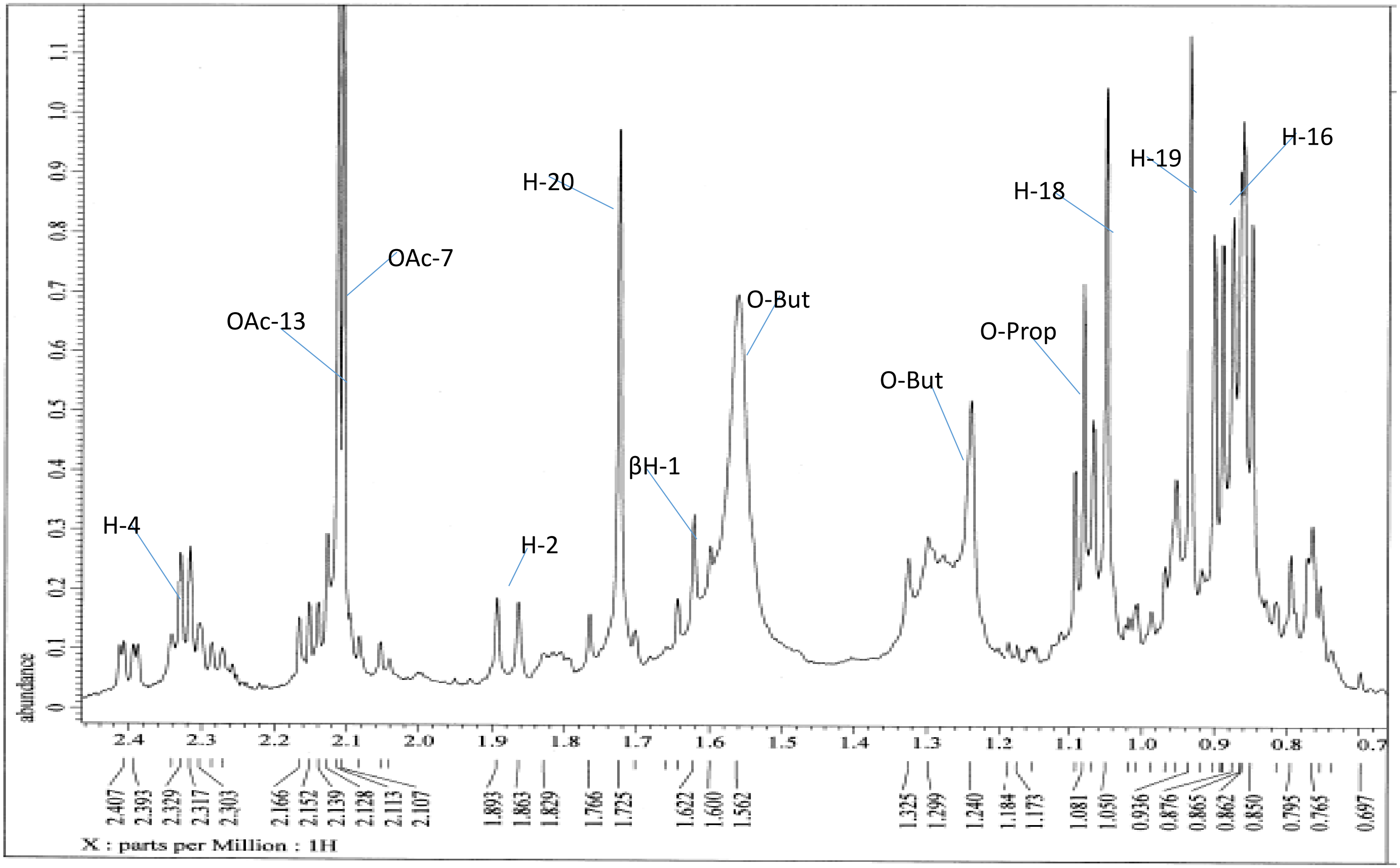

S11. ^{13}C NMR (150 MHz, $CDCl_3$) spectrum of **2**

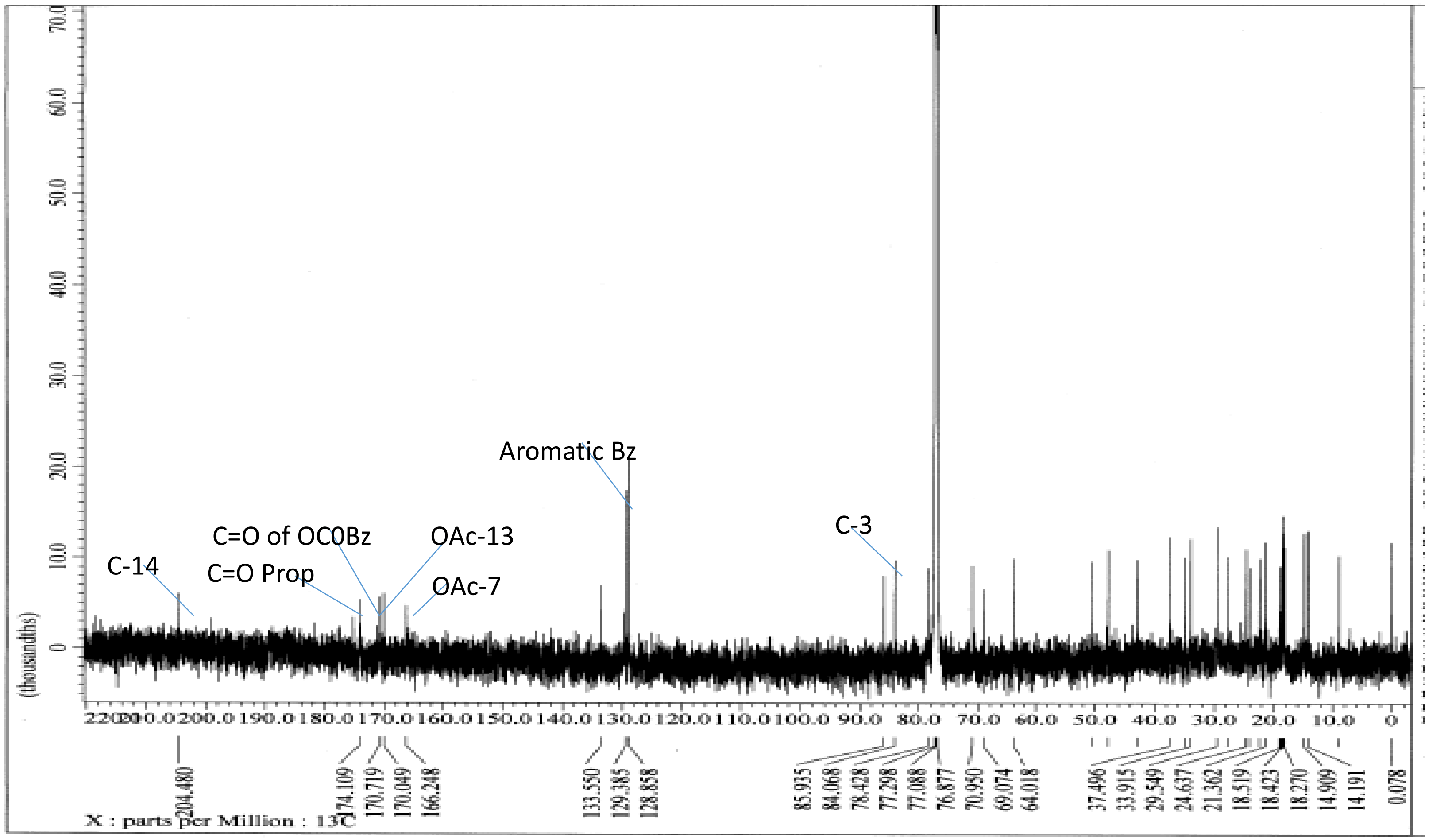

S11. ^{13}C NMR (150 MHz, $CDCl_3$) spectrum of **2**

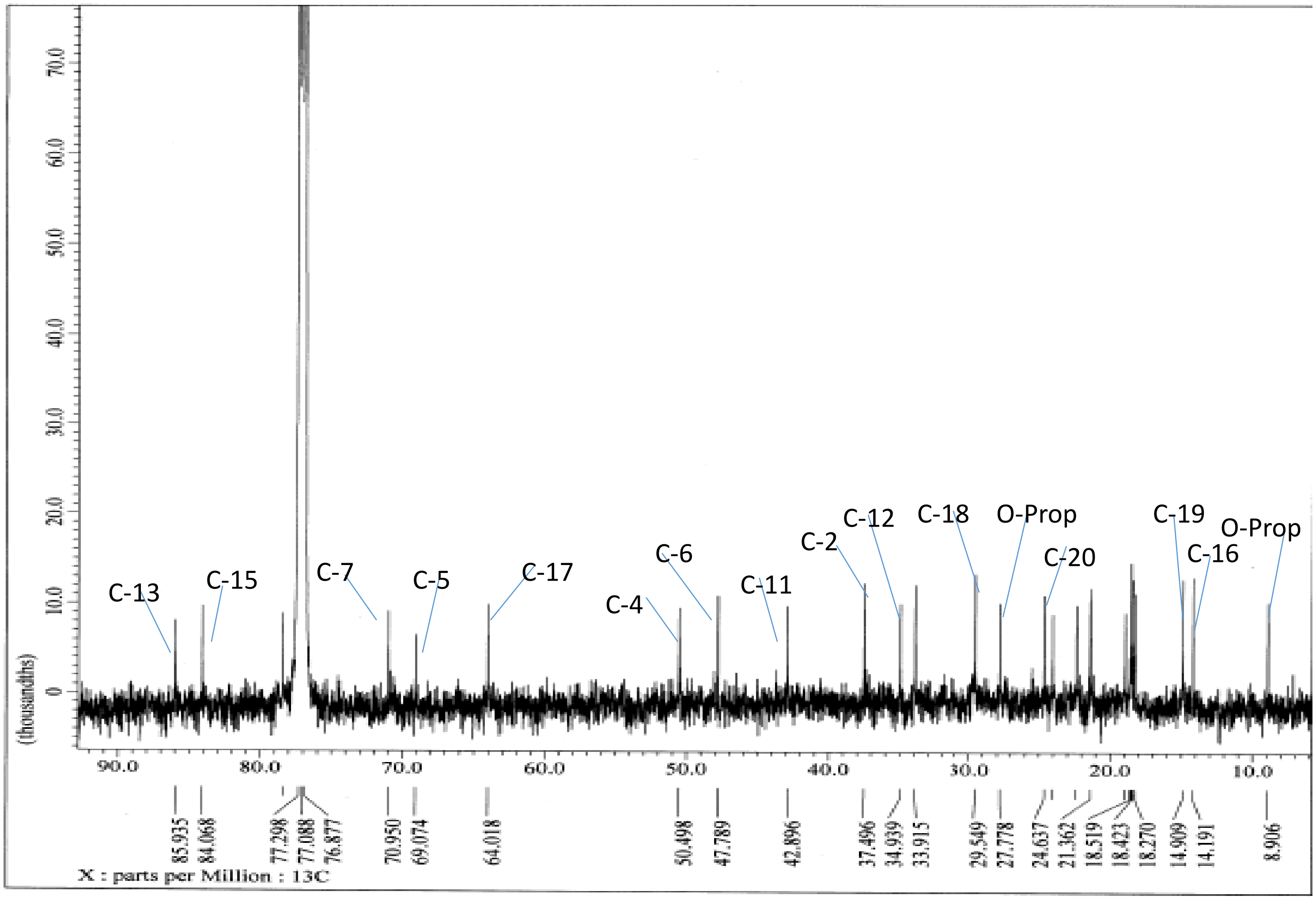

S12. DEPT (150 MHz, $CDCl_3$) spectra of **2**

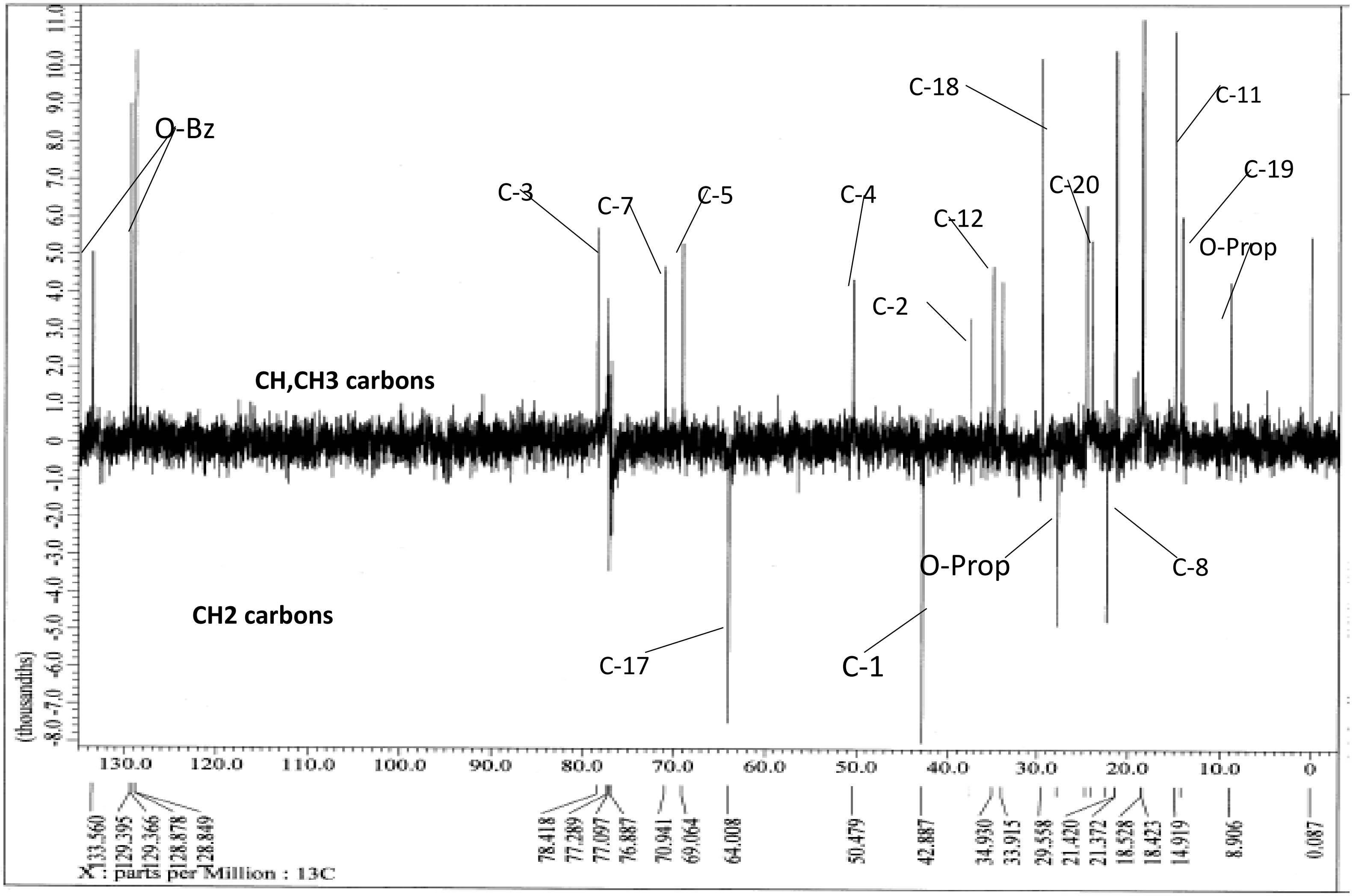

S13. ^{1}H ^{1}H COSY spectrum of **2** in $CDCl_3$

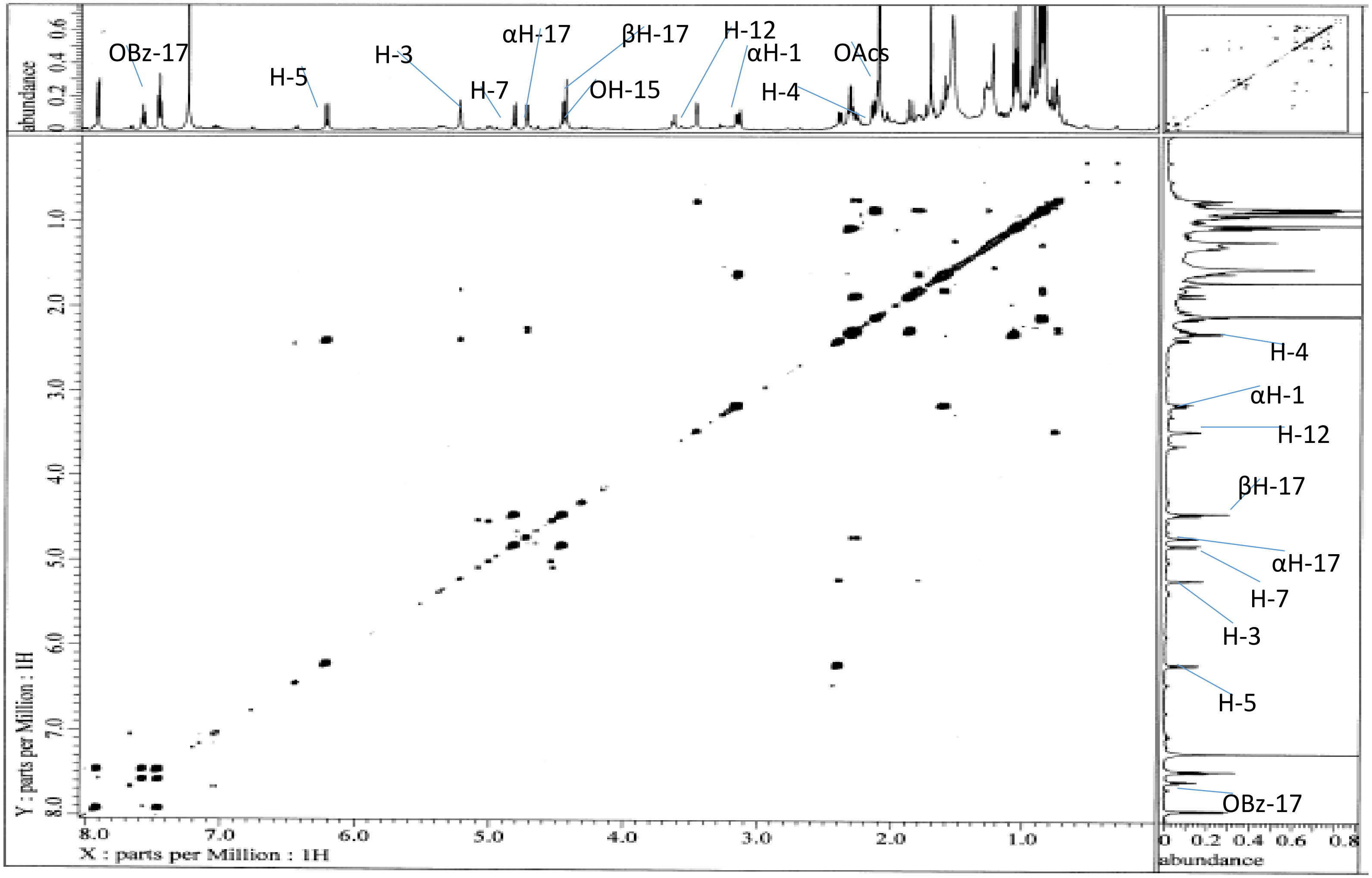

S13. ^1H ^1H COSY spectrum of **2** in $CDCl_3$

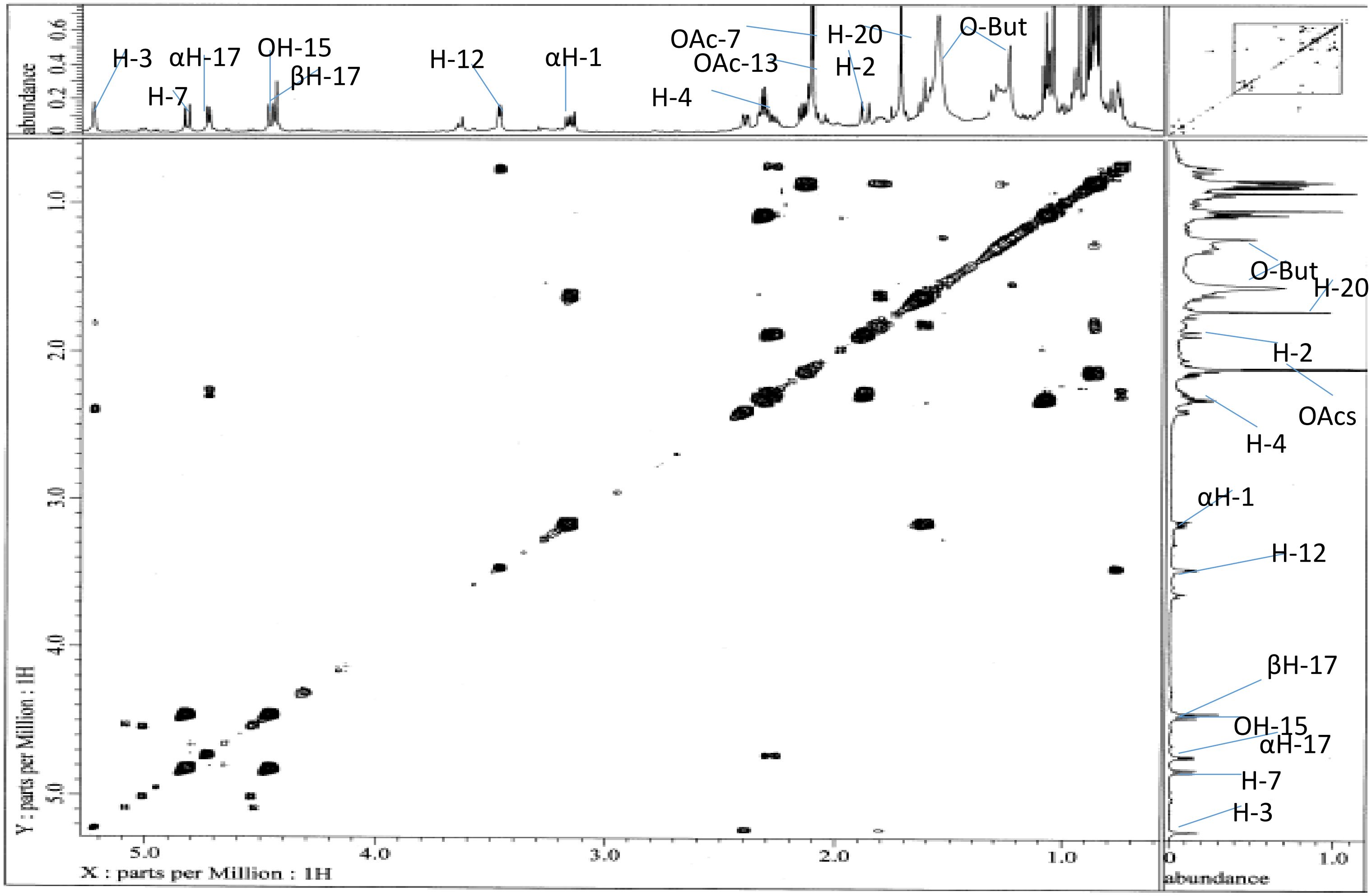

S14. HMBC spectrum of **2** in $CDCl_3$

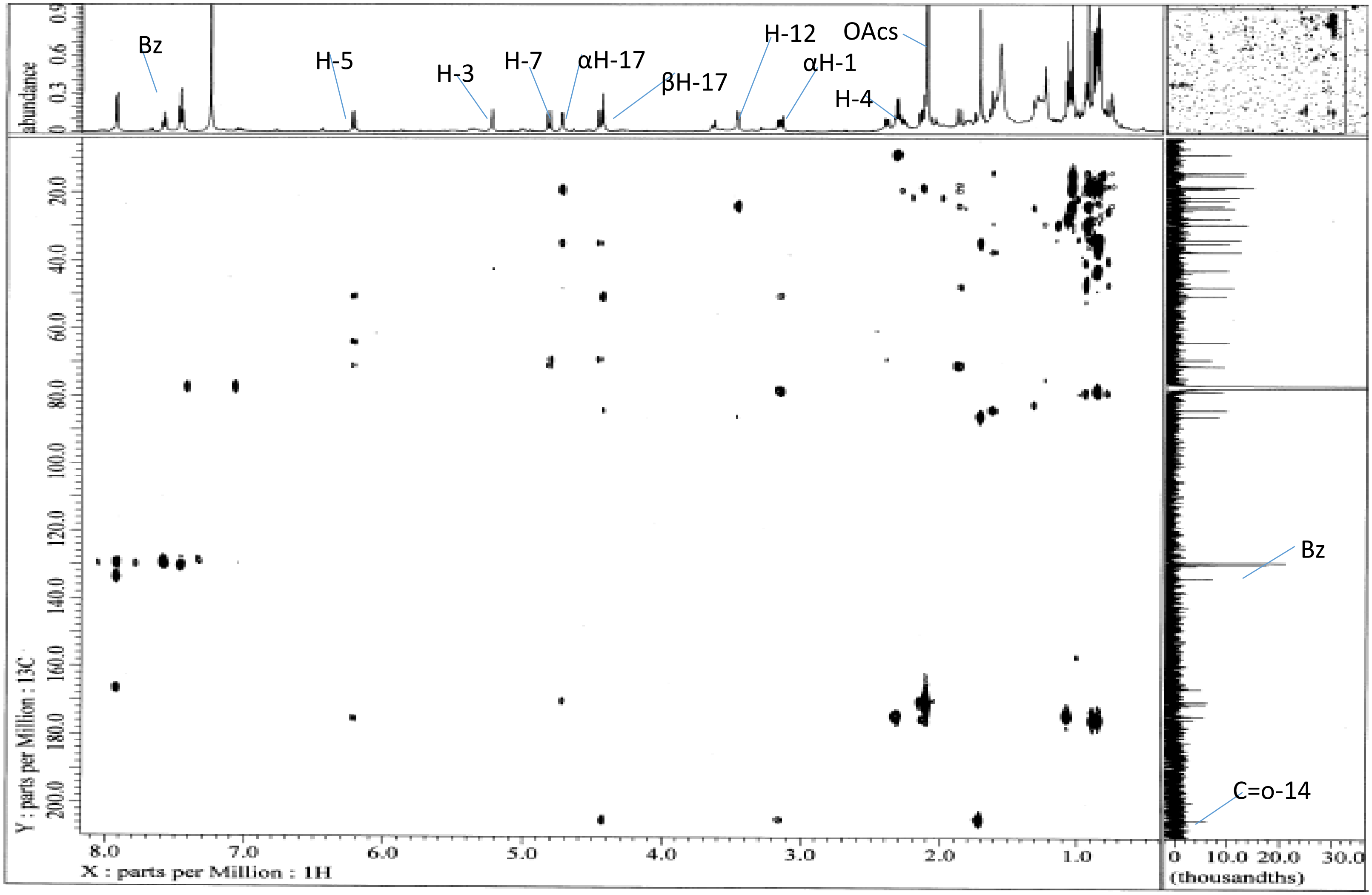

S14. HMBC spectrum of **2** in $CDCl_3$

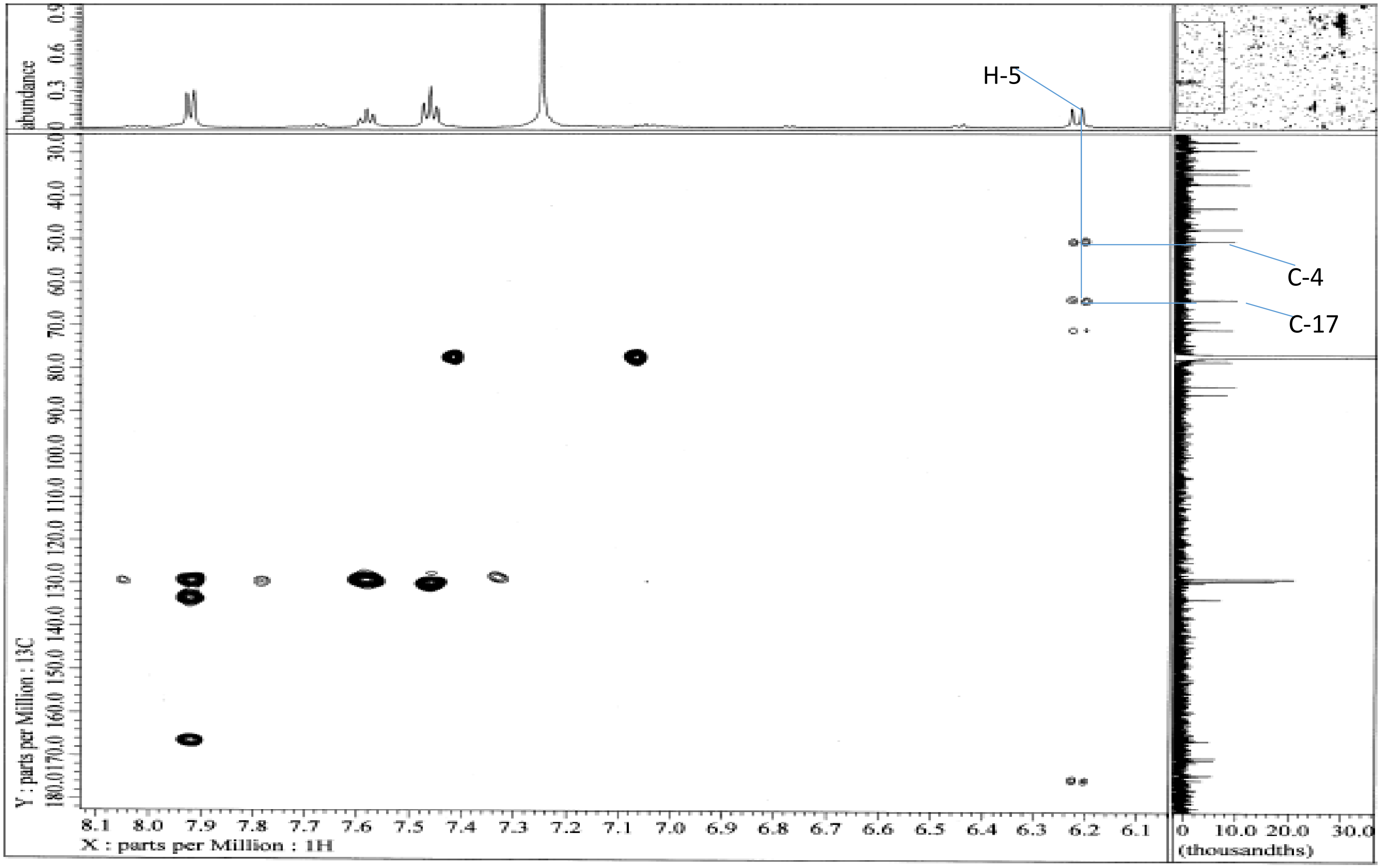

S14. HMBC spectrum of **2** in $CDCl_3$

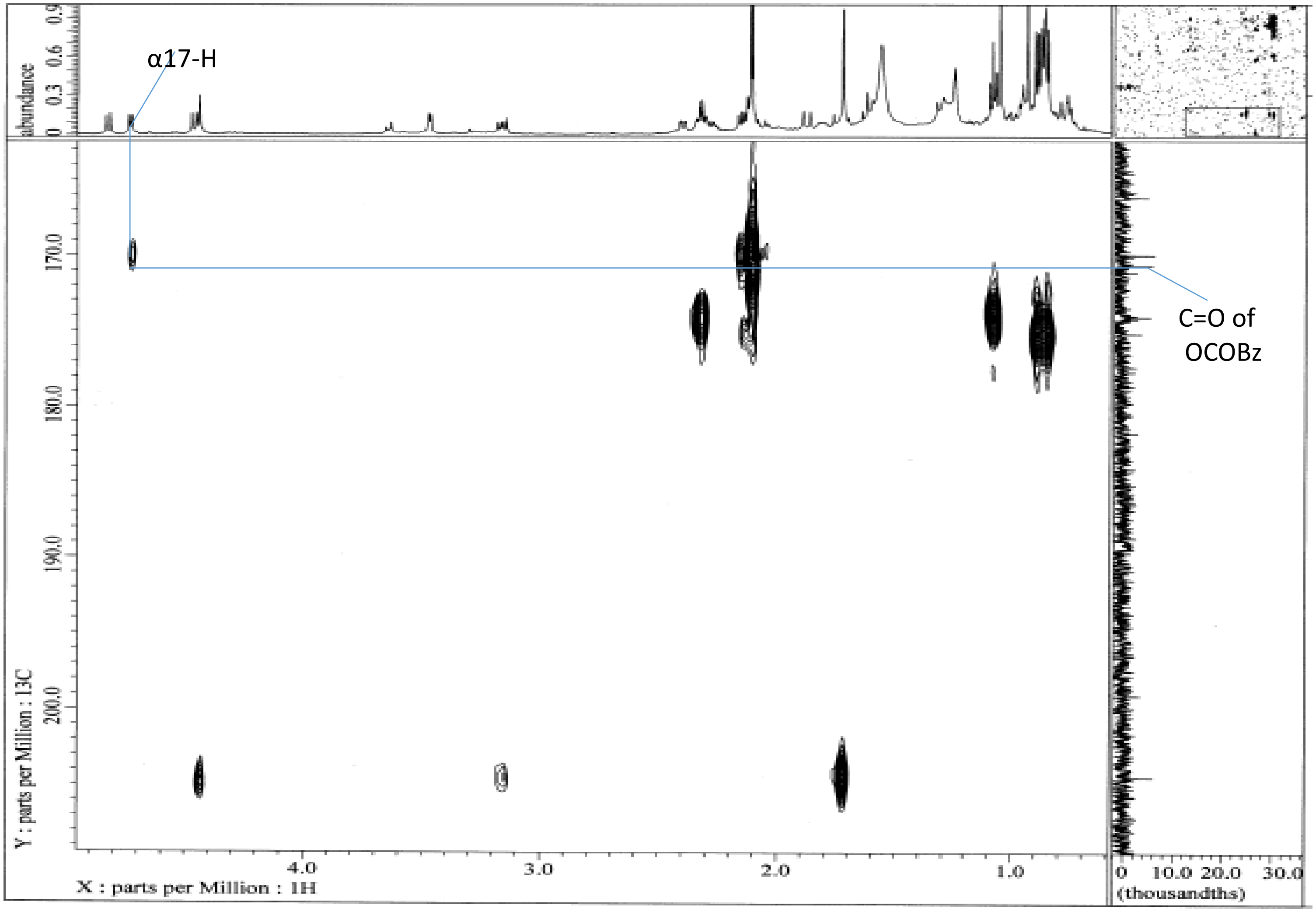

S15. HMQC spectrum of **2** in $CDCl_3$

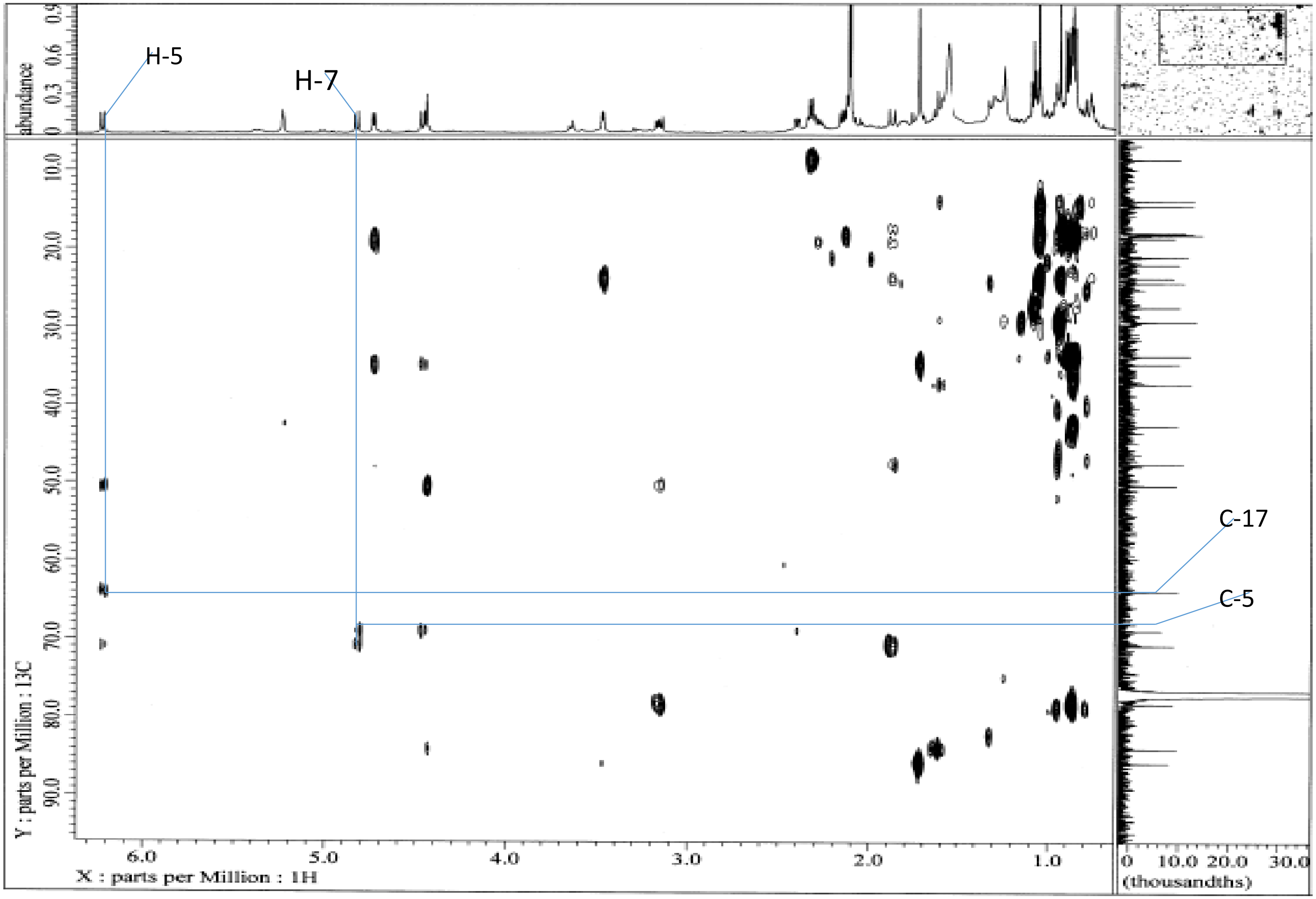

S15. HMQC spectrum of **2** in $CDCl_3$

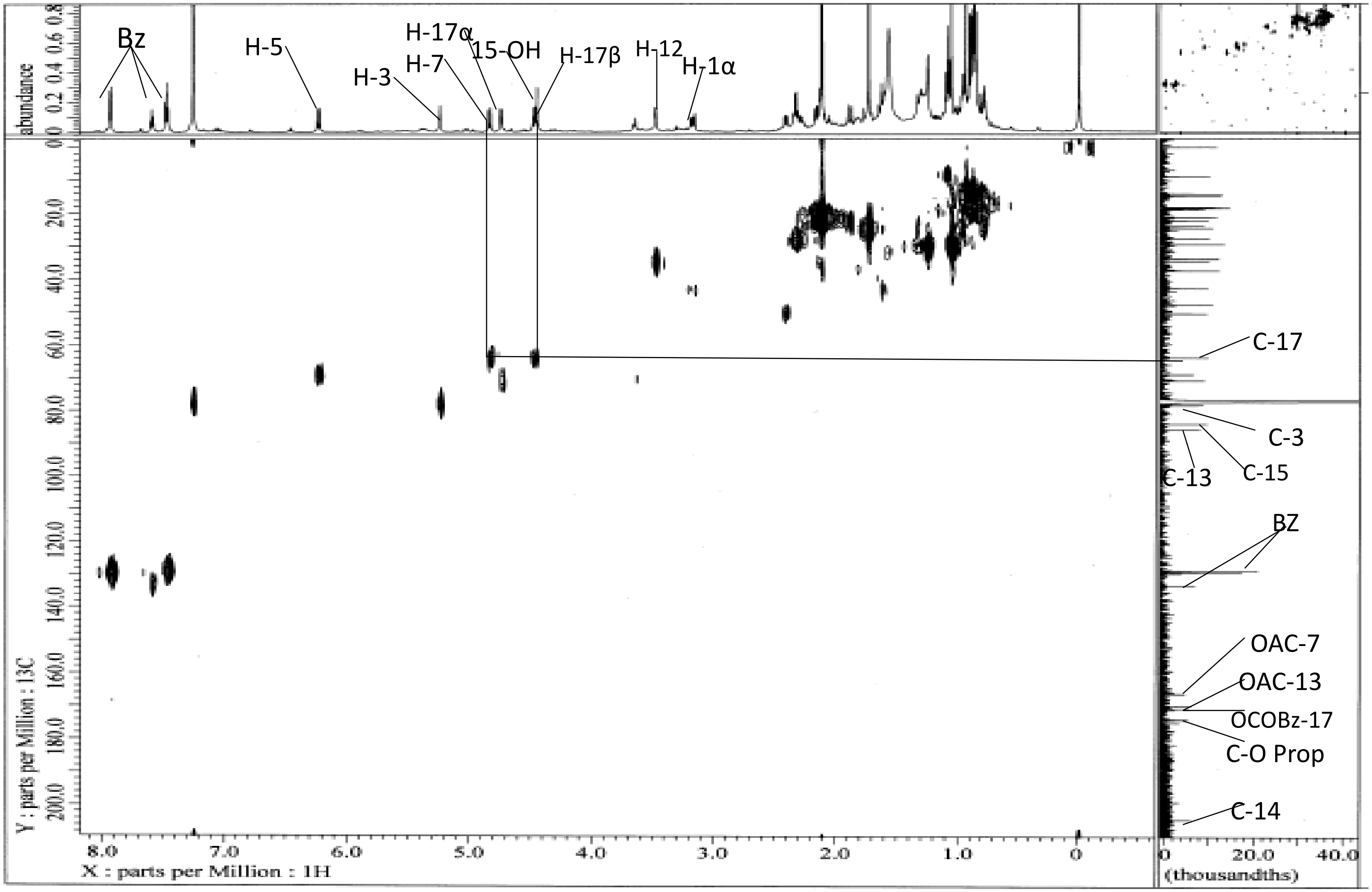

S16. HMQC spectrum of **2** in $CDCl_3$

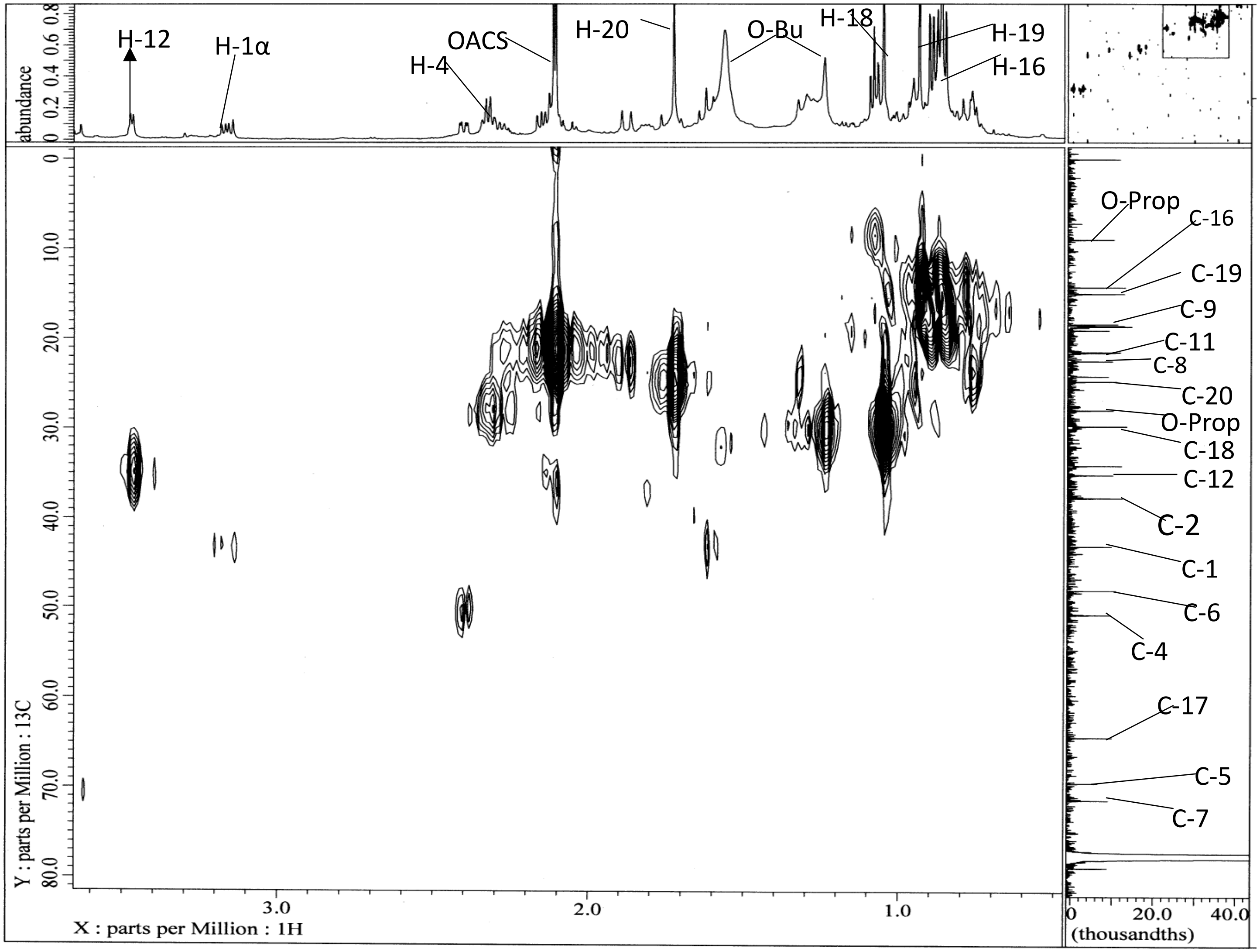

S16. NOESY spectrum of **2** in $CDCl_3$

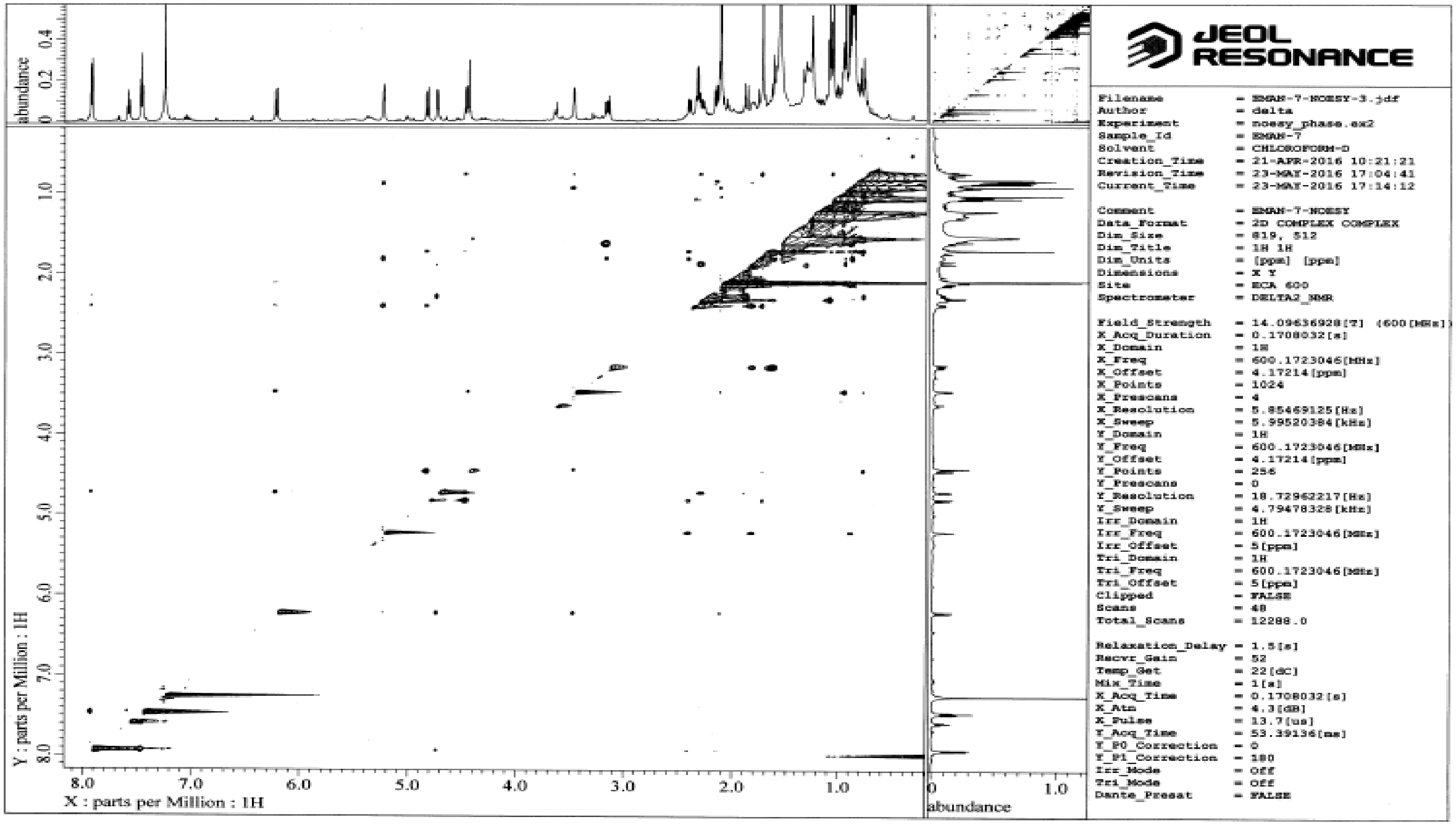

S17, . LR-EI-MS of **2**

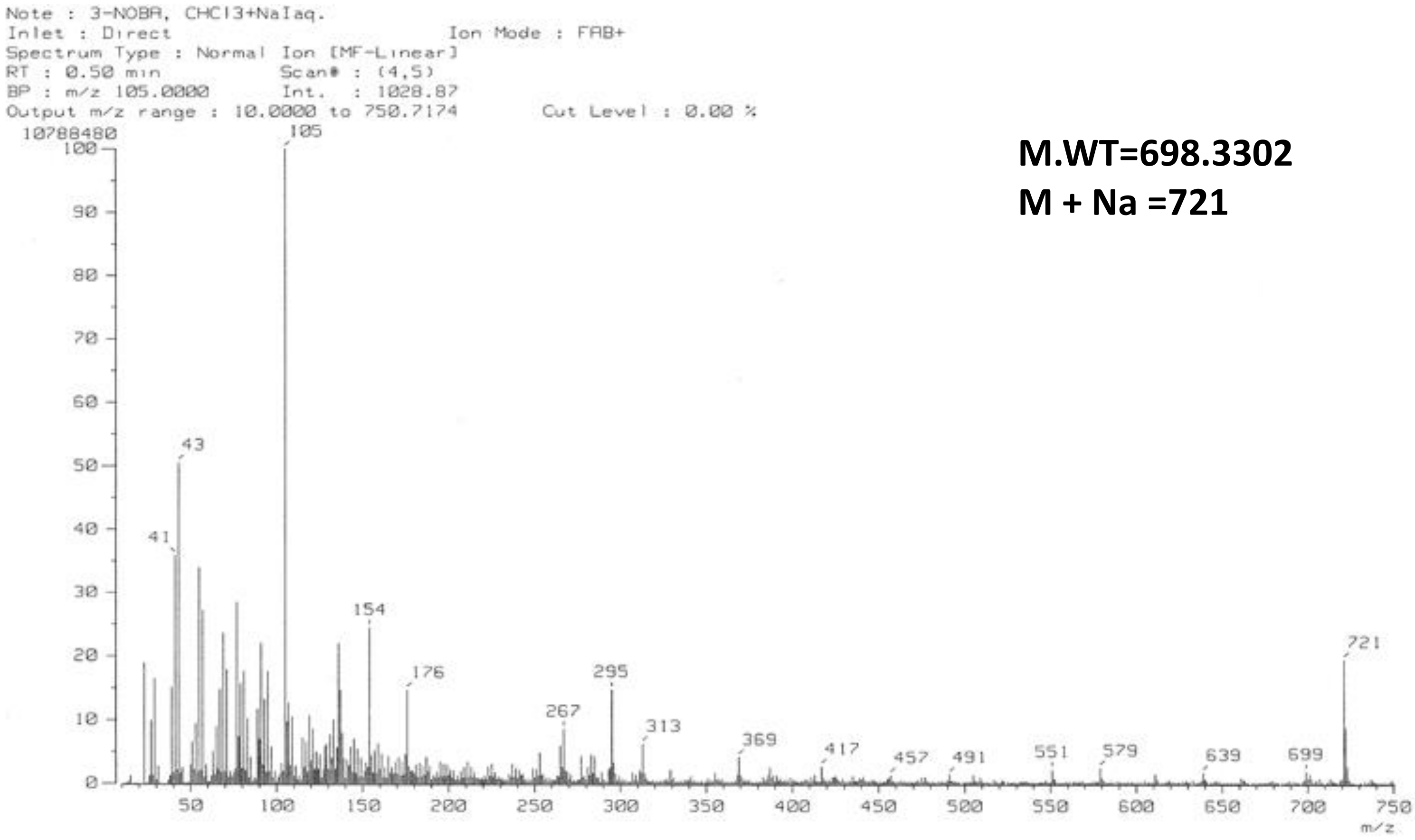

M.WT=698.3302
M + Na =721

S18. HR-EI-MS of **2**

Note : 3-NOBA, CHCl3+NaIaq.
Inlet : Direct   Ion Mode : FAB+
RT : 0.30 min   Scan#: 2
Elements : C 38/0, H 60/0, O 12/0, Na 1/0
Mass Tolerance : 1000ppm, 3mmu if m/z < 3, 5mmu if m/z > 5
Unsaturation (U.S.) : -0.5 - 30.0

Observed m/z	Int%	Err[ppm / mmu]	U.S.	Composition
721.3206	100.0	+0.9 / +0.6	13.5	C 38 H 50 O 12 Na

[Theoretical Ion Distribution]   Page: 1
Molecular Formula : C38 H50 O12 Na
(m/z 721.3200, MW 721.7976, U.S. 13.5)
Base Peak : 721.3200, Averaged MW : 721.7921(a), 721.7928(w)

m/z	INT.	
721.3200	100.0000	**
722.3234	42.7220	**************************
723.3262	11.2964	*******
724.3289	2.2277	*
725.3316	0.3584	
726.3342	0.0492	
727.3368	0.0059	
728.3394	0.0006	

S19. ^1H NMR (600 MHz, $CDCl_3$) spectrum of **3**

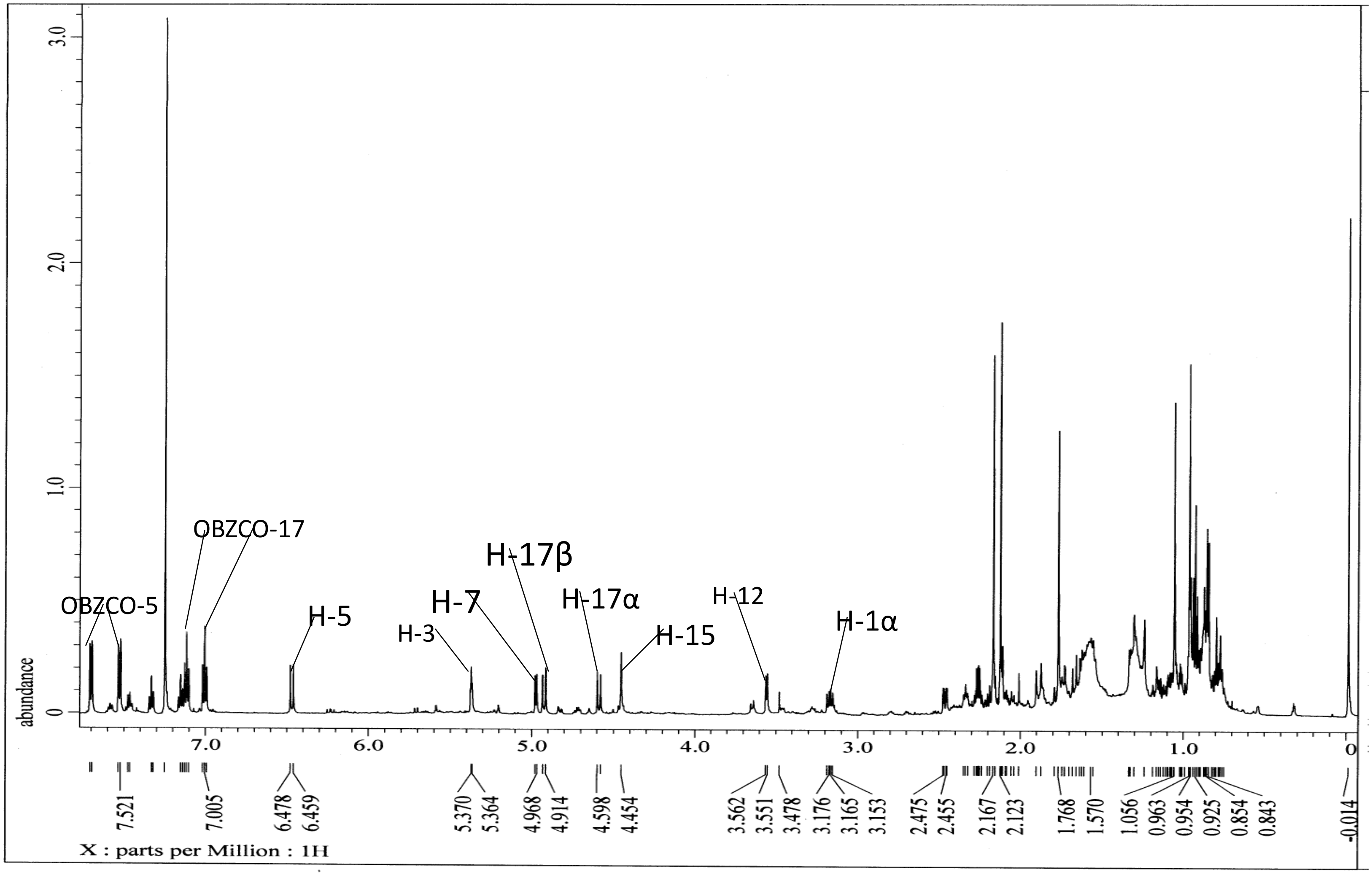

S19. ^{1}H NMR (600 MHz, $CDCl_3$) spectrum of **3**

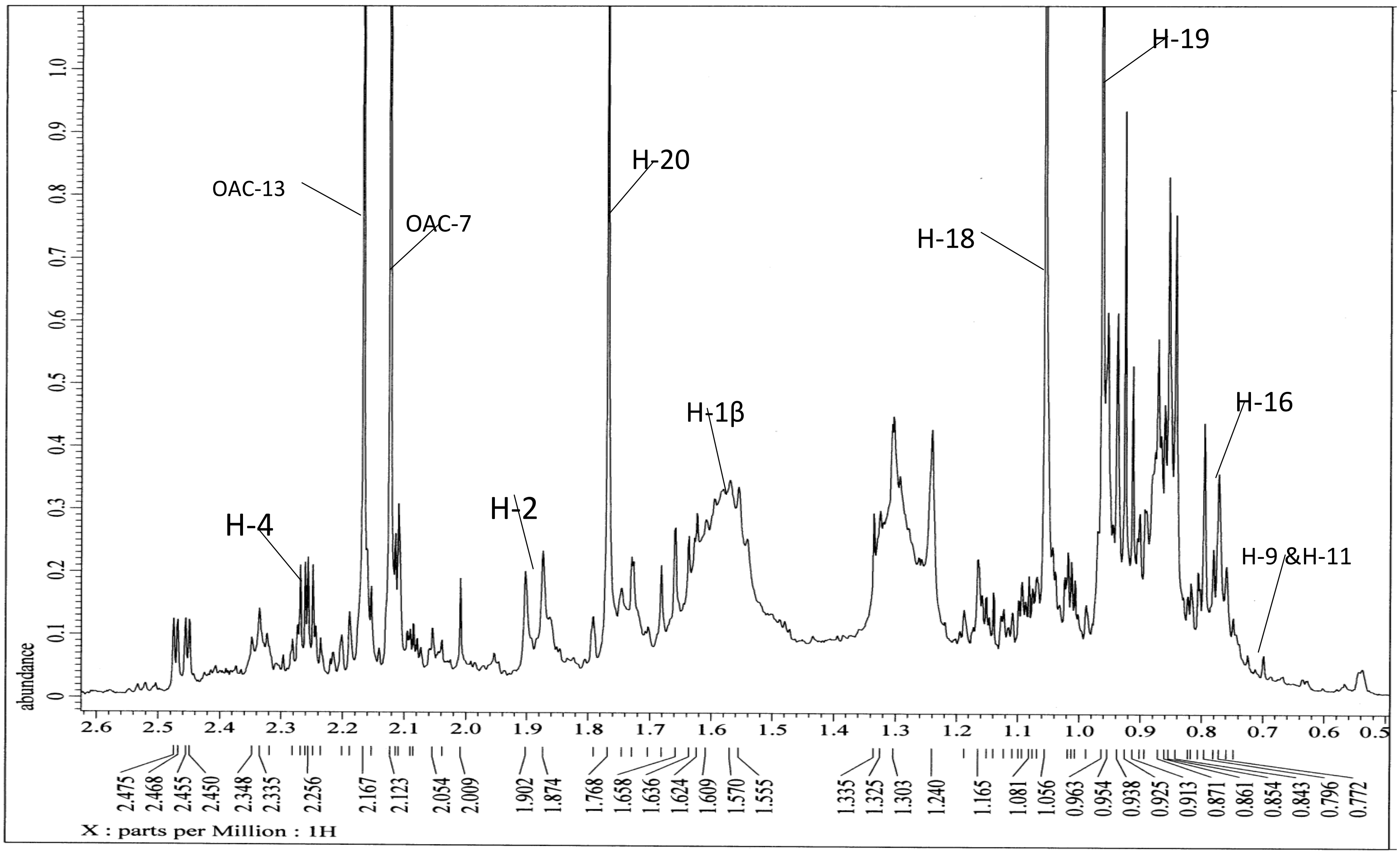

S20. ^{13}C NMR (150 MHz, $CDCl_3$) spectrum of **3**

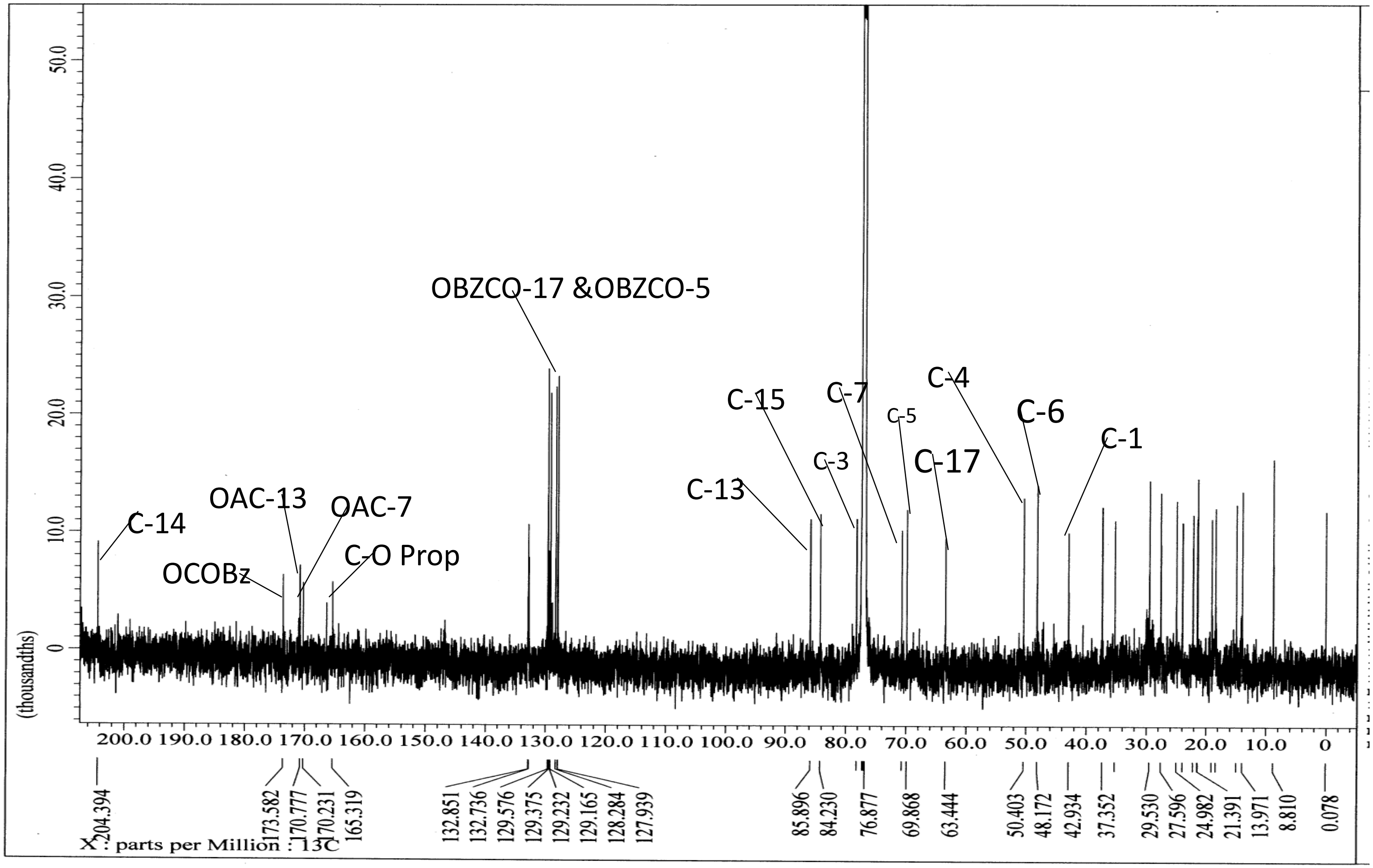

S21. DEPT (150 MHz, $CDCl_3$) spectra of **3**

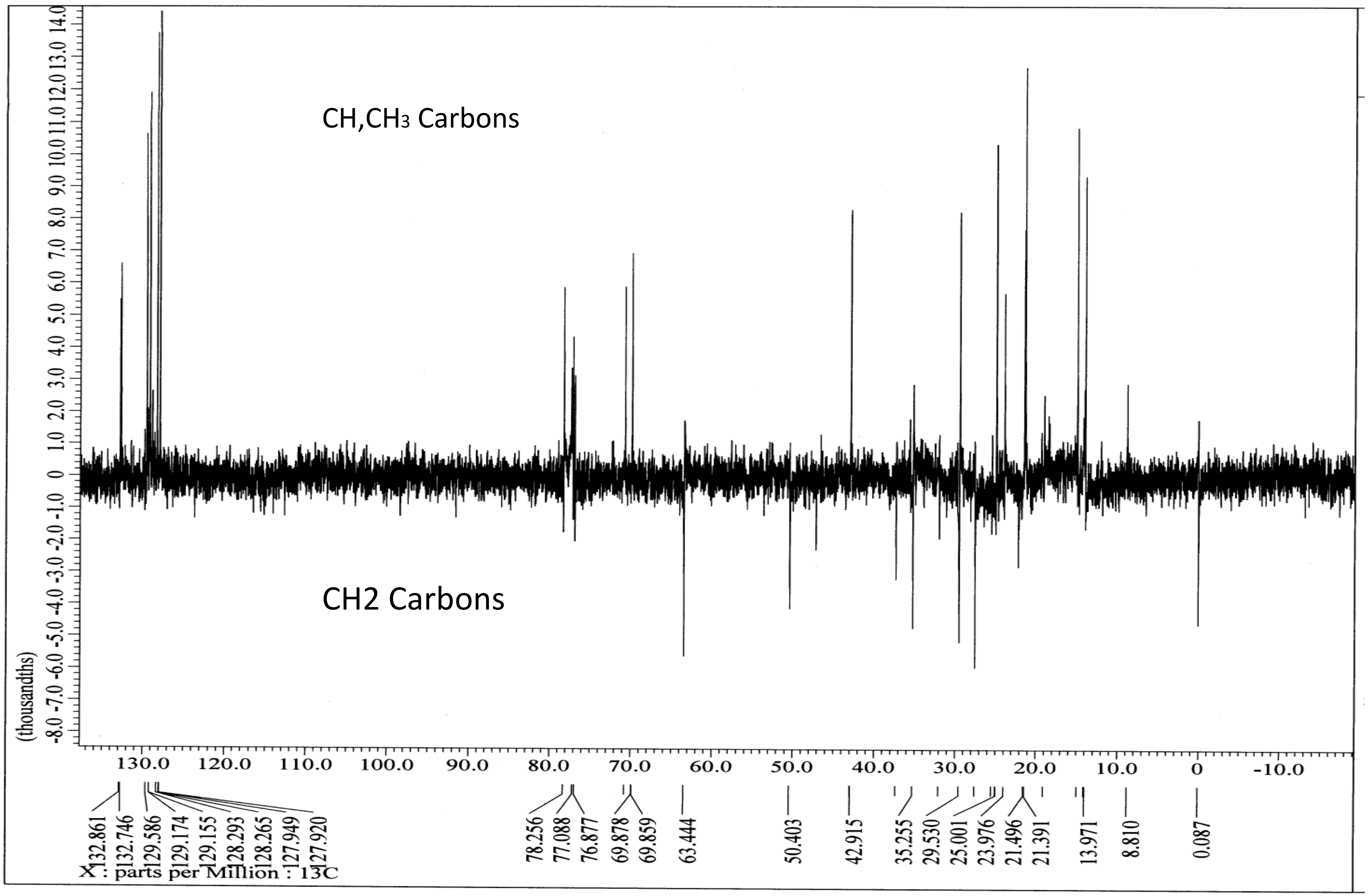

S22. ^{1}H ^{1}H COSY spectrum of **3** in $CDCl_3$

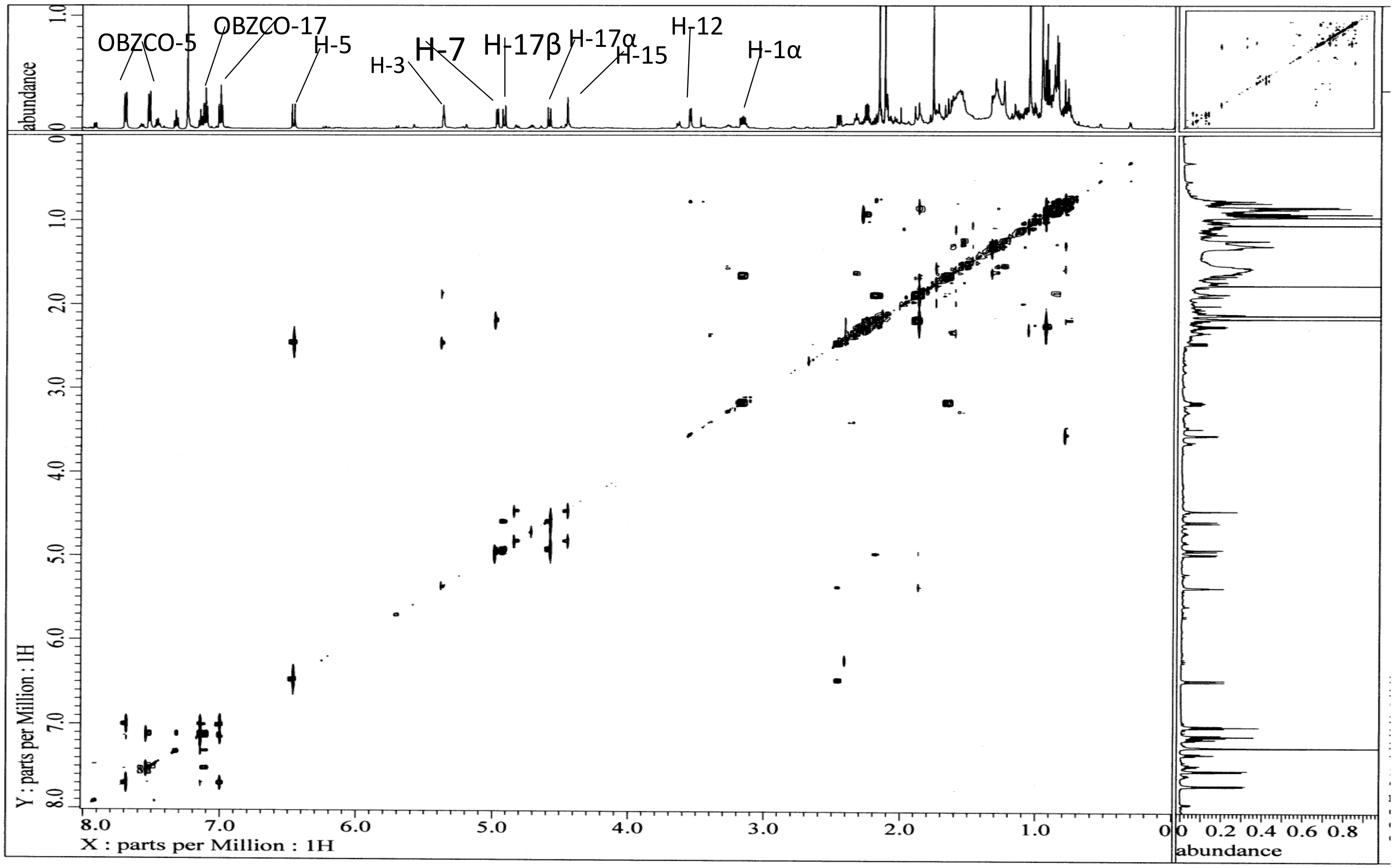

S23. HMBC spectrum of **3** in $CDCl_3$

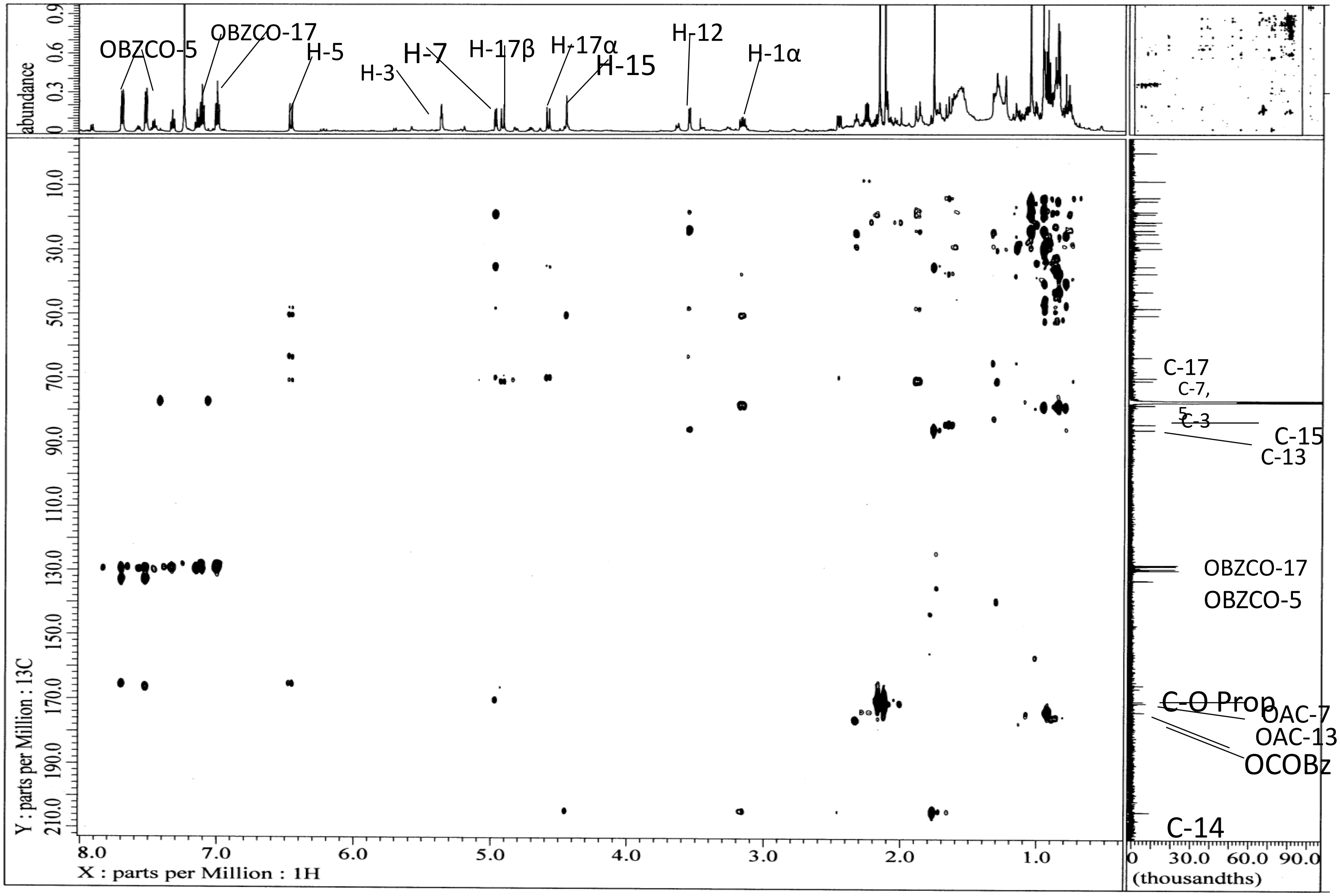

S23. HMBC spectrum of **3** in $CDCl_3$

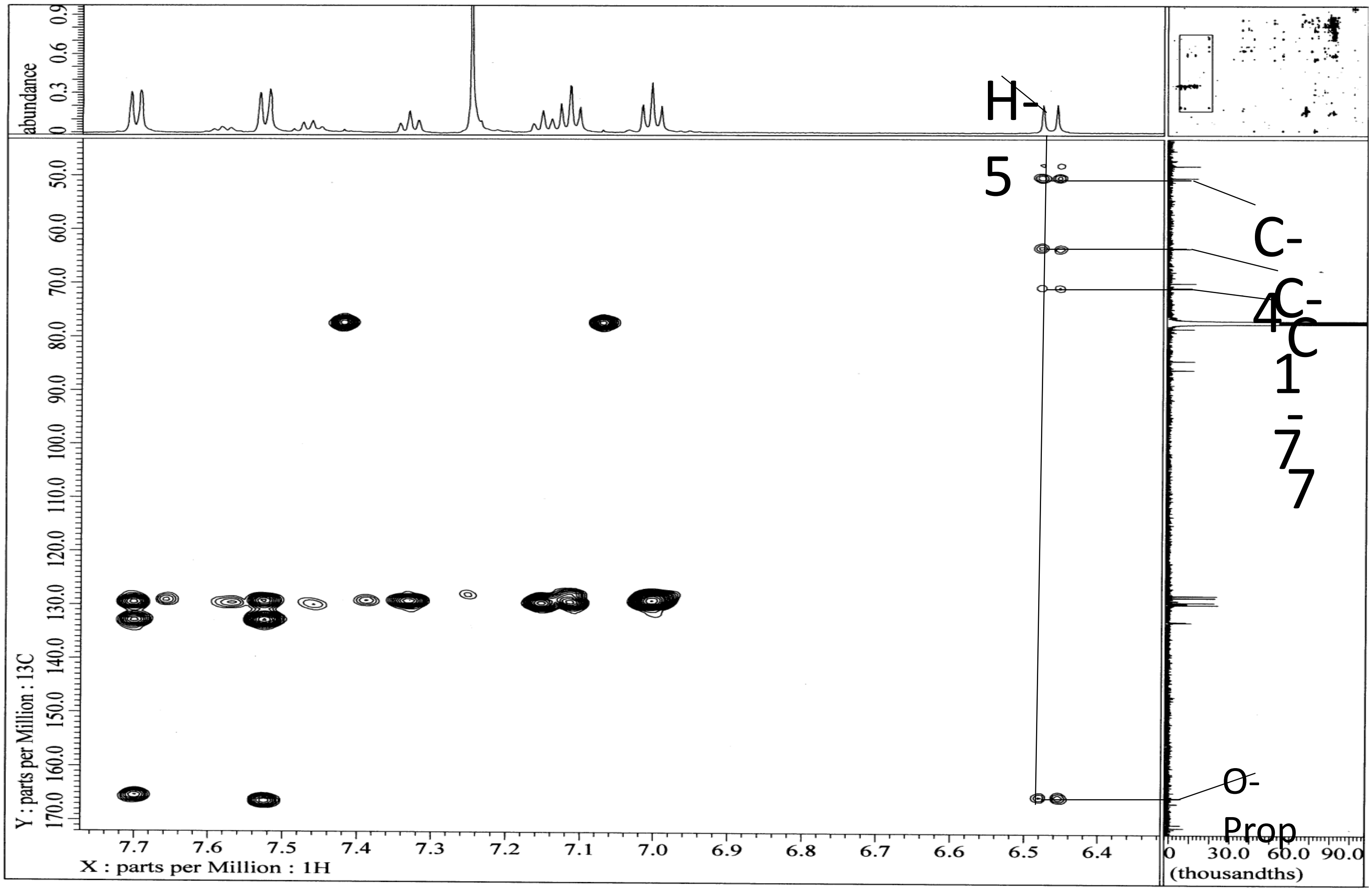

S24. HSQC spectrum of **3** in $CDCl_3$

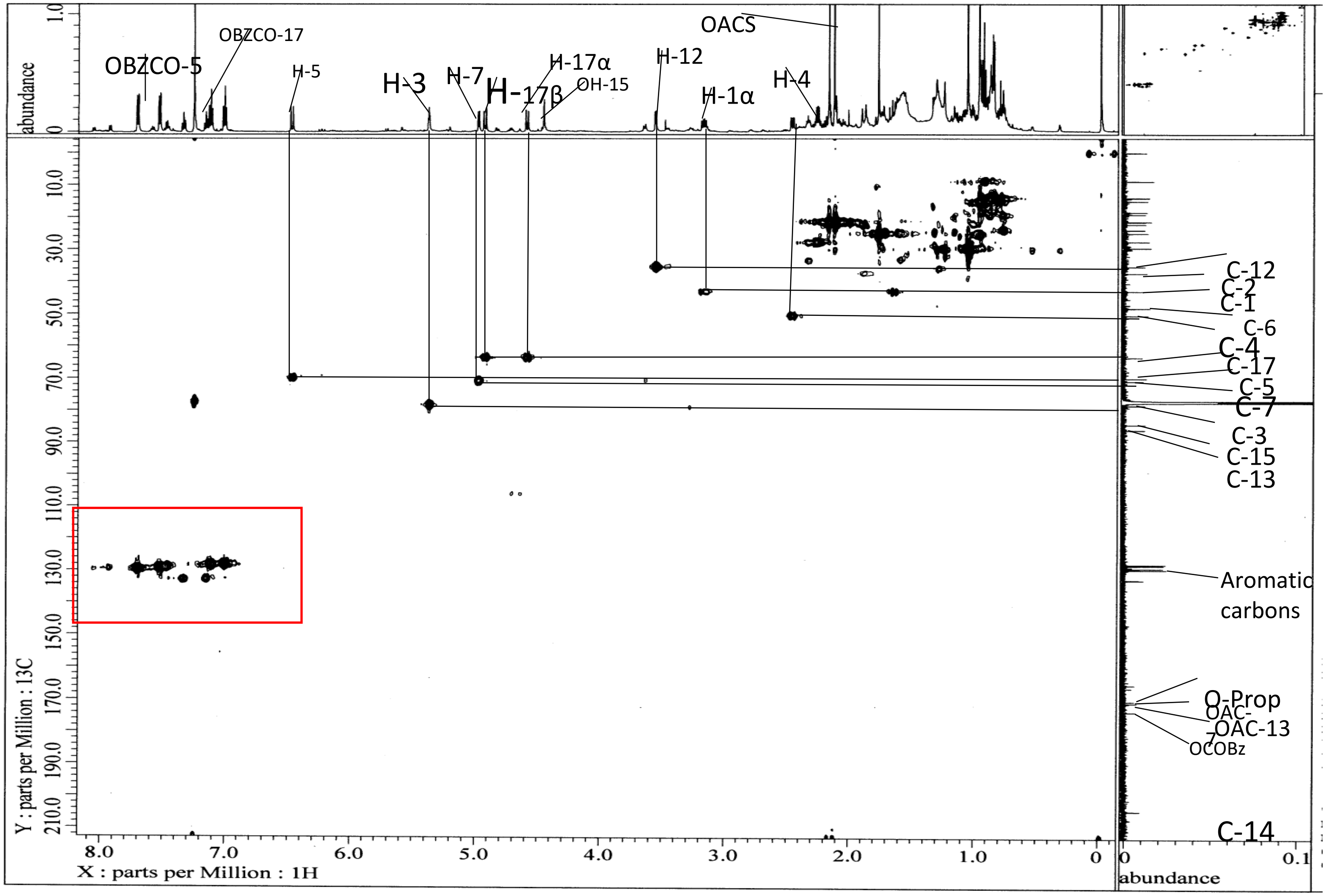

S25. NOESY spectrum of **3** in $CDCl_3$

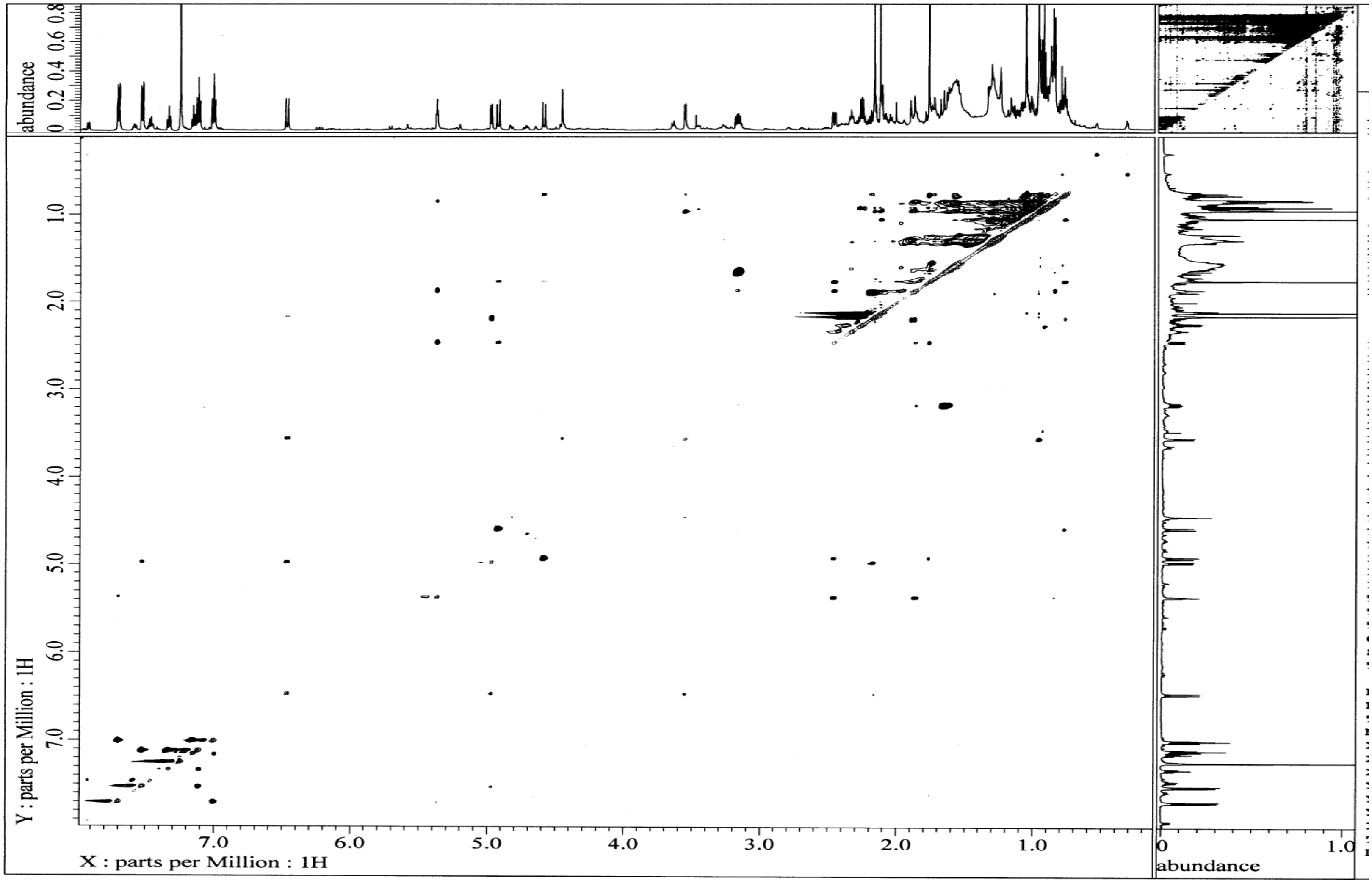

S26. LR-EI-MS of **3**

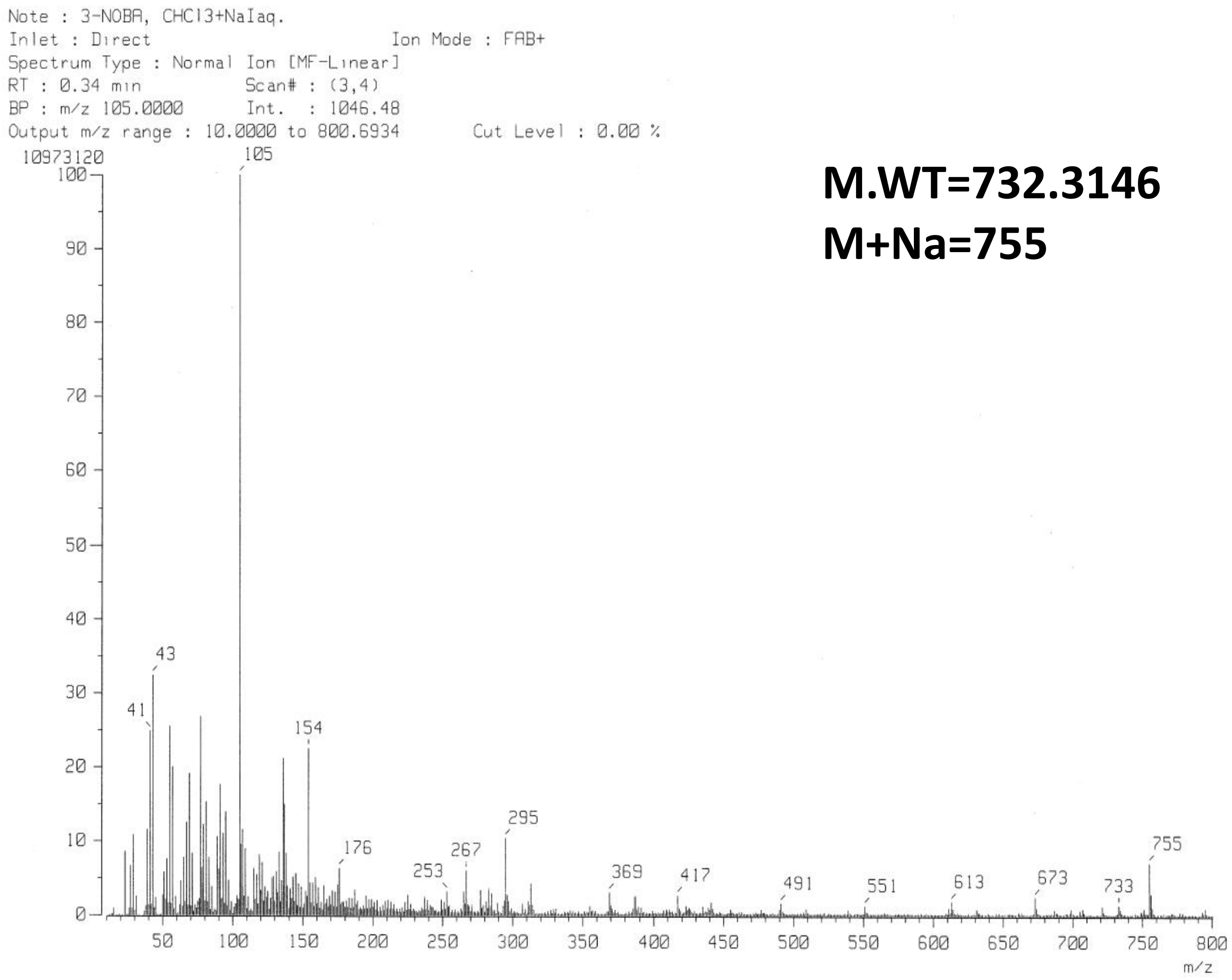

M.WT=732.3146
M+Na=755

S27. HR-EI-MS of **3**

Note : 3-NOBA, CHCl3+NaIaq.
Inlet : Direct Ion Mode : FAB+
RT : 0.30 min Scan#: 2
Elements : C 41/0, H 50/0, O 12/0, Na 1/0
Mass Tolerance : 1000ppm, 3mmu if m/z < 3, 5mmu if m/z > 5
Unsaturation (U.S.) : -0.5 - 30.0

Observed m/z	Int%	Err[ppm / mmu]	U.S.	Composition
755.3050	100.0	+0.9 / +0.6	17.5	C 41 H 48 O 12 Na

[Theoretical Ion Distribution] Page: 1
Molecular Formula : C41 H48 O12 Na
(m/z 755.3043, MW 755.8147, U.S. 17.5)
Base Peak : 755.3043, Averaged MW : 755.8095(a), 755.8103(w)

m/z	INT.	
755.3043	100.0000	**
756.3077	46.0587	****************************
757.3106	12.7591	*******
758.3134	2.6207	**
759.3161	0.4370	
760.3187	0.0620	
761.3213	0.0077	
762.3239	0.0009	

S28. ^{1}H NMR (600 MHz, $CDCl_3$) spectrum of **4**

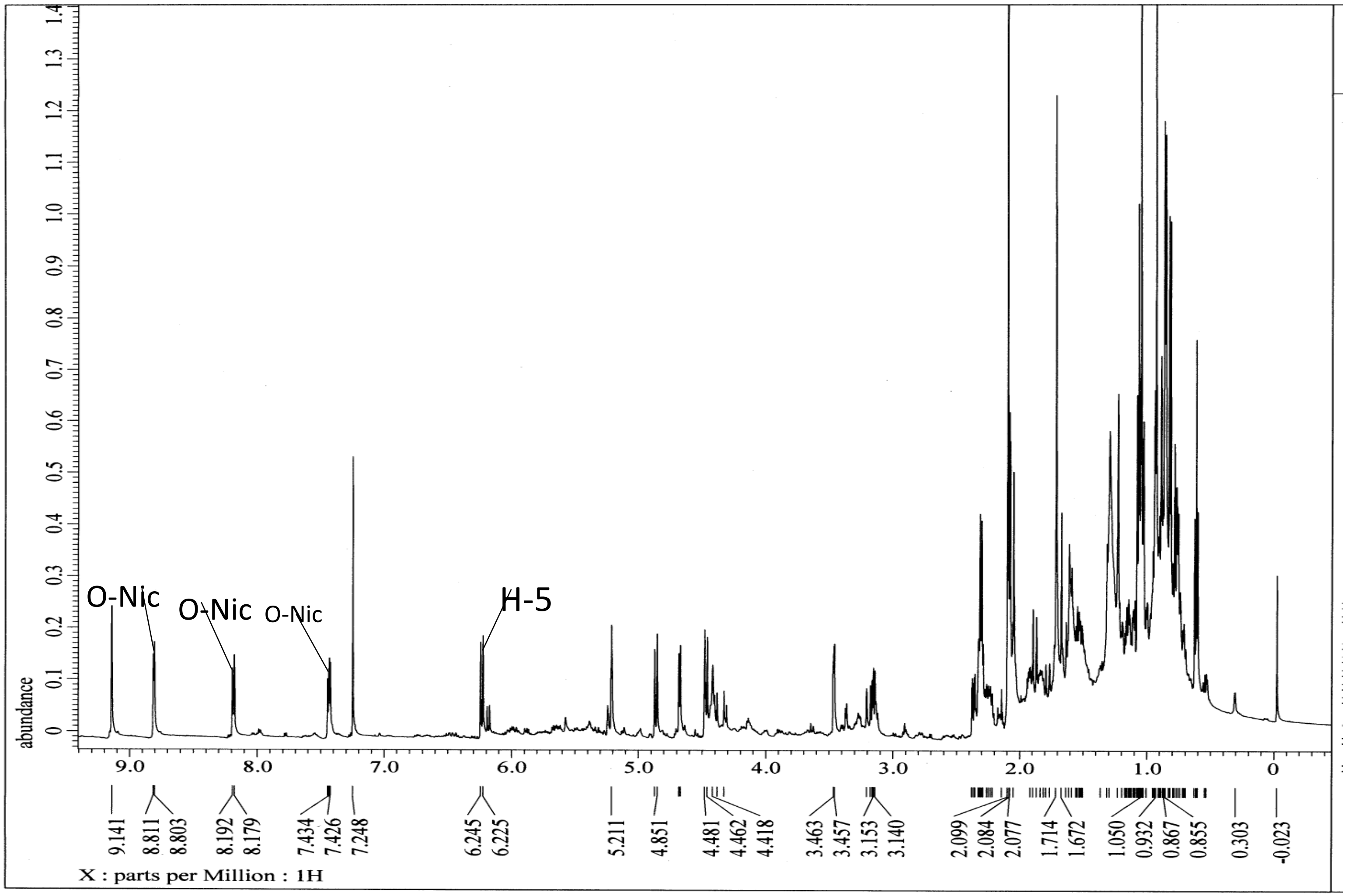

S28. ^{1}H NMR (600 MHz, $CDCl_3$) spectrum of **4**

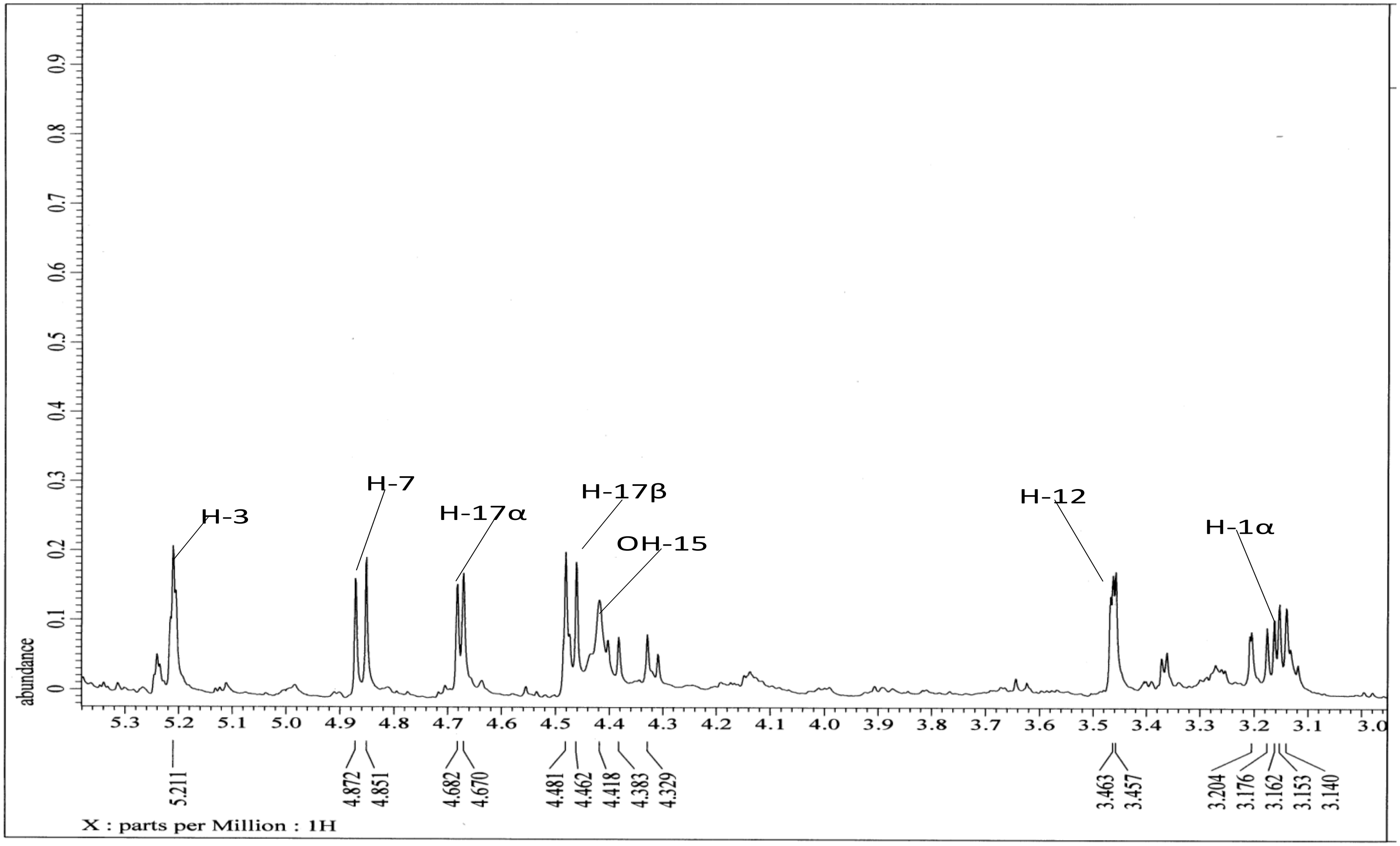

S28. ^{1}H NMR (600 MHz, $CDCl_3$) spectrum of **4**

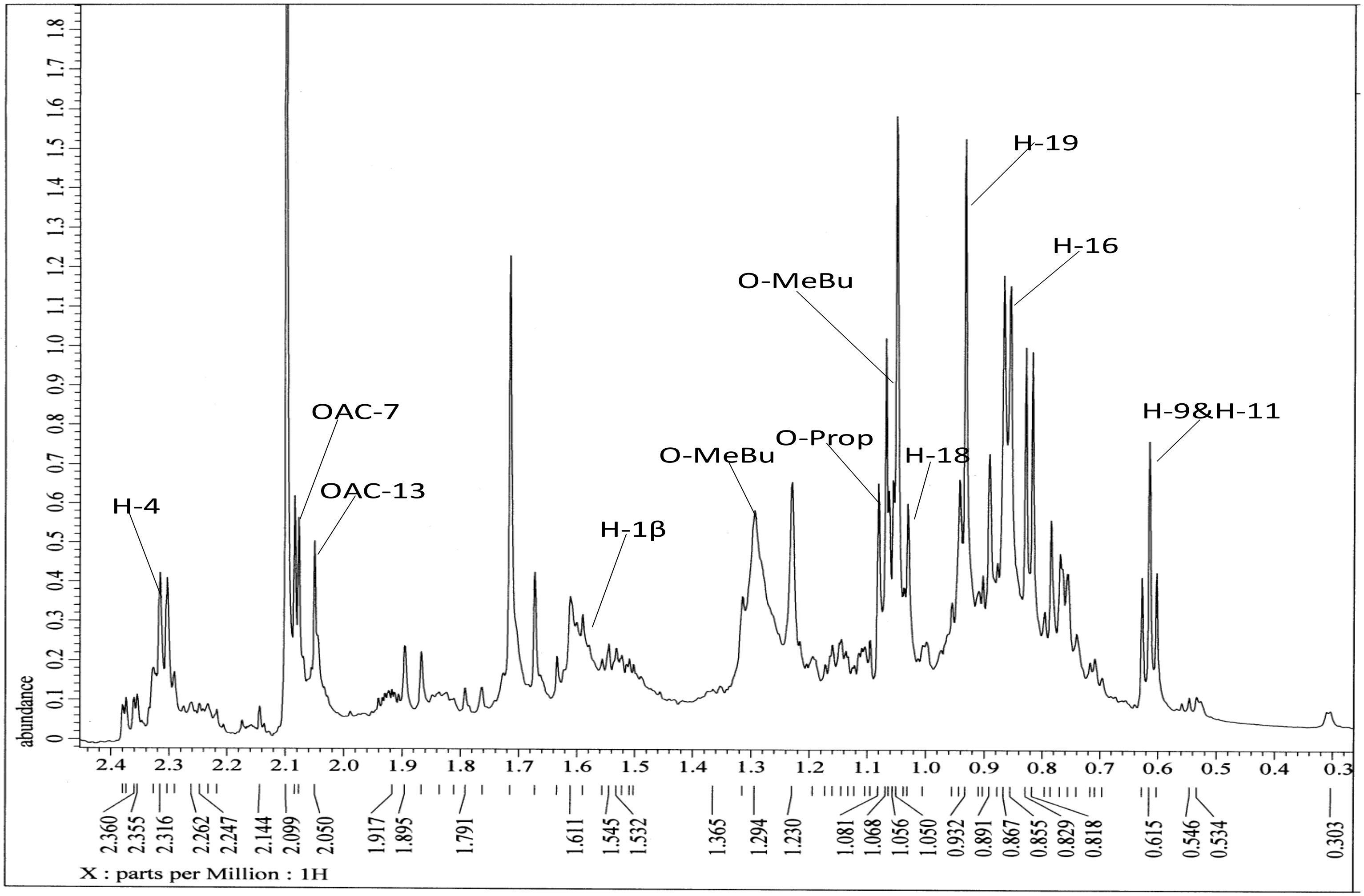

S29. ^{13}C NMR (150 MHz, $CDCl_3$) spectrum of **4**

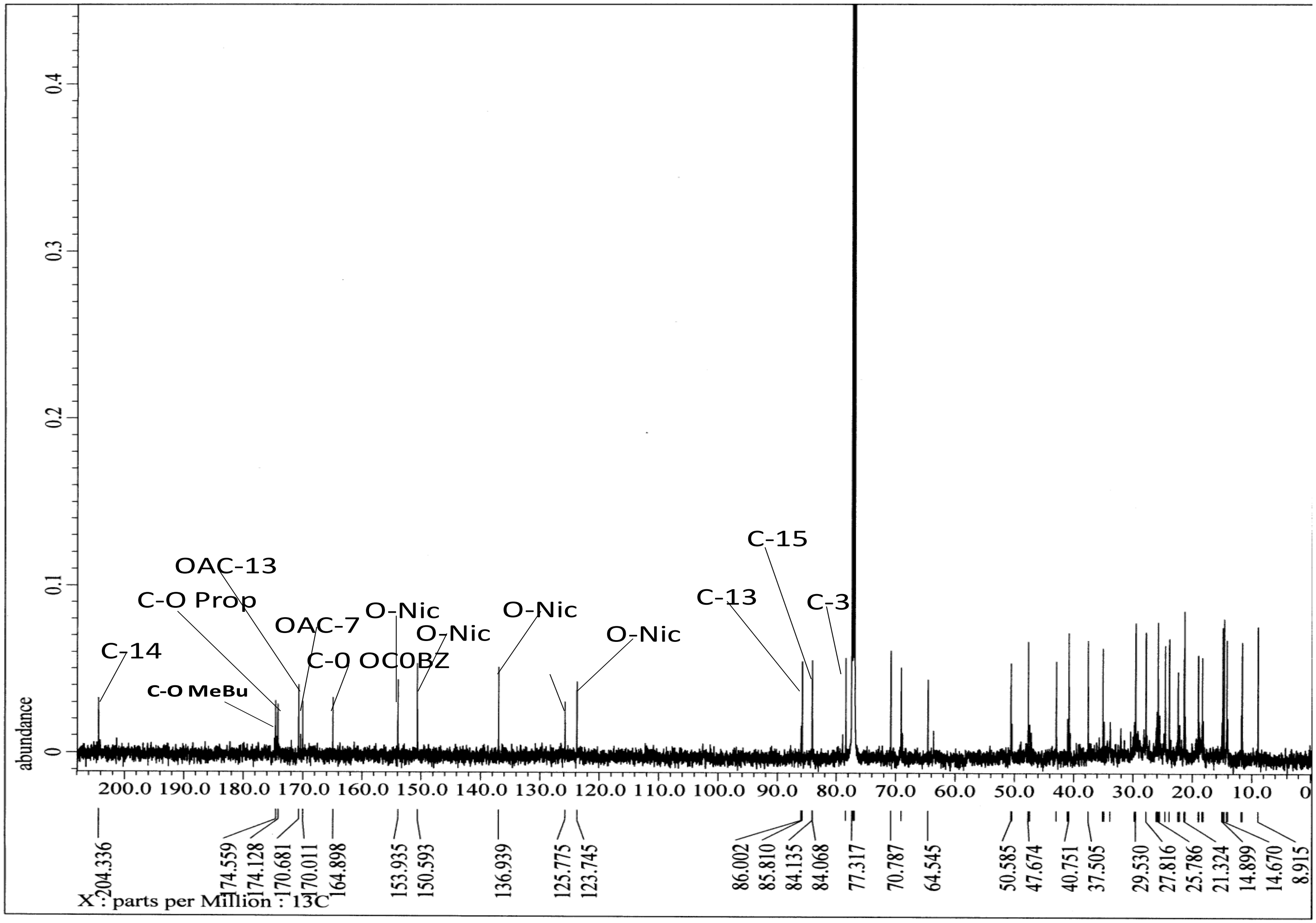

S29. ^{13}C NMR (150 MHz, $CDCl_3$) spectrum of **4**

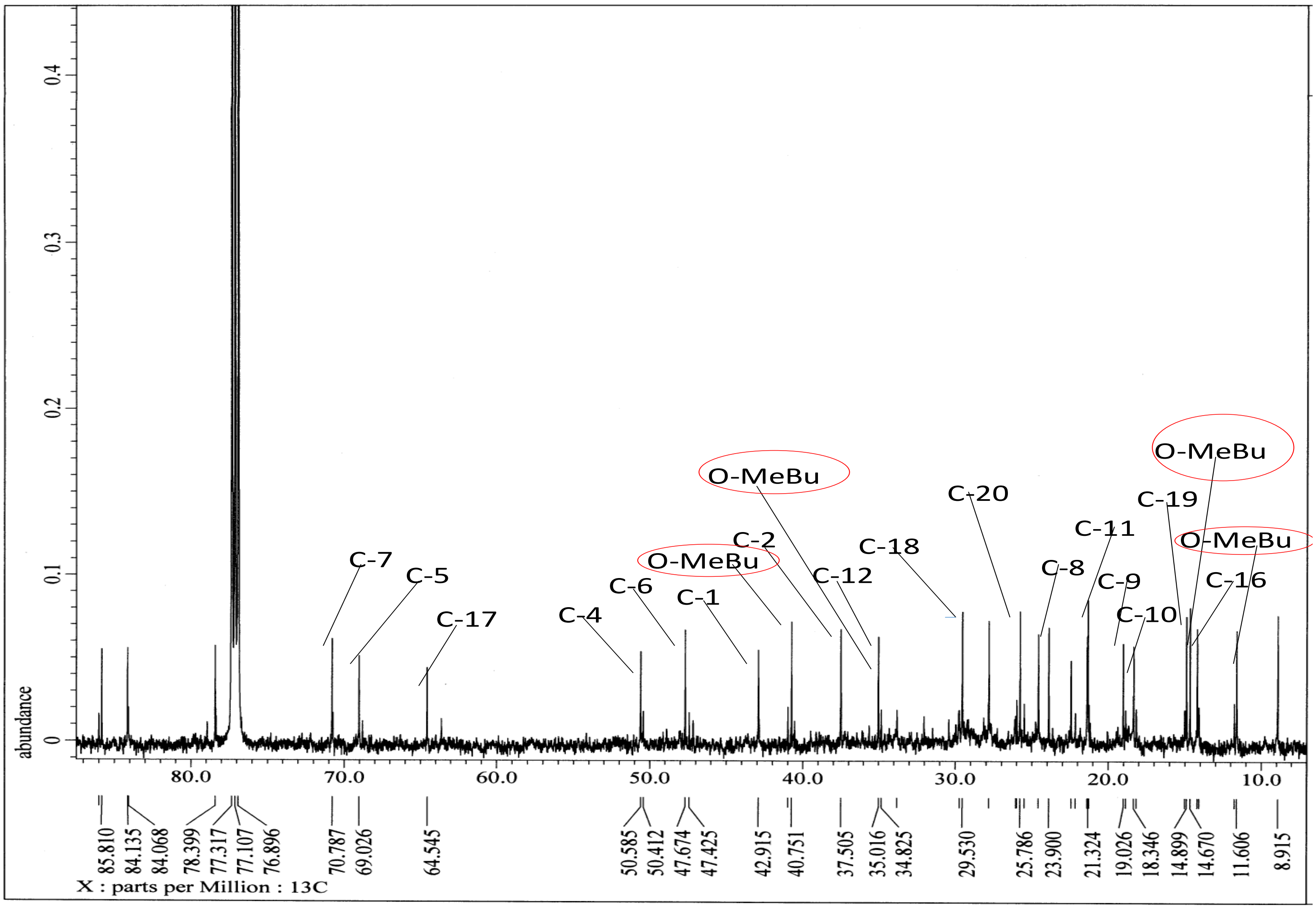

S30. DEPT (150 MHz, $CDCl_3$) spectra of **4**

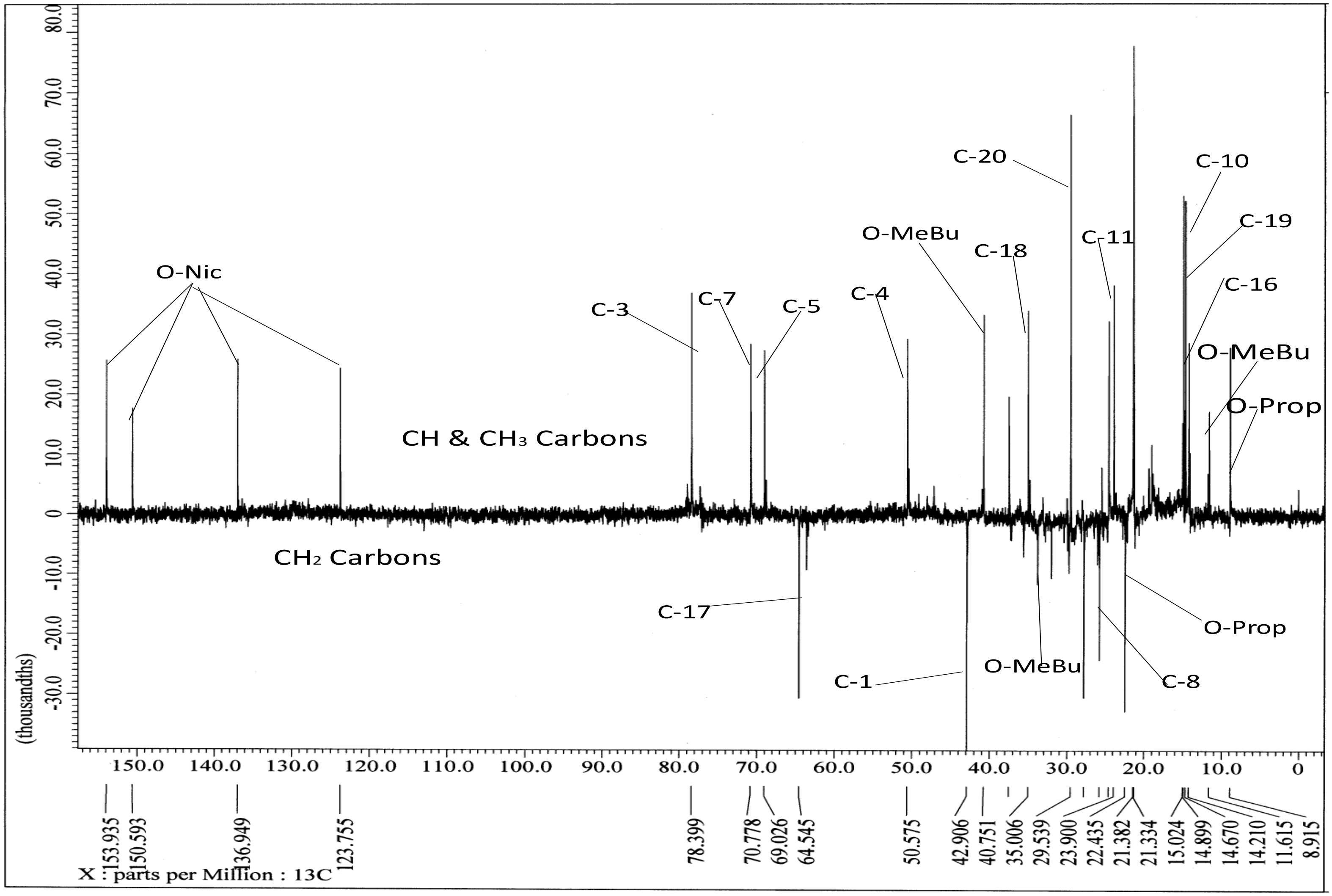

S31. ^1H ^1H COSY spectrum of **4** in $CDCl_3$

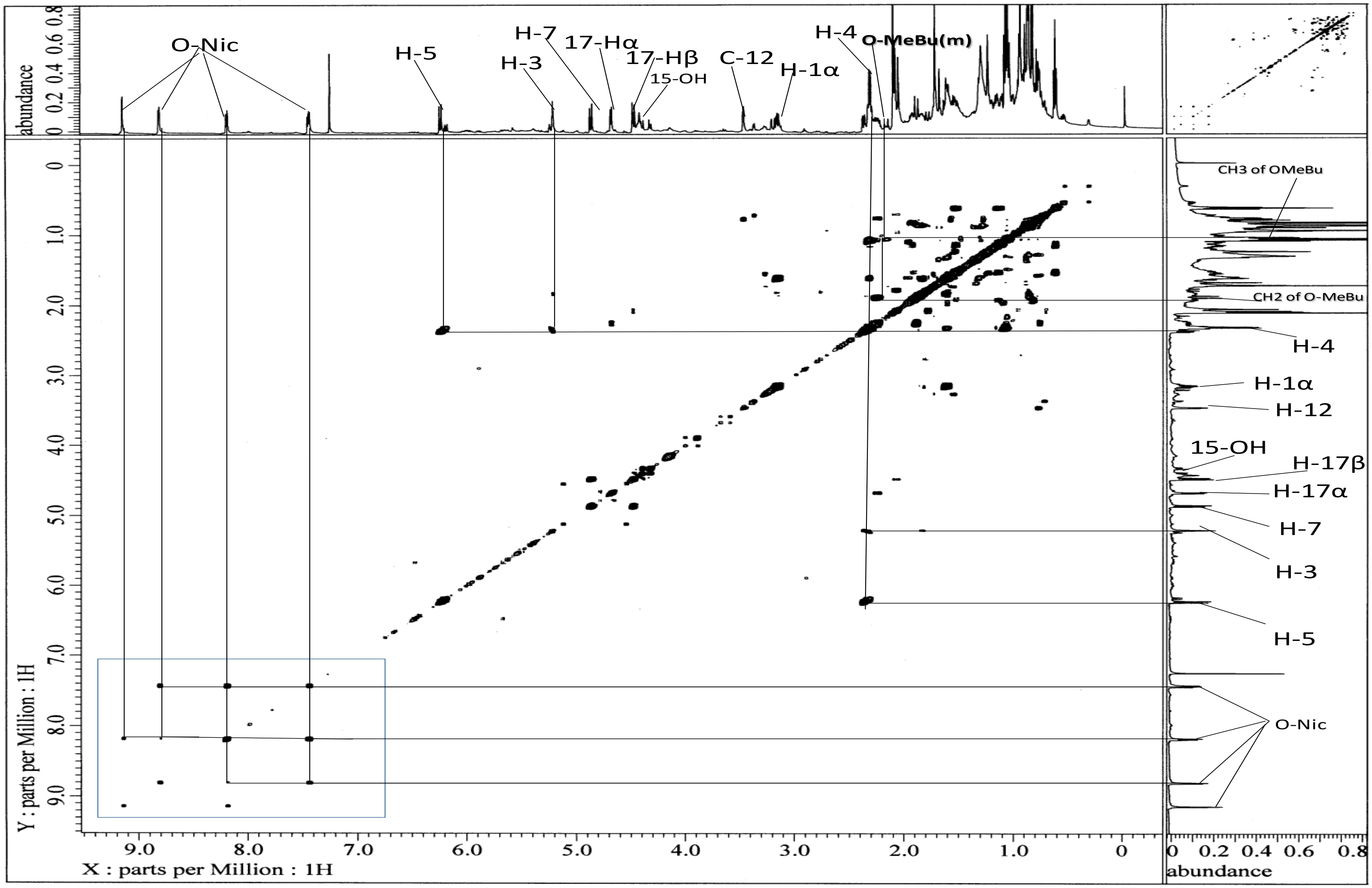

S32. HMBC spectrum of **4** in $CDCl_3$

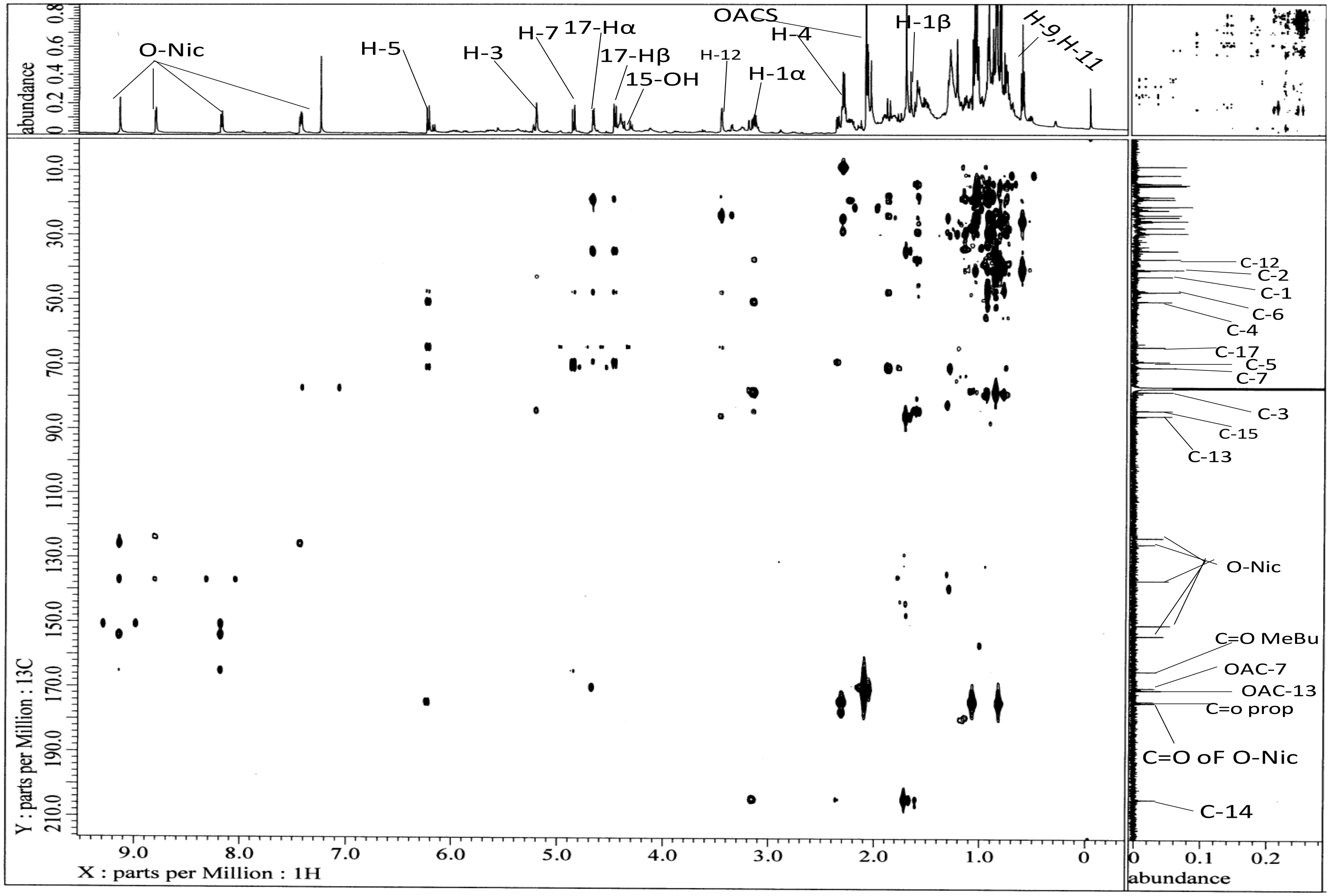

S32. HMBC spectrum of **4** in $CDCl_3$

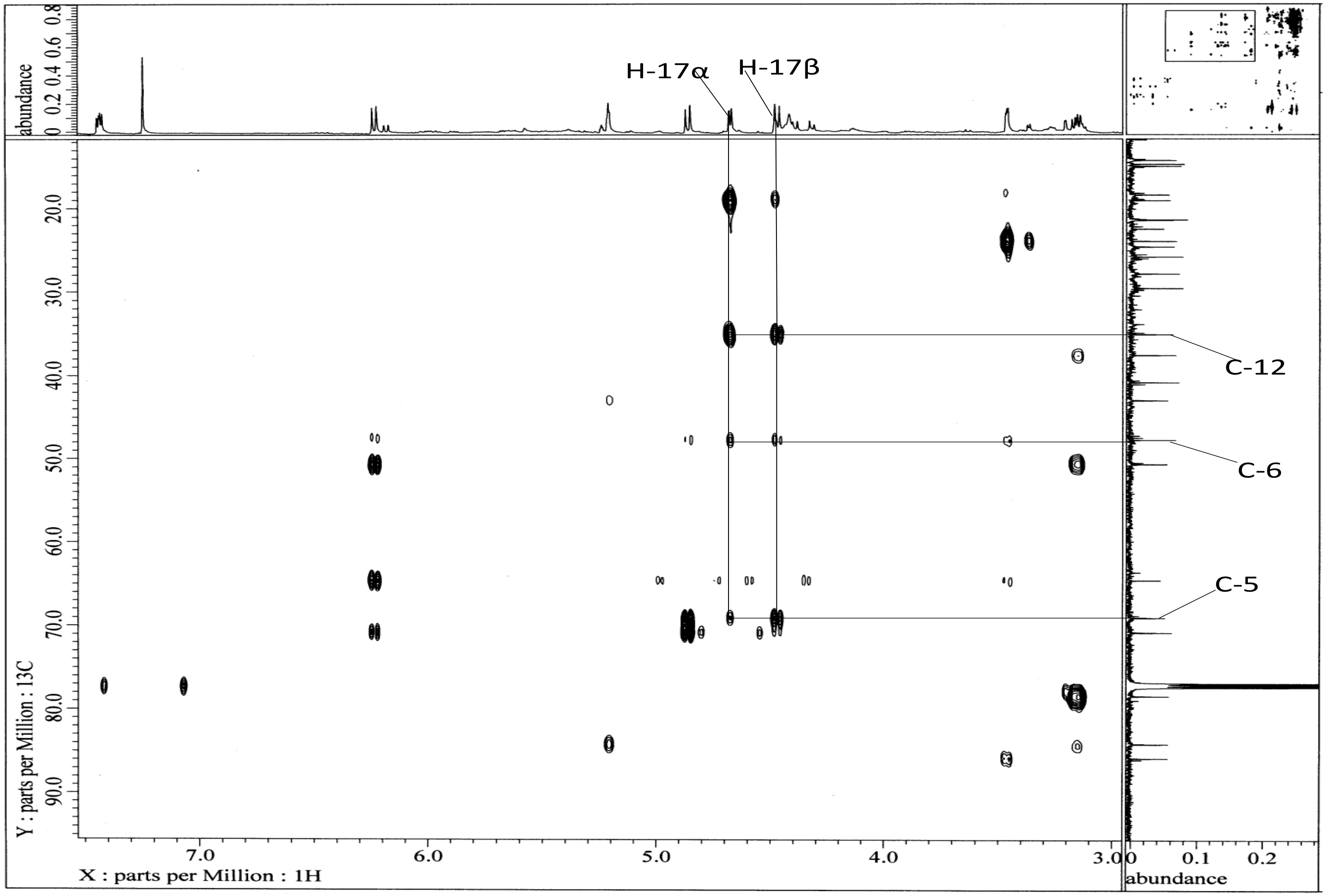

S33. HSQC spectrum of **4** in $CDCl_3$

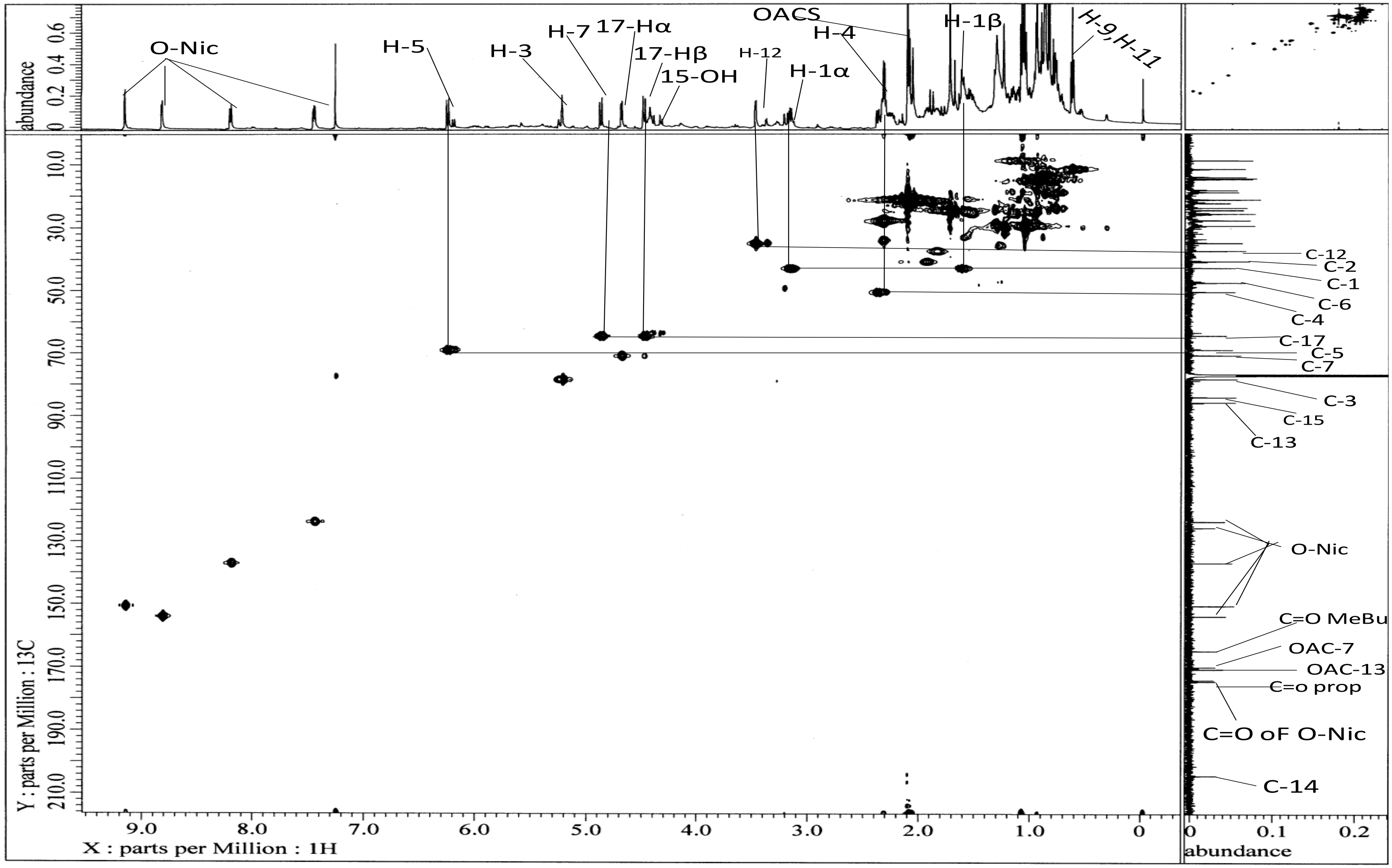

S34. NOESY spectrum of **4** in $CDCl_3$

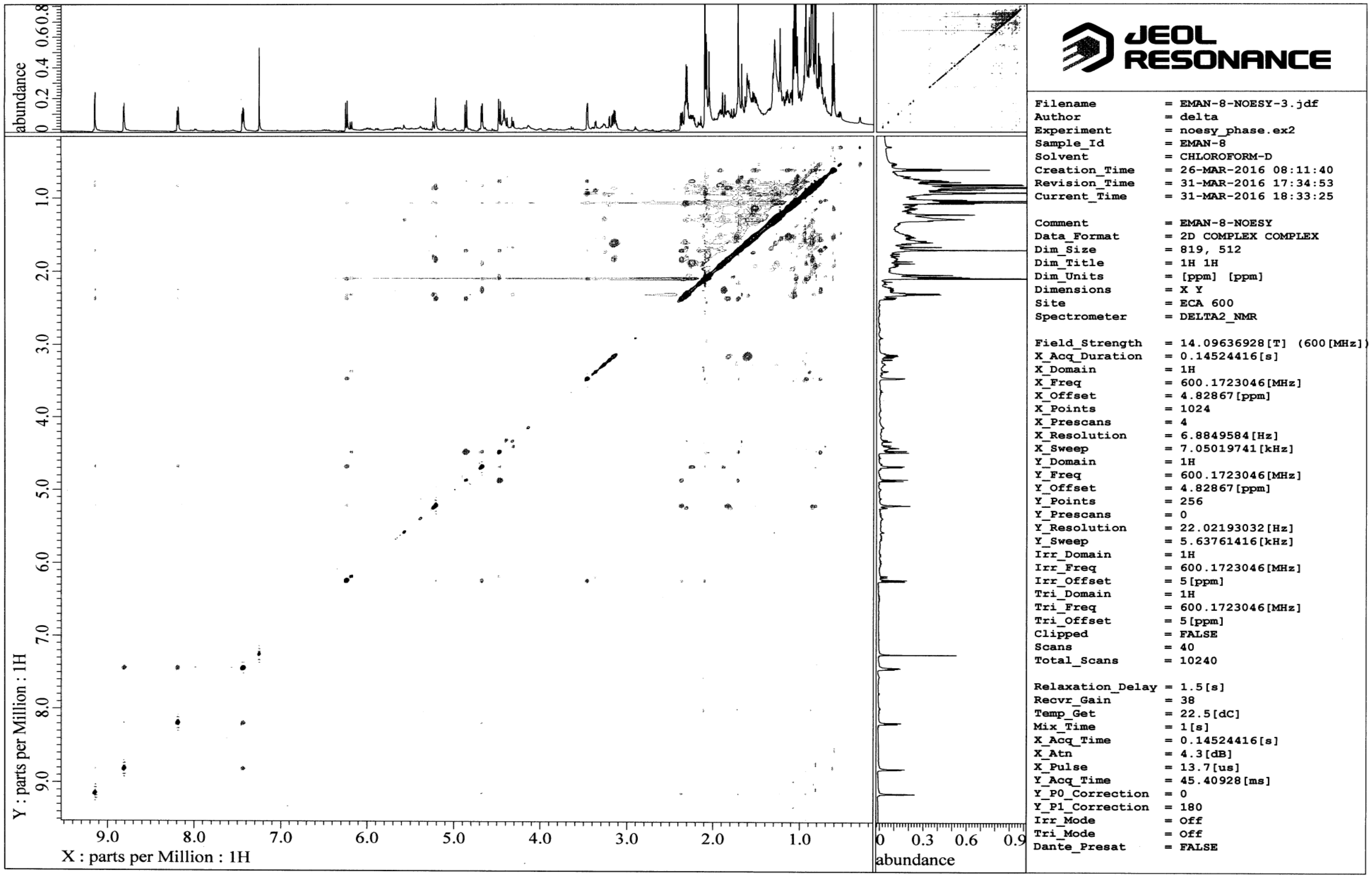

S35. LR-EI-MS of **4**

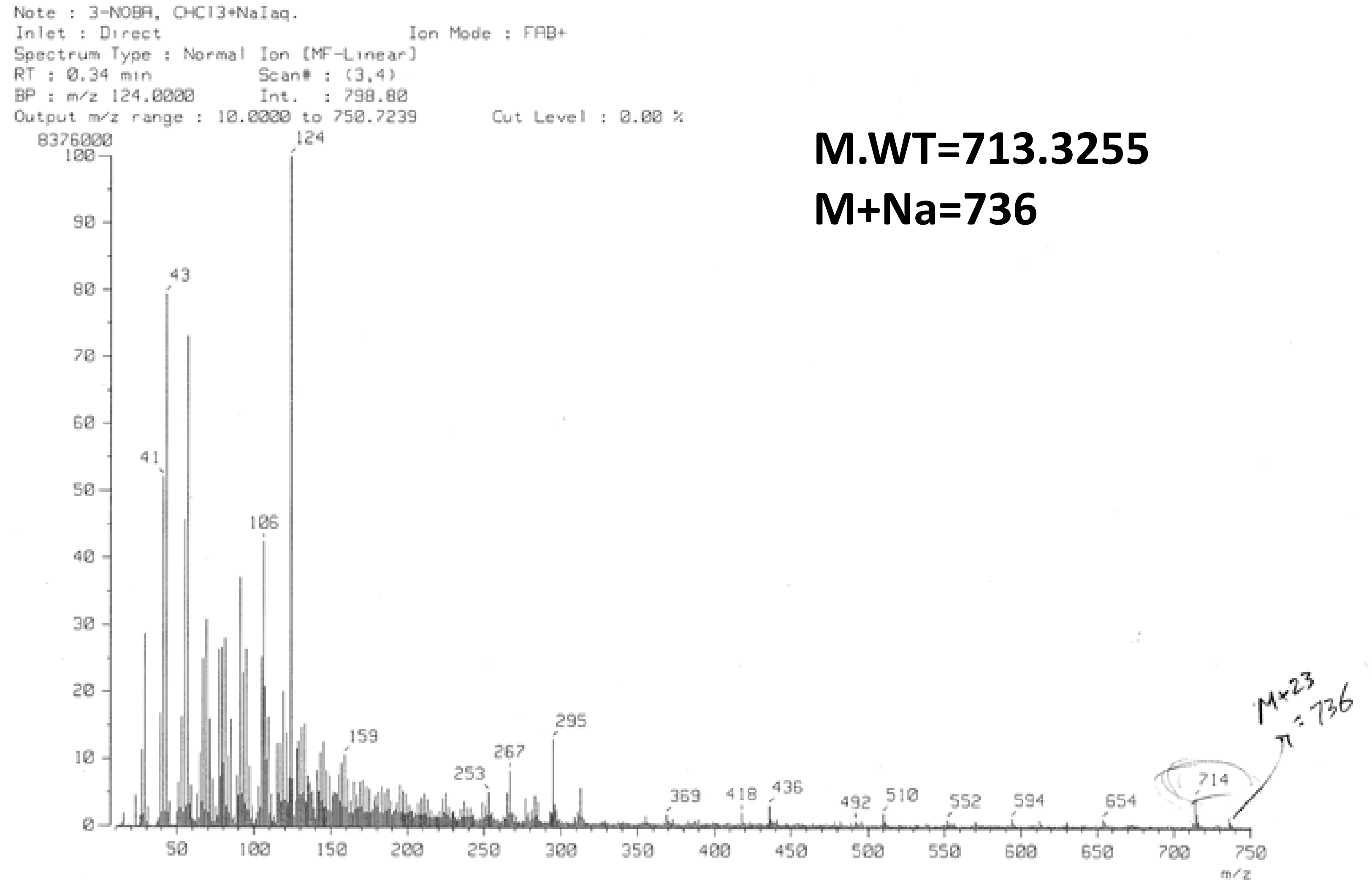

M.WT=713.3255

M+Na=736

S36. HR-EI-MS of **4**

```
Note : 3-NOBA, CHCl3+NaIaq.
Inlet : Direct                                  Ion Mode : FAB+
RT : 0.60 min                                   Scan#: 3
Elements : C 38/0, H 60/0, N 1/0, O 12/0, Na 1/0
Mass Tolerance       : 1000ppm, 3mmu if m/z < 3, 5mmu if m/z > 5
Unsaturation (U.S.) : -0.5 - 30.0

Observed m/z  Int%    Err[ppm / mmu]    U.S.   Composition
  736.3312    35.9      +0.4 /  +0.3    13.5   C 38 H 51 N O 12 Na
```

```
[ Theoretical Ion Distribution ]                                    Page: 1
Molecular Formula : C38 H51 O12 N Na
                       (m/z    736.3309, MW    736.8122, U.S. 13.5)
Base Peak :    736.3309, Averaged MW :    736.8066(a),    736.8073(w)

   m/z        INT.
 736.3309  100.0000 ************************************************************
 737.3342   43.0893 **************************
 738.3370   11.4534 *******
 739.3397    2.2692 *
 740.3424    0.3666
 741.3450    0.0505
 742.3475    0.0061
 743.3501    0.0007
```

S37. ^{1}H NMR (600 MHz, $CDCl_3$) spectrum of **5**

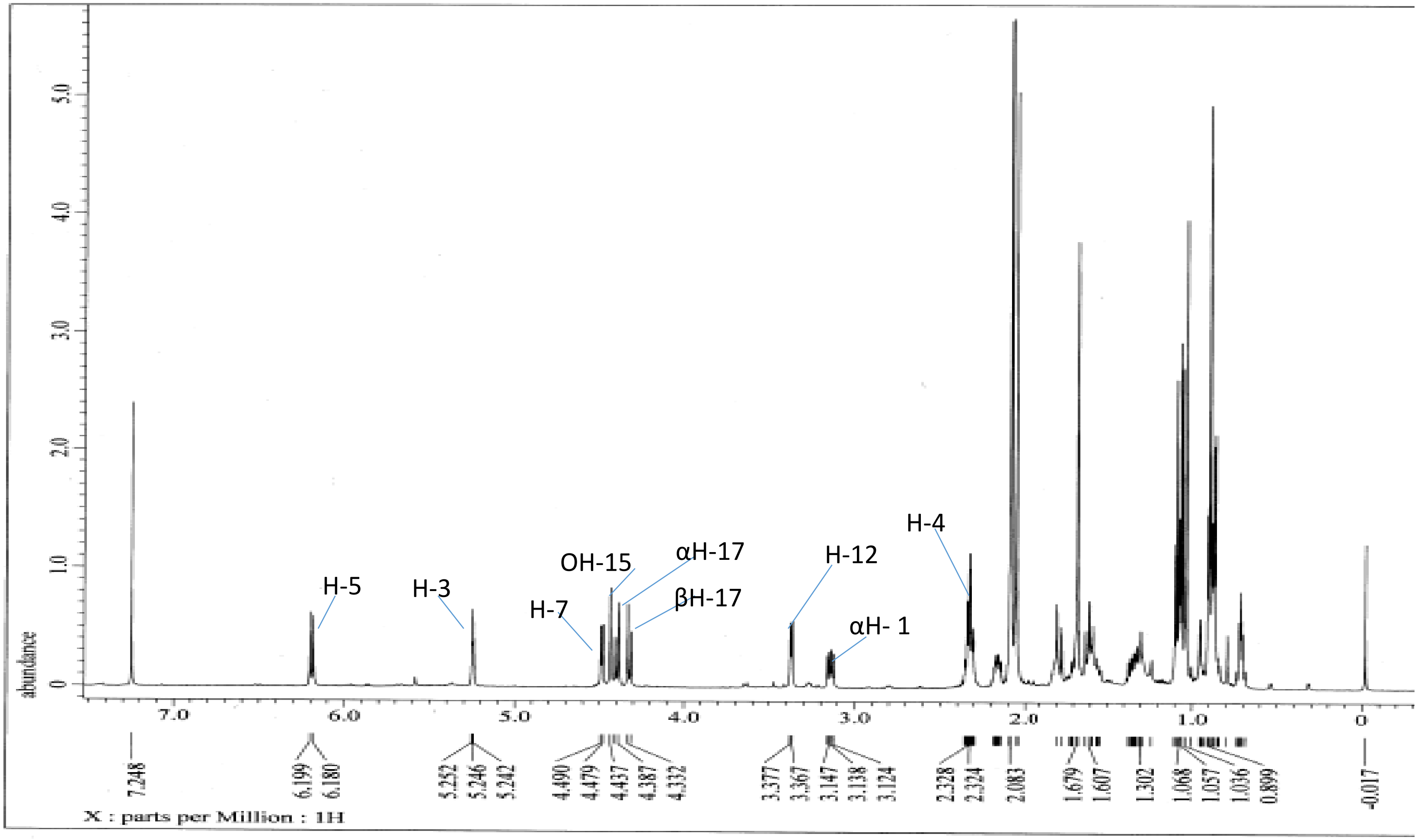

S38. ^{13}C NMR (150 MHz, $CDCl_3$) spectrum of **5**

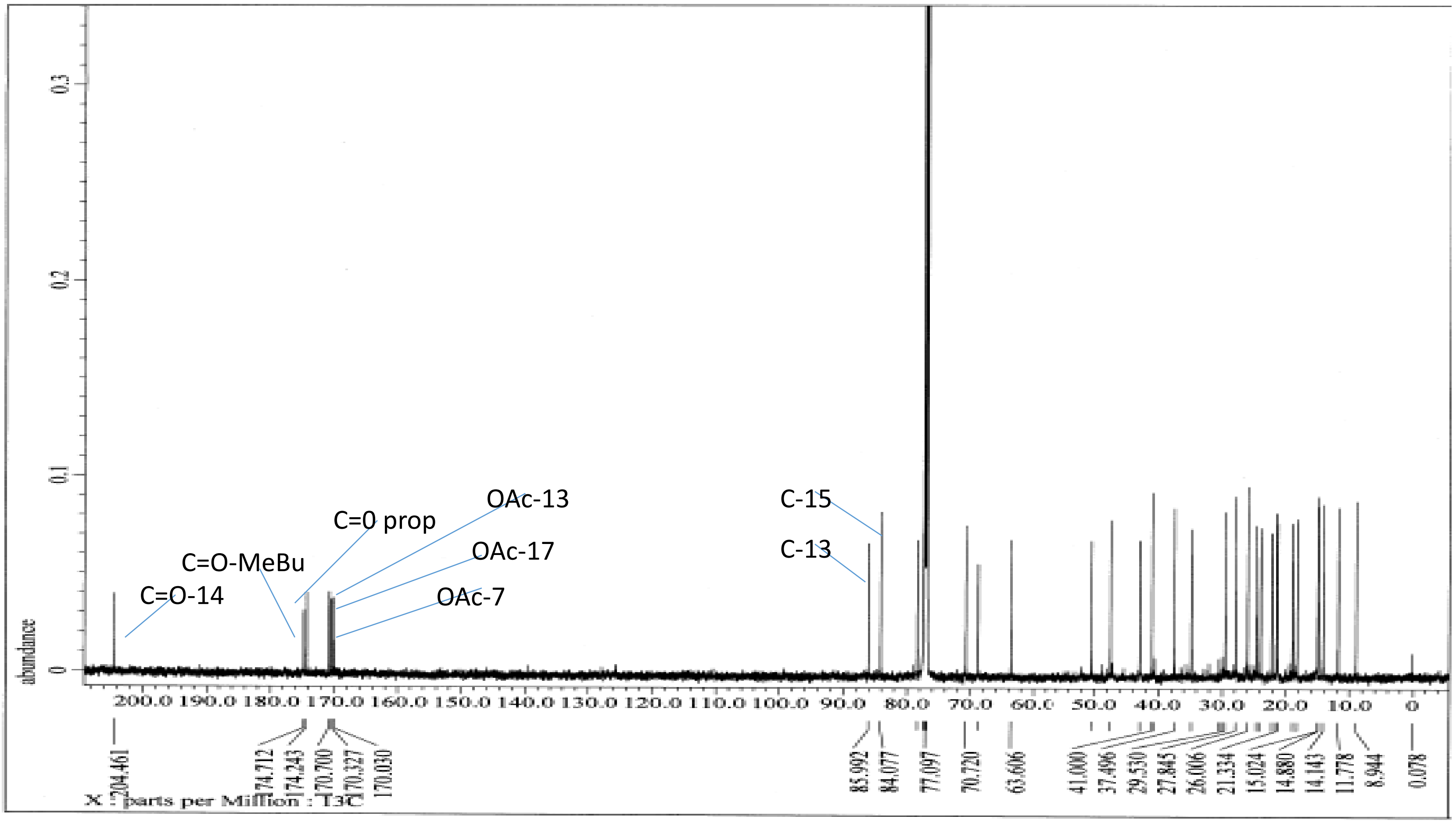

S39 . DEPT (150 MHz, $CDCl_3$) spectra of **5**

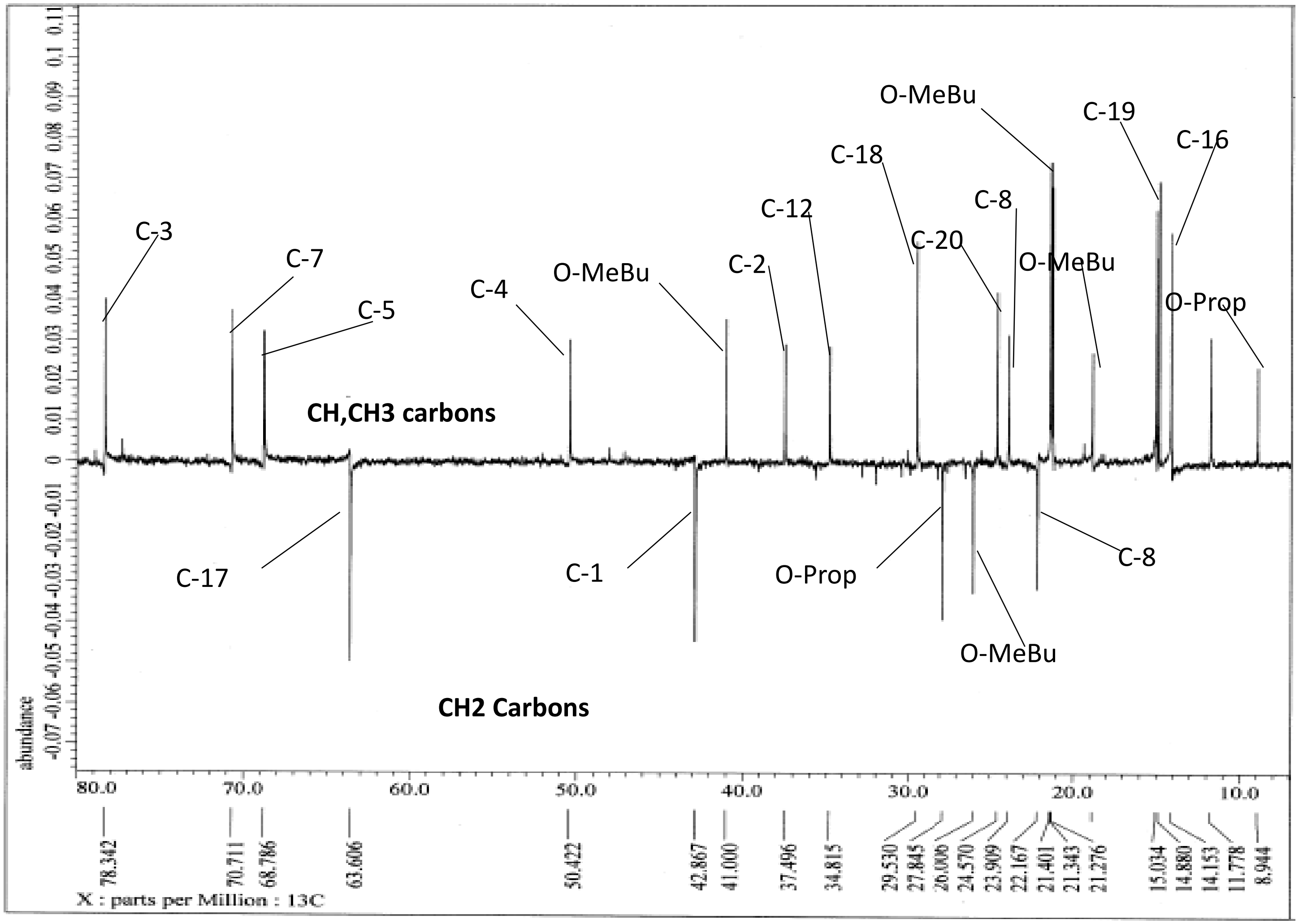

S40 ^{1}H ^{1}H COSY spectrum of **5** in $CDCl_3$

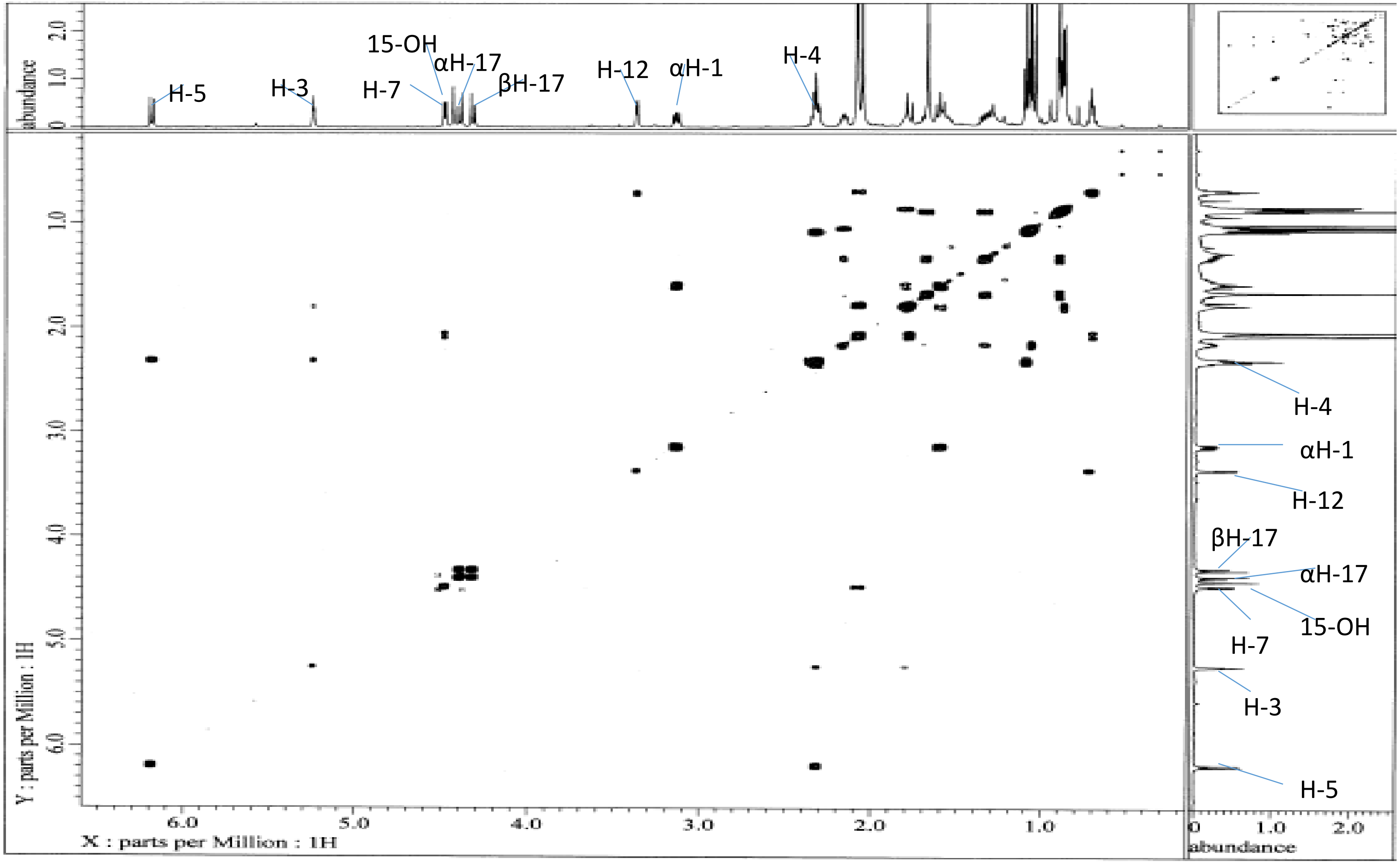

S40 ^1H ^1H COSY spectrum of **5** in $CDCl_3$

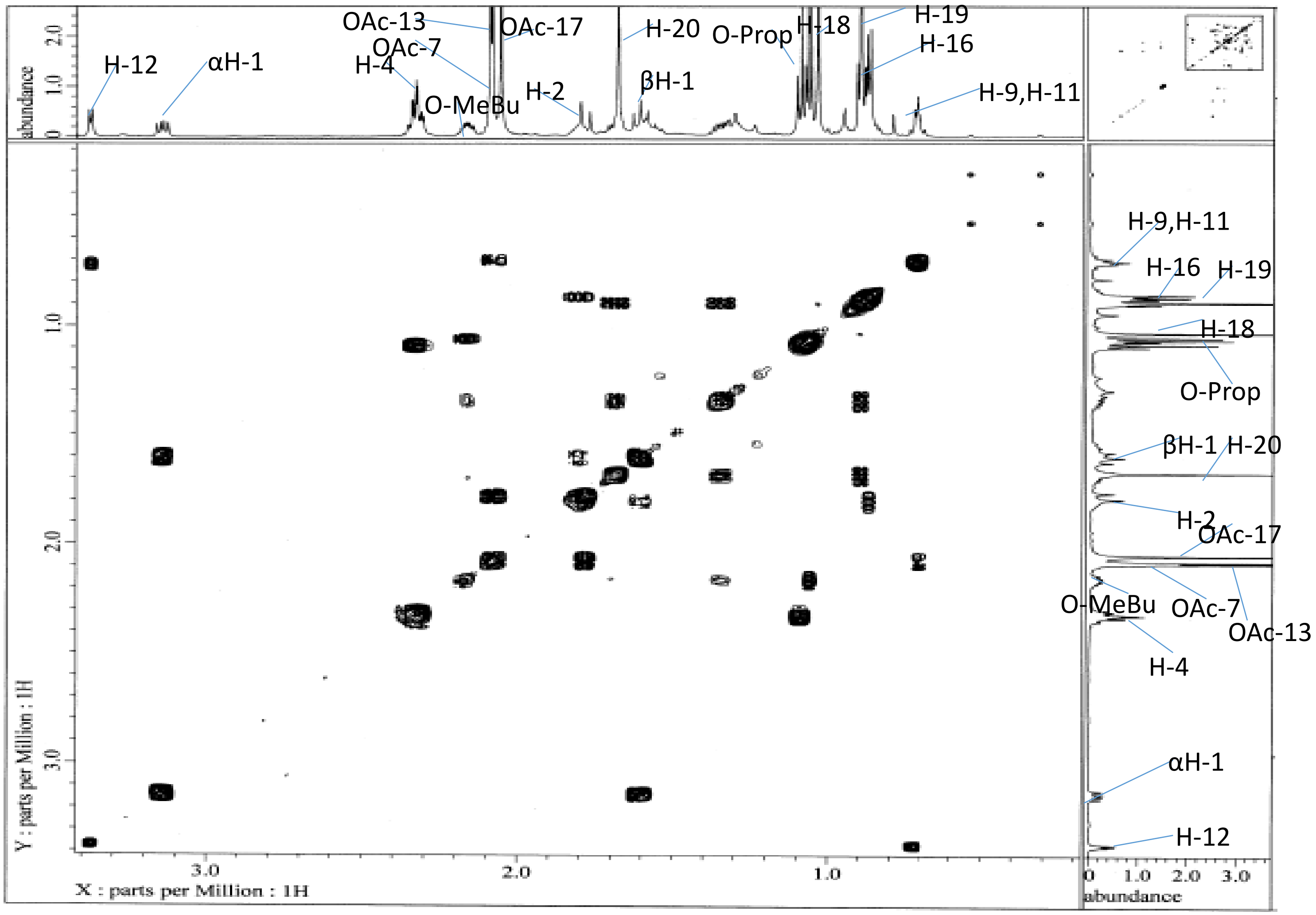

S41. HMBC spectrum of **5** in $CDCl_3$

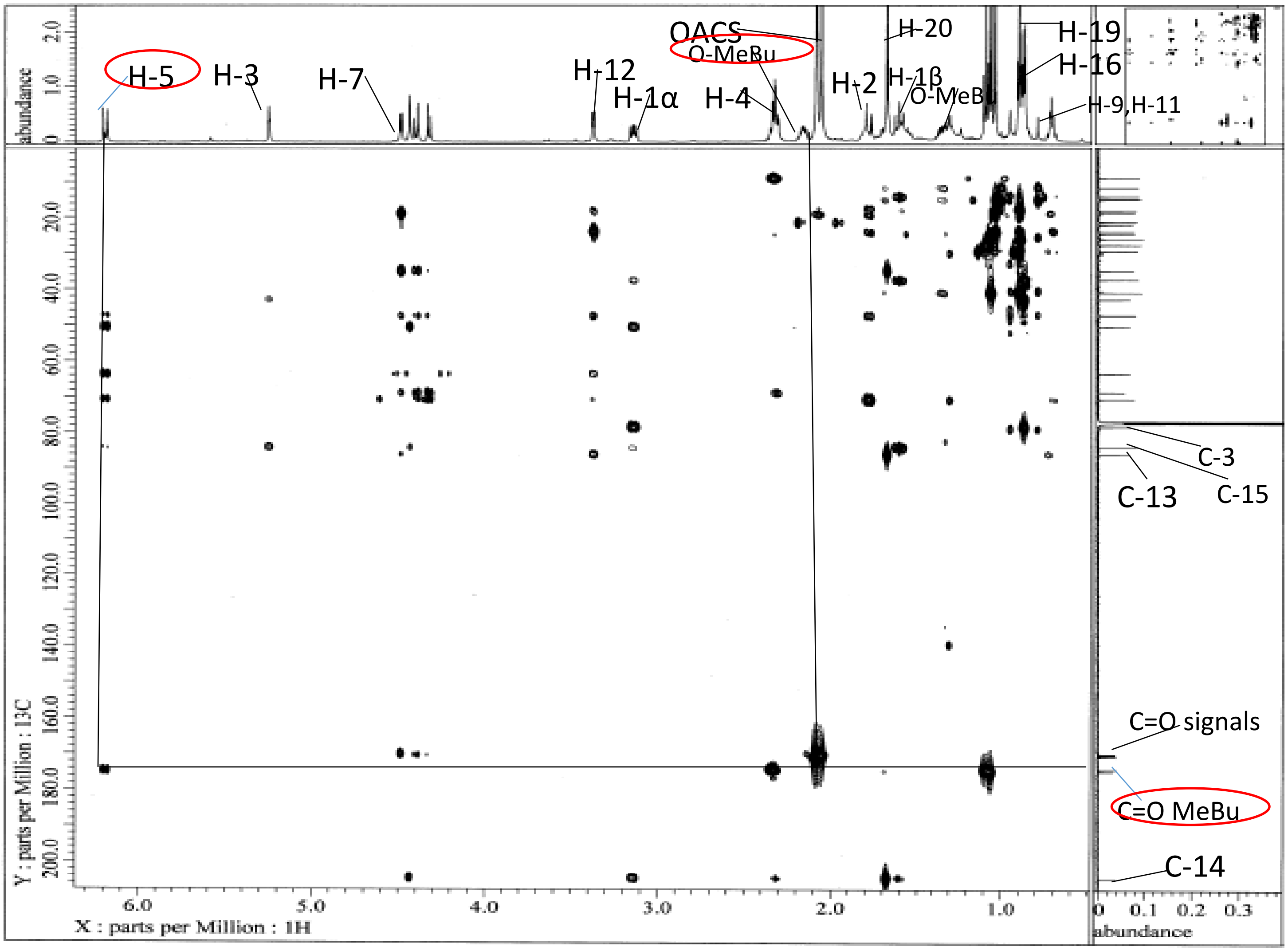

S41. HMBC spectrum of **5** in $CDCl_3$

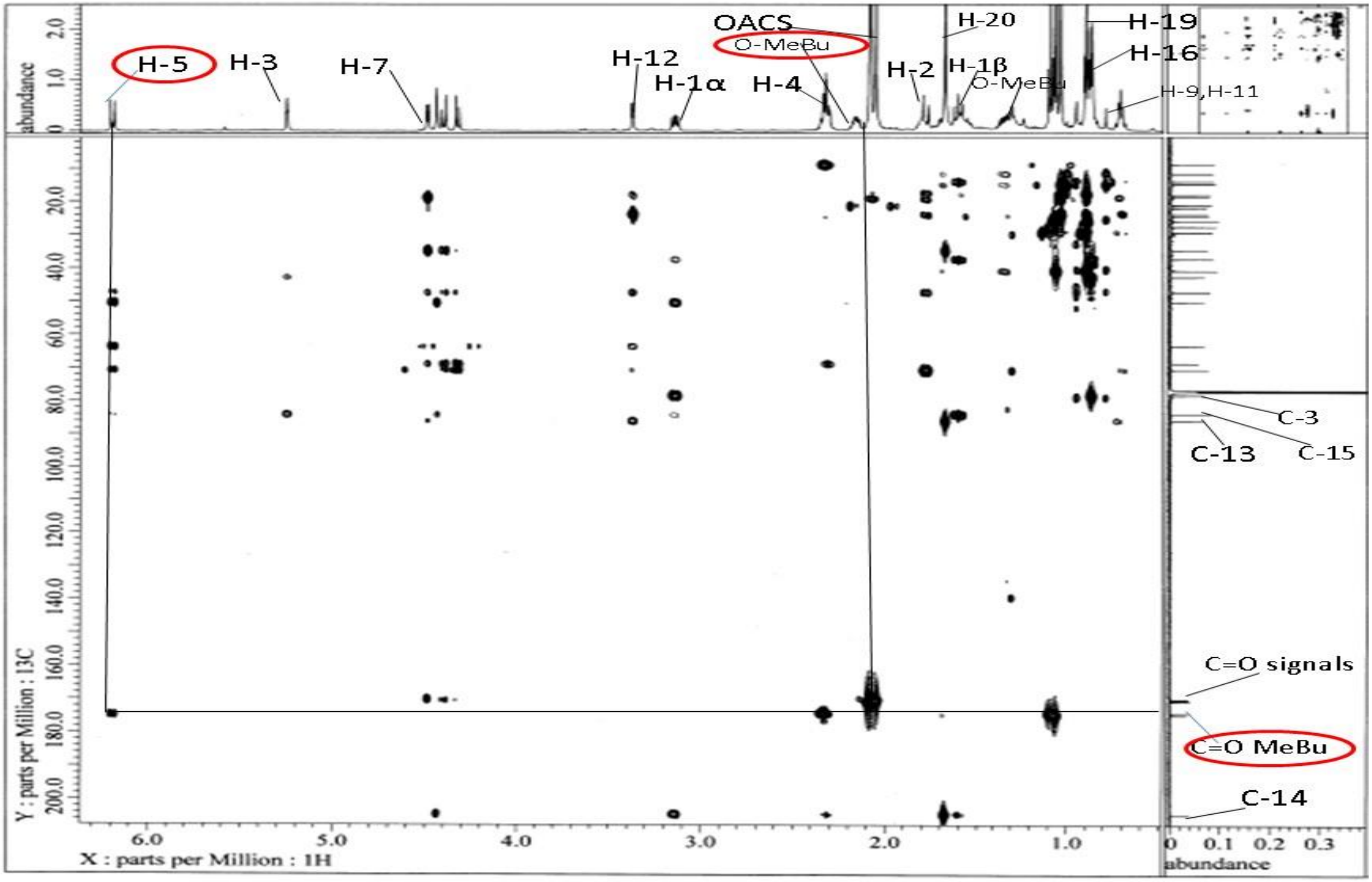

S41. HMBC spectrum of **5** in $CDCl_3$

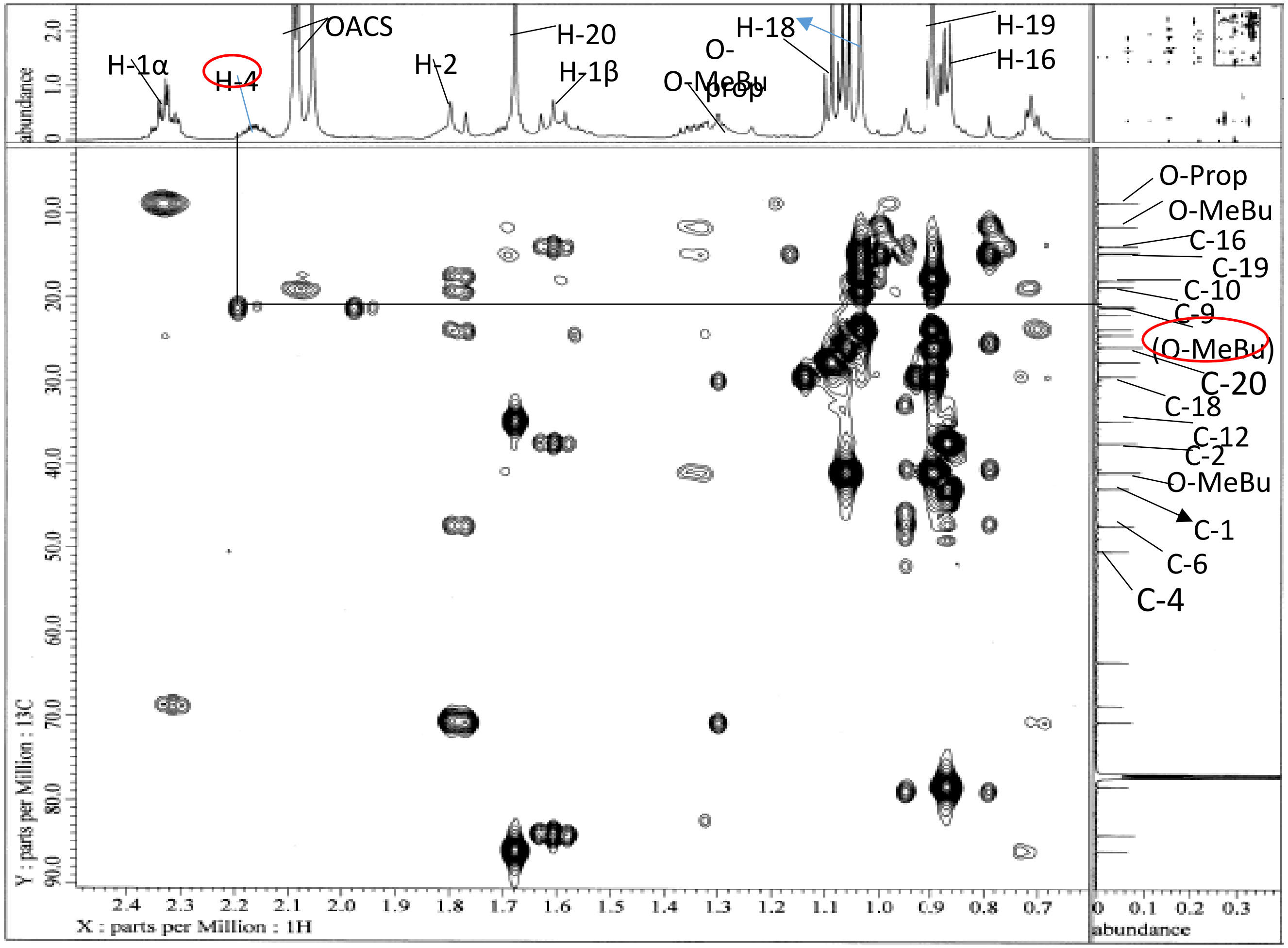

S41. HMBC spectrum of **5** in $CDCl_3$

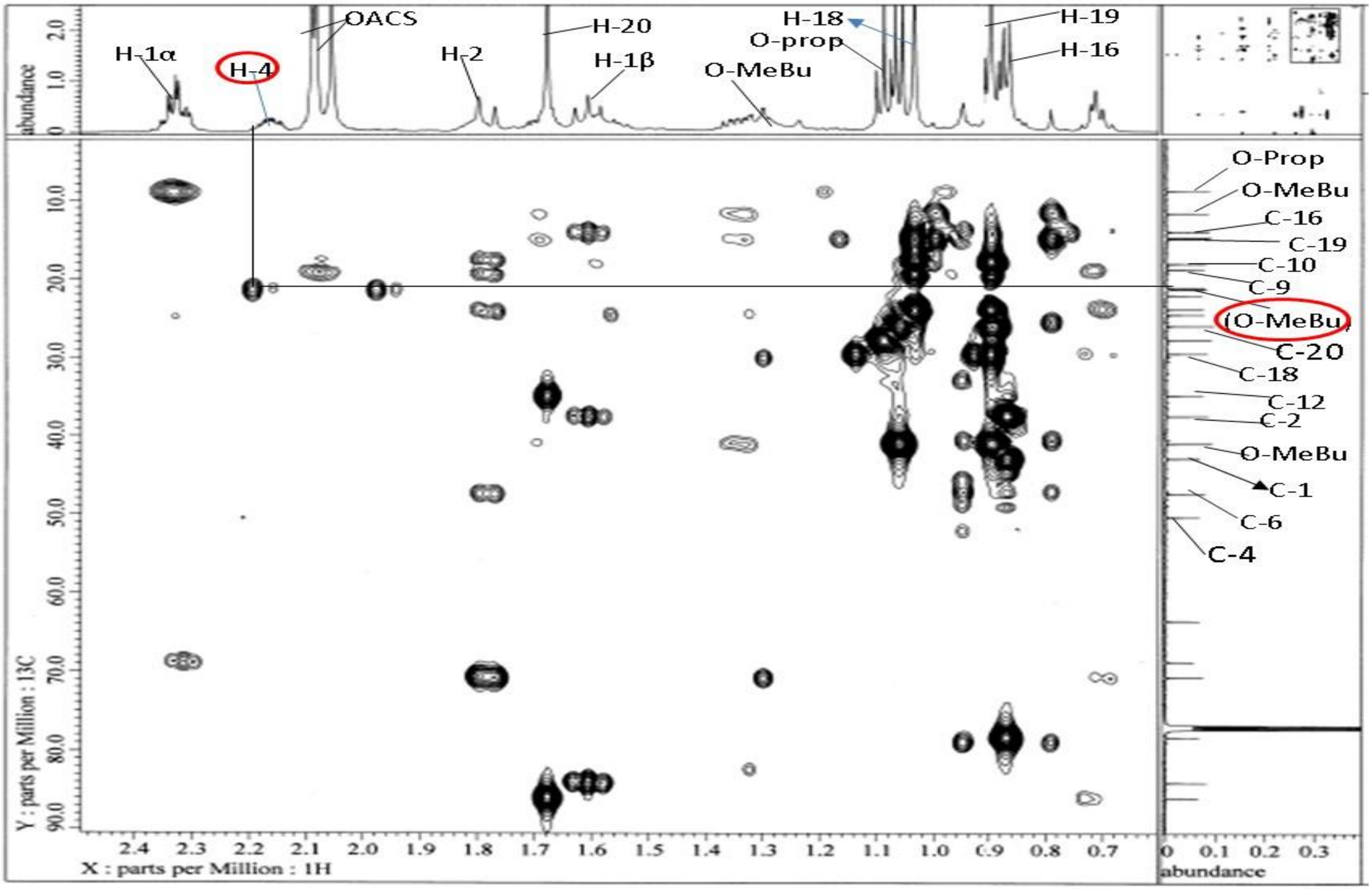

S42 HMQC spectrum of **5** in $CDCl_3$

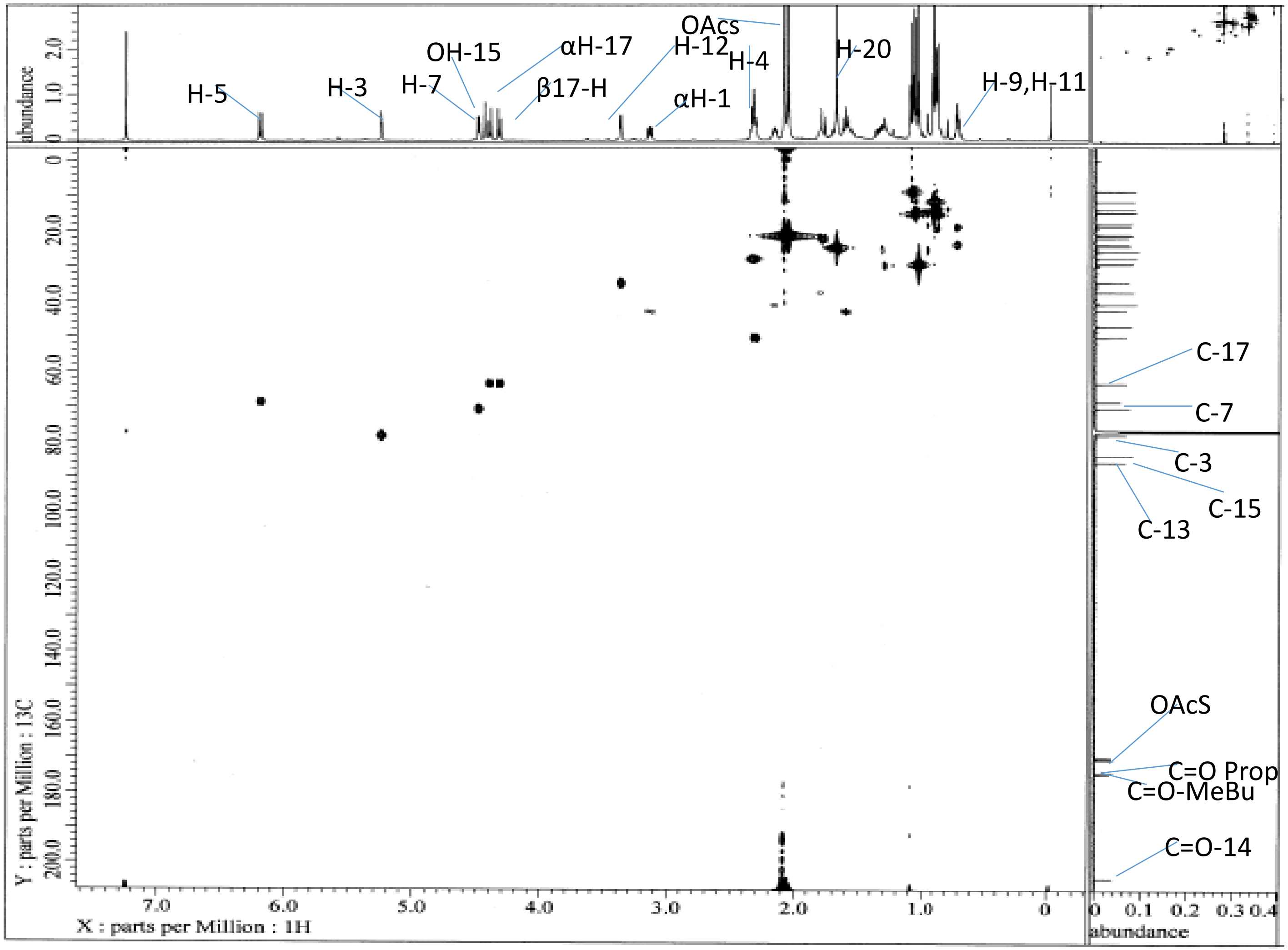

S42 HMQC spectrum of **5** in $CDCl_3$

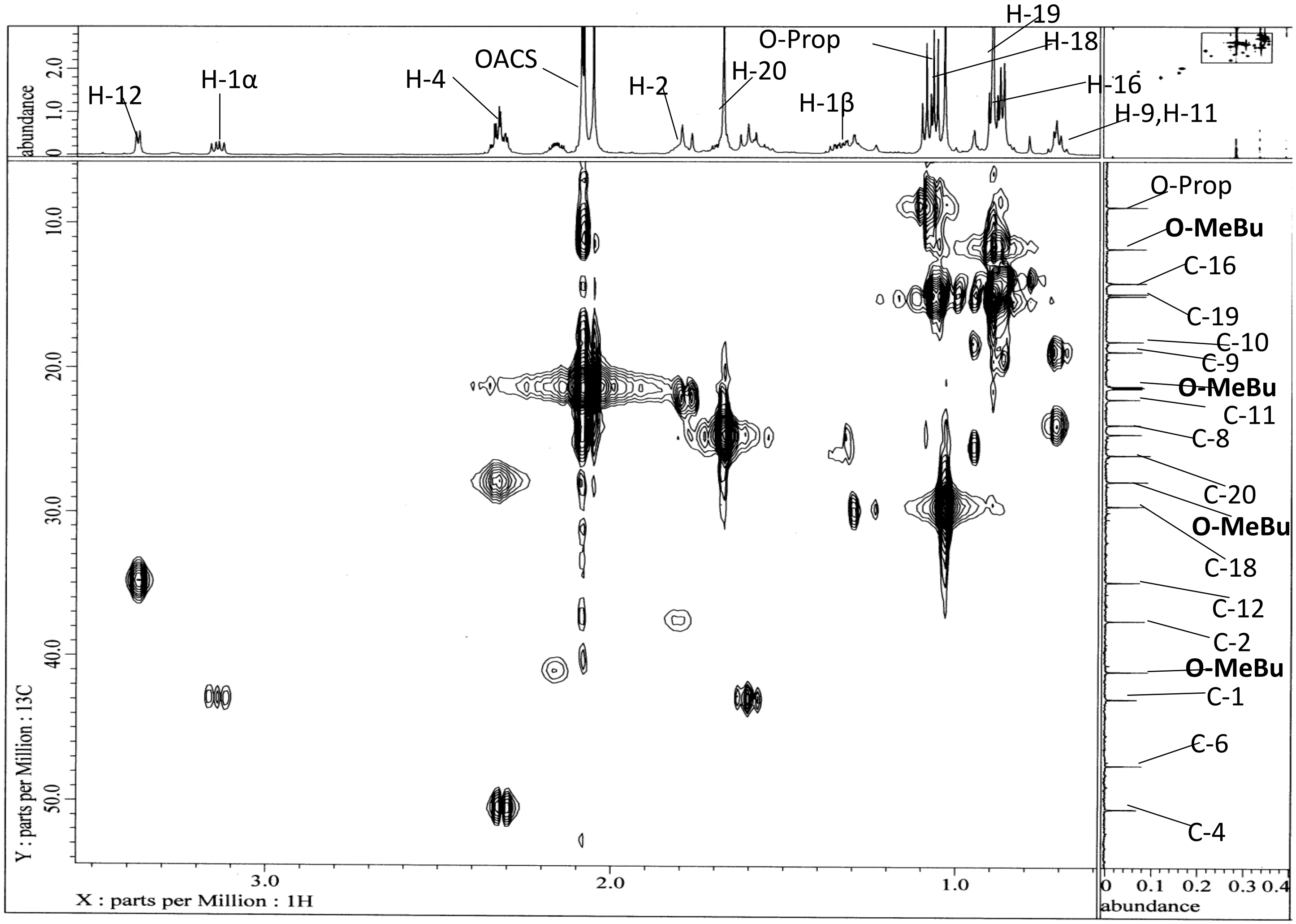

S43. NOESY spectrum of **5** in $CDCl_3$

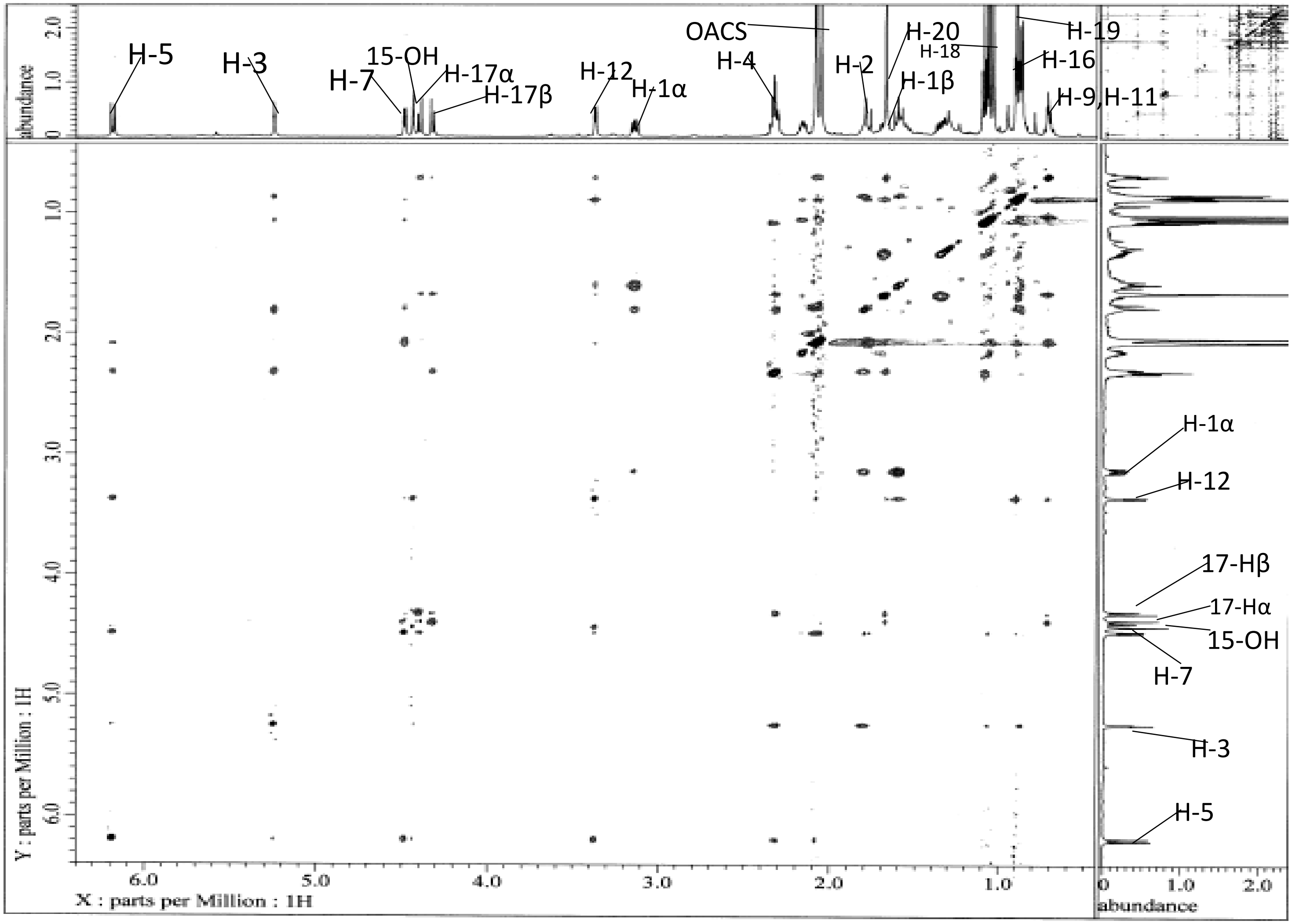

S44. LR-EI-MS of **5**

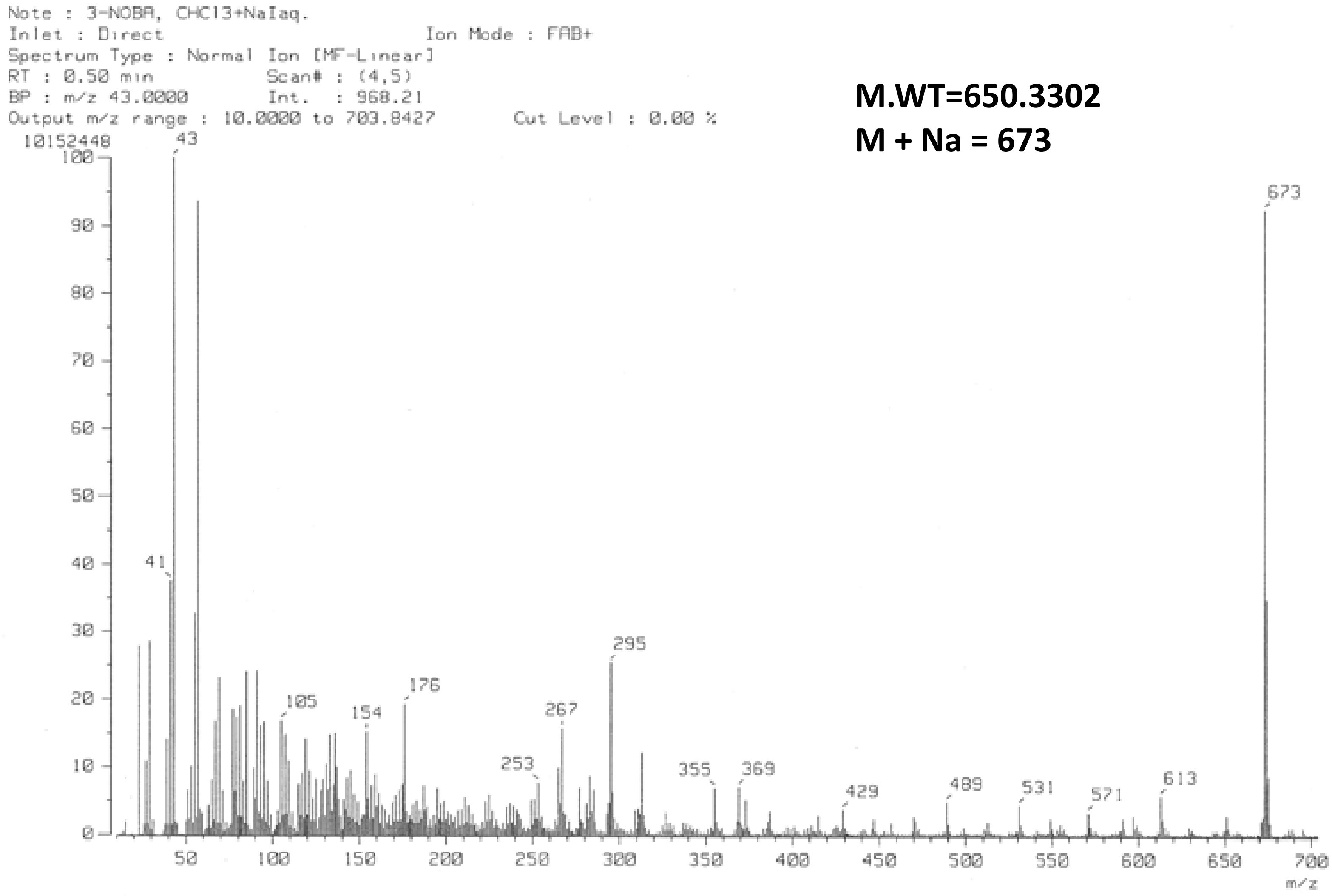

S45. HR-EI-MS of **5**

Note : 3-NOBA, CHCl3+NaIaq.
Inlet : Direct   Ion Mode : FAB+
RT : 0.00 min   Scan#: 1
Elements : C 34/0, H 60/0, O 12/0, Na 1/0
Mass Tolerance : 1000ppm, 3mmu if m/z < 3, 5mmu if m/z > 5
Unsaturation (U.S.) : -0.5 - 30.0

Observed m/z	Int%	Err[ppm / mmu]	U.S.	Composition
673.3206	100.0	+0.9 / +0.6	9.5	C 34 H 50 O 12 Na

[Theoretical Ion Distribution]   Page: 1
Molecular Formula : C34 H50 O12 Na
(m/z 673.3200, MW 673.7536, U.S. 9.5)
Base Peak : 673.3200, Averaged MW : 673.7479(a), 673.7486(w)

m/z	INT.	
673.3200	100.0000	**
674.3234	38.2731	***********************
675.3261	9.5195	******
676.3288	1.7753	*
677.3314	0.2721	
678.3340	0.0357	
679.3365	0.0041	
680.3390	0.0004	

S46. ^{1}H NMR (600 MHz, $CDCl_3$) spectrum of **6**

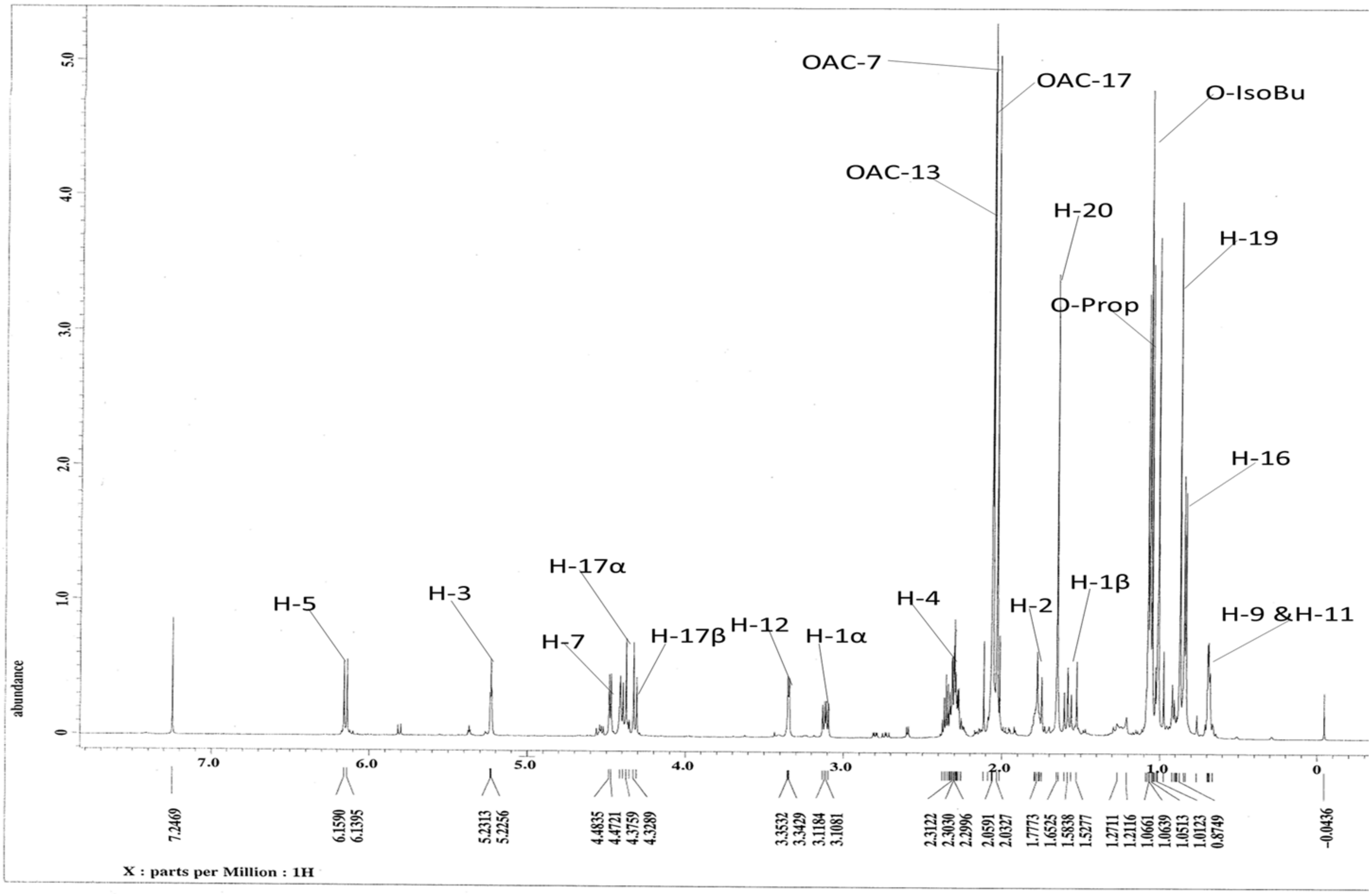

S47. ^{13}C NMR (150 MHz, $CDCl_3$) spectrum of **6**

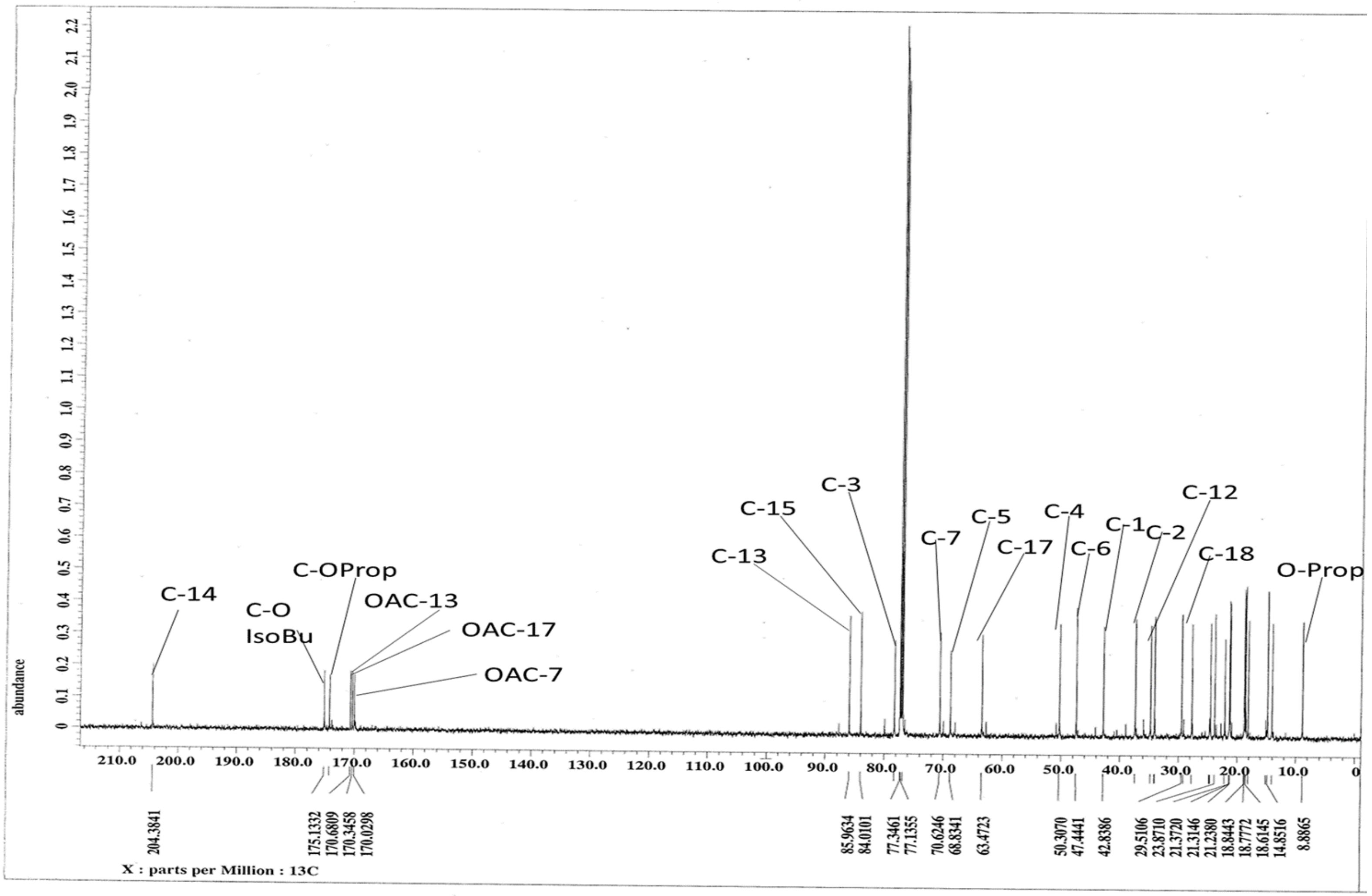

S47. ^{13}C NMR (150 MHz, $CDCl_3$) spectrum of **6**

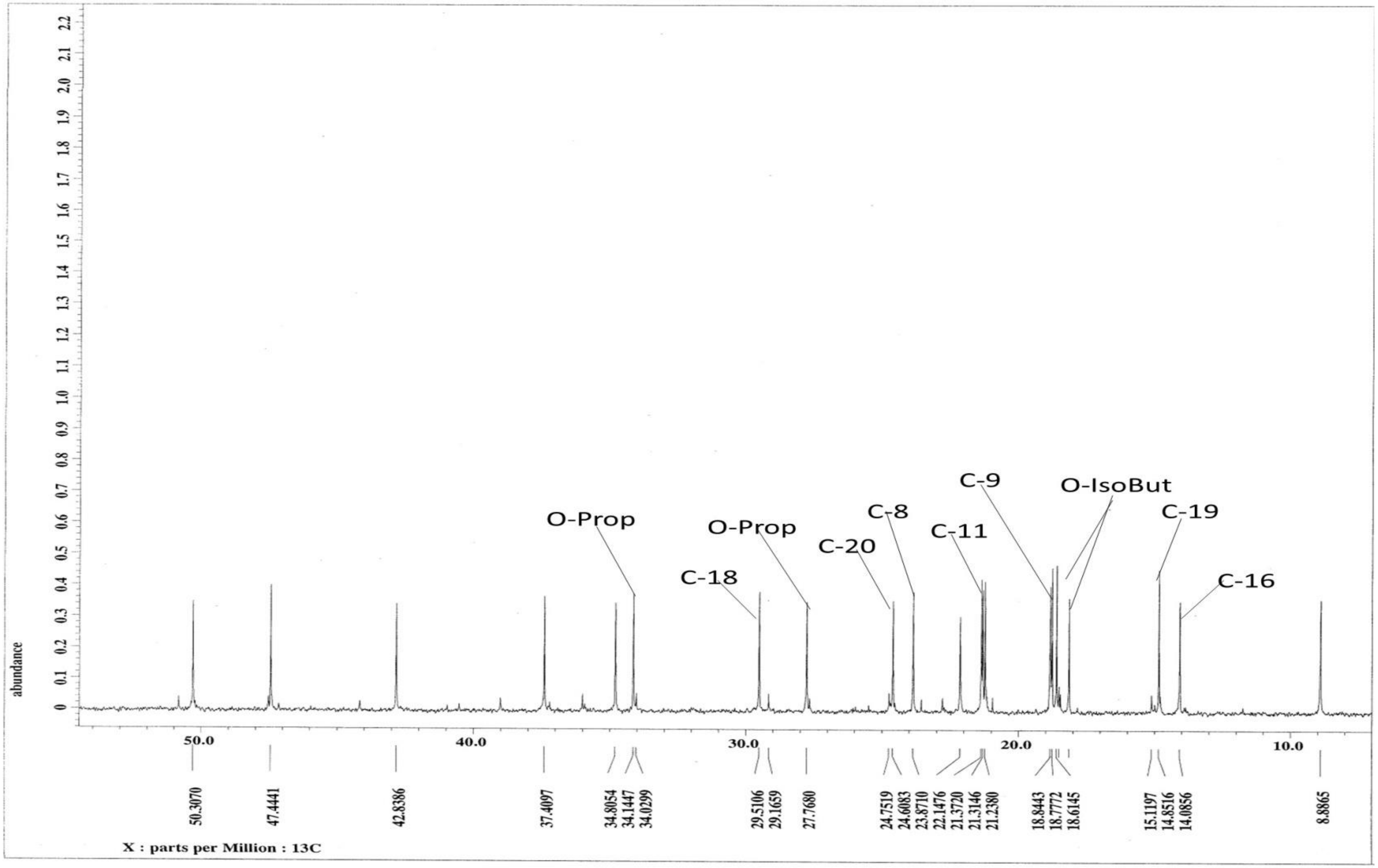

S48. DEPT (150 MHz, $CDCl_3$) spectra of **6.**

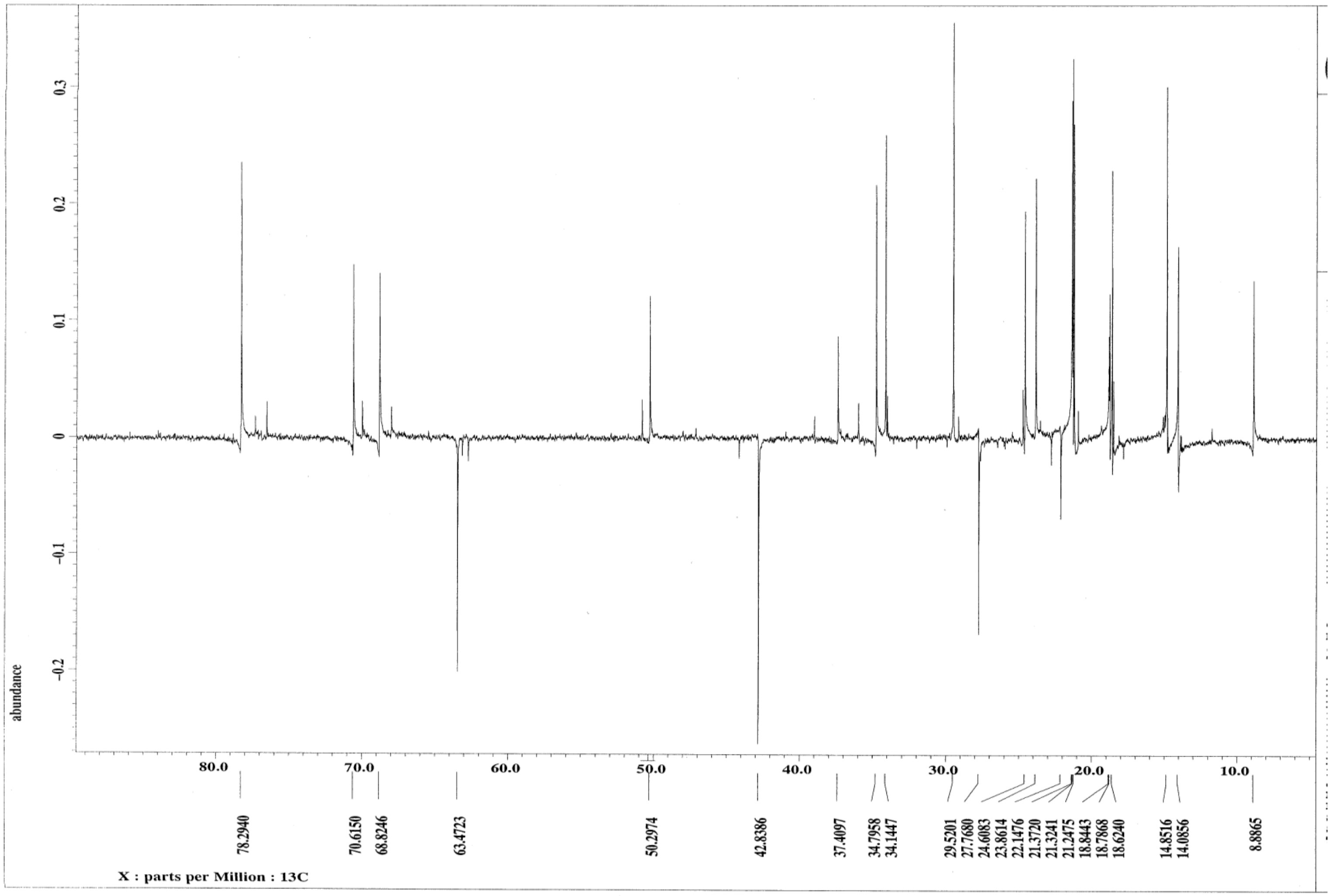

S49. ^1H ^1H COSY spectrum of **6** in $CDCl_3$

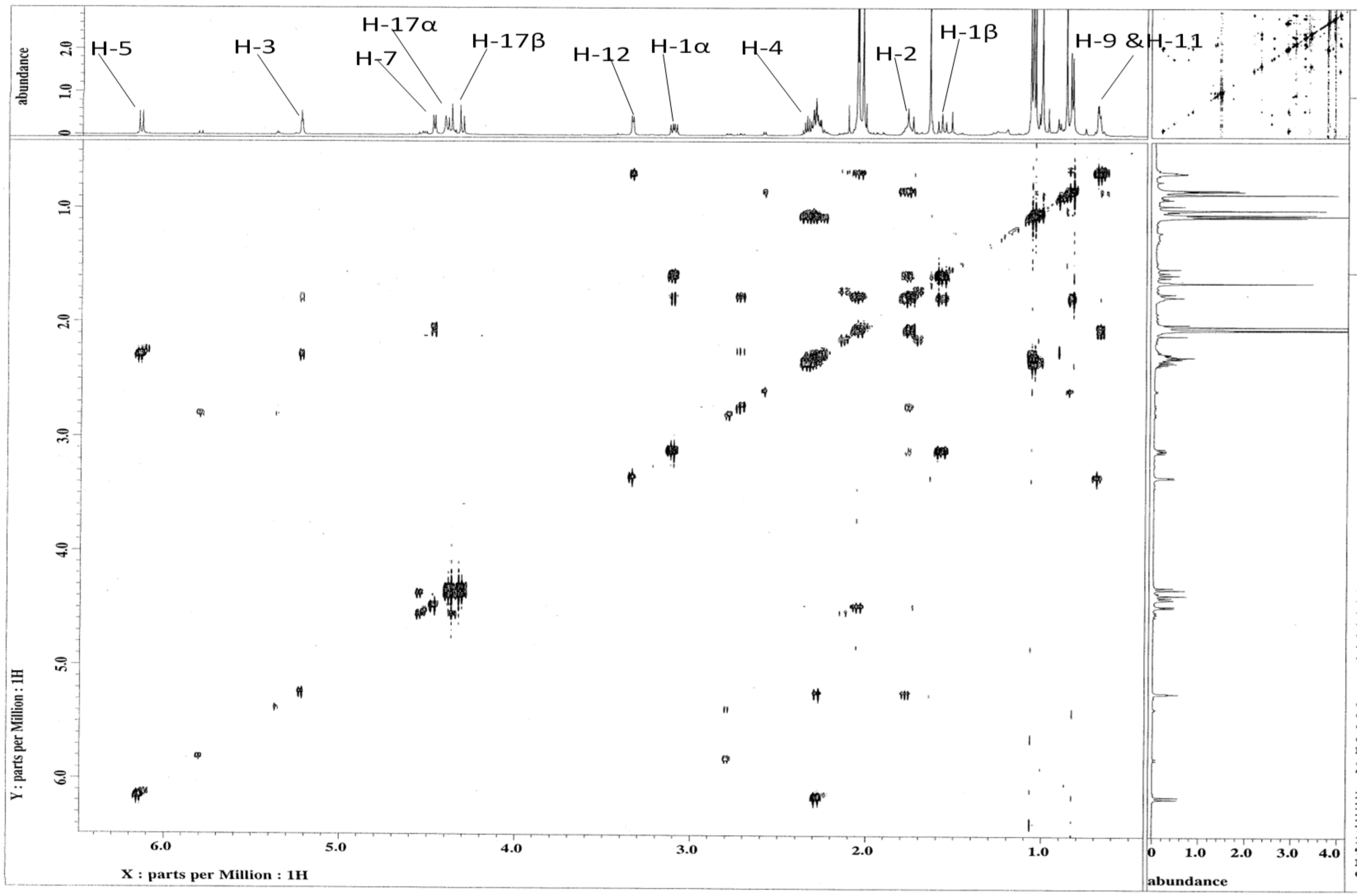

S50. HMBC spectrum of **6** in $CDCl_3$

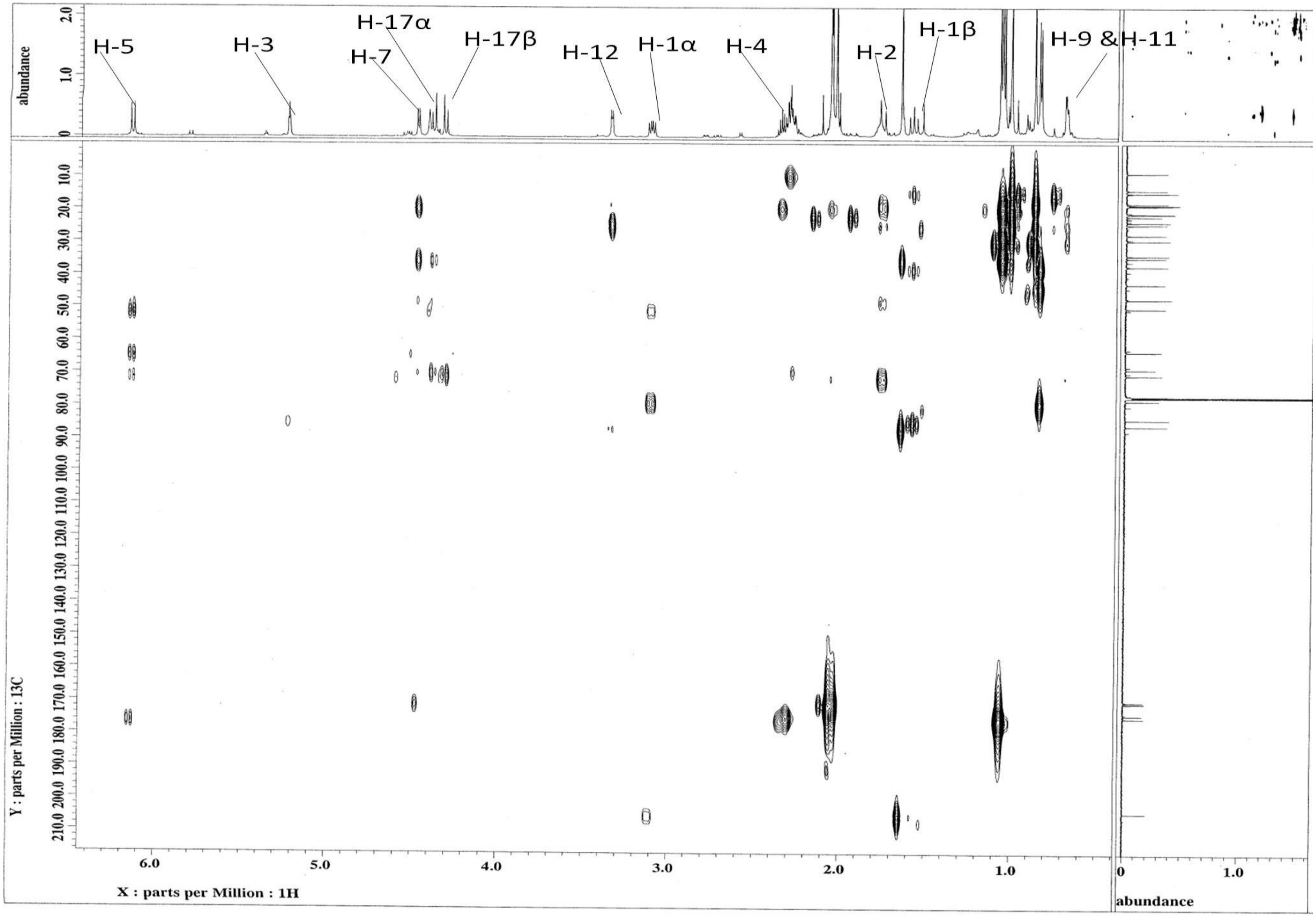

S50. HMBC spectrum of **6** in $CDCl_3$

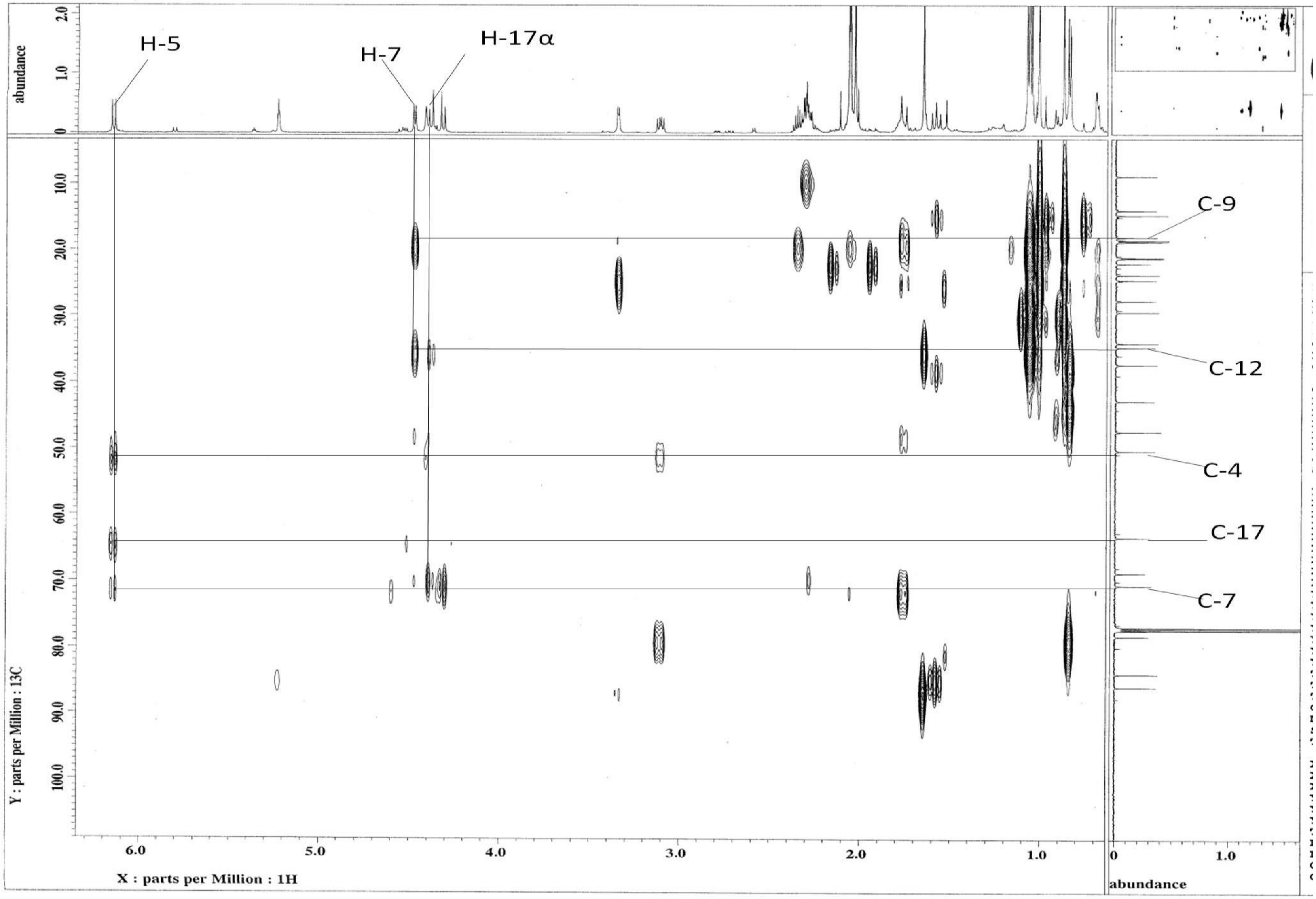

S51. HSQC spectrum of **6** in $CDCl_3$

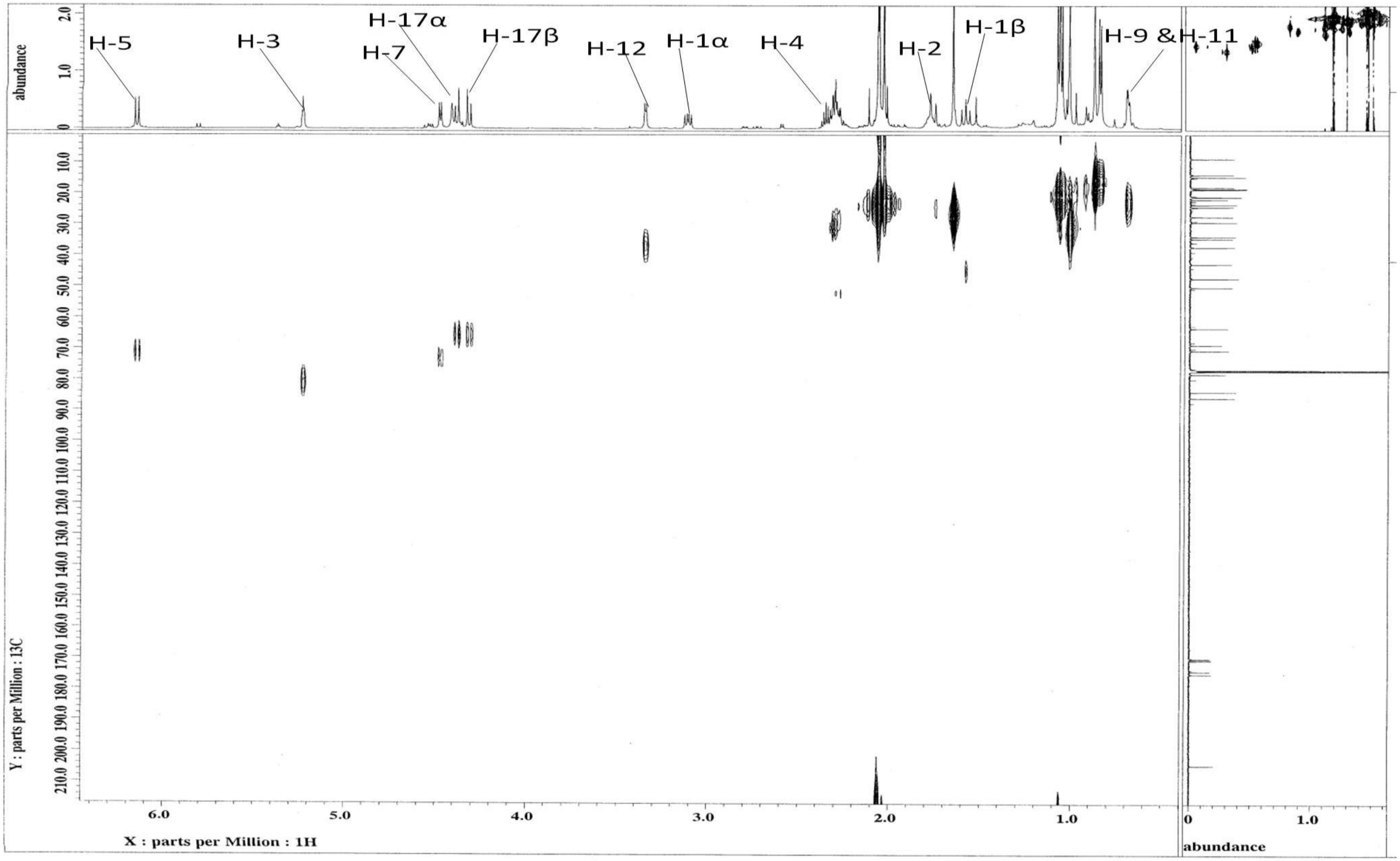

S52. ^{1}H NMR (600 MHz, $CDCl_3$) spectrum of **7**

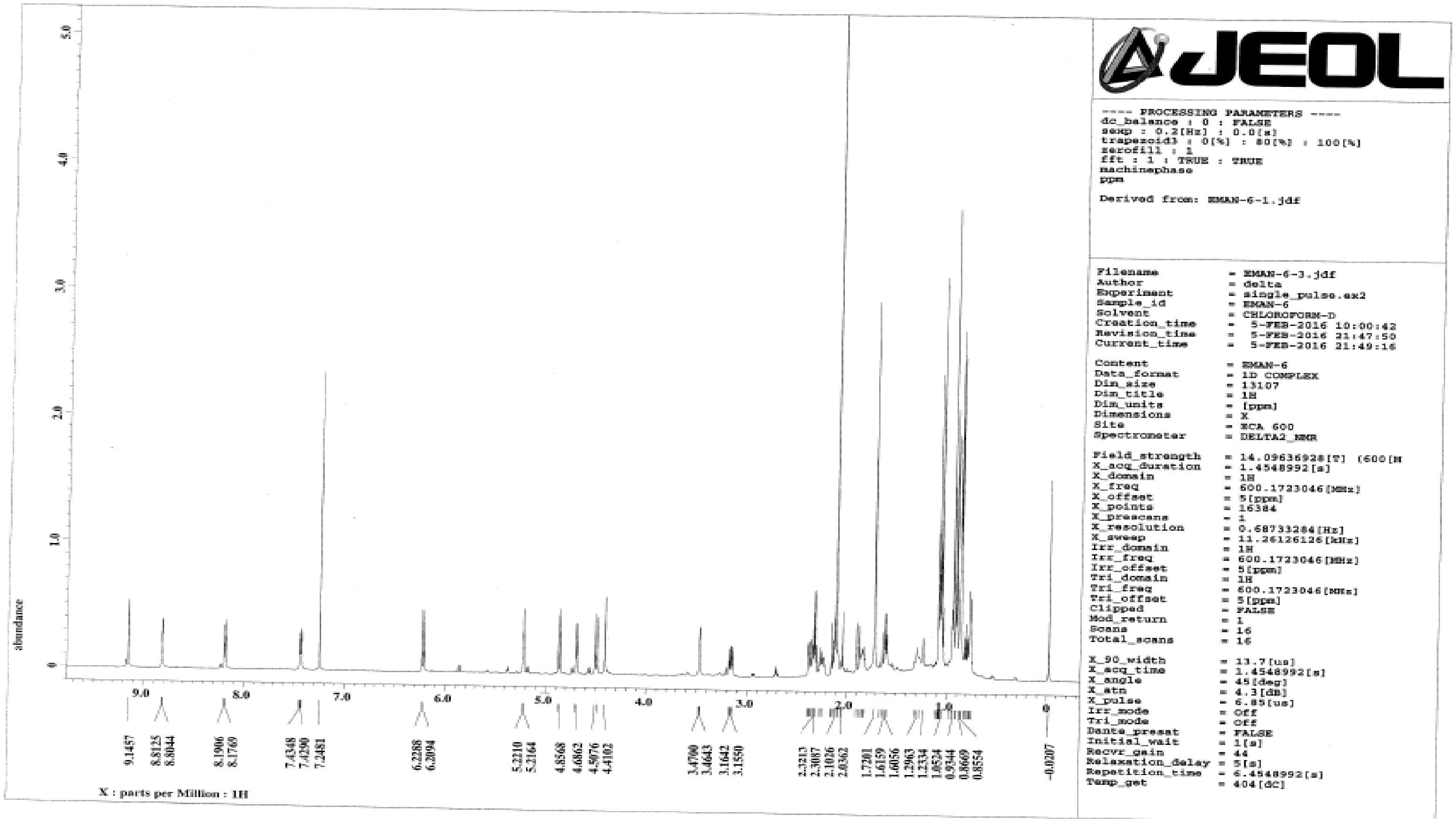

S53. ^{13}C NMR (150 MHz, $CDCl_3$) spectrum of **7**

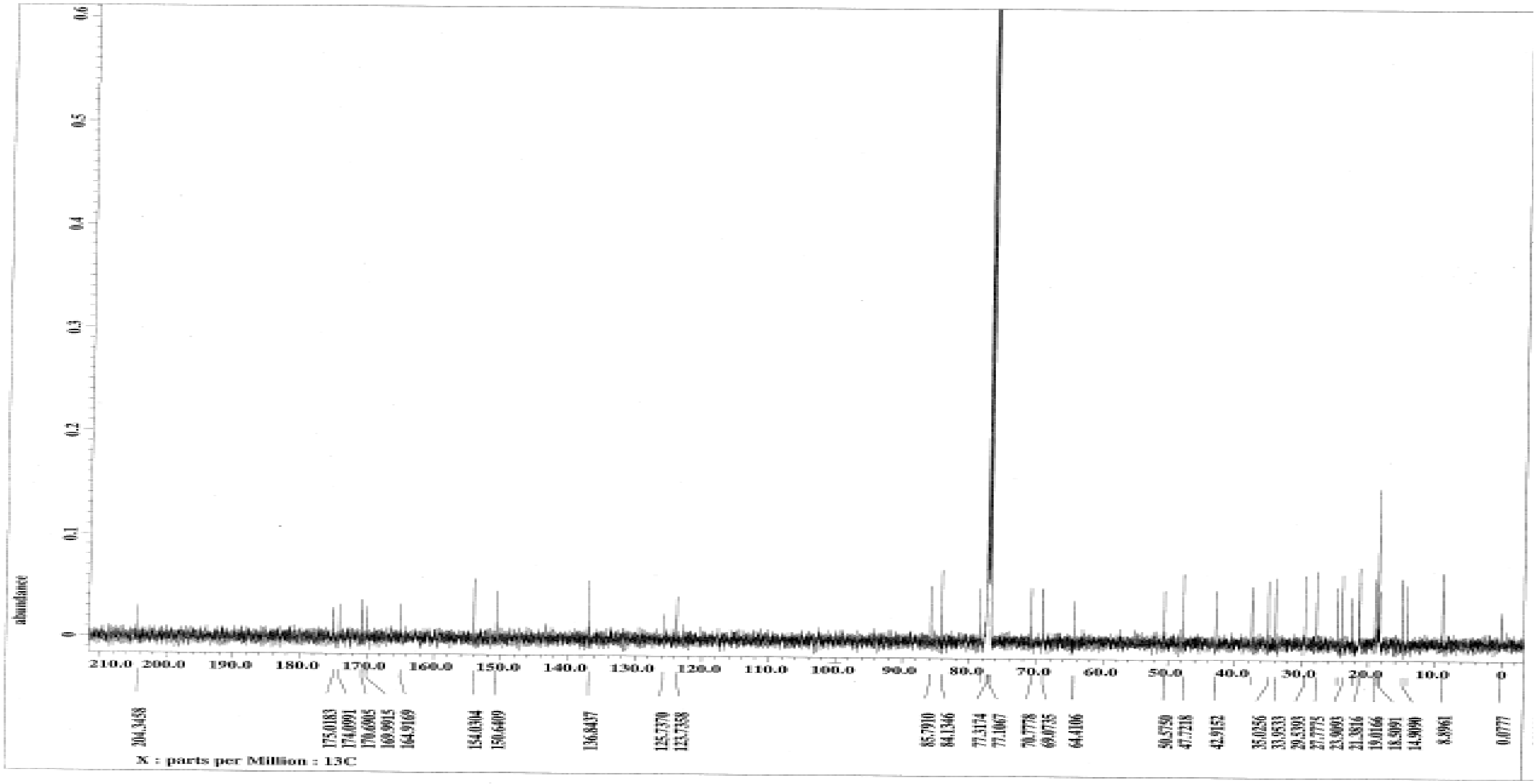

S54. LR-EI-MS of **7**

Note : 3-NOBA, CHCl3+NaIaq.
Inlet : Direct Ion Mode : FAB+
Spectrum Type : Normal Ion [MF-Linear]
RT : 0.50 min Scan# : (4,5)
BP : m/z 124.0000 Int. : 545.65
Output m/z range : 10.0000 to 751.0831 Cut Level : 0.00 %

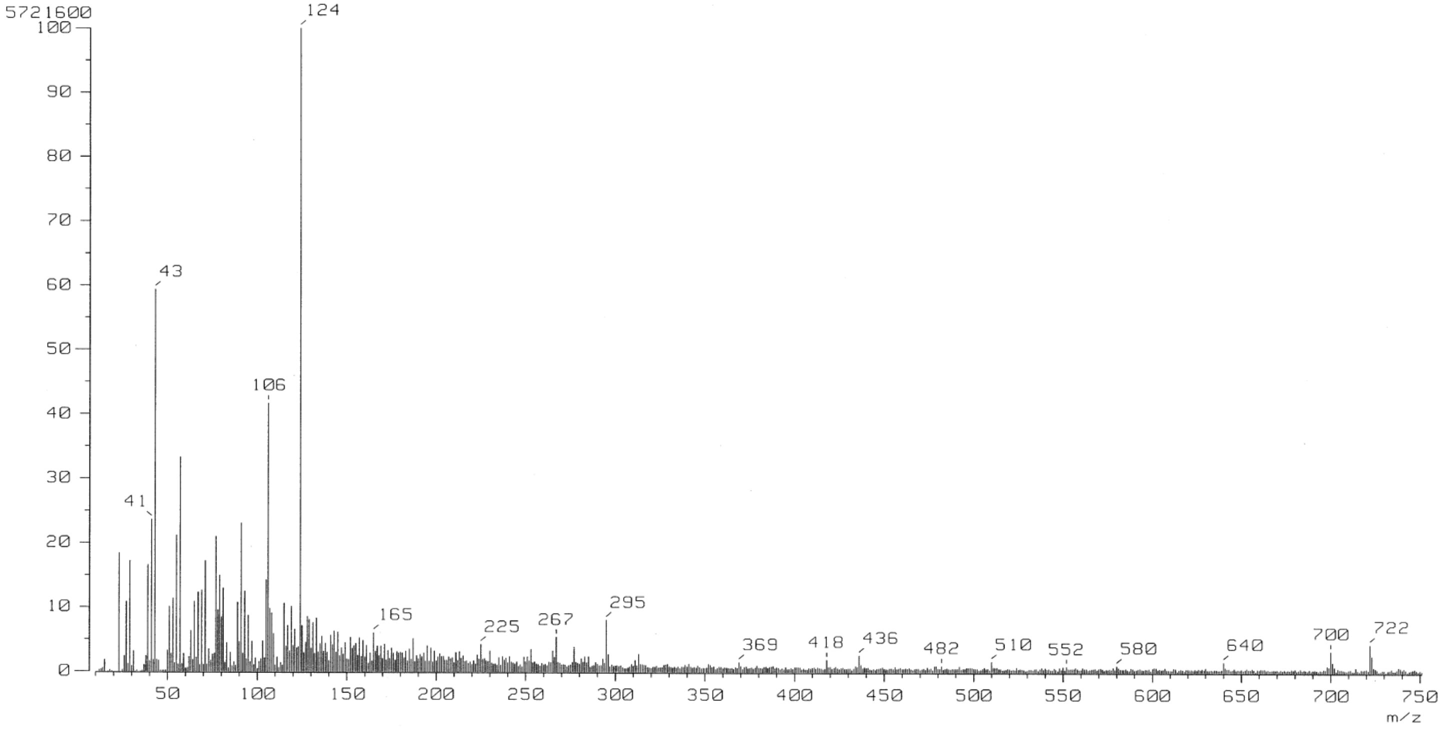

S55. ^{1}H NMR (600 MHz, $CDCl_3$) spectrum of **8**

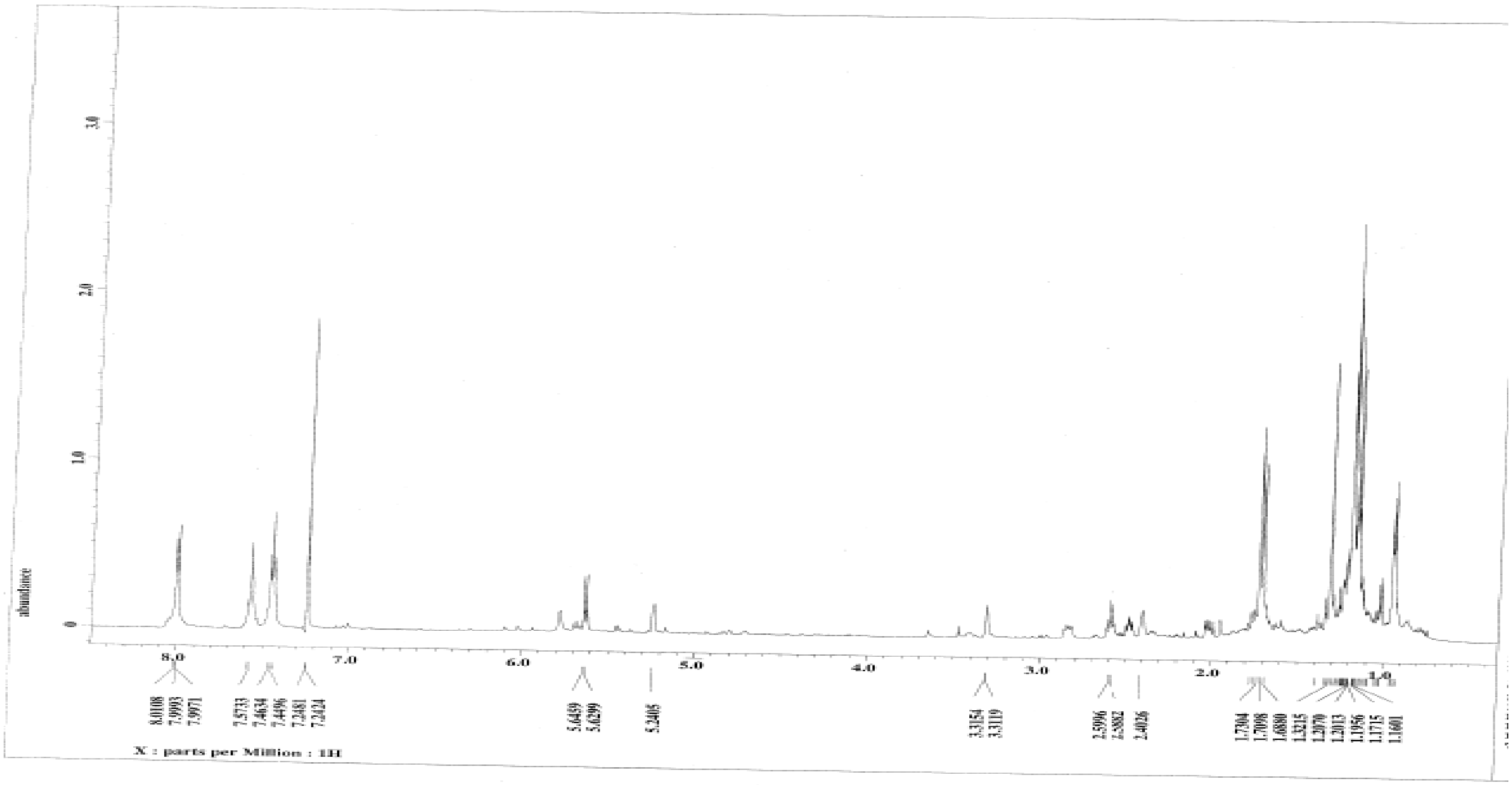

S56. ^{13}C NMR (150 MHz, $CDCl_3$) spectrum of **8**

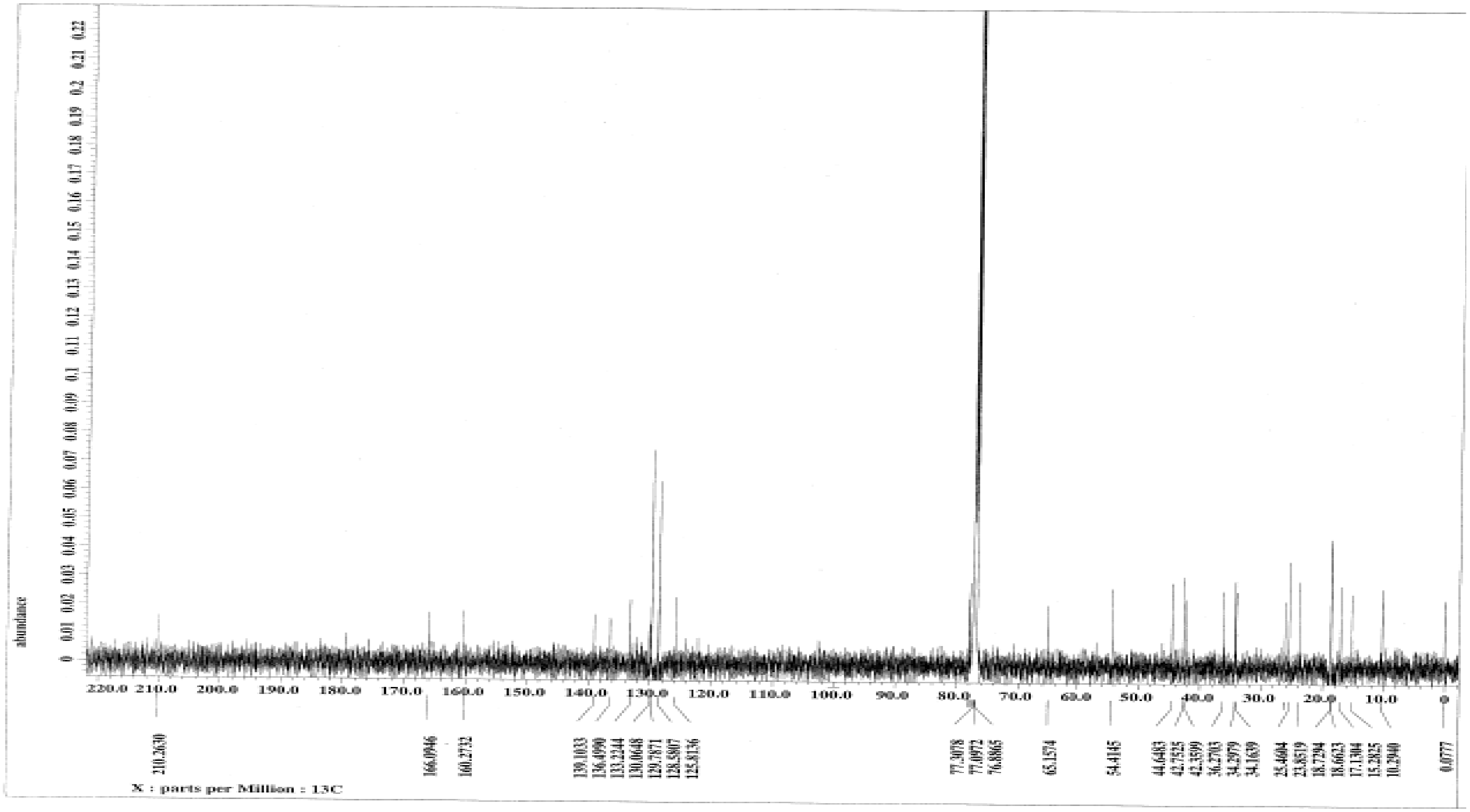

S57. LR-EI-MS of **8**

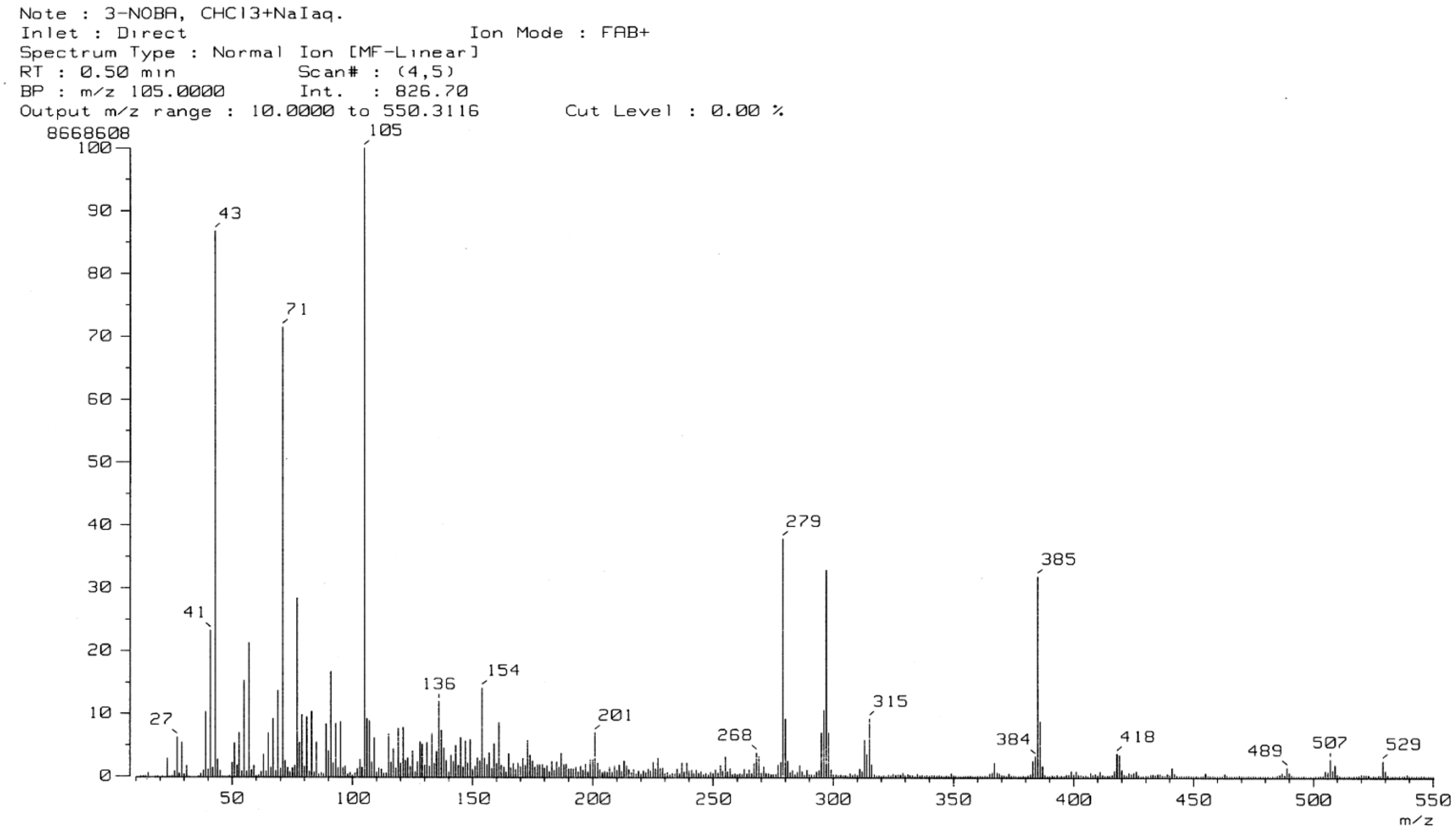

S58. ^{1}H NMR (600 MHz, $CDCl_3$) spectrum of **9**

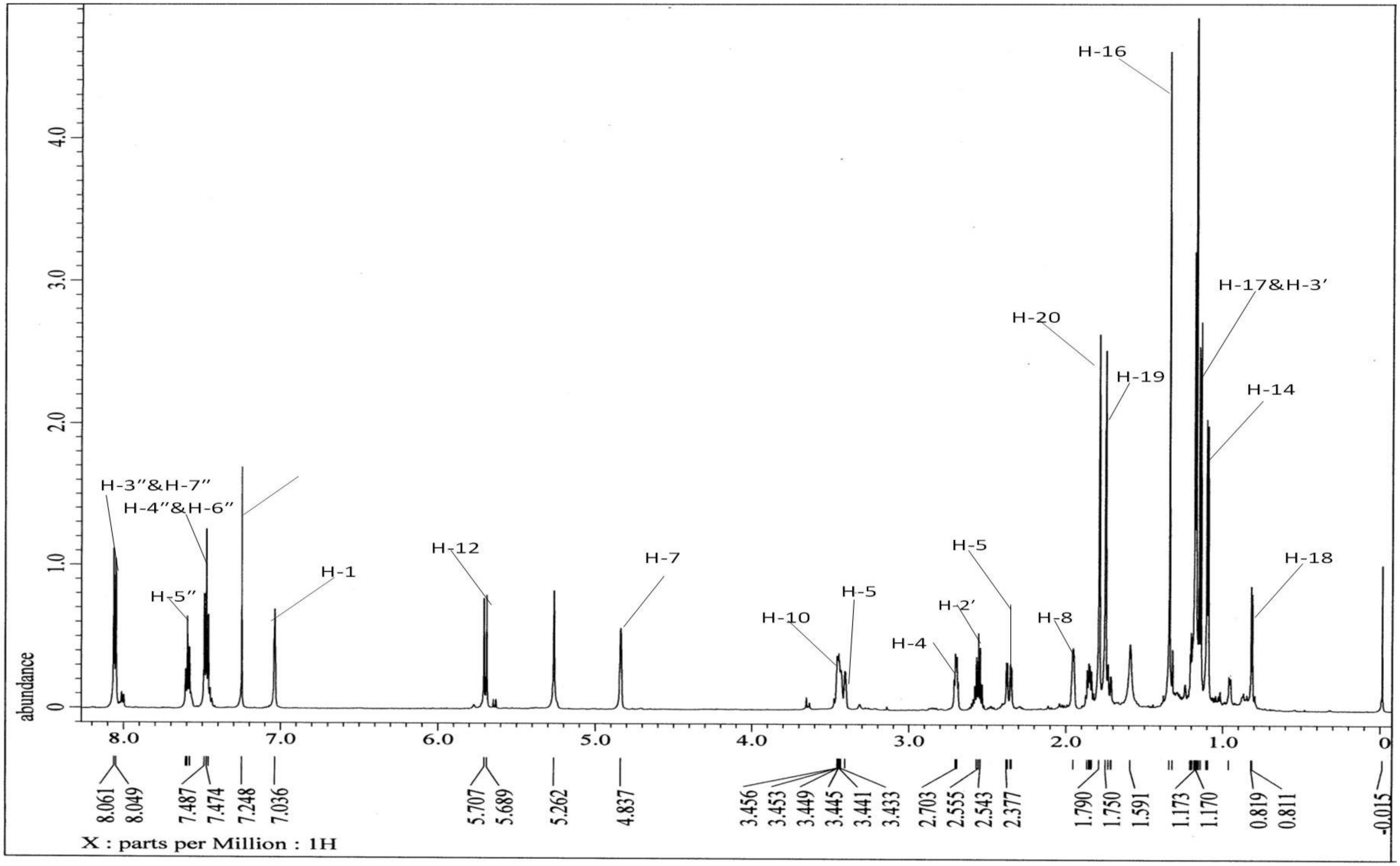

S59. ^{13}C NMR (150 MHz, $CDCl_3$) spectrum of **9**

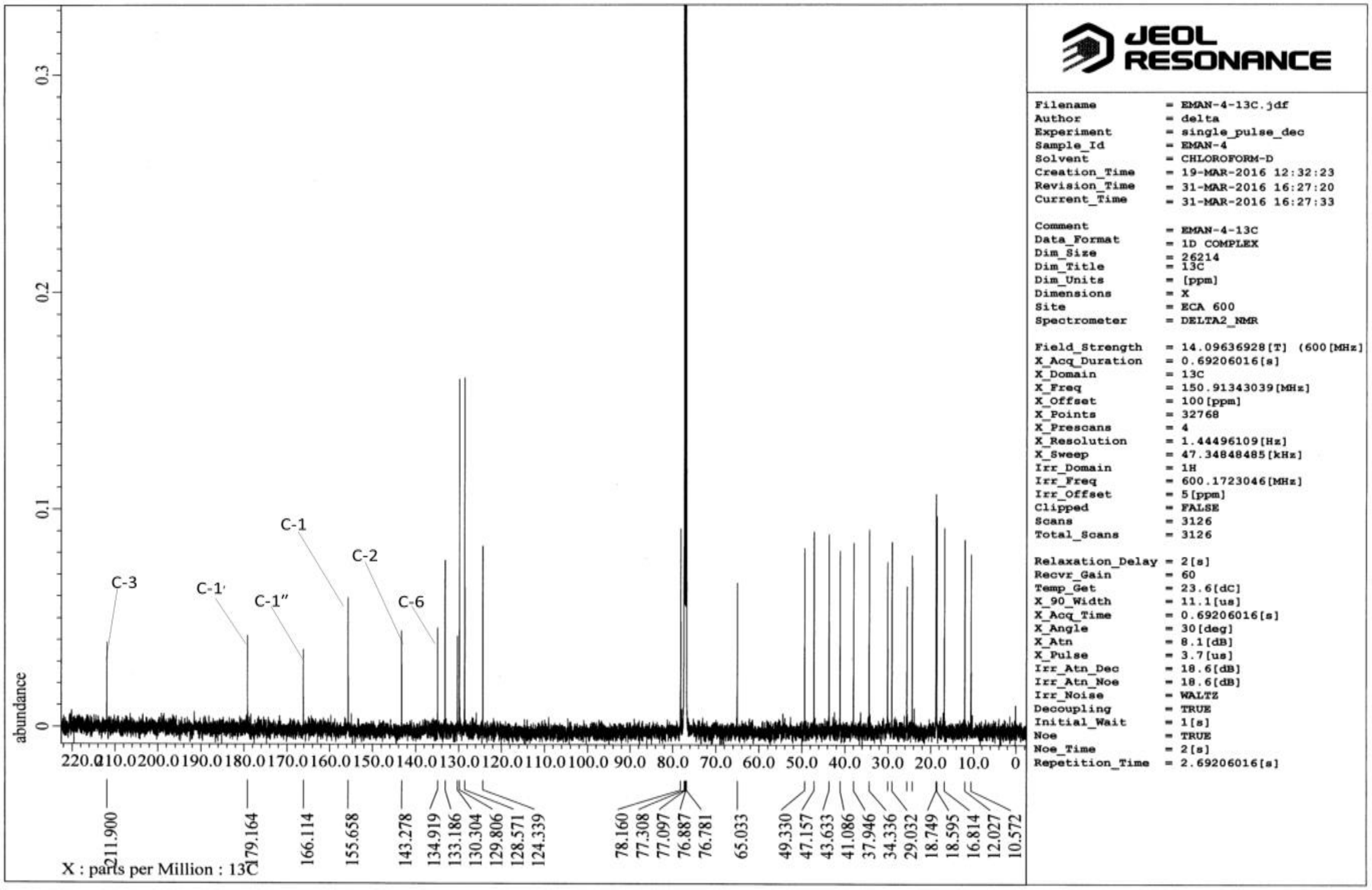

S59. ^{13}C NMR (150 MHz, $CDCl_3$) spectrum of **9**

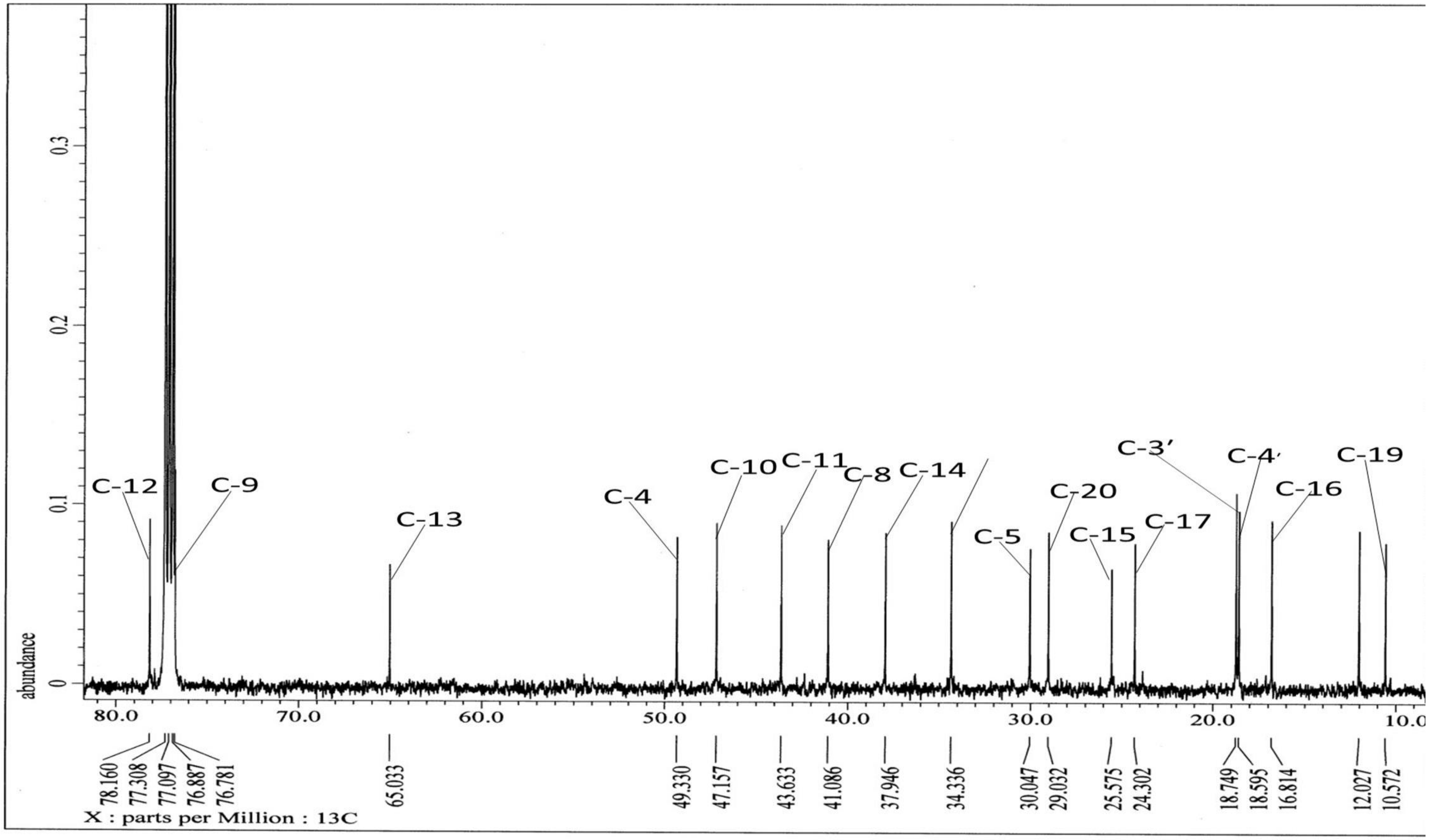

S60. . ^{1}H ^{1}H COSY spectrum of **9** in $CDCl_3$

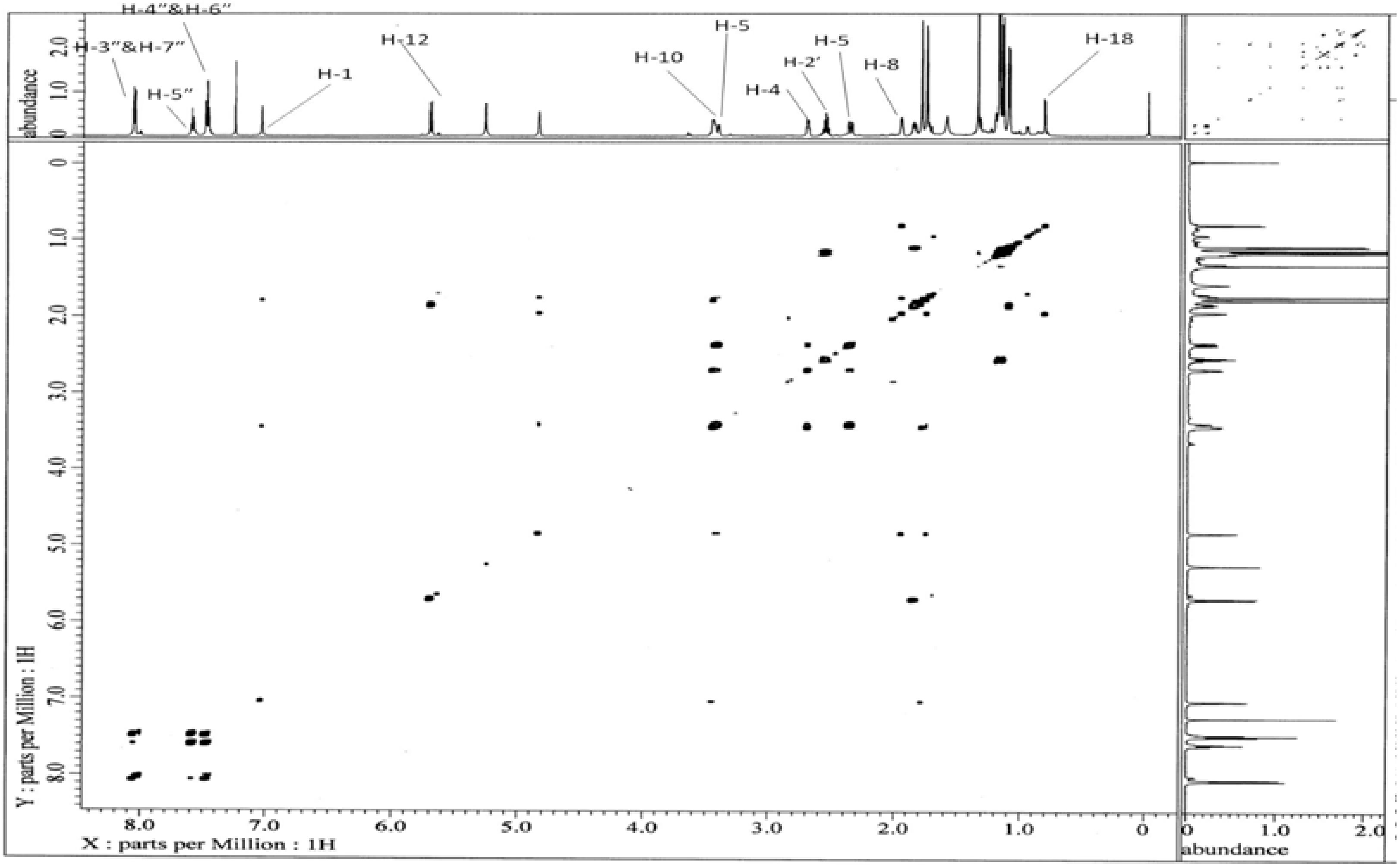

S61. . HMBC spectrum of **9** in $CDCl_3$

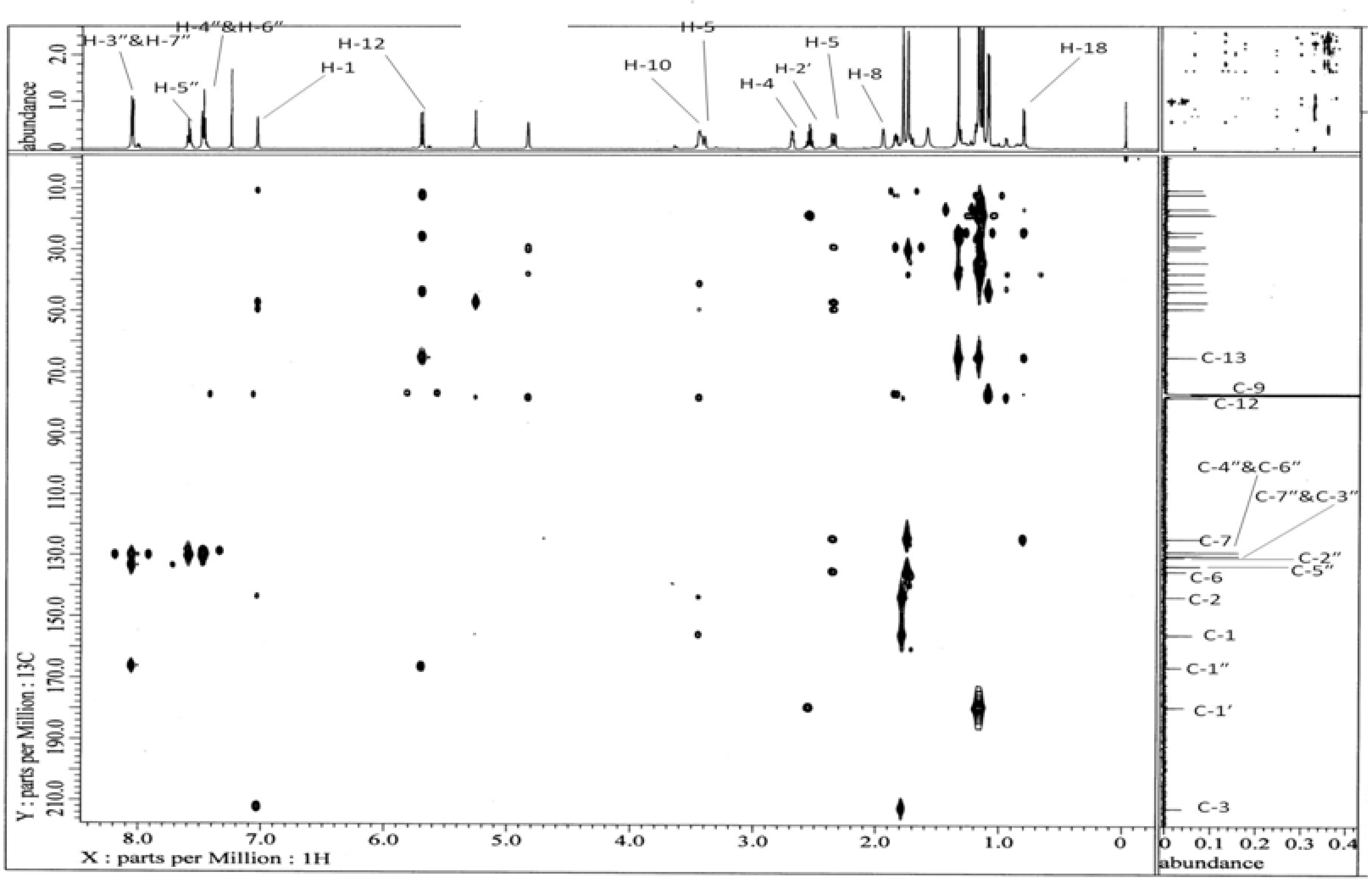

S62. HSQC spectrum of **9** in $CDCl_3$

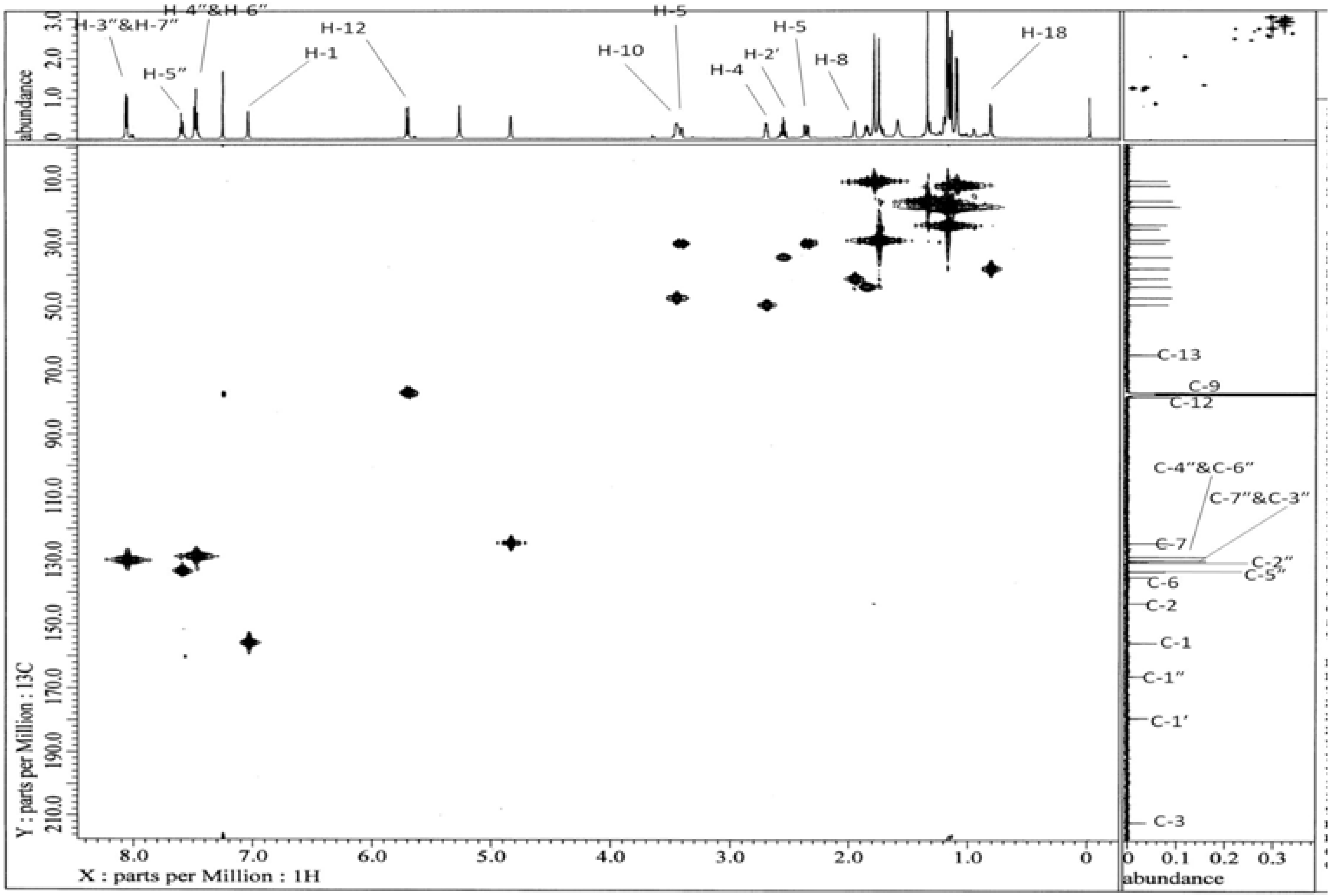

S63. NOESY experiment of **9** in $CDCl_3$

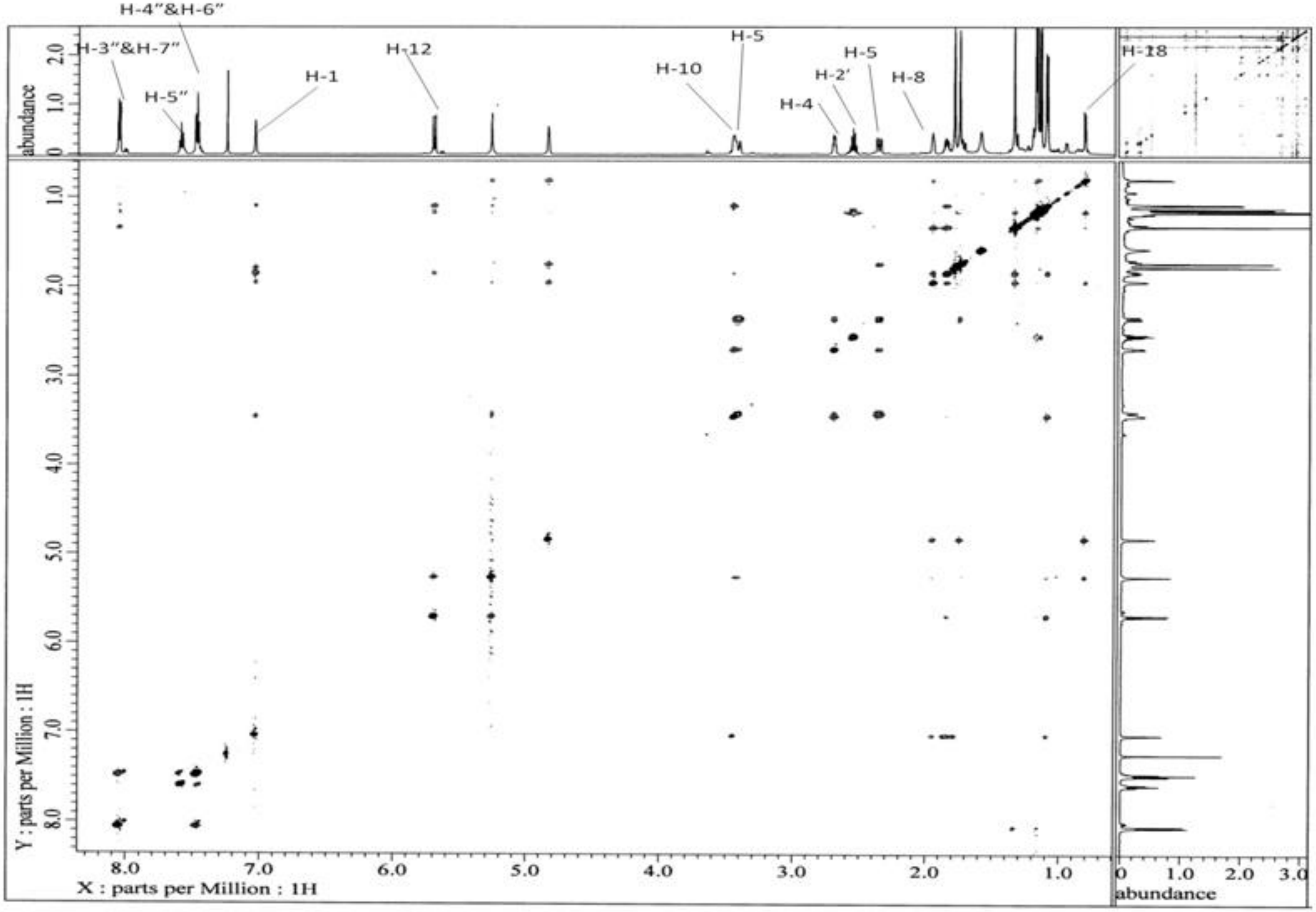

S64. . LR-EI-MS of **9**

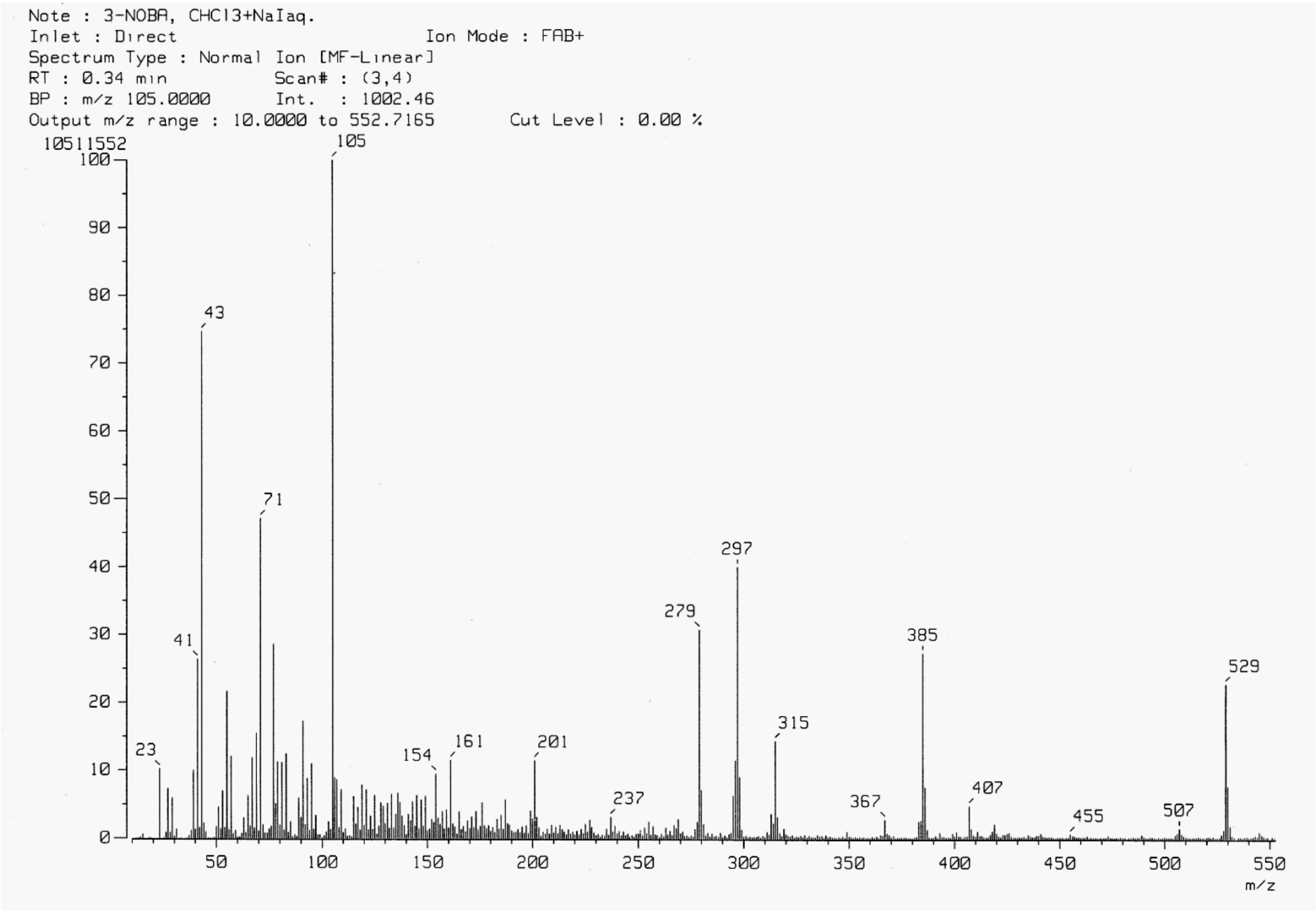

S65. ^{1}H NMR (600 MHz, $CDCl_3$) spectrum of **10**

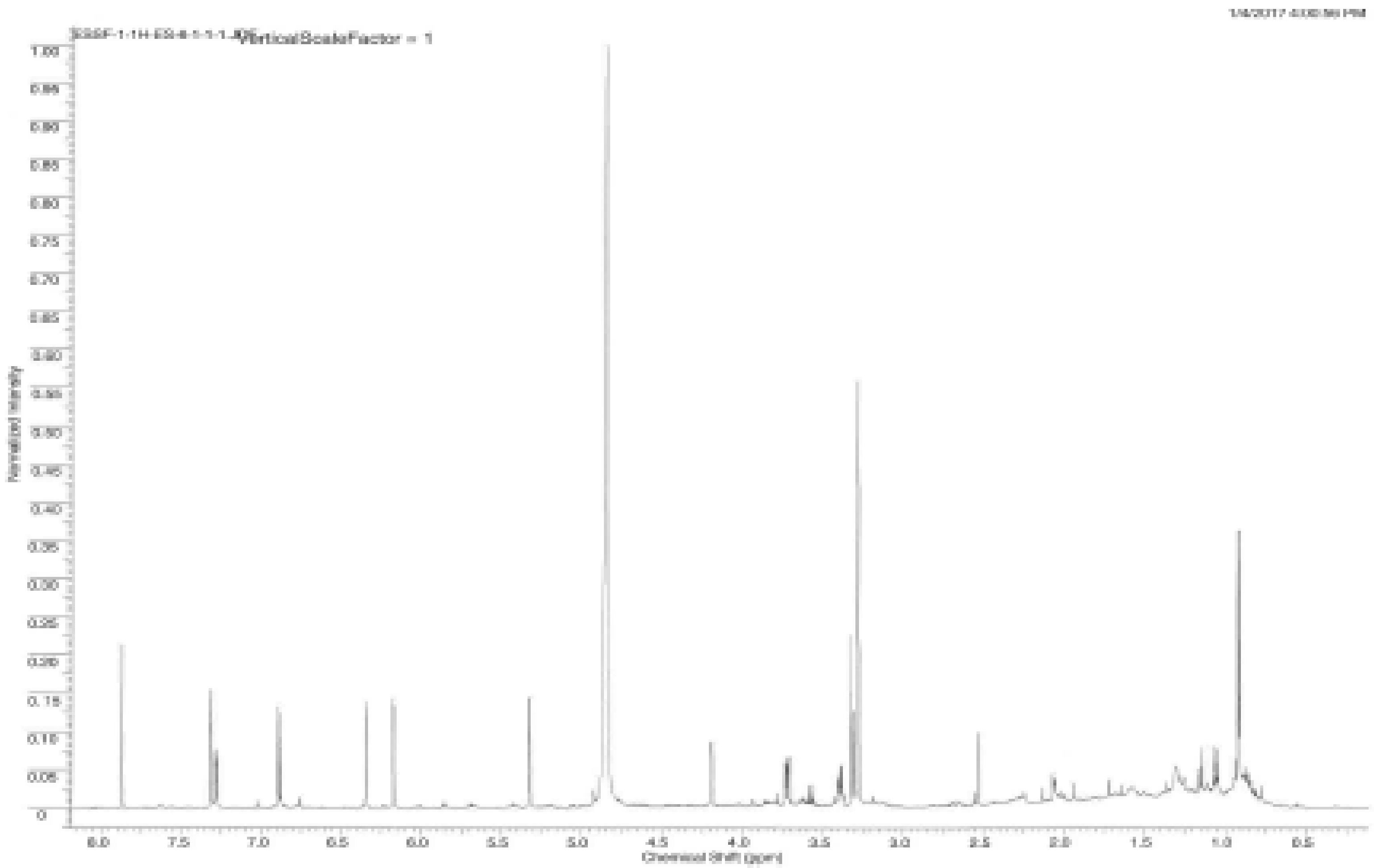

S66. ^{13}C NMR (150 MHz, $CDCl_3$) spectrum of **10**

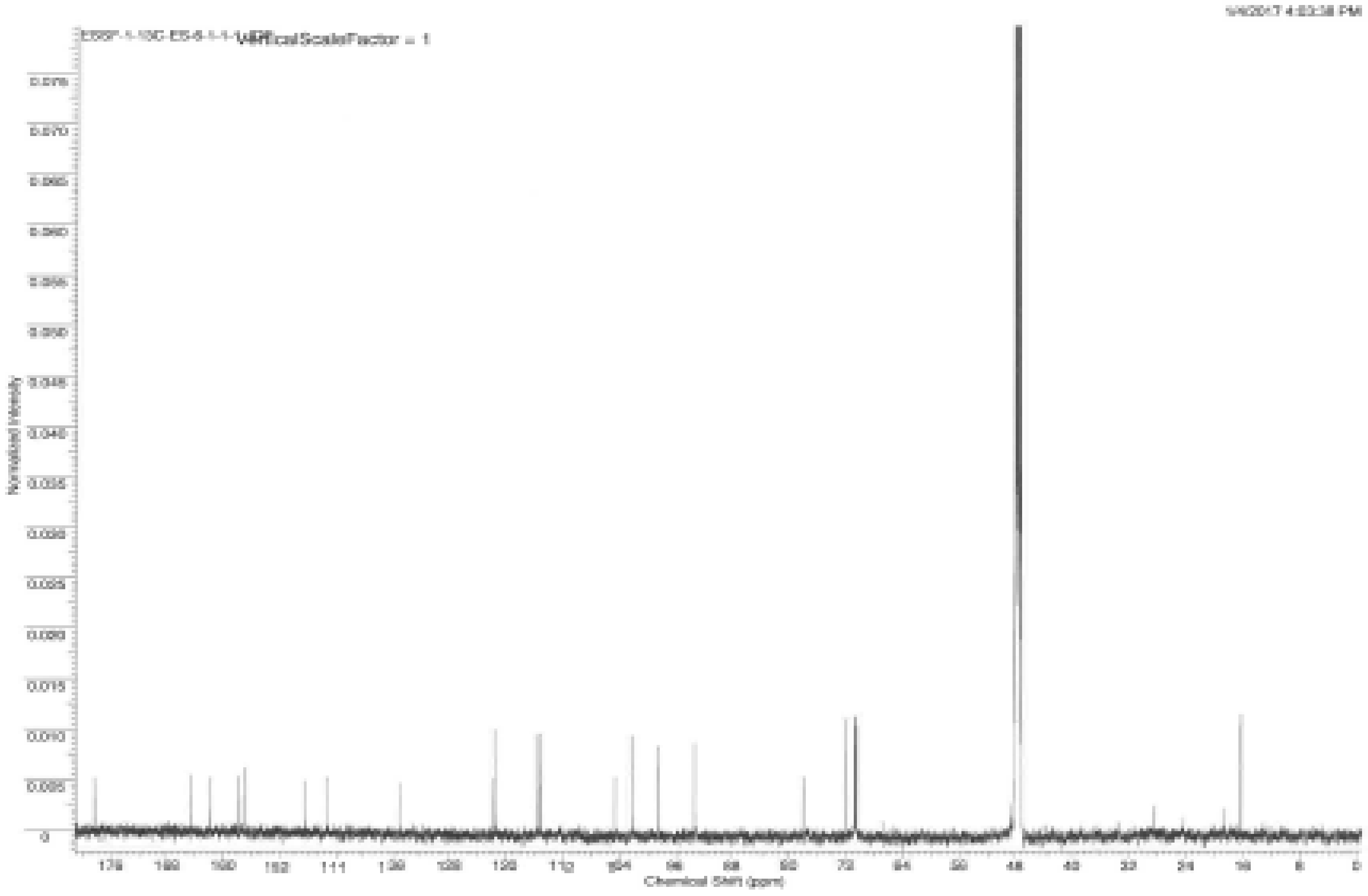

S67. ^{1}H NMR (600 MHz, $CDCl_3$) spectrum of **11**

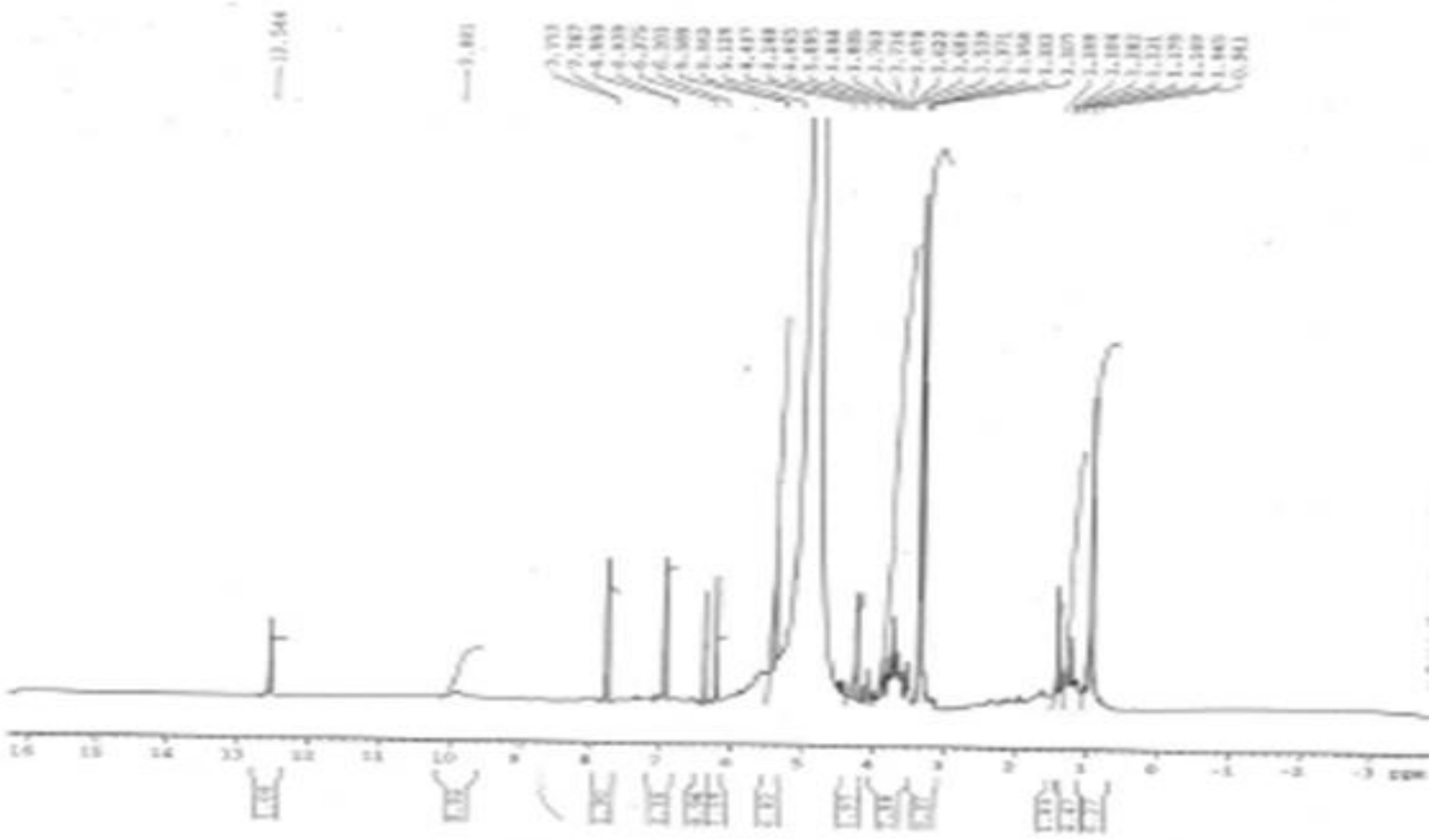

S68. ^{13}C NMR (150 MHz, $CDCl_3$) spectrum of **11**

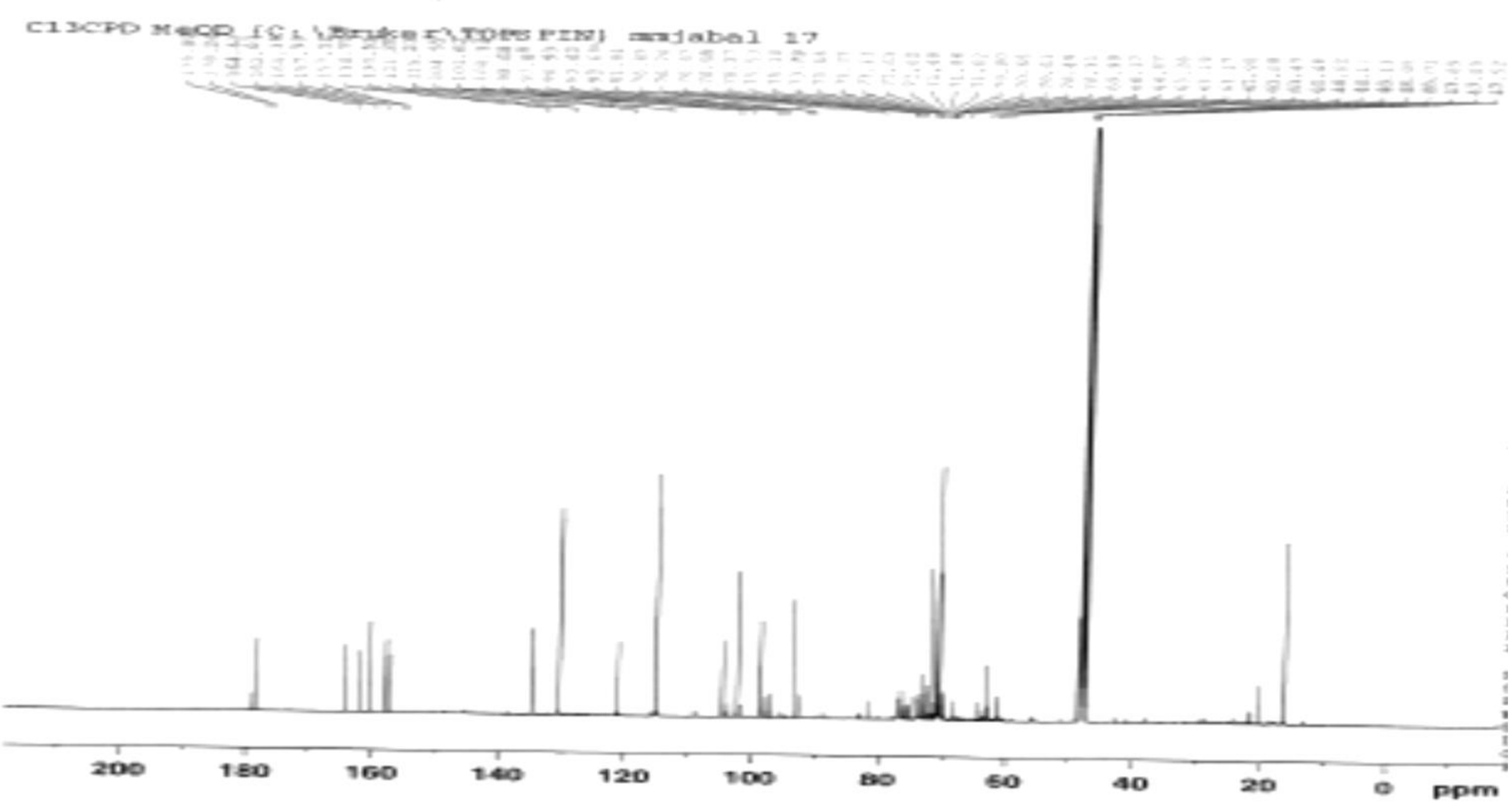

S69. ^{1}H NMR (600 MHz, $CDCl_3$) spectrum of **12**

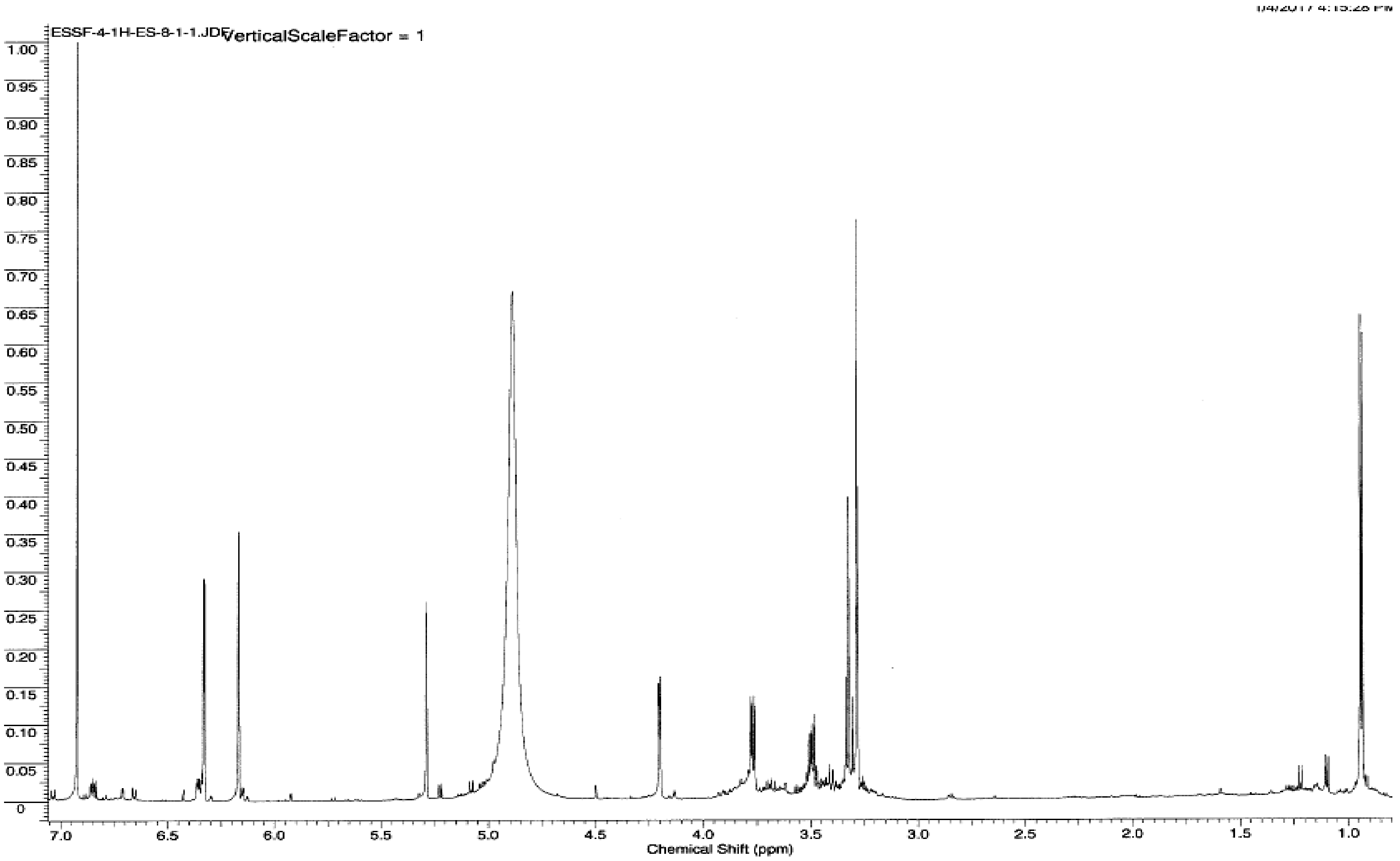

S70. ^{13}C NMR (150 MHz, $CDCl_3$) spectrum of **12**

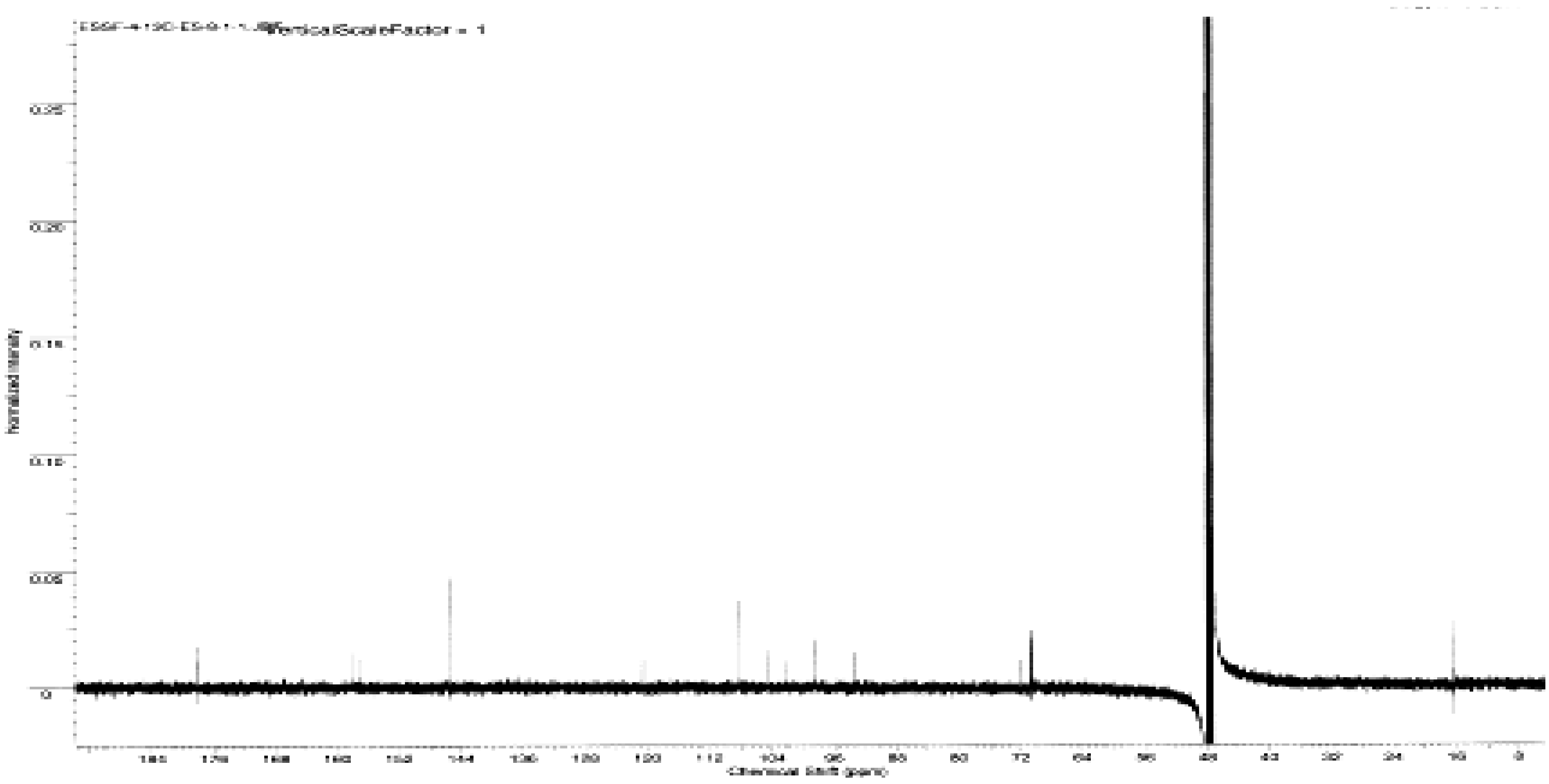

S71. ^{1}H NMR (600 MHz, $CDCl_3$) spectrum of **13**

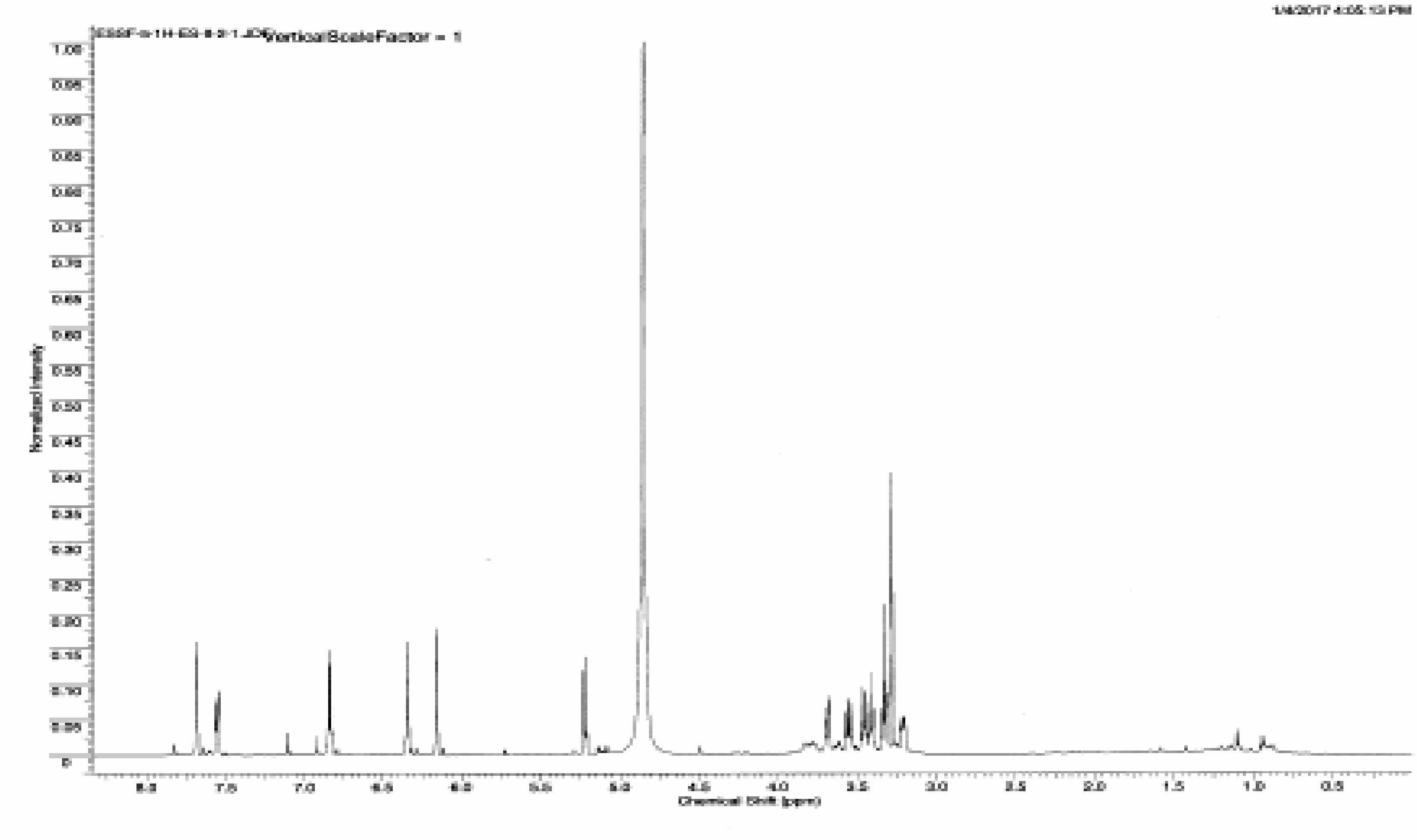

S72. ^{13}C NMR (150 MHz, $CDCl_3$) spectrum of **13**

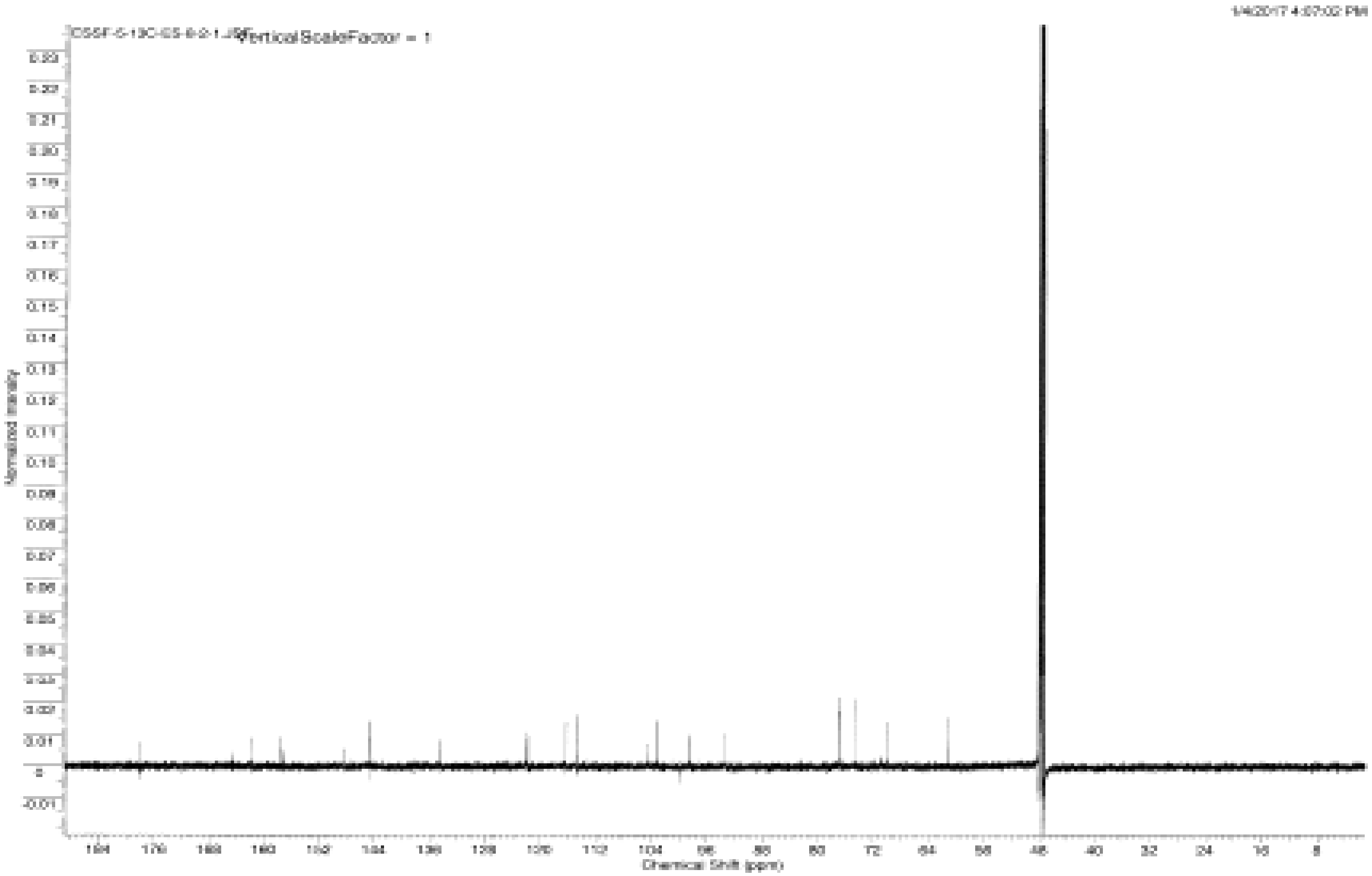

S 73. Photomicrographs showing morphological changes of Caco-2 cells following 48 h exposure to compound 1 serial dilutions as mentioned at the *Experimental* section. Morphological signs of cytotoxicity include cell rounding, shrinking and loss of monolayer integrity compared to vehicle control. Total magnifictaion=150×.

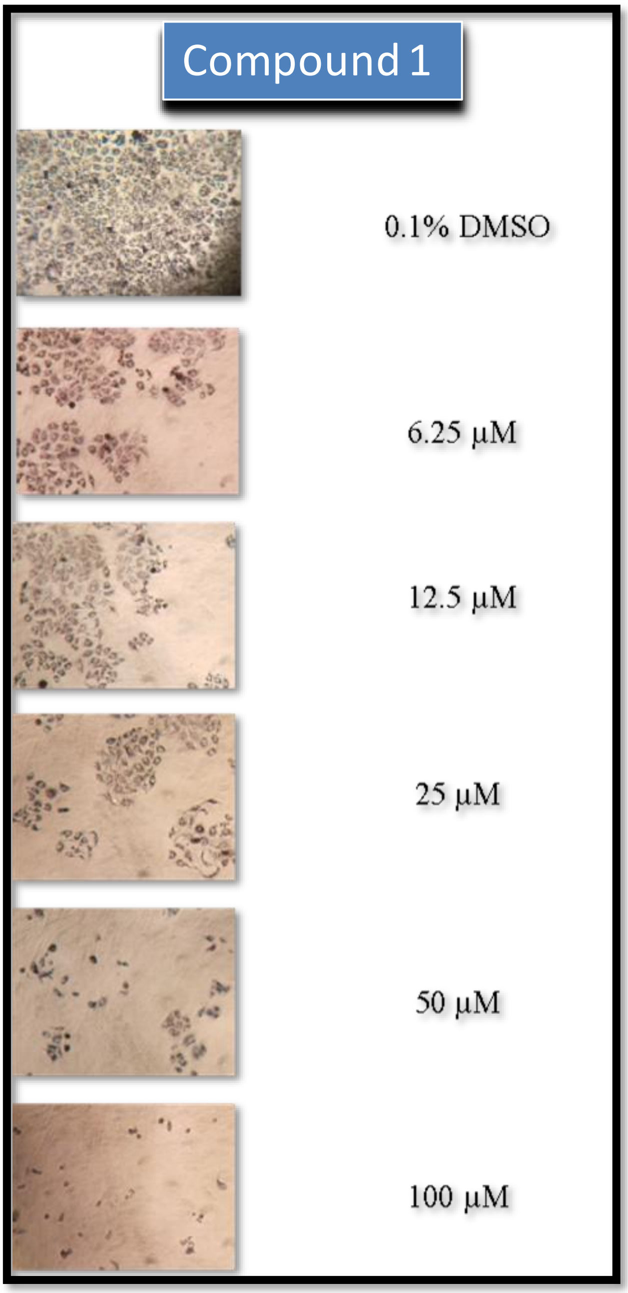

S74. Photomicrographs showing morphological changes of Caco-2 cells (A) or A549 cells (B) following 48 h exposure to compound 2 serial dilutions as mentioned at the *Experimental* section. Morphological signs of cytotoxicity include cell rounding, shrinking and loss of monolayer integrity compared to vehicle control. Total magnifictaion=150×.

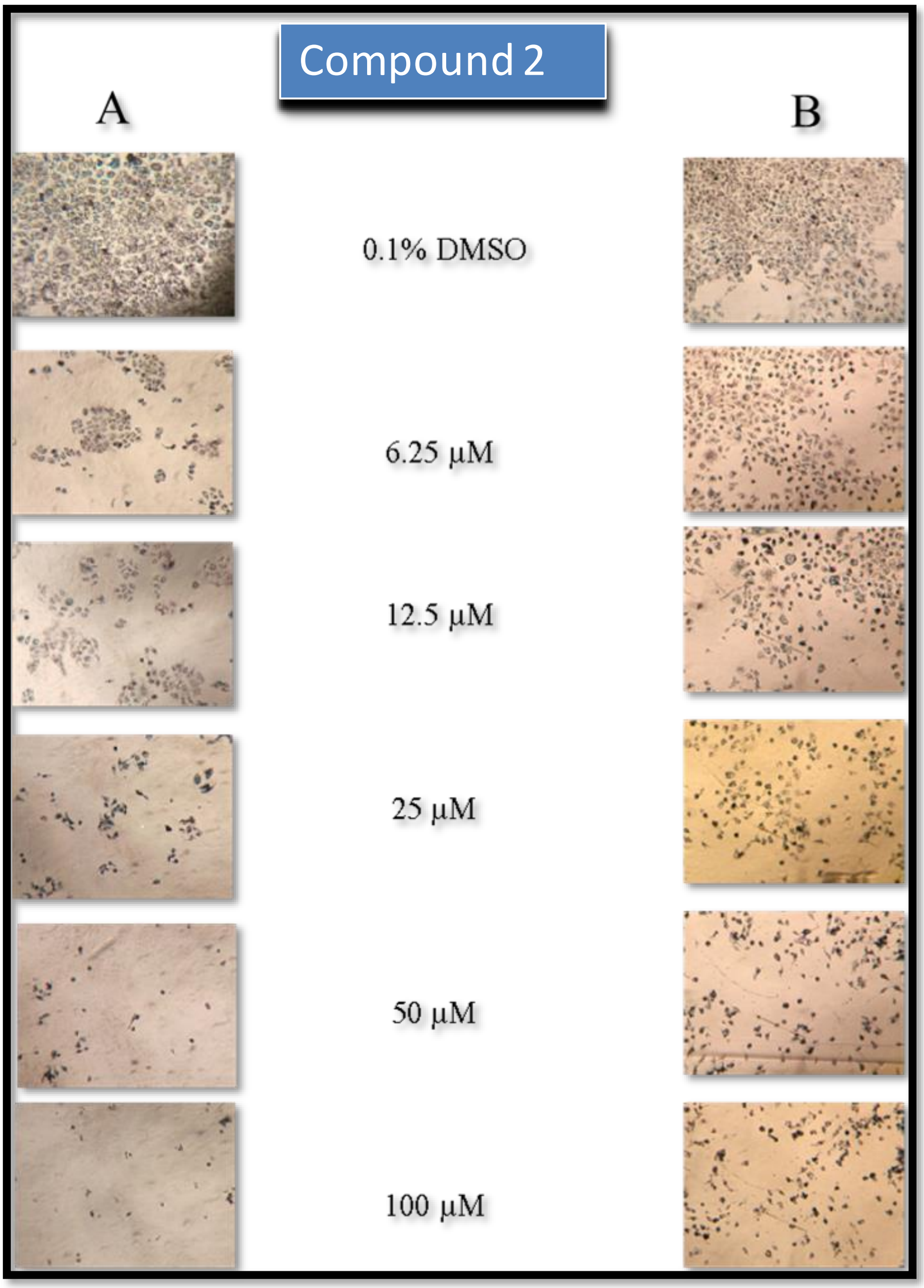

S75. Photomicrographs showing morphological changes of Caco-2 cells (A) or A549 cells (B) following 48 h exposure to compound 3 serial dilutions as mentioned at the Experimental section. Morphological signs of cytotoxicity include cell rounding, shrinking and loss of monolayer integrity compared to vehicle control. Total magnifictaion=150×.

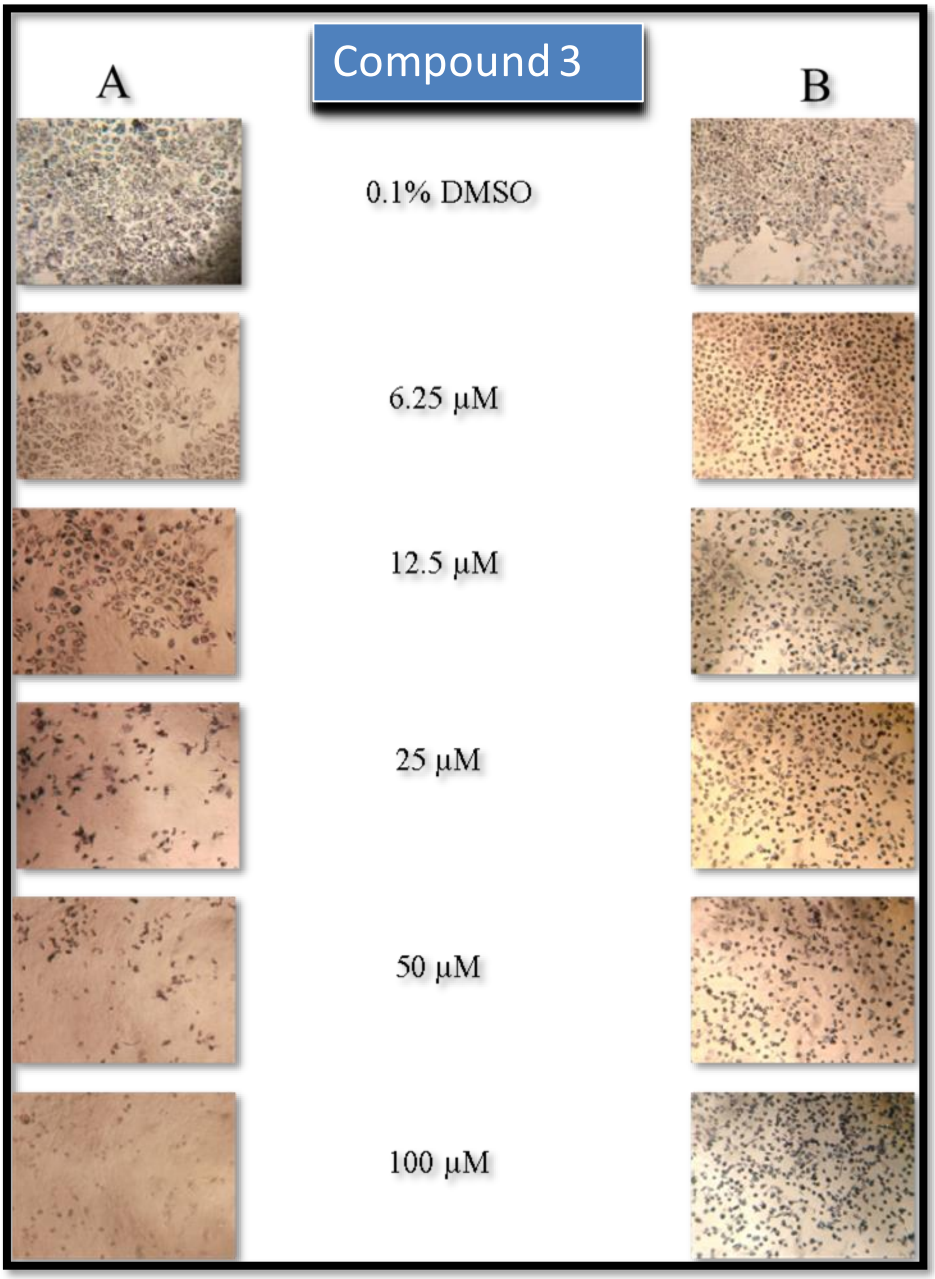

S76. Photomicrographs showing morphological changes of Caco-2 cells (A) or A549 cells (B) following 48 h exposure to compound 4 serial dilutions as mentioned at the Experimental section. Morphological signs of cytotoxicity include cell rounding, shrinking and loss of monolayer integrity compared to vehicle control. Total magnifictaion=150×.

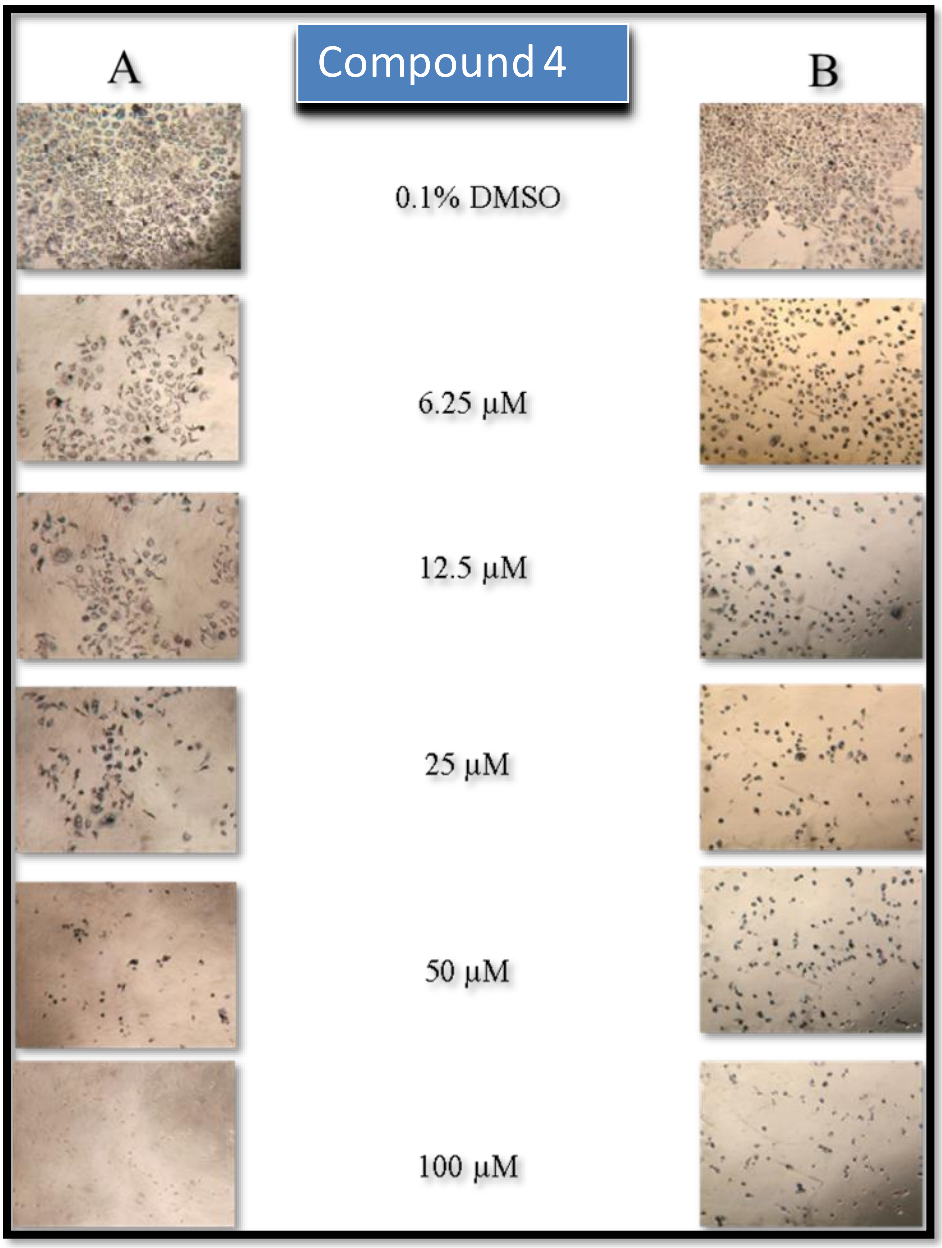

S77. Photomicrographs showing morphological changes of Caco-2 cells (A) or A549 cells (B) following 48 h exposure to compound 5 serial dilutions as mentioned at the Experimental section. Morphological signs of cytotoxicity include cell rounding, shrinking and loss of monolayer integrity compared to vehicle control. Total magnifictaion=150×.

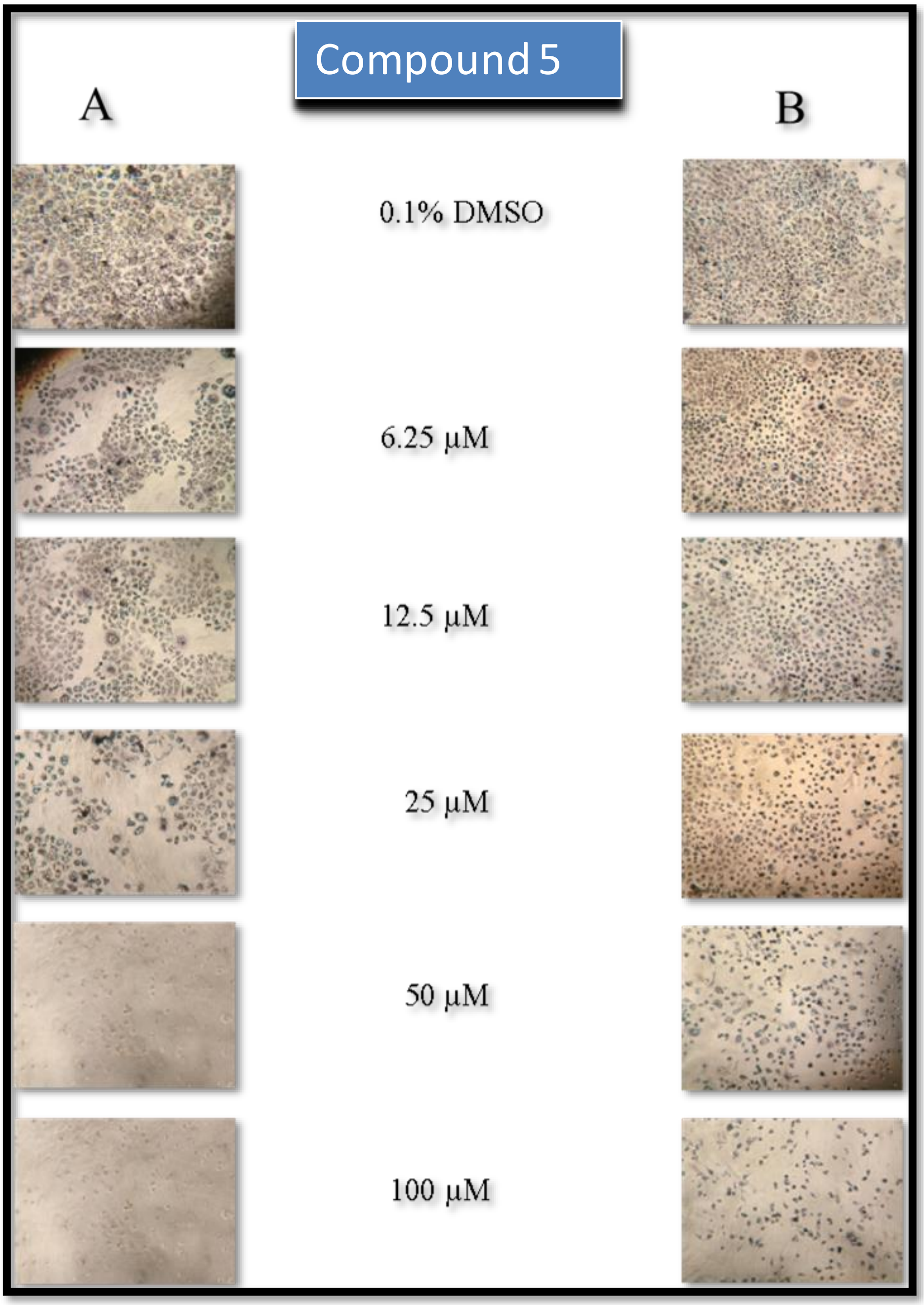

S78. Photomicrographs showing morphological changes of Caco-2 cells (A) or A549 cells (B) following 48 h exposure to compound 6 serial dilutions as mentioned at the Experimental section. Morphological signs of cytotoxicity include cell rounding, shrinking and loss of monolayer integrity compared to vehicle control. Total magnifictaion=150×.

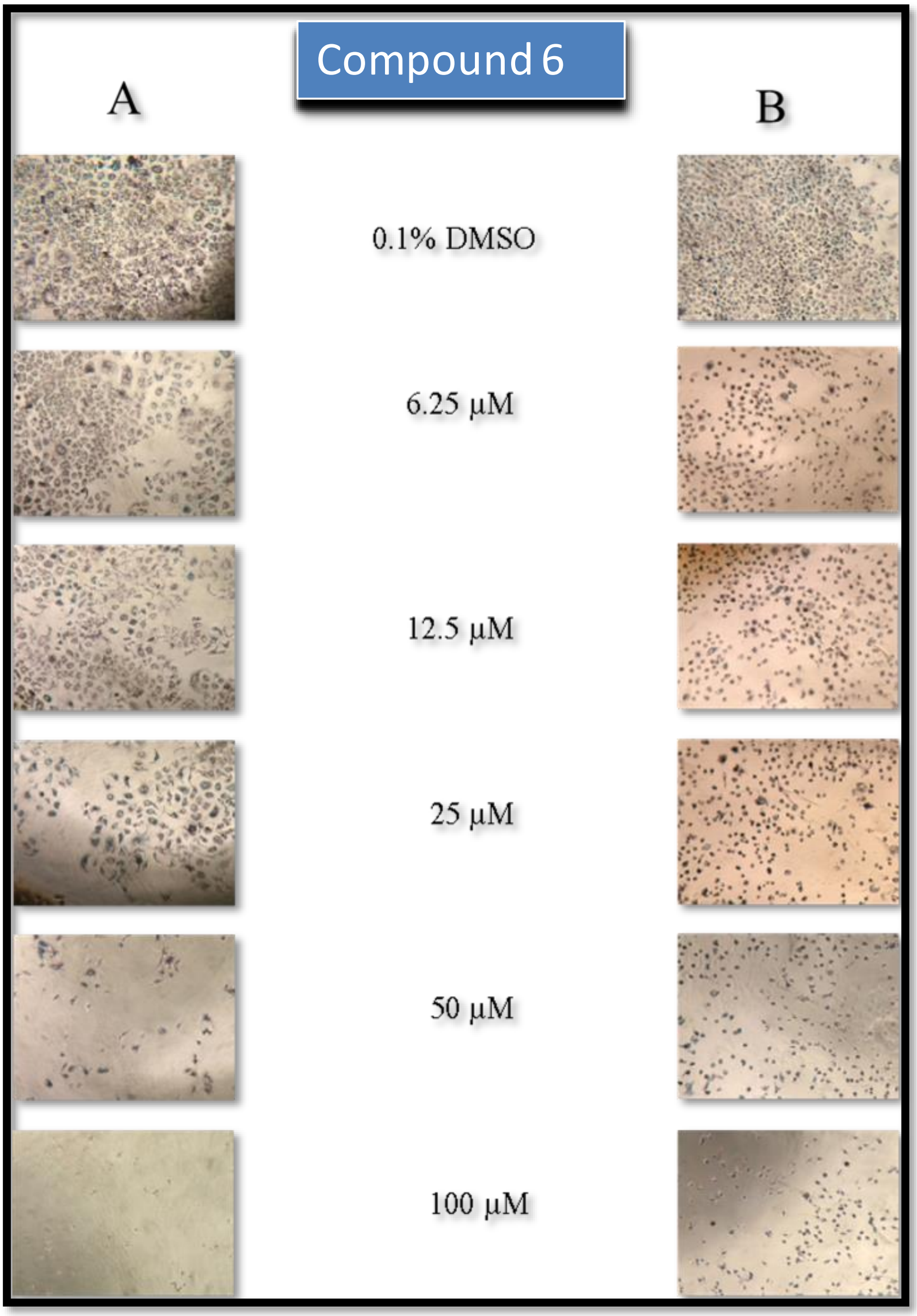

S79. Photomicrographs showing morphological changes of Caco-2 cells (A) or A549 cells (B) following 48 h exposure to compound 7 serial dilutions as mentioned at the Experimental section. Morphological signs of cytotoxicity include cell rounding, shrinking and loss of monolayer integrity compared to vehicle control. Total magnifictaion=150×.

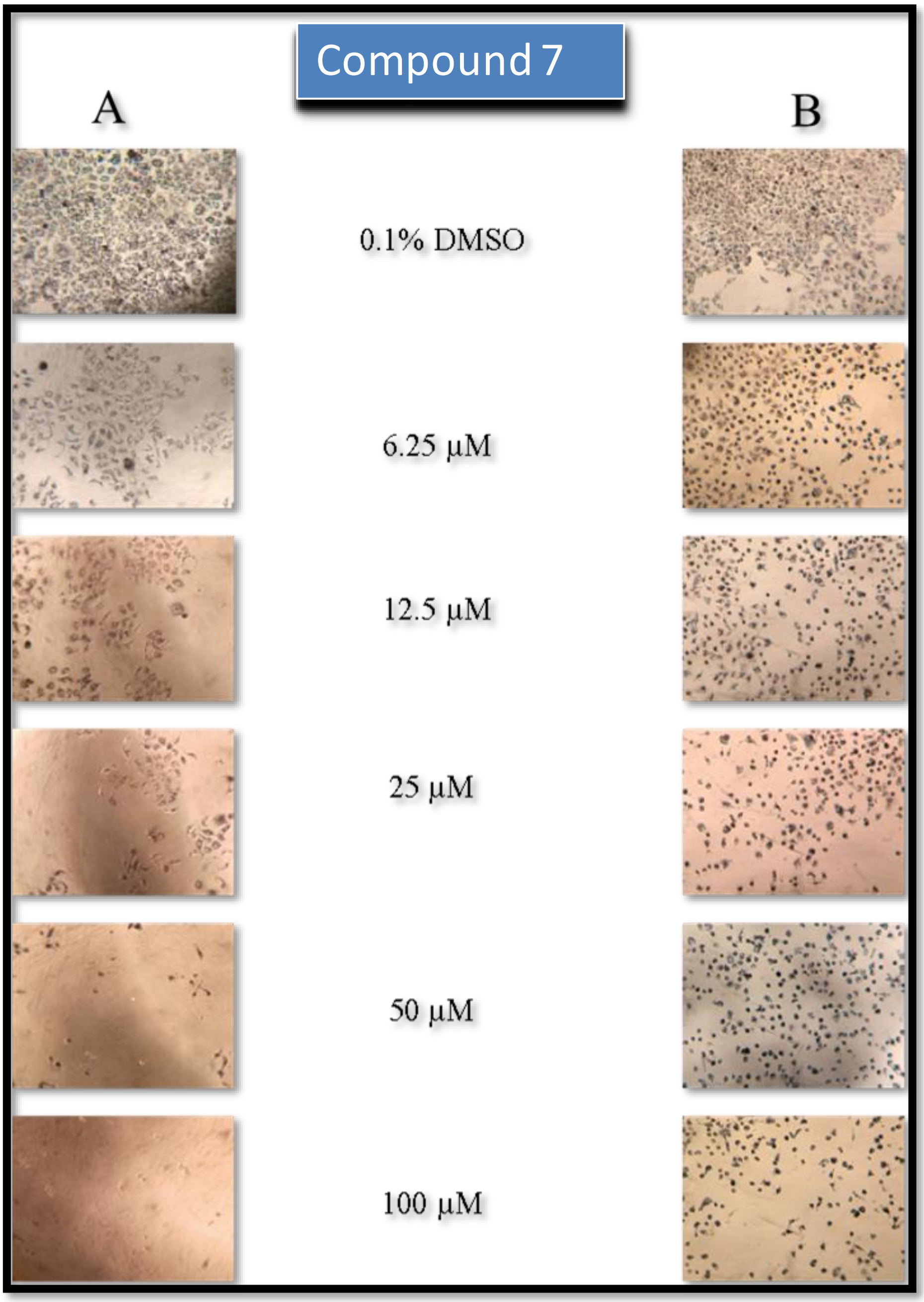

S80. Photomicrographs showing morphological changes of Caco-2 cells (A) or A549 cells (B) following 48 h exposure to compound 8 serial dilutions as mentioned at the Experimental section. Morphological signs of cytotoxicity include cell rounding, shrinking and loss of monolayer integrity compared to vehicle control. Total magnifictaion=150×.

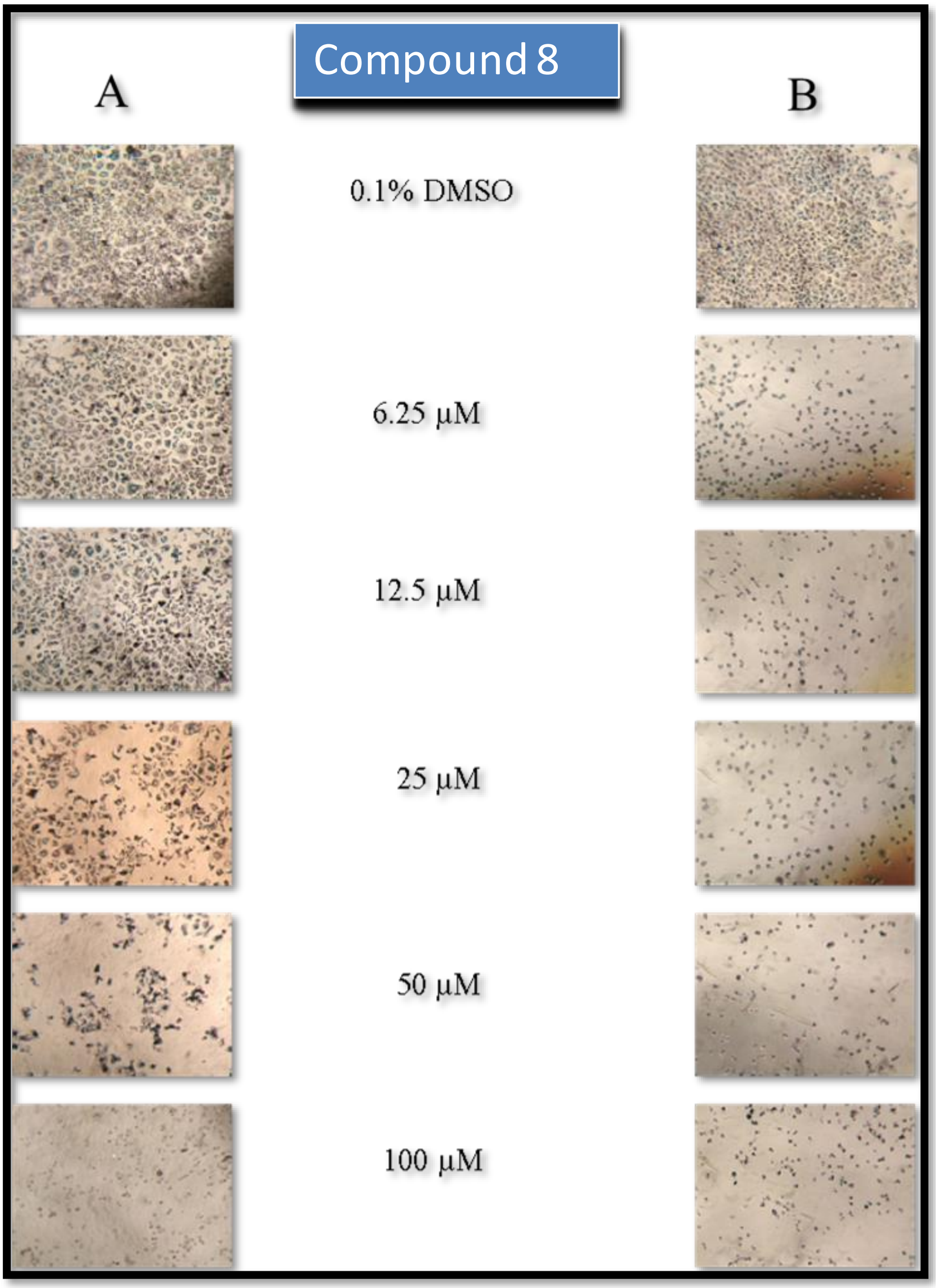

S81. Photomicrographs showing morphological changes of Caco-2 cells (A) or A549 cells (B) following 48 h exposure to compound 9 serial dilutions as mentioned at the Experimental section. Morphological signs of cytotoxicity include cell rounding, shrinking and loss of monolayer integrity compared to vehicle control. Total magnifictaion=150×.

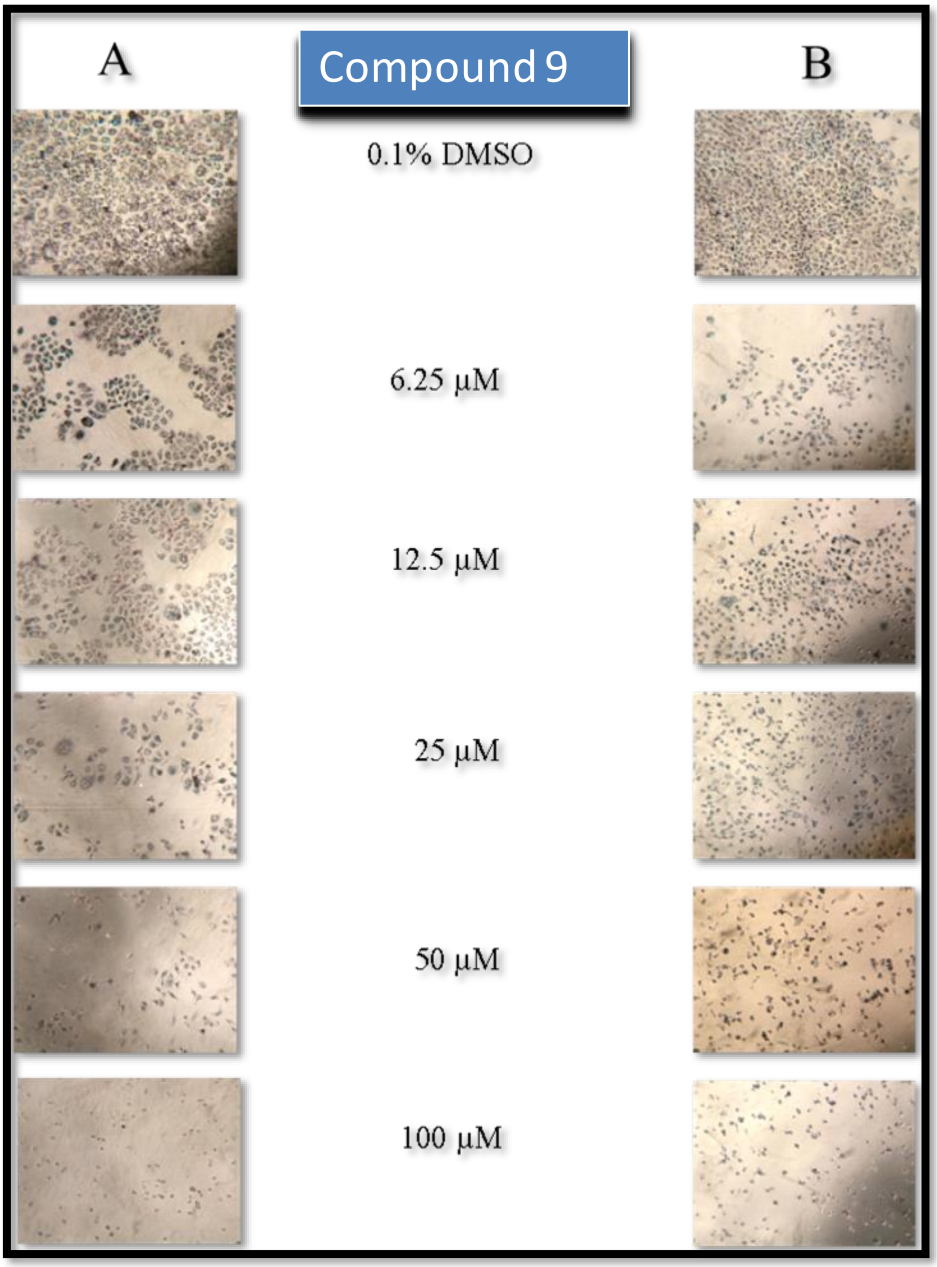

S82. Photomicrographs showing morphological changes of Caco-2 cells (A) or A549 cells (B) following 48 h exposure to compound 10 serial dilutions as mentioned at the Experimental section. Morphological signs of cytotoxicity include cell rounding, shrinking and loss of monolayer integrity compared to vehicle control. Total magnifictaion=150×.

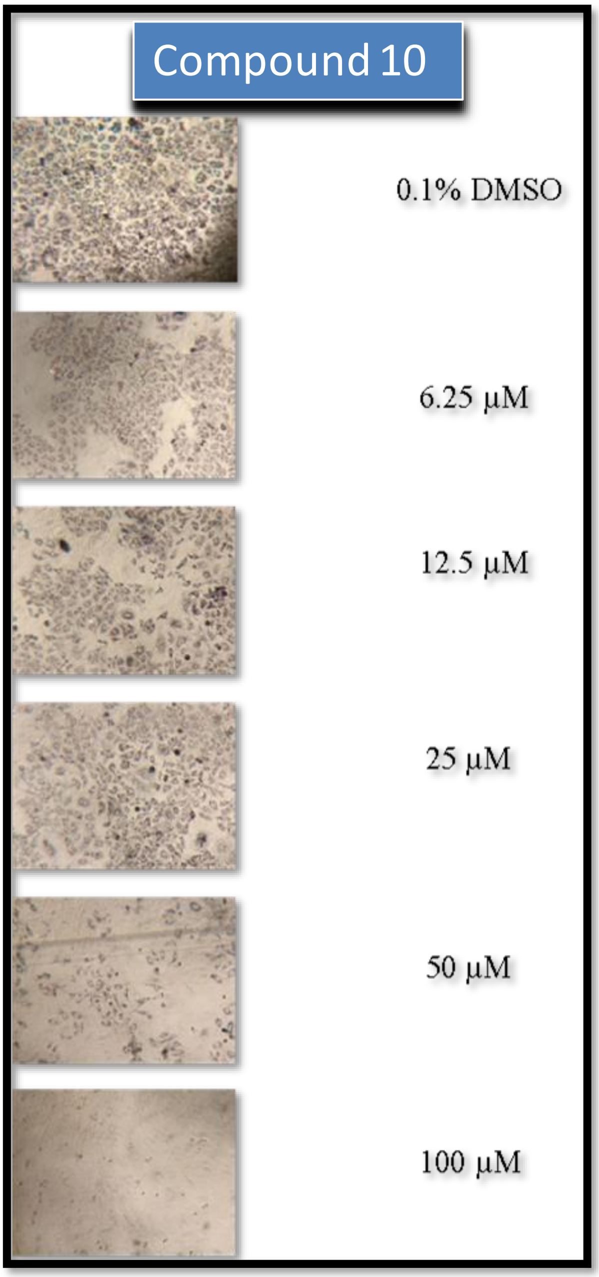

S83. Photomicrographs showing morphological changes of Caco-2 cells following 48 h exposure to compound 11 serial dilutions as mentioned at the Experimental section. Morphological signs of cytotoxicity include cell rounding, shrinking and loss of monolayer integrity compared to vehicle control. Total magnifictaion=150×.

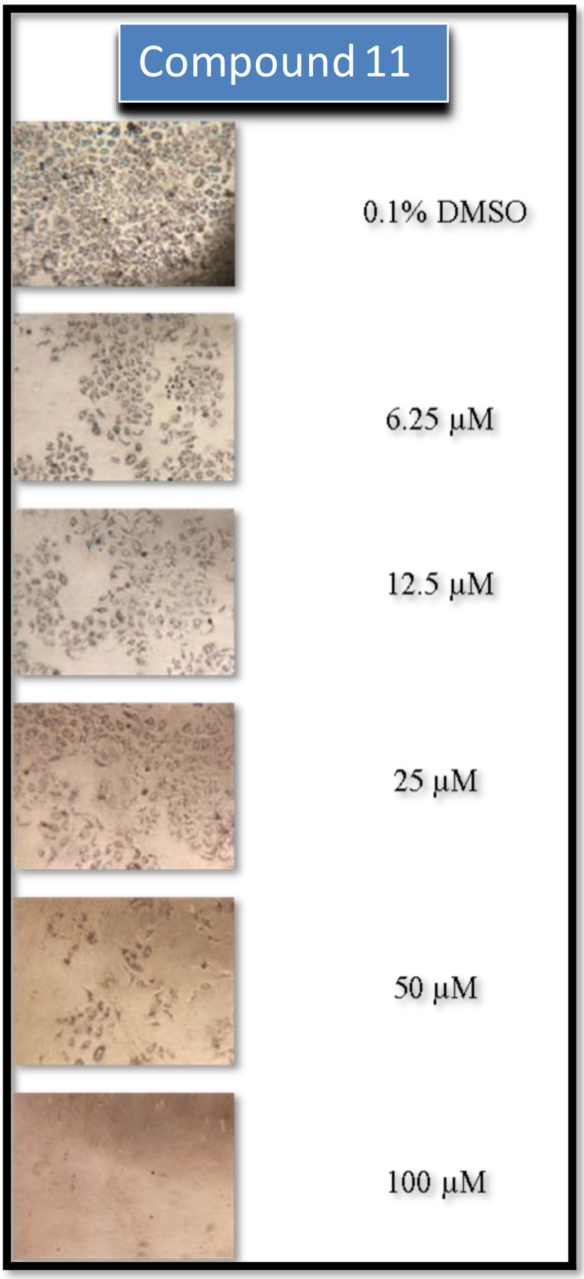

S84. Photomicrographs showing morphological changes of Caco-2 cells following 48 h exposure to compound 12 serial dilutions as mentioned at the Experimental section. Morphological signs of cytotoxicity include cell rounding, shrinking and loss of monolayer integrity compared to vehicle control. Total magnifictaion=150×.

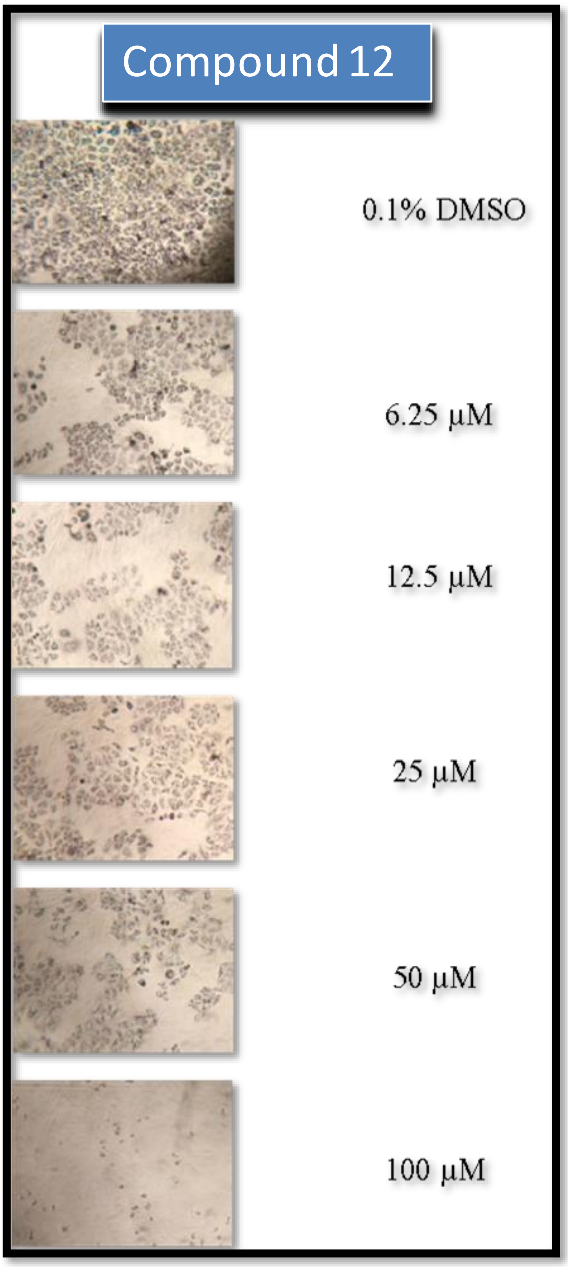

S85. Photomicrographs showing morphological changes of Caco-2 cells following 48 h exposure to compound 13 serial dilutions as mentioned at the Materials and Methods section. Morphological

signs of cytotoxicity include cell rounding, shrinking and loss of monolayer integrity compared to vehicle control. Total magnifictaion=150×.

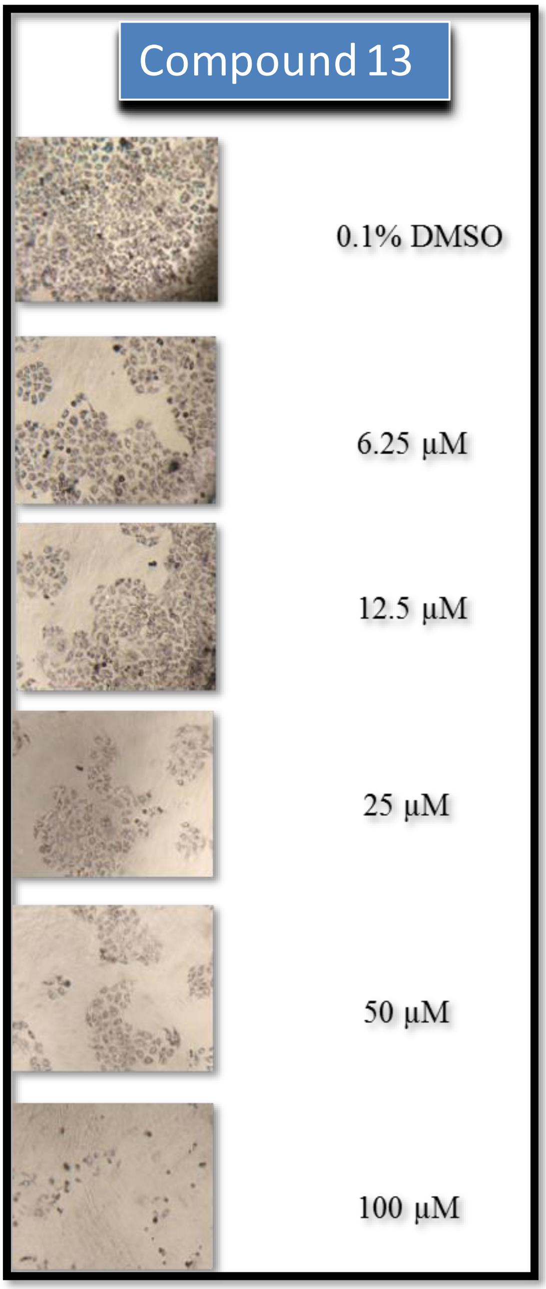

S86. Photomicrographs showing morphological changes of A549 cells following 48 h exposure to serial dilutions of doxorubicin HCl as mentioned at the Materials and Methods section. Morphological signs of cytotoxicity include cell rounding, shrinking and complete loss of monolayer integrity compared to vehicle control. Total magnifictaion=150×.

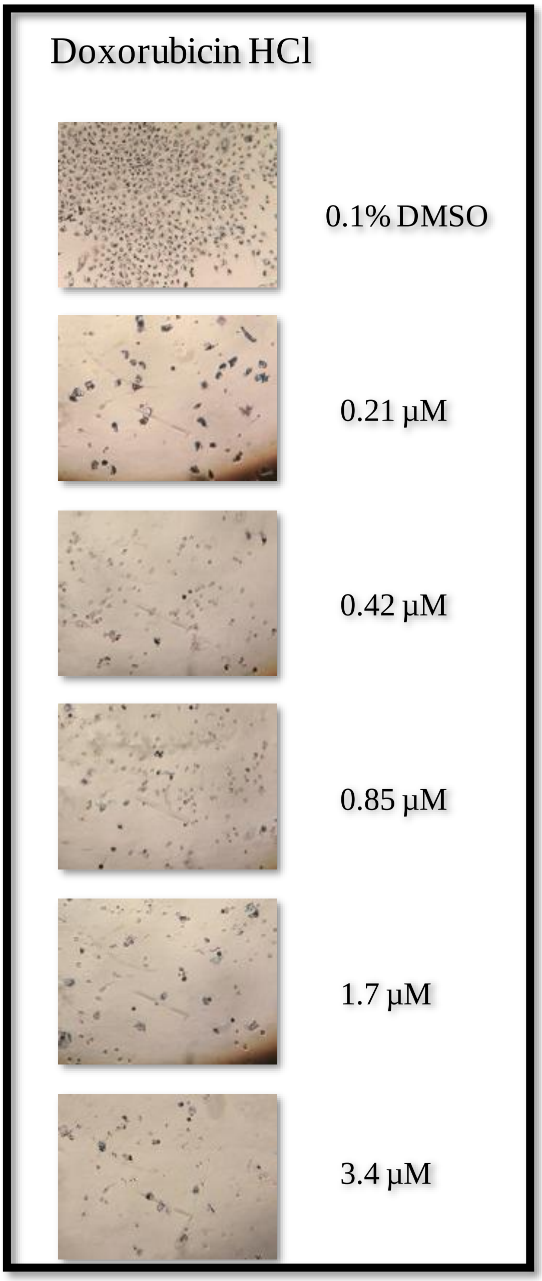

S87. Photomicrographs showing morphological changes of Caco-2 cells following 48 h exposure to serial dilutions of doxorubicin HCl as mentioned at the Materials and Methods section. Morphological signs of cytotoxicity include cell rounding, shrinking and complete loss of monolayer integrity compared to vehicle control. Total magnifictaion=150×.

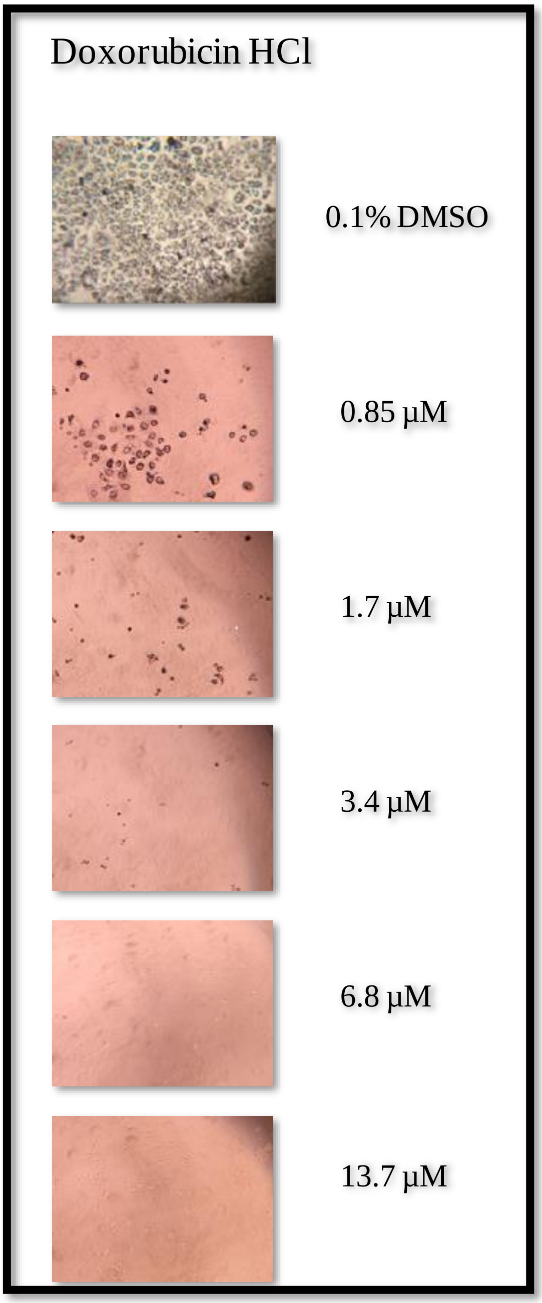

Supplement: Supplementary file 1 [file molecules-23-02221-s001.pdf]
